# Light-triggered oxygen redox activity at the edge of cobalt oxyhydroxide for superior water oxidation

**DOI:** 10.1038/s41467-026-74386-1

**Published:** 2026-06-11

**Authors:** Xin Zhang, Qiyun Wang, Qi Zhang, Haoyin Zhong, Chao Wu, Baorui Jia, Junchen Yu, Ke-Jin Zhou, Yuanjie Li, Yong-Wei Zhang, Zhi Gen Yu, Shibo Xi, Xiaopeng Wang, Junmin Xue

**Affiliations:** 1 Department of Materials Science and Engineering, National University of Singapore, Singapore, Singapore; 2 College of Materials Science and Engineering, Sichuan University, Chengdu, China; 3 Institute of Sustainability of Chemical, Energy and Environment (ISCE), Agency for Science, Technology and Research (A*STAR), Singapore, Singapore; 4 Institute for Advanced Materials and Technology, University of Science and Technology Beijing, Beijing, China; 5 Diamond Light Source, Harwell, UK; 6 Dongfang Electric (Fujian) Innovation Institute Co., Ltd., Fujian, China; 7 Institute of High Performance Computing (IHPC), Agency for Science, Technology and Research (A*STAR), Singapore, Singapore; 8 State Key Laboratory of Intelligence Construction and Healthy Operation and Maintenance of Deep Underground Engineering, Sichuan University, Chengdu, China

**Keywords:** Electrocatalysis, Electrocatalysis, Hydrogen energy, Electrocatalysis

## Abstract

Introducing oxygen redox chemistry into cobalt oxyhydroxide effectively enhances catalytic activity by enabling direct O-O coupling, thereby bypassing the rate-limiting ^*^OOH step in the conventional adsorbate evolution mechanism. However, the key challenge is to preserve the accessibility of non-bonding oxygen states while maintaining cobalt-oxygen covalency. Here we show that light irradiation triggers ligand-to-metal charge transfer in sulfur-treated cobalt oxyhydroxide (S-CoOOH), generating non-bonding oxygen states. These states then couple with adjacent ones to form direct O-O bonds. Through this way, the sulfur-treated sample performs enhanced OER activity under light, achieving an overpotential of 194 ± 3 mV at 10 mA cm^−2^, which is 41 mV lower than in the dark. Further analysis reveals that light-induced oxygen redox activity is confined to the edge of catalyst. This activity originates from electron transitions from (M-O) to non-overlapping regions of Co 3 *d* and 4*p* orbitals, driven by high-spin Co^3+^ at the edge. This work highlights the critical role of light in inducing non-bonding oxygen states in transition metal-based catalysts and guides the development of oxygen-redox electrocatalysts.

## Abstract (web summary)

Light-driven oxygen redox offers a new strategy to boost oxygen evolution activity by enabling direct O–O bond formation. Here, the authors report that sulfur-treated cobalt oxyhydroxide generates non-bonding oxygen states under light, enhancing oxygen evolution performance.

## Introduction

The oxygen evolution reaction (OER) is a pivotal anodic half-reaction for oxygen production, often coupled with hydrogen evolution or carbon dioxide reduction in water splitting and artificial photosynthesis, both essential for sustainable energy and environmental technologies^1–3^. Nonetheless, OER encounters a bottleneck due to its sluggish kinetics, involving multiple proton-coupled electron transfer processes, which lead to substantial energy losses^4,5^. To overcome this challenge, the development of low-cost and high-performance transition-metal-based electrocatalysts is crucial for accelerating electron transfer and improving OER efficiency^6,7^. According to the conventional adsorbate evolution mechanism (AEM), OER proceeds via multiple adsorbed intermediates on metal active sites^8^. In this mechanism, the scaling relation between two key intermediates ^*^OH and ^*^OOH gives rise to the limitation on OER activity^9,10^. Recently, the introduction of oxygen redox activity has been proven effective in bypassing the strongly correlated adsorption of intermediates, specifically avoiding the rate-determining step of ^*^OOH formation in AEM^11,12^. This mechanism operates through oxygen redox instead of metal redox in AEM, enabling a direct oxygen-oxygen bond^13,14^. The accessibility of non-bonding oxygen (*O*_NB_) states is thus crucial for achieving oxygen redox activity^15^. However, the main challenge remains generating *O*_NB_ states while maintaining the structural integrity of the metal-oxygen octahedron^16^.

Cobalt oxyhydroxide (CoOOH) is widely recognized as an ideal OER catalyst due to its low cost, natural abundance, excellent catalytic activity, and robust structural stability^17–19^. To date, activating oxygen redox activity in CoOOH remains challenging, with only a few studies incorporating low-valent metal ions (e.g., Zn^2+^, Cu^2+^, Ag^+^) into CoOOH (Co^3+^) to introduce *O*_NB_ states and facilitate direct O-O coupling^8,20,21^. However, this doping strategy generates *O*_NB_ states in the bulk of CoOOH, significantly sacrificing Co-O hybridization, and ultimately causing bulk collapse and CoO_6_ octahedral destabilization^8,16^. Our previous study demonstrated that light irradiation could trigger reversible crystal distortion in nickel-based oxyhydroxide, enabling oxygen redox activity through a mechanism termed the coupled oxygen evolution mechanism (COM)^22^. Motivated by this finding, we sought to investigate whether light irradiation could activate oxygen redox activity in cobalt-based oxyhydroxide, offering an alternative to conventional doping strategies. *Operando* X-ray absorption spectroscopy (XAS) confirms that light irradiation triggers ligand-to-metal charge transfer (LMCT) in sulfur-treated cobalt oxyhydroxide (S-CoOOH), where electron transitions from (M-O) to Co 3 *d* orbitals, leading to the formation of *O*_NB_ states. Further analysis reveals that these neighboring *O*_NB_ states couple to form direct O-O coupling, with oxygen redox activity occurring at the edges of CoOOH. As a result, upon exposure to light irradiation, S-CoOOH shows improved OER performance (41 mV reduction in overpotential at 10 mA cm^−2^), whereas the reference CoOOH sample without sulfurization treatment (R-CoOOH) exhibits no variation. Finally, project density of states (PDOS) simulations and resonant inelastic X-ray scattering (RIXS) reveal that the light-induced enhancement in OER performance originates from the emergence of non-overlapping regions between Co 3 *d* and 4 *s* orbitals, driven by high-spin Co^3+^ at the edge of S-CoOOH. This work underscores the significance of applying light stimuli to induce *O*_NB_ states, and provides a guide for designing highly efficient and stable OER electrocatalysts.

## Results

### Effect of light on OER activity

**Fig. 1:**
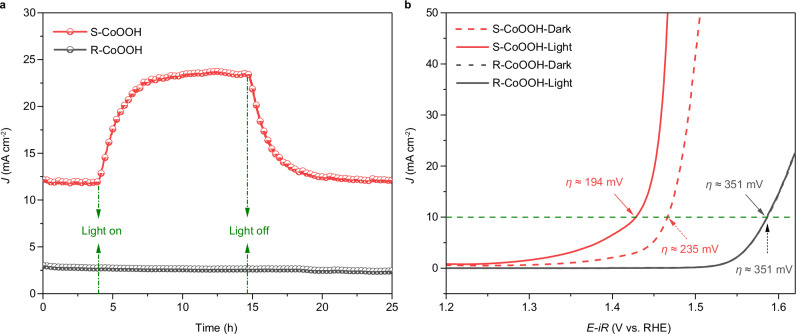
OER activities of R-CoOOH and S-CoOOH with and without light irradiation. **a** Light-response test of R-CoOOH and S-CoOOH using chronoamperometry measurement at a potential of 1.55 V and 1.45 V versus a reversible hydrogen electrode (RHE), respectively. The light source was supplied with a 300 W xenon lamp equipped with an AM 1.5 G filter. **b** OER polarization curves of R-CoOOH and S-CoOOH with 90% *iR* correction in the dark (dashed line) and under light (solid line) (scan rate = 1 mV s^−1^, pH = 13.92 ± 0.02, and electrode area = 1 × 2 cm^−2^), at room temperature (300 K) in 1 M KOH electrolyte using Pt as the counter electrode and Hg/HgO as the reference electrode. The corresponding non-*iR* corrected curves and resistance values are provided in Supplementary Figs. S6 and 16. The horizontal dashed line marks a current density of 10 mA cm^−2^ for evaluating the overpotential (*ŋ*).

Two types of CoOOH were used in our work: the first, designated as S-CoOOH, was derived from the oxidation of cobalt sulfides, featuring unsaturated coordination at the edge of CoOOH^17^. The cobalt sulfides were completely reconstructed into β-CoOOH (R-3m space group, JCPDS 07-0169), leaving negligible residual sulfur, as confirmed by X-ray diffraction (XRD), X-ray photoelectron spectroscopy (XPS), Co *K*-edge XAS, and inductively coupled plasma (ICP) measurements (Supplementary Figs. S1, 2 and Table S1). The second form, standard β-CoOOH, was produced via hydrothermal synthesis and served as the reference, referred to as R-CoOOH. Figure 1 demonstrates that S-CoOOH exhibits better OER activity under light irradiation, with its current density increasing from 11.75 mA cm^−2^ to 23.86 mA cm^−2^ over approximately 8 hours. Upon cessation of light exposure, the current density returns to 12.05 mA cm^−2^, which closely approximates its initial level observed before irradiation, down from 23.43 mA cm^−2^ within 7 h (Fig. 1a). In stark contrast, R-CoOOH displays negligible changes in current density under identical light irradiation conditions (Fig. 1a). Furthermore, the repeatability of light-induced behavior in S-CoOOH is verified in Supplementary Fig. S3, demonstrating a prolonged operating duration of six consecutive on/off cycles spanning about 160 hours. This long-term repeatability excludes the possibility of structural reconstruction. Moreover, linear sweep voltammetry (LSV) curves in Fig. 1b demonstrate that under light, S-CoOOH achieves a current density of 10 mA cm^−2^ at 1.424 V, corresponding to an overpotential of 194 ± 3 mV after 90 % current resistance (*iR*) correction, which is 41 mV lower than in the dark (235 ± 5 mV). This performance is competitive among reported cobalt-based catalysts (Supplementary Table S2). In contrast, R-CoOOH exhibits negligible enhancement in OER activity, with an overpotential of 351 mV under light compared to 352 mV in the dark (Fig. 1b). Additional electrochemical characterization results are provided in Supplementary Figs. S4–6. The photon response of different light intensities and wavelengths is not related to a conventional bandgap excitation, as further detailed in Supplementary Figs. S7, 8.

Initially, several potential reasons for the distinctive increase in OER performance under light illumination are excluded, including thermal effect (22.7 °C–23.3 °C across the entire catalyst layer), changes in grain size, generation of light-induced holes, or any additional reaction (Supplementary Figs. S9–S15). Besides, the Nyquist plots recorded under light and dark nearly overlap, ruling out any electrochemical potential shifts or changes in the equilibrium interfacial potential (Supplementary Fig. S16). Furthermore, the enhancement of conductivity induced by photocurrent is also ruled out based on potentiostatic electrochemical impedance spectroscopy (PEIS) results, showing no noticeable changes between dark and light conditions collected at different applied voltages (1.35 V, 1.40 V, 1.45 V, and 1.50 V vs. RHE) (Supplementary Fig. S17). Additionally, structural reconstruction is also excluded based on pre- and post-reaction Co *K*-edge XAS spectra, XRD patterns, and TEM images, as all results before and after OER under light irradiation remain nearly identical (Supplementary Figs. S18–20).

### Ligand-to-metal charge transfer under light

**Fig. 2:**
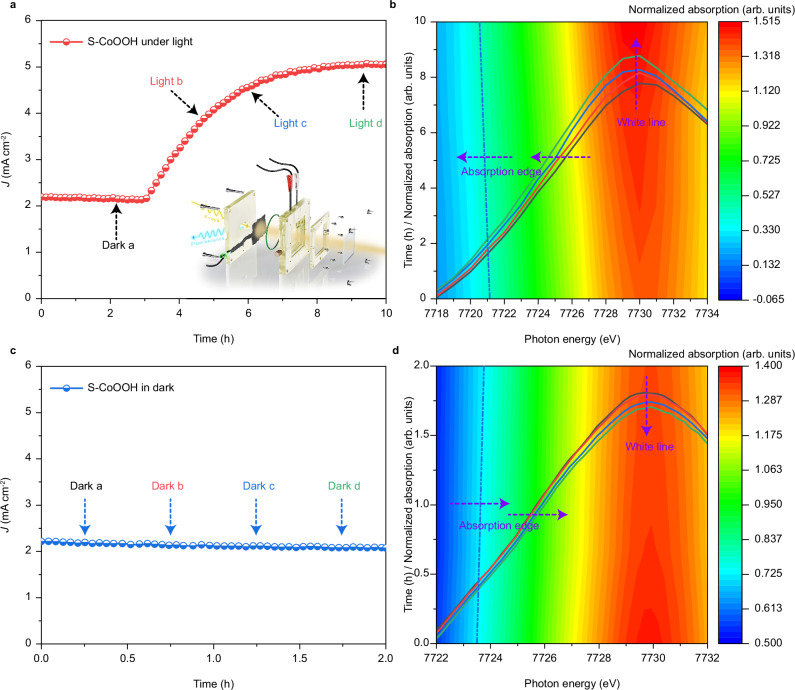
Identification of *O*_NB_ states formation in S-CoOOH subjected to light irradiation. **a** Electrochemical curve for *operando* Co *K*-edge XAS measurements recorded during OER from dark to illumination at four selected points: Dark a, Light b, Light c, and Light d. The inset is a schematic of the *operando* XAS setup, with a carbon rod as the counter electrode and Ag/AgCl as the reference electrode. **b** *Operando* Co *K*-edge XAS spectra of S-CoOOH during OER under light, with dashed purple lines revealing spectral shifts. **c** Electrochemical curve for *operando* Co *K*-edge XAS measurements in the dark, recorded during the chronoamperometry (CA) process at four selected points: Dark a, Dark b, Dark c, and Dark d. **d** *Operando* Co *K*-edge XAS spectra of S-CoOOH during OER in the dark, with dashed purple lines indicating spectral shifts.

Next, it is hypothesized that light-induced enhancement in OER performance of S-CoOOH arises from a shift in OER mechanism, which was investigated using *operando* Co *K*-edge XAS in a customized electrochemical cell designed to simulate real OER conditions (the inset of Fig. 2a and Supplementary Figs. S21, 22). Figure 2a presents the electrochemical data collected during this *operando* experiment from dark to illumination. As illustrated in the XAS results (Fig. 2b and Supplementary Fig. S23), the absorption edge (7718 to 7725 eV) of S-CoOOH exhibits a redshift (marked with purple arrows) under light compared to darkness, and this redshift intensifies with prolonged light exposure. The redshift in photon energy reflects a reduction in Co valence states, with an increase in the 3 *d* orbital occupancy. The redshift observed under light is consistent with our earlier findings in NiOOH, where light triggers a decrease in Ni valence from Ni^3+^ to Ni^2+^^22^. Based on this, light induces a reduction in Co valence from +3 to +2. In contrast, *Operando* XAS measurement under identical OER conditions in darkness (Fig. 2c) exhibits a gradual blueshift in absorption edge (Fig. 2d and Supplementary Fig. S24), which differs from the behavior under light but aligns with the conventional Co transition between CoOOH (+3) and CoO_2_ (+4) during OER^5^.

Based on this difference in the adsorption edge under illumination (${{\rm{Co}}}^{3+}\leftrightarrow {{\rm{Co}}}^{2+}$) versus darkness (${{\rm{Co}}}^{3+}\leftrightarrow {{\rm{Co}}}^{4+}$), it is inferred that the observed reduction of partial Co under light results from ligand-to-metal charge transfer (LMCT, M-O band to Co 3 *d* orbital)^23,24^. Here, the light-triggered LMCT proceeds by preferentially removing electrons from Co 3 *d* states (metal) to form an excited state, which then de-excites to the ground state by taking electrons from (M-O) (ligand), thereby partially oxidizing O and returning Co to a lower oxidation state^23^. As a result, *O*_NB_ states featuring seven outermost electrons are formed during this OER process when exposed to light irradiation. Importantly, the LMCT involved here is not a conventional photophysical LMCT, but rather a step of OER processes that is coupled to chemical bond formation and thus cannot be detected by transient absorption spectroscopy (Supplementary Fig. S25). Further evidence for LMCT is provided by the combination of *operando* Co *K*-edge XAS and subsequent TMAOH chemical probe experiments, which together reveal a decrease of cobalt (electron transfer to metal) and the presence of *O*_NB_ states (electron transfer from ligand).

Moreover, *operando* XAS results for S-CoOOH demonstrate intensification of the while line with prolonged light irradiation (Fig. 2b), suggesting a more regular CoO_6_ octahedral structure under light without any phase transformation. The trend is contrary to our prior findings on NiOOH nanoribbon (NR-NiOOH)^22^, a discrepancy attributed to the distinct electron configurations of Co^2+^ (${3d}^{7}$) and Ni^2+^ (${3d}^{8}$)^25^, as discussed in Supplementary Fig. S26. The increased white-line intensity and reversibility indicate that there is no change in translational and time-reversal symmetry (Supplementary Fig. S26 and Fig. S3). The atomic coordination numbers (CNs) of Co-O and Co-Co are fitted based on the *operando* Co *K*-edge Fourier-transformed extended X-ray absorption fine structure (FT-EXAFS) spectra of S-CoOOH. As illustrated in Supplementary Fig. S27 and Table S3, it is revealed that the CNs of Co-O and Co-Co for S-CoOOH increase progressively with prolonged light irradiation. Besides, no distinct changes in Co-O or Co-Co bond lengths are observed upon irradiation, suggesting no structural strain.

### Verification of oxygen redox activity under light

**Fig. 3:**
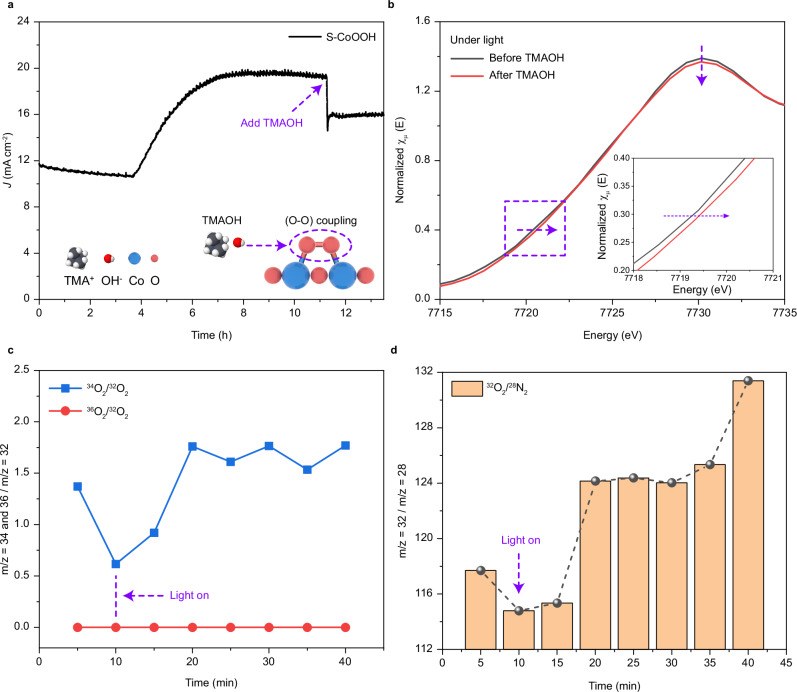
Confirmation of *O*_NB_ states participation in the redox activity subjected to light irradiation. **a** The electrochemical curves respond to the addition of TMAOH under light, where the inset is the schematic diagram of the interaction between TMA^+^ cations and *O*_NB_ states. **b** Co *K*-edge XAS spectra of S-CoOOH subjected to light irradiation before and after adding TMAOH. **c**,**d** ^18^O isotope labeling experiments. To minimize the impacts of residual gas in the electrochemical cell and tube on the gas chromatography-mass spectrometry (GC-MS) analysis under 3 mL/min N_2_ flow, the average ratio of ^34^O_2_ to ^32^O_2_ and ^36^O_2_ to ^32^O_2_ (**c**), as well as ^32^O_2_ to ^28^N_2_ (**d**) were measured every 300 s.

According to the above discussion, light is verified to trigger electron transfer from the M-O bond to the Co 3 *d* orbital (that is, LMCT), thereby generating *O*_NB_ states with seven outermost electrons during OER under light. These *O*_NB_ states are regarded as the critical species for oxygen redox activity, which might enable direct O-O coupling^8^. Here, whether O-O coupling actually takes place when exposed to light was investigated using a tetraalkylammonium (TMA^+^) probe and ^18^O isotope labeling measurements. As depicted in the inset of Fig. 3a, TMA^+^ cation exhibits a specific interaction with *O*_NB_ states, thereby blocking the formation of O-O coupling^26^. If OER kinetics are inhibited upon adding TMA^+^, it signifies the presence of O-O coupling. Figure 3a demonstrates a significant reduction in the OER activity under light following the addition of TMA^+^, contrasting with the stable OER activity of S-CoOOH in the dark after adding TMA^+^ (Supplementary Fig. S28a). This discrepancy implies O-O coupling is formed exclusively upon exposure to light. Besides, the TMA^+^ probe combined with Co *K*-edge XAS can reveal changes in the valence state of Co upon adding TMA^+^. When O-O coupling is present, the addition of TMA^+^ triggers an inner electron transfer from the Co atom to TMA^+^, leading to an increase in cobalt valence^22^. As shown in the Co *K*-edge spectra of S-CoOOH before and after adding TMA^+^ (Fig. 3b), a blueshift occurs under light, signifying an increase in cobalt valence, while no detectable change is observed for S-CoOOH in the dark upon TMA^+^ addition (Supplementary Fig. S28b). These results support the formation of O-O coupling exclusively under light, with light acting as the sole driving force for this light-induced LMCT process.

Furthermore, ^18^O isotope labeling measurements provide additional evidence of oxygen redox activity in OER under light. Details of the procedure are seen in the Methods section. As shown in Fig. 3c, the ratio of ^34^O_2_/^32^O_2_ initially decreases in the dark, but exhibits a sharp increase upon light illumination. The decrease in the ^34^O_2_/^32^O_2_ ratio in the dark is attributed to the gradual consumption of previously absorbed ^18^O-labeled ^*^OH during the formation of ^*^OOH from two adsorbed ^*^OH, which aligns with the trend reported in AEM^27^. At the 10th minute, when light is turned on, the abrupt increase in the ^34^O_2_/^32^O_2_ ratio suggests that more ^18^O-labeled oxygen within the catalyst actively participates in the OER process, thereby confirming oxygen redox activity under light. Besides, the ^36^O_2_/^32^O_2_ ratio remains consistently near zero in both dark and illuminated conditions, possibly due to substantial consumption of ^18^O-labeled oxygen during the initial dark phase. To further validate this hypothesis, subsequent *operando* isotope measurements were conducted under fully dark and fully illuminated conditions.

In addition, to further substantiate our ^18^O isotope analysis, we compared the OER kinetics under both dark and illuminated conditions. Figure 3d presents the ratio of ^32^O_2_ to ^28^N_2_ collected over a specific period, serving as an indicator of the oxygen production rate of S-CoOOH during the OER process. Gas samples were taken at five-minute intervals, starting with the first 15 min in darkness, followed by light exposure from 15 to 40 min. The ratios of ^32^O_2_ to ^28^N_2_ measured every five minutes between 15 and 40 min are consistently higher than those recorded during the initial 15 min, demonstrating an accelerated rate of oxygen production under illumination compared to darkness.

### Sites of oxygen redox activity

**Fig. 4:**
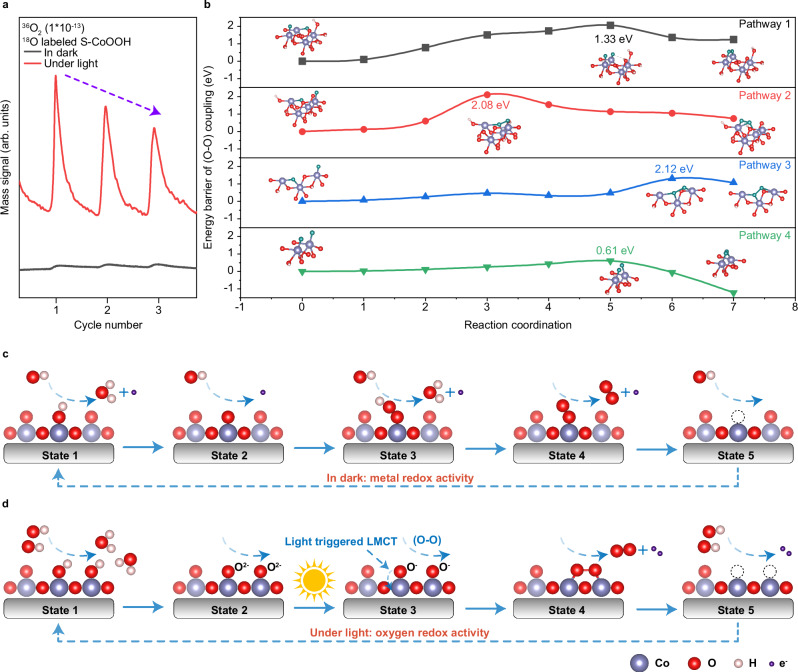
Determination of sites of oxygen redox activity. **a** Differential electrochemical mass spectrometry (DEMS) signals of O_2_ products for S-CoOOH measured with and without light irradiation. **b** Energy profile diagram of O-O coupling involving oxygen atoms located either in the bulk or at the edge. The inset shows the proposed O-O coupling occurring at different oxygen sites, referred to as Pathways 1-4, where oxygen atoms marked in green represent the atoms involved in O-O coupling. **c** Electron transfer pathway of S-CoOOH in the dark, where cobalt acts as a redox center. **d** Electron transfer pathway of S-CoOOH under light, where oxygen serves as a redox center with ligand-to-metal charge transfer (LMCT) triggered *O*_NB_ states formation.

To further identify the sites of oxygen redox activity under light, specifically whether these processes occur at the catalyst edge or within its bulk, *operando* differential electrochemical mass spectrometry (DEMS) coupled with ^18^O isotope labeling measurements was carried out on S-CoOOH under both dark and light conditions for comparison. Details of the procedure are provided in the Methods section. No ^36^O_2_ signal observed in the dark (Fig. 4a) indicates S-CoOOH follows metal redox center AEM under dark conditions (Supplementary Figs. S29, 30). In contrast, if oxygen redox activity exists, ^36^O_2_ should be detected (Supplementary Figs. S31, 32). Additionally, the site of oxygen redox activity is related to the variation in ^36^O_2_ content over time. Specifically, if a direct O-O coupling occurs within the CoO_6_ lattice; the ^36^O_2_ content would remain relatively stable over time due to the abundant ^18^O-labeled oxygen within the CoO_6_ lattice^28^. However, if an O-O coupling takes place at the edges of S-CoOOH, the ^36^O_2_ ratio would gradually decrease over time, as the limited residual ^18^O at the edge is consumed^27^. As shown in Fig. 4a and Supplementary Fig. S33, the ^36^O_2_ signal is evidently discernible under illumination and decreases over time. These *operando* DEMS findings confirm that S-CoOOH exhibits oxygen redox activity occurring at the edge of S-CoOOH under light. This further supports the hypothesis proposed above in Fig. 3c.

Next, calculations were carried out to further determine whether oxygen redox activity under light occurs at the edge or within the bulk of S-CoOOH. The S-CoOOH model was constructed based on our previous report^17^, featuring 4-coordinated Co at the edge and 6-coordinated Co in the bulk. Considering light-triggered *O*_NB_ states in S-CoOOH, the overall charge was adjusted, yielding an optimized S-CoOOH model (Supplementary Figs. S34, 35). Based on this model, the energetic profiles of four proposed *O*_NB_-mediated O-O coupling pathways at different oxygen sites, namely Pathways 1-4, were calculated. As depicted in the inset of Fig. 4b, Pathways 1-3 all involve one lattice oxygen and one edge oxygen atom, with the primary difference being the distance between two cobalt atoms bonded to the oxygen atoms involved in O-O coupling. In contrast, Pathway 4 exclusively features edge oxygen atoms in O-O coupling. Our results show that when oxygen redox activity occurs exclusively at the edge of S-CoOOH (Pathway 4), the energy barrier is 0.61 eV, which is significantly lower than those for the coupling of lattice oxygen and edge oxygen in Pathways 1-3 (1.33, 2.08, or 2.12 eV). Moreover, thermodynamic calculations under dark and light conditions suggest that the OER pathway under light exhibits a smaller theoretical overpotential than that in dark (Supplementary Figs. S36, 37), with the potential-determining step shifting from ^*^OOH formation in dark to proton transfer under light. Additional simulation results are provided in the Supplementary Information (Supplementary Fig. S38 and Table S4).

Based on the aforementioned *operando* XAS, *operando* DEMS, and TMAOH chemical probe experiments, the OER electron pathway of S-CoOOH shifts from metal redox activity in dark to oxygen redox activity under light, with neighboring *O*_NB_ states at its edge undergoing O-O coupling. The above comparative experiments under both dark and light conditions confirm that light is the sole driving force for LMCT. The comparison of electron transfer pathways in S-CoOOH under dark and illuminated conditions is presented in Fig. 4c,d, and the light-induced processes are illuminated as follows: 1) Initially, Co^3+^ bonded ^*^OH species (where ^*^ refers to the metal sites) undergo deprotonation upon interaction with an adsorbed ${{\rm{OH}}}^{-}$ species from the electrolyte, resulting in the formation of ${{\rm{O}}}^{2-}$. 2) Next, light-triggered LMCT coupled to OER occurs (State 3 in Fig. 4d), involving an electron transfer from (M-O) to Co 3 *d* orbitals, thereby generating *O*_NB_ states with seven outermost electrons (${{\rm{O}}}^{-}$). 3) These two adjacent ${{\rm{O}}}^{-}$ species at the edge of S-CoOOH then combine directly to yield O-O coupling. 4) Finally, O-O coupling is deprotonated and transformed into an O_2_ molecule. 5) The evolution of O_2_ molecule creates two oxygen vacancies, which are later filled by the adsorption of two ${{\rm{OH}}}^{-}$ from the electrolyte. In contrast, S-CoOOH follows the traditional AEM pathway in dark conditions, proceeding via ^*^OH adsorption, ^*^OOH formation, and O_2_ release. The primary distinction between the electron transfer pathways under light and in darkness centers on the oxygen intermediates. In the dark, ${{\rm{O}}}^{2-}$ adsorbs ${{\rm{OH}}}^{-}$ to yield ^*^OOH, whereas under light, ${{\rm{O}}}^{2-}$ is oxidized to form ${{\rm{O}}}^{-}$, which then couples with another to form O-O coupling. This light-triggered oxygen redox pathway, involving direct O-O coupling, bypasses the rate-determining step of ^*^OOH formation in AEM. Besides, this oxygen redox activity happens at the edge of S-CoOOH, preserving cobalt-oxygen covalency without sacrificing the integrity of the octahedral structure.

Briefly, the mechanism of light-triggered catalytic enhancement in S-CoOOH is related to the change in the OER reaction mechanism, illustrated in Fig. 4c, d. Light irradiation triggers electron transfer from oxygen (ligand) to cobalt (metal) in S-CoOOH, leading to a reduction in cobalt valence and the formation of *O*_NB_ states; then, two neighboring *O*_NB_ states couple to form direct O-O coupling, thereby bypassing the rate-determining step of ^*^OOH formation in the dark metal-redox pathway. As such, light shifts the redox activity from metal to oxygen, which is the reason for the enhancement of catalytic activity. To further clarify the light-matter interaction, Fig. 4d (State 3) depicts the underlying photoexcitation that in our work is coupled to OER: Upon light illumination, electrons are transferred from oxygen to cobalt, generating *O*_NB_ states that are stabilized via coupling with the neighboring *O*_NB_ during O-O bond formation. Accordingly, the light carriers represent a special Co-O octahedral framework, composed of reduced Co centers and O-O coupled oxygen species, which are spatially confined and do not exhibit mobility.

### The origin of light-triggered LMCT

**Fig. 5:**
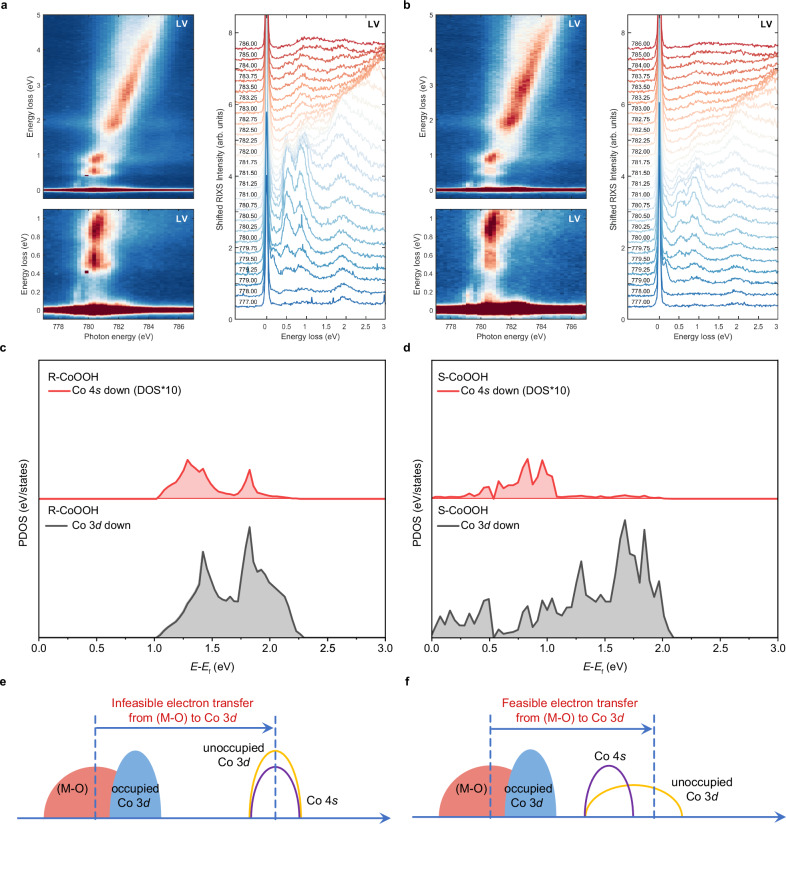
Differences in the electronic structures of R-CoOOH and S-CoOOH. **a** Co 2p3d RIXS spectra of R-CoOOH. **b** Co 2p3d RIXS spectra of S-CoOOH. **c** PDOS of cobalt 3 *d* and 4 *s* orbitals (spin-down) in R-CoOOH. **d** PDOS of cobalt 3 *d* and 4 *s* orbitals (spin-down) in S-CoOOH. **e** Schematic diagram of infeasible electron transfer in R-CoOOH, with a complete overlapping region between Co 3 *d* and 4 *s* orbitals. **f** Schematic diagram of feasible electron transfer in S-CoOOH, with a distinct non-overlapping region between Co 3 *d* and 4 *s* orbitals.

Finally, the rationale behind the occurrence of O-O coupling at the edge of S-CoOOH is addressed. The formation of *O*_NB_ states is the fundamental basis for initiating oxygen redox activity, which in our case arises from light-triggered electron excitation via LMCT (M-O bond to Co 3 *d* orbital). Consequently, the key question becomes why LMCT only occurs in S-CoOOH, specifically confined to its edge, whereas no such LMCT is observed in R-CoOOH. To investigate this, fine electronic structures of both R-CoOOH and S-CoOOH were studied, using Co 2p3d resonant inelastic X-ray scattering (RIXS) technique (Fig. 5a, b). According to previously reported calculations on ground state and excited states energies of Co^3+^^29^, the 0.2 eV peak in energy loss spectra corresponds to the energy excitation from ^1^A_1g_ ground state to ^5^T_2g_ excited state. The ^5^T_2g_ state (S = 2) is typically associated with a high-spin configuration of Co^3+^ (${t}_{2{\rm{g}}}^{4}{e}_{{\rm{g}}}^{2}$). The observed peak (0.2 eV) in S-CoOOH (Fig. 5b) is slightly higher than that of R-CoOOH (Fig. 5a), indicating the presence of high-spin state Co^3+^ in S-CoOOH, consistent with our previous findings^17^.

Additionally, the two peaks at 0.5 eV and 0.9 eV in energy loss spectra represent the energy excitations from ^1^A_1g_ ground state to ^3^T_1g_ and ^3^T_2g_ excited states, respectively. A reduction in the intensity of these peaks suggests enhanced LMCT, reflecting a stronger metal-ligand covalency^30^. As shown in Fig. 5a, b S-CoOOH exhibits a significantly weaker peak intensity at 0.5 eV compared to R-CoOOH, indicating enhanced LMCT and stronger Co-O covalency. This aligns with our previous findings that sulfurization introduces coordinatively unsaturated Co atoms and π-donor oxygen ligands at the edge, resulting in stronger Co-O covalency^17^. The enhanced covalency increases the splitting of Co 3 *d*, 4 *s*, and 4 *p* orbitals, which in turn favors the stabilization of high-spin Co atoms. Our prior work further confirms that the high-spin Co configuration in our S-CoOOH sample is localized exclusively at the edge of S-CoOOH, originating from the coordinatively unsaturated Co atoms present in that region^17^.

As mentioned above, the high-spin configuration is associated with the splitting of Co 3 *d* and 4 *s* orbitals, which is further confirmed by the projected density of states (PDOS). The splitting could create accessible empty states that facilitate LMCT. Specifically, enabling LMCT in CoOOH necessitates the presence of empty antibonding orbitals near the Fermi level, which implies that Co 3 *d* and 4 *s* orbitals should not completely overlap. Figures 5c and d display the PDOS of Co 3 *d* and 4 *s* orbitals in R-CoOOH and S-CoOOH, respectively. For R-CoOOH, the region of Co 4 *s* orbital above the Fermi level significantly overlaps with Co 3 *d* orbitals. This indicates that the electronic states above the Fermi level predominantly exhibit the characteristics of 4 *s* orbital, making this type of light-induced LMCT unfeasible (Fig. 5e). Conversely, S-CoOOH showcases a distinct non-overlapping region between Co 3 *d* and 4 *s* orbitals around the Fermi level, facilitating the possibility of light-induced LMCT (Fig. 5f). Moreover, the calculations show that the estimated energy required for this potential electron transfer from (M-O) to Co 3 *d* orbitals in S-CoOOH (>0.98 eV, <1260 nm) is significantly lower than that in R-CoOOH (>2.05 eV, <605 nm) (Supplementary Fig. S39). This suggests that light-triggered LMCT is more likely to occur in S-CoOOH. Our photon response measurements across different light wavelengths are consistent with this simulation result, showing pronounced current increase at 575 nm and 800 nm, a minimal increase at 1200 nm, and no detectable response at 1310 nm (Supplementary Fig. S8). These findings indicate that photons with an energy of over 1.03 eV (λ < 1200 nm) are required to activate LMCT in S-CoOOH, which is in close agreement with the calculated value of 0.98 eV (1260 nm). The photophysical property of S-CoOOH was further examined by UV-Vis absorption and diffuse reflectance spectra (Supplementary Fig. S40a, b), revealing intense light absorption over 200–2400 nm (0.52–6.20 eV), which provides the required energy to promote electron transfer from (M-O) to Co 3 *d* orbitals (LMCT) in S-CoOOH.

Here, we summarize the reason why oxygen redox activity occurs exclusively in S-CoOOH and is confined to its edge. This behavior originates from the presence of coordinatively unsaturated Co atoms at the edge, which leads to a high-spin configuration and causes the splitting of Co 3 *d* and 4 *s* orbitals, thereby enabling light-triggered LMCT. As these coordinatively unsaturated Co atoms exist only at the edge of S-CoOOH, the resulting *O*_NB_ states generated via LMCT are also localized in this region, and thus oxygen redox activity is restricted to the edge of S-CoOOH.

## Discussion

In summary, a light-induced enhancement in OER performance is observed in sulfur-treated CoOOH (S-CoOOH), with the current nearly doubling and the overpotential at 10 mA cm^−2^ decreasing by 41 mV to 194 ± 3 mV. It stands out as one of the most exceptional OER performances among cobalt-based catalysts. In contrast, no such enhancement is seen in reference CoOOH (R-CoOOH). *Operando* Co *K*-edge XAS confirms that light triggers electron transfer from the M-O bond to the cobalt 3 *d* orbital (that is, LMCT) in S-CoOOH, leading to the formation of *O*_NB_ states. TMA^+^ probe and ^18^O isotope labeling measurements demonstrate oxygen redox activity in S-CoOOH under light, where two neighboring *O*_NB_ states directly couple to form O–O coupling, which bypasses the rate-determining step of ^*^OOH formation in the dark AEM pathway. Moreover, *operando* DEMS along with simulations validates that oxygen redox activity is localized at the edge of S-CoOOH. Finally, RIXS and PDOS elucidate why oxygen redox activity occurs exclusively in S-CoOOH and is confined to its edge. It is because light-triggered LMCT occurs only in non-overlapping Co 3 *d* and 4 *s* orbitals, driven by edge-localized high-spin Co^3+^ in S-CoOOH. The resulting *O*_NB_ states via LMCT are confined at the edge, enabling O-O coupling at these sites. This work elucidates the role of light in enhancing the OER performance of metal oxyhydroxide-based alkaline catalysts, and such a light-induced effect shows promising potential for extension to acidic catalysts that share a similar octahedral structure and to industrial electrolyzers.

## Methods

### Materials and reagents

Unless specifically noted, all materials were purchased from Sigma-Aldrich Co., Ltd. and utilized in their original condition without undergoing further purification. Cobalt sulfate heptahydrate (CoSO_4_·7H_2_O, CAS 10026-24-1, Purity 99%), cobalt nitrate hexahydrate (Co(NO_3_)_2_·6H_2_O, 10026-22-9, 98%), boric acid (H_3_BO_3_, 10043-35-3, 99.5%), hexamethylenetetramine (HMT, (CH_2_)_6_N_4_, 100-97-0, 99.5%), sulfur (S, 7704-34-9, 99.9%), potassium hydroxide solution (KOH, 1310-58-3, 45 wt % in H_2_O), potassium persulfate (K_2_S_2_O_8_, 7727-21-1, 99.0%), water-^18^O (H_2_^18^O, 14314-42−2, 97 atom % ^18^O). A platinum plate electrode holder (J110, Φ 6 × 80 mm) was used as the working electrode holder, a platinum plate electrode (Pt 252, 5 × 5 × 0.2 mm) was used as the counter electrode, and a Hg/HgO electrode (R0501, 1 M KOH) was used as the reference electrode. All electrodes were purchased from Tianjin Aida Co., Ltd. Carbon cloth was purchased from CeTech Co., Ltd. The porous membrane used for DEMS measurements was purchased from Hangzhou Cobetter Filtration Co., Ltd. (pore size: ≤ 20 nm; thickness: 40 μm).

### Samples preparation

Sulfide-treated CoOOH and traditional CoOOH samples, referred to as S-CoOOH and R-CoOOH, were prepared using the following methods^17^. For working electrode preparation, the sample was directly grown on carbon cloth (CC, 1 × 2 cm^2^), which was then clamped using the above-mentioned platinum plate electrode holder and used as the corresponding working electrode. S-CoOOH was synthesized through electro-oxidation of cobalt sulfide. Initially, the carbon cloth was subjected to a pretreatment process at 500 °C for 1 h in air. Following this, metallic Co was electrodeposited onto the pretreated CC in a two-electrode configuration, employing CC as the working electrode and Pt as the counter electrode. The electrodeposition was carried out in a solution containing 0.15 M CoSO_4_·7H_2_O and 0.6 M H_3_BO_3_, with a constant current density of -10 mA cm^−2^ applied for 1 h. The loaded electrode was then rinsed with deionized (DI) water and dried at 60 °C for 4 h. The subsequent step involved preparing cobalt sulfide. The Co-coated CC sample was heated with 0.1 g sulfur at 400 °C in an N_2_ atmosphere for 1 h. After cooling down to room temperature (300 K), it was washed and dried under the conditions previously described. Finally, the dried sample underwent electro-oxidation in a three-electrode configuration, with Pt as the counter electrode and Hg/HgO as the reference electrode. This was done under a constant current density of 10 mA cm^−2^ for approximately 10 h.

R-CoOOH was prepared through topotactic conversion of β-Co(OH)_2_^17^. Initially, a 30 mL aqueous solution containing 5 mmol Co(NO_3_)_2_·6H_2_O and 10 mmol HMT, was prepared and subjected to vigorous ultrasonic agitation. Subsequently, the mixture along with cleaned CC was transferred to a 50 mL Teflon-lined stainless steel autoclave. This system was then sealed and maintained at 120 °C for 6 h. Post-thermal treatment, the resultant sample was washed using DI water and dried at 60 °C for 4 h. Finally, electro-oxidation of the prepared sample was performed in a three-electrode configuration, using Pt as the counter electrode and Hg/HgO as the reference electrode. A potential of 1.58 V versus Hg/HgO, without *iR*-compensation, was applied for 10 h in 1 M KOH electrolyte.

### Photocatalytic measurement

The photocatalytic oxygen evolution activity was evaluated on an MC-SPB10 system (Merry Change Co., Ltd., China) under 300 W Xe lamp equipped with an AM 1.5 G filter. 10 mg S-CoOOH powder was suspended in 20 mL potassium phosphate buffer (K_2_S_2_O_8_, 0.1 M, pH = 7)^22^, followed by ultrasonication for 5 min. Before the photocatalytic reaction, the chamber was evacuated to create a vacuum. Subsequently, nitrogen gas was introduced at a flow rate of 10.0 mL/min and kept for 10 h to remove all dissolved oxygen gas in the solution. Upon ensuring the complete removal of oxygen traces, light was introduced. The suspension was continuously stirred at a rate of 800 rpm and kept at 5 °C throughout the experiment. The gas product was manually collected and subsequently analyzed by GCMS-QP2010 SE (SHIMADZU, Japan).

### Electrocatalytic OER measurement

The electrochemical measurements were performed using an electrochemical workstation (VPM3, Bio-logic Inc., France), equipped with a built-in electrochemical impedance spectroscopy (EIS) analyzer. A three-electrode setup was utilized, consisting of an as-prepared sample based on carbon cloth (1 × 2 cm^2^) as the working electrode, Pt as the counter electrode, and pre-calibrated Hg/HgO as the reference electrode. The catalyst loadings of S-CoOOH and R-CoOOH were measured to 0.87 and 0.64 mg cm^−2^, respectively, determined from the weight difference of the carbon cloth substrate before and after catalyst preparation. The calibration of the reference Hg/HgO electrode was performed in an H_2_-saturated 1 M KOH electrolyte, using Pt as both the working and counter electrodes. In this study, the potential of Hg/HgO reference electrode was determined to be 0.098 V vs. SHE (standard hydrogen electrode). All potentials were converted to the reversible hydrogen electrode (RHE) scale according to: $E\left({\rm{RHE}}\right)=E+0.098+0.059\times {\rm{pH}}$. The electrolyte was 1 M aqueous KOH with a pH of 13.92 ± 0.02, freshly prepared and purified to remove Fe before use. The linear scan voltammetry (LSV) was measured at a scan rate of 1 mV s^−1^. The EIS analysis was performed over a frequency range from 10 mHz to 100 kHz. The spectra were recorded either at open-circuit potential or at applied potentials of 1.35, 1.40, 1.45, and 1.50 V versus RHE. For experiments conducted in dark situations, the electrochemical measurements took place in a dark box. Conversely, for those carried out under light conditions, the electrochemical cell was connected to a thermostatic bath with continuous water flow to maintain a constant catalyst surface temperature. A solar simulator (NBeT Solar-500, 300 W) emulating AM 1.5 G (100 mW cm^−2^) was used as the light source. The light intensity impinging on the electrocatalyst was regulated by adjusting the output current and irradiation distance of the solar simulator and was monitored using a light power meter (Newport 91150). In addition, light filters with wavelengths of 575, 800, 1200, and 1310 nm were applied for wavelength-dependent photon response measurements.

### Electrochemical surface area (ECSA) measurement

The ECSA data of each catalyst were estimated by assessing the double-layer capacitance via cyclic voltammetry (CV) in a static electrolyte. Potential sweeps were typically conducted in the non-faradaic region, centered at the open-circuit potential (OCP) over 0.1 V window. Here, the chosen potential ranged from 0.60 to 0.70 V versus RHE. The scan rates were varied from 10 to 100 mV s^−1^ in increments of 10 mV s^−1^. Charging current values (${i}_{c}$) were then determined from these CVs at multiple scan rates. A linear relationship emerged when plotting the charging current (${i}_{{\rm{c}}}$) against the scan rates (ν), with a slope indicative of the electrochemical double-layer capacitance (${C}_{{\rm{DL}}}$). The ECSA was subsequently obtained by dividing the ${C}_{{\rm{DL}}}$ by the specific capacitance ${C}_{{\rm{S}}}$ $({\rm{ECSA}}={C}_{{\rm{DL}}}/{C}_{{\rm{S}}})$, with ${C}_{{\rm{S}}}$ commonly valued at 0.04 mF cm^−2^^31^.

### *Operando* X-ray absorption spectroscopy (XAS)

*Operando* Co *K*-edge XAS spectra under light illumination were acquired at the X-ray absorption fine structure for catalysis (XAFCA)^32^ beamline of Singapore Synchrotron Light Sources (SSLS) using the fluorescence mode. *Operando* Co *K*-edge XAS spectra in the dark were conducted at the wiggler XAS beamline (121D) in Hutch B in Mode 2 at the Australian Synchrotron (Melbourne, Australia) using a Si (111) double-crystal monochromator in the fluorescence mode. The storage ring operated at 0.7 GeV with an electron current of approximately 200 mA under top-up mode. Energy calibration was conducted using standard cobalt foil. Data acquisition occurred in total electron yield mode with a photon energy resolution of 450 meV. The photon energy calibration referenced the characteristic intensity dip, which was associated with the carbon contamination of the beamline optical components at 284.4 eV. All XAS spectra were normalized to the incident photon intensity (I_0_) monitored by the focusing mirror. The Athena software was utilized for energy calibration and background removal^33^. The *operando* measurements were facilitated by a homemade PEFF electrochemical cell with a 2 × 2 cm^2^ Kapton tape-sealed window (see Supplementary Figs. S21, S22). This three-electrode setup employed a sample based on carbon cloth as the working electrode, a carbon rod as the counter electrode, and Ag/AgCl as the reference electrode in 1 M KOH electrolyte. During operation, to minimize interference from generated bubbles and avoid structural impacts from voltage variation, the electrochemical experiment was conducted at a constant low voltage of 1.43 V vs. RHE, with adjustments made to maintain an initial current of approximately 2 mA.

### ^18^O isotope technology

This technology is used to ascertain whether lattice oxygen within the catalyst participates in generating oxygen gas, thereby further confirming the electron transfer pathway^15^. The experimental procedure is divided into two sections: the ^18^O labeling of S-CoOOH catalyst and the subsequent analysis of gas products via mass spectrometry. In the first ^18^O labeling step, the pre-catalyst cobalt sulfide^17^ based on carbon cloth was dispersed in 97% H_2_^18^O KOH electrolyte (1 M), and activated via chronoamperometry (CA) at a current density of 10 mA cm^−2^ for 10 h to ensure thorough labeling of S-Co^18^O^18^OH. After labeling, the resulting material was then rinsed with ^16^O DI water to remove residual H_2_^18^O, followed by drying for further use. In the following gas product analysis section, the labeled sample was transferred to a H_2_^16^O KOH gas-tight electrochemical cell, linked to both a gas carrier and a mass spectrometer. Before gas collection, N_2_ gas was circulated for 24 h to remove dissolved oxygen from the electrolyte and to ensure the gas-tight system was free of oxygen. The experiment was conducted at a constant current of 3 mA cm^−2^, designed based on the established correlation between electron flow and oxygen generation. The gas collection process spanned a total of 45 min, with 0–15 min in the dark and 15–40 min under AM 1.5 G light irradiation. During the test, N_2_ flowed at a rate of 3 mL/min to facilitate the rapid expulsion of oxygen gas during the reaction. The gas product during each interval was gradually captured in separate gas carriers, after which the stored gas was analyzed using gas chromatography-mass spectrometry (GC-MS). In this analysis, the signals for mass-to-charge ratio *m/z* = 32 corresponding to ^32^O_2_ (^16^O^16^O), *m/z* = 34 to ^34^O_2_ (^16^O^18^O), and *m/z* = 36 to ^36^O_2_ (^18^O^18^O) were first collected, based on which the average ratios of ^34^O_2_/^32^O_2_, ^36^O_2_/^32^O_2_, and ^32^O_2_/^28^N_2_ were subsequently calculated.

### *Operando* DEMS with isotope labeling

The ^18^O-labeled differential electrochemical mass spectrometry (DEMS) measurements were operated using an *operando* DEMS system that consists of two interconnected vacuum chambers, with a high vacuum chamber housing a mass spectrometer and an ambient chamber containing an electrochemical cell. A schematic illustration of the DEMS electrochemical cell is provided in Supplementary Fig. S41. In the electrochemical system, Hg/HgO and Pt function as the reference and counter electrodes, respectively, while S-CoOOH on carbon cloth substrate serves as the working electrode. The in-situ generated gaseous oxygen products were directly transferred through a porous membrane into the vacuum chamber for mass spectrometer analysis^28^. S-CoOOH samples were tested under both complete darkness (in a sealed dark box) and illuminated conditions (AM 1.5 G). The process involves two steps using H_2_^18^O and H_2_^16^O as the supporting electrolyte (1 M KOH). Initially, the catalyst was labeled with ^18^O by performing three LSV cycles in an ^18^O-labeled 1 M KOH electrolyte (1.10-1.65 V vs. RHE, 5 mV s^−1^) under either dark or illuminated conditions. Subsequently, the ^18^O-labeled catalysts were rinsed with ^16^O water to remove any residual H_2_^18^O. Finally, the above catalysts underwent three LSV cycles in a normal electrolyte 1 M KOH with H_2_^16^O as the solvent, under the same potential range and scan rate. Each sample was tested under the same illumination conditions as during labeling, that is, a sample labeled in the dark was tested in the dark, whereas a sample labeled under illumination was tested under illumination. Meanwhile, the gaseous oxygen products, including ^32^O_2_ (^16^O^16^O), ^34^O_2_ (^16^O^18^O), and ^36^O_2_ (^18^O^18^O), were monitored in real-time by the online mass spectrometer.

### Theoretical calculation

Density functional theory (DFT) calculations were conducted using the Vienna Ab initio Simulation Package (VASP), employing the Perdew-Burke-Ernzerhof (PBE) functional and projector-augmented wave (PAW) pseudopotentials^34^. DFT + U (U-J = 3.52 for Co)^35^ and DFT-D3 dispersion correction^36^ were considered in all calculations. Plane wave kinetic energy cutoff was set to 500 eV, and the energy and force convergence criteria were set to 10^−5 ^eV and 0.03 eV Å^−1^, respectively. The S-CoOOH supercell was constructed with a size of a = 14.861 Å, b = 23.115 Å, and c = 33.130 Å containing 108 Co, 108 H and 216 O atoms (Supplementary Figs. S34, 35). For R-CoOOH model construction, first, the standard CoOOH structure adopted from the experimental result underwent an optimization process. The optimized model was then used to calculate the density of states. The CONTCAR files of the optimized S-CoOOH and R-CoOOH models are provided in Supplementary Data 1.Due to the supercell approach, only the Gamma k-point was used for DFT simulations. In this case, we changed the total number of electrons in the system by setting NELECT in INCAR. We add one more electron into the supercell model for 7-electron O. The climbing image nudged elastic band (CI-NEB) method^37^ was employed to calculate the energy barrier and find the transition state. A vibrational frequency calculation was carried out at this saddle point, and the presence of a single imaginary frequency confirms that the structure is indeed a first-order saddle point (Supplementary Table S5). The thermodynamic calculations were performed using the optPBE-vdW functional with the van der Waals (vdW) correction. The solvent effect was considered using the implicit solvation model implemented in the VASP model. The free energies were referenced to U = 0 V and 1.23 V vs. RHE, where the pH effect is inherently included in the reference potential. More detailed computational parameters are provided in Supplementary Information.

### Resonant inelastic X-ray scattering (RIXS)

Co 2p3d resonant inelastic X-ray scattering (RIXS) spectra were recorded at i21 Diamond Light Source in the UK^38^. Samples were transferred to the spectrometer using a vacuum-transfer suitcase to prevent air exposure. It was followed by evacuation to ultra-high vacuum (UHV) and overnight degassing. RIXS maps were acquired in 0.25 eV energy increments over the 776–788 eV range. RIXS line scans were recorded at the resonance energy for molecular O_2_ (530.5 eV). All data were collected in a partial fluorescence mode for bulk sensitivity. All measurements were conducted at 20 K to minimize any possible beam damage.

### UV-Vis absorption and reflectance measurements

UV-Vis absorption and diffuse reflectance spectra of S-CoOOH powder were collected on a spectrophotometer (LAMBDA 950, PerkinElmer, USA) equipped with an integrating sphere. The measurements were performed in the wavelength range of 200–2400 nm with a step size of 5 nm, using BaSO_4_ as the reflectance reference. A deuterium lamp was used for the 200–312 nm region, while a tungsten lamp was employed for the 312–2400 nm region.

## Supplementary information

Supplementary Information

Description of Additional Supplementary Files

Supplementary Data 1

Transparent Peer Review file

## Source data

Source Data

## Data Availability

All the data supporting the findings of this study are included within the paper and its supporting files and are available from the corresponding authors on request. Source data are provided with this paper.
