# Supplementary material for: Light-triggered oxygen redox activity at the edge of cobalt oxyhydroxide for superior water oxidation
Source: Nat Commun. 2026 Jun 11;17:7417. doi: 10.1038/s41467-026-74386-1 (PMC13408805; doi:10.1038/s41467-026-74386-1)
Supplement: Supplementary file 1 — Supplementary Information [file 41467_2026_74386_MOESM1_ESM.pdf]

Supplementary Information

Light Triggered Oxygen Redox Activity at the Edge of Cobalt Oxyhydroxide for Superior Water Oxidation

Xin Zhang[1], Qiyun Wang[2], Qi Zhang[1], Haoyin Zhong[1], Chao Wu[2,3], Baorui Jia[1,4], Junchen Yu[1], Ke-Jin Zhou[5], Yuanjie Li[6], Yong-Wei Zhang[1,7], Zhi Gen Yu[1,7], Shibo Xi[3*], Xiaopeng Wang[2,8*], Junmin Xue[1*]

[1]Department of Materials Science and Engineering, National University of Singapore, Singapore 117575, Singapore.

[2]College of Materials Science and Engineering, Sichuan University, Chengdu 610065, China.

[3]Institute of Sustainability of Chemical, Energy and Environment (ISCE), Agency for Science, Technology and Research (A*STAR), Singapore 627833, Singapore.

[4]Institute for Advanced Materials and Technology, University of Science and Technology Beijing, Beijing 10083, China.

[5]Diamond Light Source, Harwell OX110QX, UK.

[6]Dongfang Electric (Fujian) Innovation Institute Co., Ltd., Fujian 350108, China.

[7]Institute of High Performance Computing (IHPC), Agency for Science, Technology and Research (A*STAR), Singapore 138632, Republic of Singapore.

[8]State Key Laboratory of Intelligence Construction and Healthy Operation and Maintenance of Deep Underground Engineering, Sichuan University, Chengdu 610065, China.

*Corresponding to msexuejm@nus.edu.sg (Junmin Xue); wangxiaopeng0620@163.com (Xiaopeng Wang); Xi_shibo@isce2.a-star.edu.sg (Shibo Xi)

Contents

Structural characterization of S-CoOOH 3

Pro-long repeatability of light-induced OER behavior 5

Electrochemical performance in dark vs. under light 6

Photon response under varying light intensities and wavelengths 9

Possible reasons for enhanced OER activity triggered by light 12

Operando Co *K*-edge XAS experiments 25

TMA^+ probe experiments in the dark 33

Operando DEMS with ^{18}O isotope measurements 34

Optimized model for simulations 39

PDOS results for Co 3*d* and O 2*p* orbitals 45

Photophysical property of S-CoOOH 46

Schematic of DEMS electrochemical cell 47

Elemental analysis of S-CoOOH 48

Summary of OER performance of cobalt-based catalysts 49

Fitted atomic coordination number 52

Calculated entropy and enthalpy values 53

Vibrational frequency analysis of S-CoOOH mode 54

Supplementary references 55

Structural characterization of S-CoOOH

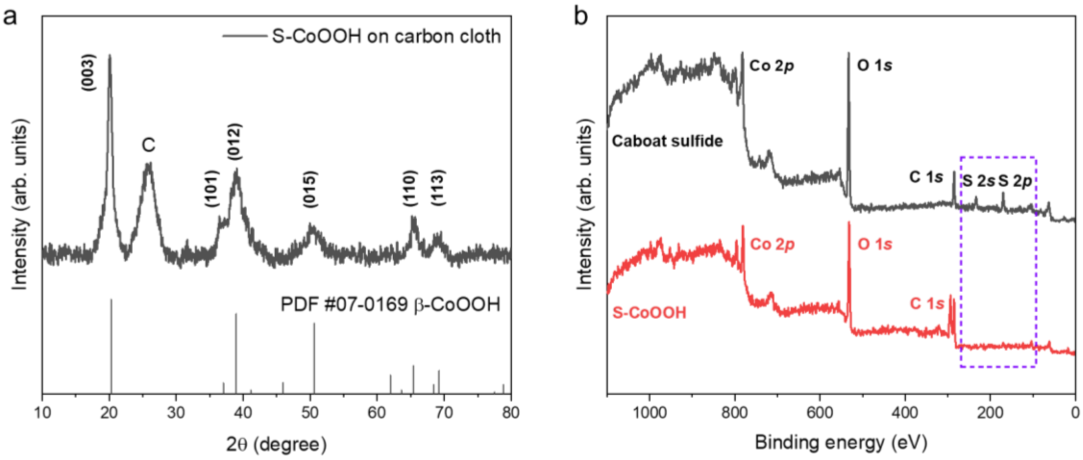

Supplementary Figure S1. Structural characterization of cobalt sulfide pre-catalyst before and after electrochemical oxidation. a XRD pattern of S-CoOOH based on carbon cloth, with standard β-CoOOH listed (PDF #07-0169). **b** XPS spectra of cobalt sulfide before (top) and after activation (bottom) in a wide range. Source data for Supplementary Fig.S1 are provided as a Source Data file.

In this section, after long-term chronopotentiometry (CP) treatment, cobalt sulfide is verified to convert into CoOOH with negligible residual sulfur, as confirmed by X-ray diffraction (XRD), X-ray photoelectron spectroscopy (XPS), X-ray absorption spectroscopy (XAS), and inductively coupled plasma (ICP) analysis (Supplementary Figs.S1-S2, Table S1). As shown in Supplementary Fig.S1a, after a long-time electro-oxidation process (10-hour CP), XRD result reveals that cobalt sulfide pre-catalyst is fully reconstructed into rhombohedral β-CoOOH (R-3m space group, JCPDS 07-0169). The diffraction peaks at 20.24°, 36.98°, 38.89°, 50.58°, 65.34°, and 69.17° can be indexed to (003), (101), (012), (015), (110), and (113) crystal planes of β-CoOOH, respectively. The diffraction peak at 25.83° can be attributed to (002) crystal plane of carbon cloth substrate. No additional reflections associated with other crystalline phases are detected after electro-oxidation, suggesting a complete structural reconstruction. Besides, Supplementary Fig.S1b presents the wide-range XPS spectra of cobalt sulfide before and after activation. The peaks at binding energies of 160, 221, 279, 527, and 780 eV correspond to S 2*p*, S 2*s*, C 1*s*, O 1*s*, and Co 2*p*, respectively[1]. After long-time activation, the S 2*p* and S 2*s* signals disappear entirely in S-CoOOH.

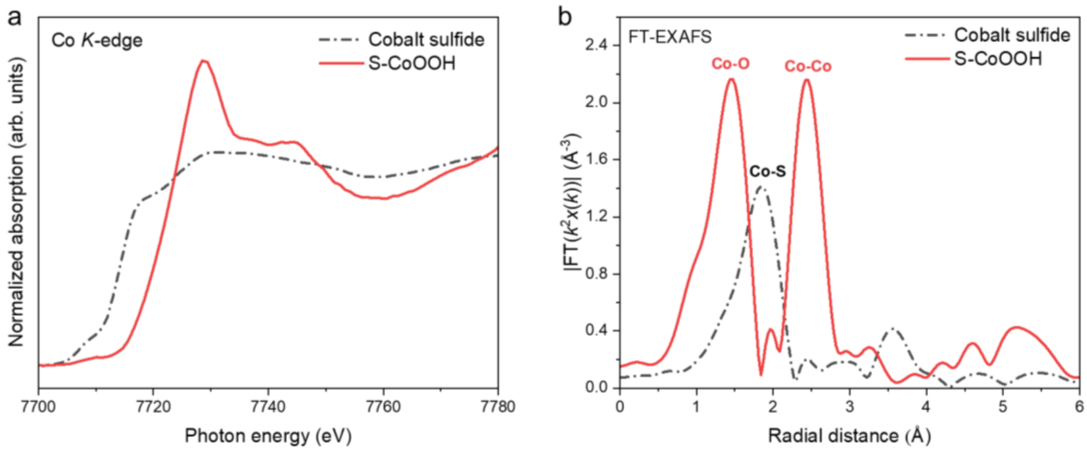

Supplementary Figure S2. Co *K*-edge XAS characterization of cobalt sulfide pre-catalyst before and after electrochemical oxidation. a Normalized Co *K*-edge XAS spectra of cobalt sulfide and S-CoOOH. **b** FT-EXAFS spectra of Co *K*-edge of cobalt sulfide and S-CoOOH. Source data for Supplementary Fig.S2 are provided as a Source Data file.

In addition, the structural characterization of cobalt sulfide before and after electro-oxidation is studied using XAS at Co *K*-edge. As shown in Supplementary Fig.S2a, cobalt sulfide undergoes electro-oxidation to S-CoOOH, exhibiting a red-shifted absorption edge and higher white-line intensity, which reflects a significant phase transformation. Supplementary Fig.S2b presents the corresponding Fourier-transformed extended X-ray absorption fine structure (FT-EXAFS) spectra. The peaks associated with Co-S bonds in cobalt sulfide disappear fully in S-CoOOH, indicating that all Co-S bonds are broken after electro-oxidation.

Pro-long repeatability of light-induced OER behavior

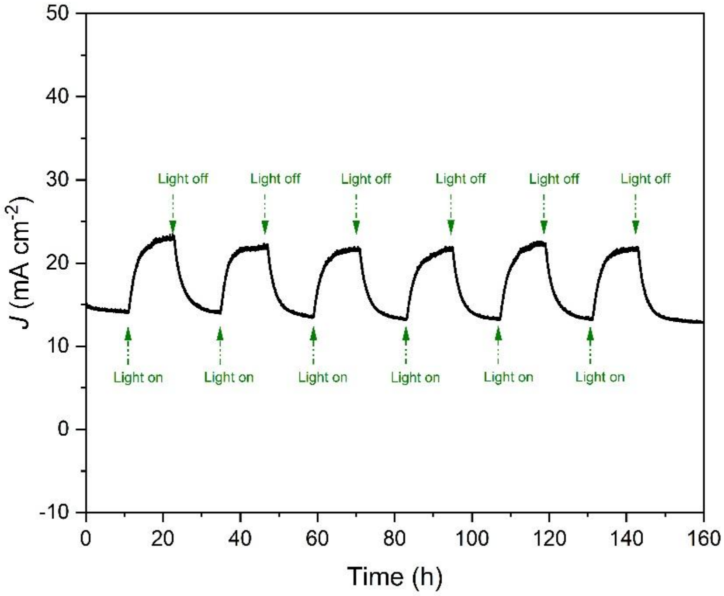

Supplementary Figure S3. The repeatability experiments for electrochemical measurements of S-CoOOH. Chronoamperometry (CA) curve for S-CoOOH at a constant potential of 1.45 V versus a reversible hydrogen electrode (RHE), with multiple cycles of turning light on and off. Source data for Supplementary Fig.S3 are provided as a Source Data file.

Supplementary Fig.S3 indicates the long-term repeatability of S-CoOOH over six light turn-on/off cycles, maintaining stability over 160 hours. The current after 160 hours of illumination cycling remains nearly identical to the initial value, indicating excellent operational stability under light irradiation. This clearly indicates that the light-enhanced OER activity is reversible behavior, rather than an irreversible process caused by reconstruction. As such, the possibility of reconstruction could be excluded.

Electrochemical performance in dark vs. under light

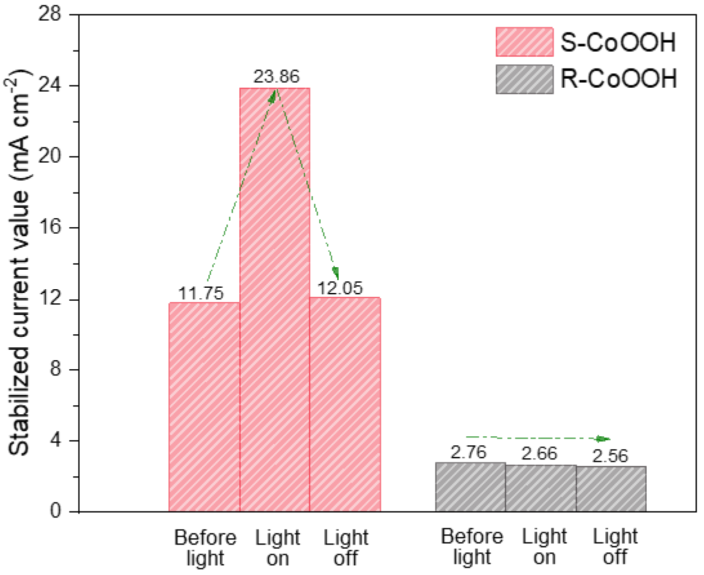

Supplementary Figure S4. Effect of light irradiation on the current response of S-CoOOH and R-CoOOH. Stabilized current values at 1.55 V and 1.45 V for R-CoOOH and S-CoOOH across three conditions: pre-illumination (Before light), during illumination (Light on), and post-illumination (Light off), with data obtained from **Fig.1a.** Source data for Supplementary Fig.S4 are provided as a Source Data file.

To illustrate the impact of light illumination on oxygen evolution reaction (OER) performance, stabilized current values across three states are depicted in Supplementary Fig.S4. The transitions from dark to light and back to dark during the OER are sequentially recorded as Before light, Light on, and Light off. These results reveal that for S-CoOOH, the current density increases to approximately twofold upon light irradiation and gradually returns to its initial value when the light is turned off. In contrast, R-CoOOH exhibits no appreciable change in current density under illumination, indicating no light response. This observation further verifies the reversible enhancement in OER performance of S-CoOOH under light illumination.

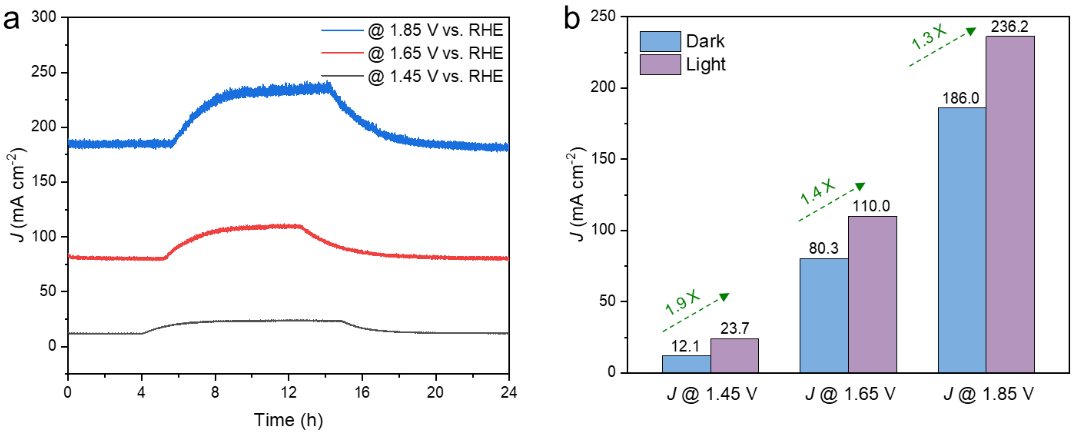

Supplementary Figure S5. Electrochemical measurements of S-CoOOH across a range of potentials. a Chronoamperometry curves for S-CoOOH at constant potentials of 1.45 V, 1.65 V, and 1.85 V versus a reversible hydrogen electrode (RHE) in 1 M KOH electrolyte. **b** Comparison of the current density of S-CoOOH in dark and under light at low (1.45 V), middle (1.65 V), and high potentials (1.85 V). Source data for Fig.S5 are provided as a Data file.

As evidenced by the linear sweep voltammetry (LSV) curves in Fig.1b, S-CoOOH consistently maintains its light-enhanced OER performance across a range of potentials. To further assess the light response of S-CoOOH at various potentials, potentials of 1.45 V, 1.65 V, and 1.85 V versus a reversible hydrogen electrode (RHE) were applied, yielding low, medium, and high current densities, respectively. Consistent light intensity, calibrated at 100 mW cm^{-2} by a light power meter, was irradiated from a light source to simulate AM 1.5 G condition. Constant voltages were applied at these three potentials, and the current density response to light was monitored (Supplementary Fig.S5a), corroborating the trends observed in the LSV curves (Fig.1b).

Supplementary Fig.S5a illustrates that at 1.45 V, the current density increases from 11.78 mA cm^{-2} to 23.74 mA cm^{-2} over approximately 7 hours, representing an increase of about 101.44 %. At 1.65 V, the current density rises from 79.93 mA cm^{-2} to 109.08 mA cm^{-2} within the same duration, an increase of approximately 36.47 %. Similarly, at 1.85 V, the current density climbs from 185.32 mA cm^{-2} to 236.27 mA cm^{-2} over 7 hours, making an increase of about 27.49 %. Supplementary Fig.S5b then presents the stabilized current density derived from Supplementary Fig.S5a under both dark and light conditions, highlighting a distinct improvement in current density at all three voltages upon exposure to light.

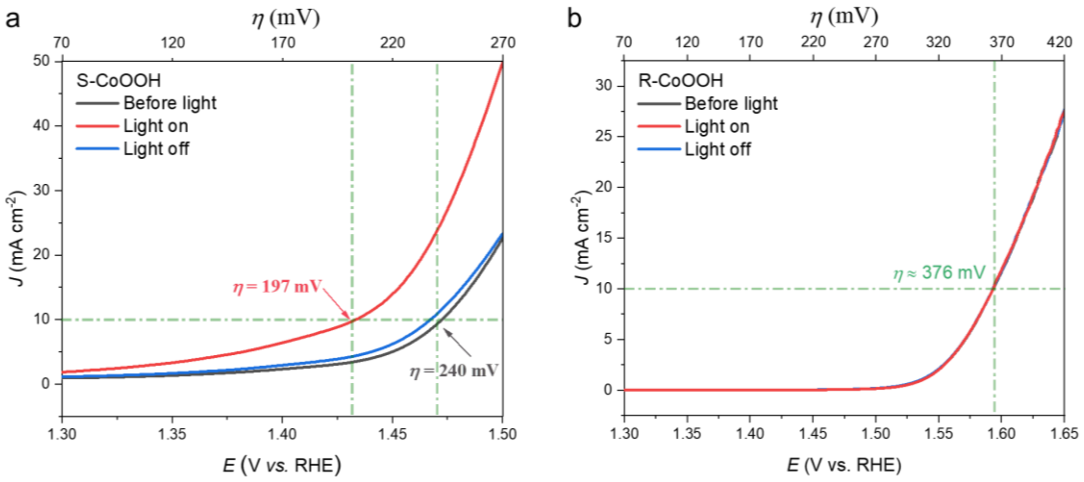

Supplementary Figure S6. Raw OER polarization curves of S-CoOOH and R-CoOOH before, during, and after light irradiation. a, b OER polarization curves of S-CoOOH (a) and R-CoOOH (b) without *iR* correction, across three conditions: pre-illumination (Before light), during illumination (Light on), and post-illumination (Light off) (scan rate = 1 mV s^{-1}, pH = 13.92 ± 0.02, and electrode area = 1×2 cm^{-2}). The experimental was carried out at room temperature (300 K) in 1 M KOH electrolyte, using Pt as the counter electrode and Hg/HgO as the reference electrode. The horizontal dashed line makes a current density of 10 mA cm^{-2} for evaluating the overpotential (η). Source data for Supplementary Fig.S6 are provided as a Source Data file.

The raw LSV curves without *iR* correction are given in Supplementary Fig.S6. This demonstrates that both the pre- and post-light illumination conditions (labeled as Before light and Light off) display identical overpotentials of 240 mV at a current density of 10 mA cm^{-2} in S-CoOOH, demonstrating the reversibility of light-enhanced OER performance.

Photon response under varying light intensities and wavelengths

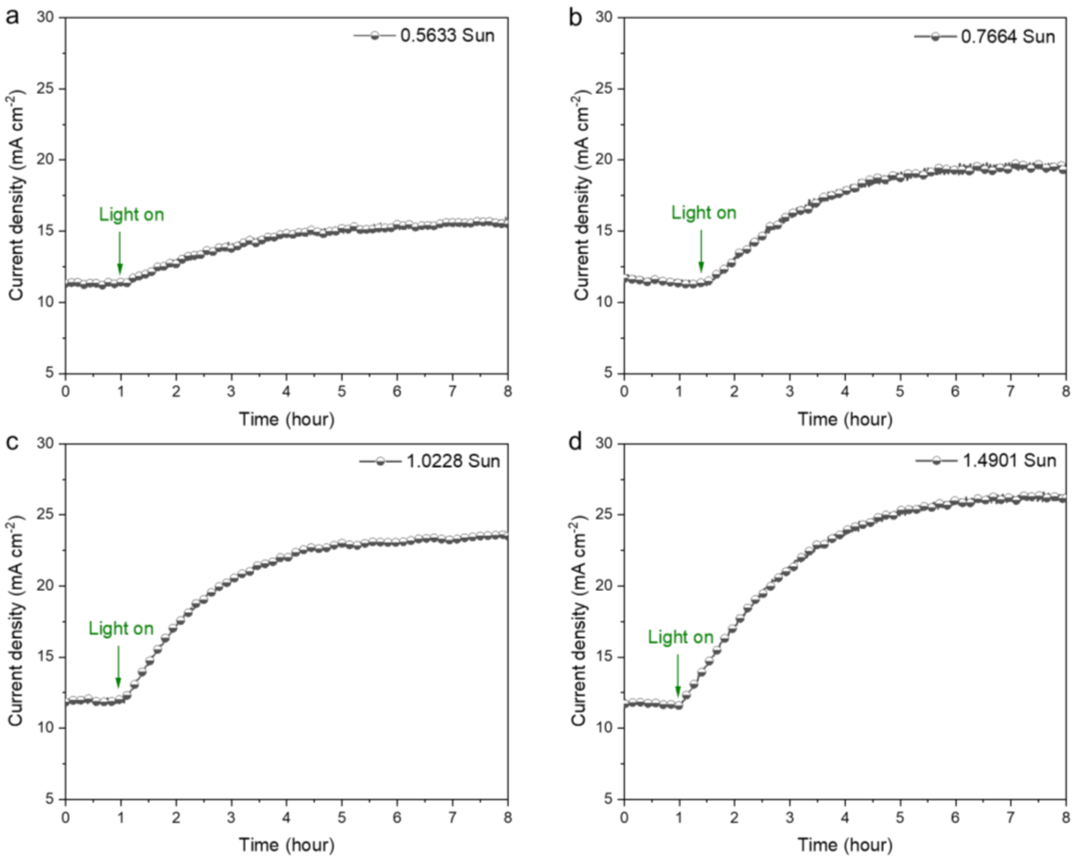

Supplementary Figure S7. Photon response measurements under different light intensities: **a** 0.5633 Sun (56.33 mW cm^{-2}), **b** 0.7664 Sun (76.64 mW cm^{-2}), **c** 1.0228 Sun (102.28 mW cm^{-2}), and **d** 1.4901 Sun (149.01 mW cm^{-2}). Source data for Supplementary Fig.S7 are provided as a Source Data file.

Supplementary Fig.S7 presents the photon response measurements under varying illumination intensities, revealing that the light-induced behavior is closely related to light intensity. As the light intensity increases from 0.5633 to 0.7664, 1.0228, and 1.4901 Sun, the increase in current density becomes progressively more pronounced.

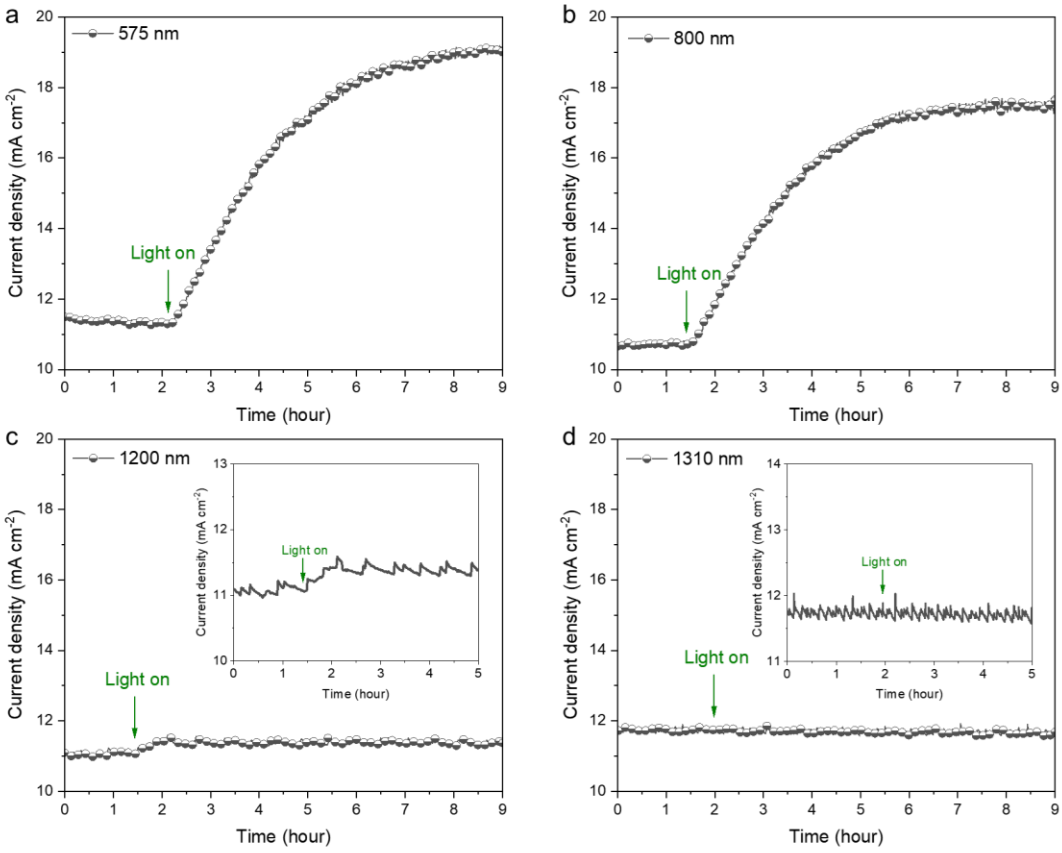

Supplementary Figure S8. Photon response measurements equipped with different wavelength filters of 575 nm (**a**), 800 nm (**b**), 1200 nm (**c**), and 1310 nm (**d**), respectively. Source data for Supplementary Fig.S8 are provided as a Source Data file.

Supplementary Fig.S8 provides the photon response measurements under different illumination wavelengths. It is shown that with a wavelength of 575 nm, the current increases significantly when light is switched on (Supplementary Fig.8a). As the wavelength increases to 800 nm, a noticeable current enhancement can still be observed (Supplementary Fig.8b). The PDOS of cobalt 3*d* and oxygen 2*p* orbitals reveals an energy barrier of 0.98 eV between (M-O) to Co 3*d* orbital in S-CoOOH, corresponding to a light wavelength of about 1260 nm (Supplementary Fig.S39). When a light source of 1200 nm is used, the current increment is barely detectable (Supplementary Fig.S8c), indicating that the wavelength of 1200 nm could trigger ligand to metal charge transfer (LMCT) minimally, which aligns with our calculations. Interestingly, switching the light source to a higher wavelength of 1310 nm does not trigger any observable change in the current (Supplementary Fig.8d), which suggests the inability of this

wavelength to trigger LMCT due to the kinetic energy barrier. Thus, based on these results, it can be concluded that the lowest wavelength that can trigger LMCT in S-CoOOH is about 1200 nm (1.03 eV), which is in close agreement with the calculated value (0.98 eV, 1260 nm).

In our work, the observed photon response of different light wavelengths is not related to a conventional band gap excitation, but is instead associated with light-induced electron transfer from occupied (M-O) bond to unoccupied Co 3*d* orbitals (Fig.5e,f). As discussed above, these photon response measurements show good agreement with the calculated energy barrier between (M-O) to Co 3*d* orbital (Supplementary Fig.S8). These findings strongly validate that photon response measurements under varying illumination wavelengths in our work are closely associated with electron transfer from (M-O) bond to Co 3*d* orbitals.

Possible reasons for enhanced OER activity triggered by light

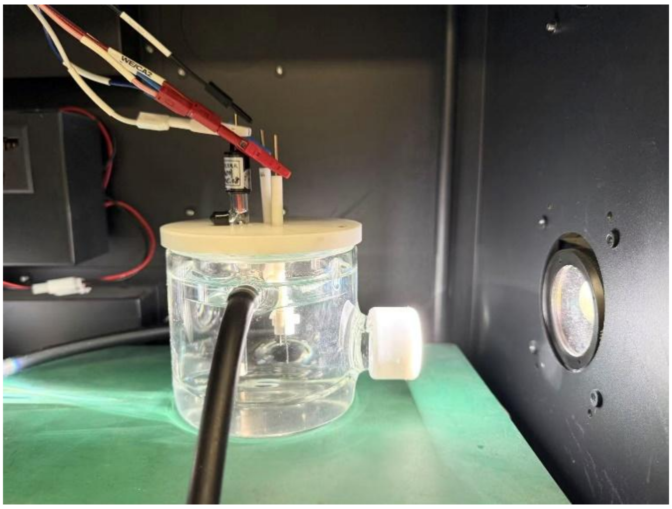

Supplementary Figure S9. Photograph of the actual thermostatic bath connected to an electrochemical cell.

Several potential causes for the distinctive increase in OER performance under light illumination are explored. Here, the thermal effect on the catalytic activities is ruled out. Moreover, electrochemical surface area (ECSA) findings and electrochemical impedance spectroscopy (EIS) dismiss grain size changes and reductions in the catalyst's ohmic resistance due to photon influx under illumination[2]. Additionally, the photocatalytic oxygen evolution experiment was performed to assess the potential of S-CoOOH to generate light-induced holes. The detailed discussion is as follows.

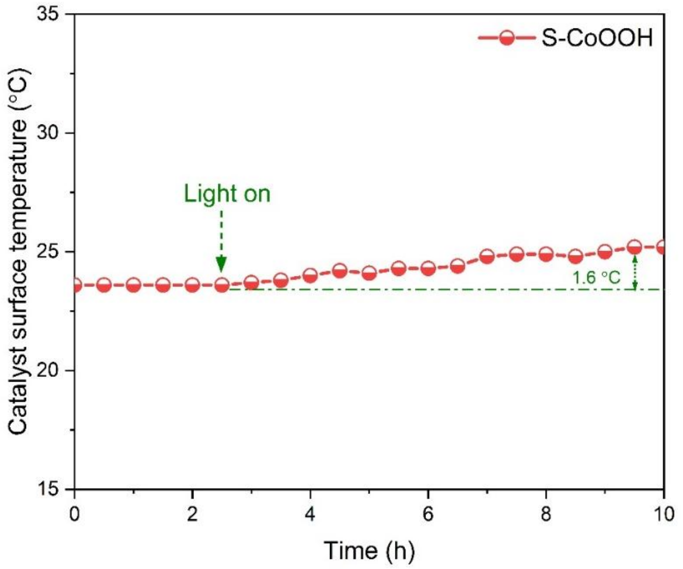

Supplementary Figure S10. Catalyst surface temperature recorded under light illumination during chronoamperometry (CA) process, corresponding to **Fig.1a**. Source data for Supplementary Fig.S10 are provided as a Source Data file.

To avoid the thermal effect on the catalytic activities, the electrochemical cell connected to a thermostatic bath was used for electrochemical testing under light illumination, whereby cooling water was used to keep the temperature of the system constant (Supplementary Fig.S9). The surface temperature of catalysts was monitored by a thermocouple and was found to increase by less than 2 ºC throughout the whole measurement process (Supplementary Fig.S10).

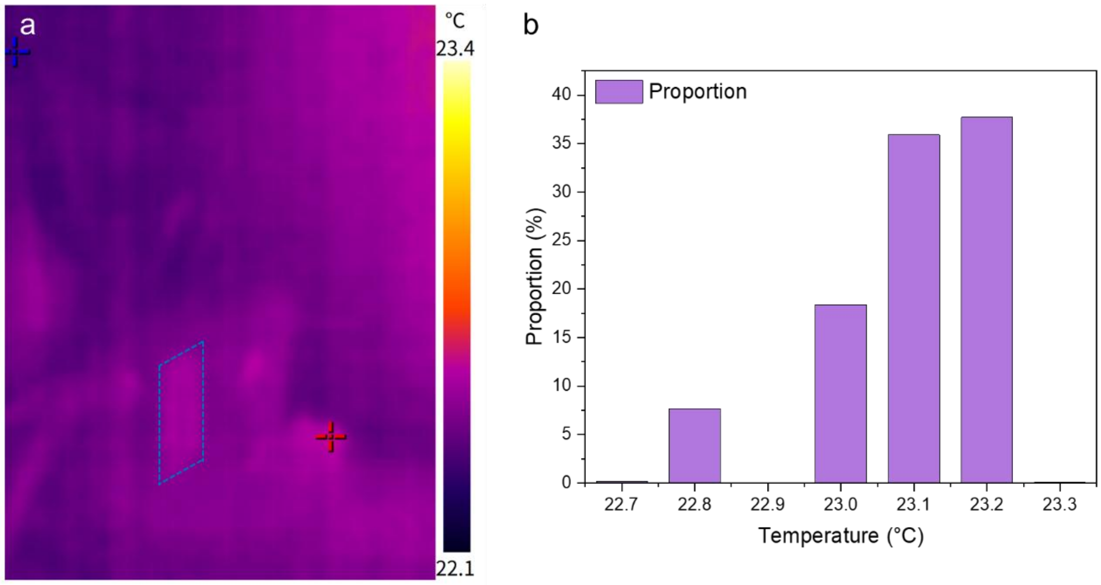

Supplementary Figure S11. Photothermal IR characterization of S-CoOOH during OER under light. a Photothermal IR mapping of S-CoOOH during OER under light, with the region enclosed by a blue box corresponding to S-CoOOH sample based on carbon cloth substrate. **b** Temperature histogram of S-CoOOH based on carbon cloth, highlighted by a blue box in Supplementary **Figure S11a**. Source data for Supplementary Fig.S11b are provided as a Source Data file.

Supplementary Figure S11a presents photothermal IR mapping of S-CoOOH during OER under light, in which the sample region is marked by a blue box. The corresponding temperature histogram of this region (Supplementary Figure S11b) reveals a spatially uniform temperature distribution across the entire catalyst layer (22.7-23.3 °C), with no discernible localized “hot spots” or localized heating. Current characterization techniques make it challenging to direct probe nanoscale "hot spots"; however, the photothermal IR mapping shows no detectable microscale thermal inhomogeneity, which partially suggests that illumination does not induce significant localized heating. The existence of nanoscale hot spots remains an ongoing question and warrants further investigation in the future.

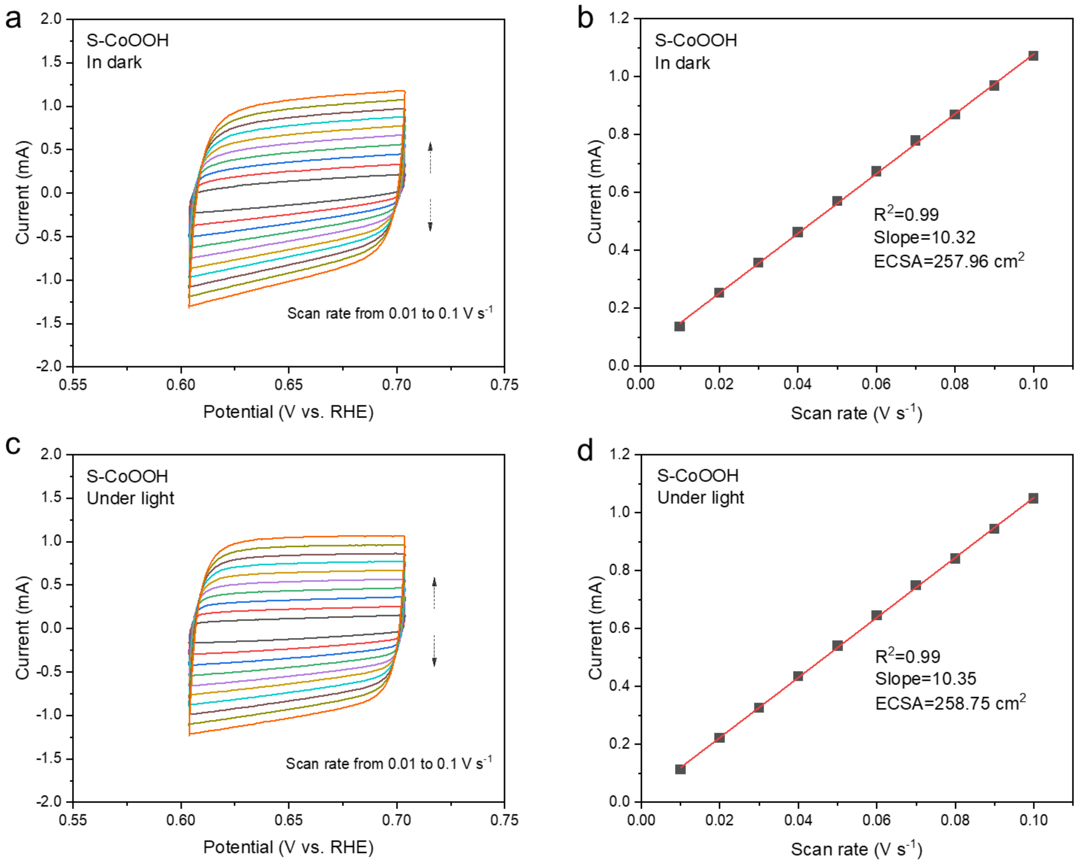

Supplementary Figure S12. The ECSA of S-CoOOH with and without light. a, c cyclic voltammogram (CV) curves of S-CoOOH without or with light, in 1 M KOH at different scan rates from 10 to 100 mV s^{-1}, charging current densities at 0.65 V versus RHE plotted against the scan rate. **b, d** The current density improvement (Δj) versus scan rate plot for the estimation of ECSA, where R^2 (R-squared) is the coefficient of determination for fitting. Source data for Supplementary Fig.S12 are provided as a Source Data file.

The intrinsic activities of S-CoOOH and R-CoOOH in response to light irradiation are presented, with current density normalized to ECSA (Supplementary Figs.S12-14). As shown in Supplementary Fig.S12, the ECSA for S-CoOOH is 257.96 cm^2 in dark and 258.75 cm^2 under light, demonstrating a negligible change. These findings indicate that there is no grain growth under illumination, with no significant changes in grain size. Combined with the reversibility of the light-induced OER enhancement, it rules out grain size variation as a contributing factor to the observed improvement[2].

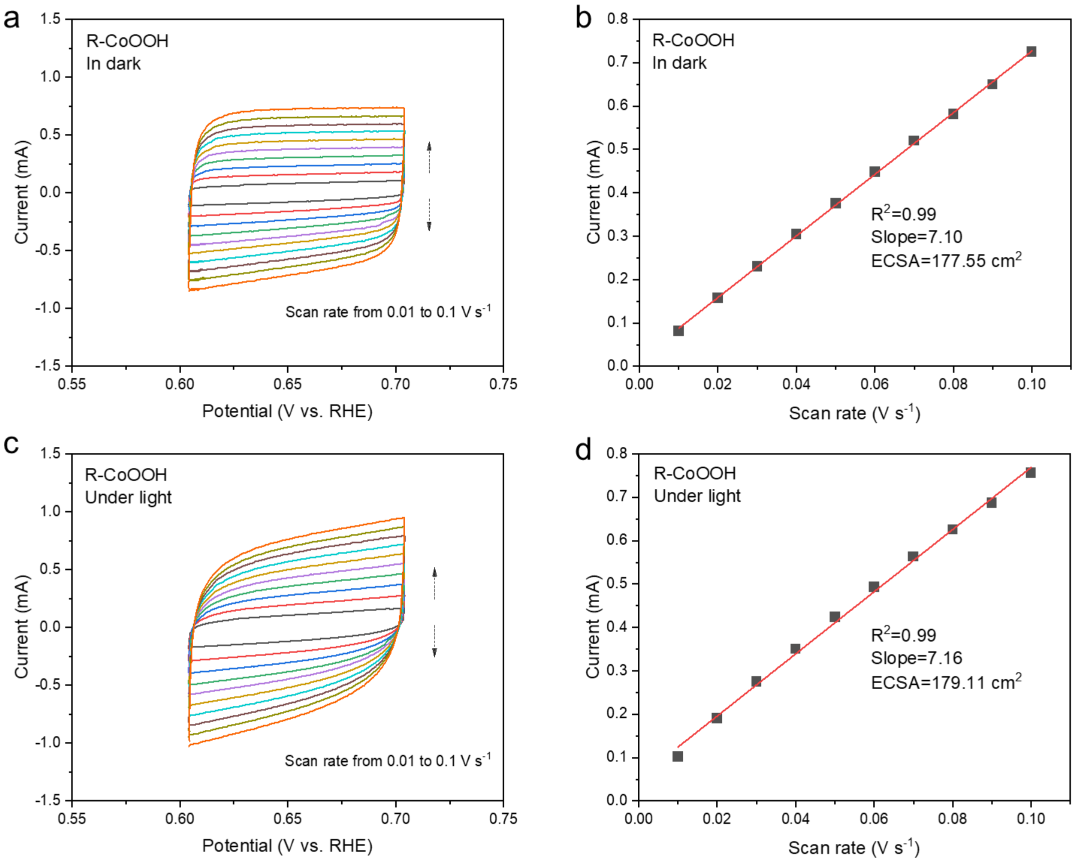

Supplementary Figure S13. The ECSA of R-CoOOH with and without light. a, c CV curves of R-CoOOH without or with light, in 1 M KOH at different scan rates from 10 to 100 mV s^{-1}, charging current densities at 0.65 V versus RHE plotted against the scan rate. **b, d** The current density improvement (Δj) versus scan rate plot for the estimation of ECSA, where R^2 (R-squared) is the coefficient of determination for fitting. Source data for Supplementary Fig.S13 are provided as a Source Data file.

Similarly, the ECSA for R-CoOOH is 177.55 cm^2 in dark and 179.11 cm^2 under light, showing a negligible change, as detailed in Supplementary Fig.S13.

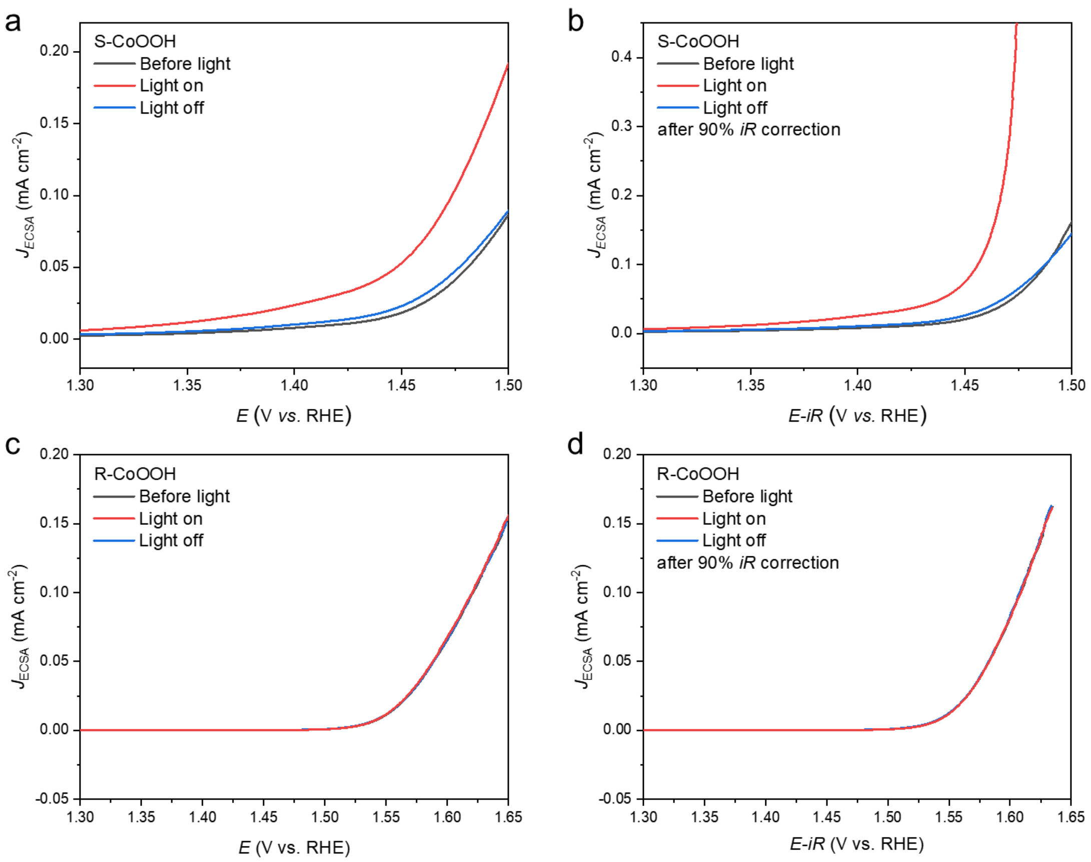

Supplementary Figure S14. OER polarization curves of S-CoOOH and R-CoOOH normalized to ECSA across three conditions: pre-illumination (Before light), during illumination (Light on), and post-illumination (Light off). a, b LSV curves of S-CoOOH without and with *iR*-correction, respectively. **c, d** LSV curves of R-CoOOH without and with *iR*-correction, respectively. The experiments were carried out at room temperature (300 K) in 1 M KOH electrolyte, using Pt as the counter electrode and Hg/HgO as the reference electrode. Source data for Supplementary Fig.S14 are provided as a Source Data file.

As presented in Supplementary Fig.S14, the ECSA normalized current density difference between light and dark conditions for S-CoOOH at an overpotential of 270 mV is 0.263 mA cm^{-2}. In contrast, the normalized current densities for R-CoOOH under both dark and light conditions are nearly identical.

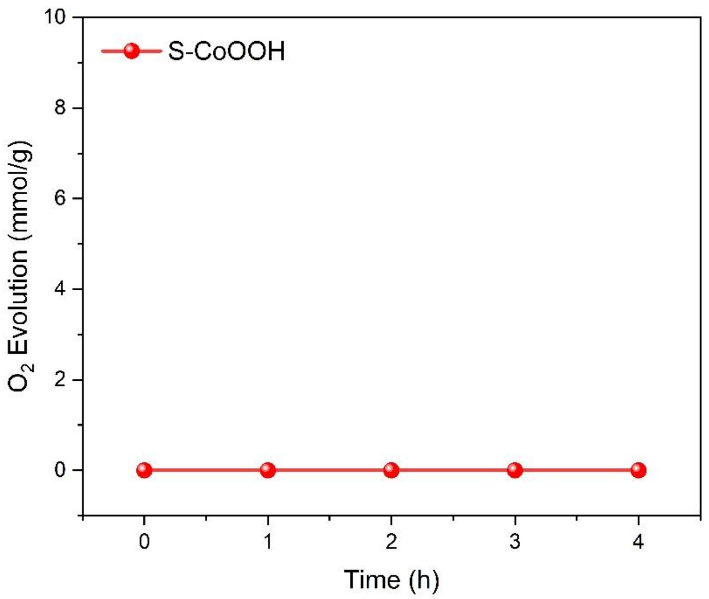

Supplementary Figure S15. Photocatalytic oxygen generation as a function of irradiation time. The gas chromatograph measurement of S-CoOOH, using $K_2S_2O_8$ as electron acceptor under AM 1.5G condition. Source data for Supplementary Fig.S15 are provided as a Source Data file.

Next, standard photocatalytic oxygen evolution measurements were conducted using sacrificial electron acceptors to evaluate whether the band position of S-CoOOH is suitable for functioning as a photocatalyst for oxygen evolution[2]. The potential of holes was assessed using gas chromatography with $K_2S_2O_8$ serving as the electron acceptor, and no oxygen was detected over a 4-hour test period (Supplementary Fig.S15). This outcome confirms that the observed improvement in OER performance of S-CoOOH is not due to the generation of light-induced holes.

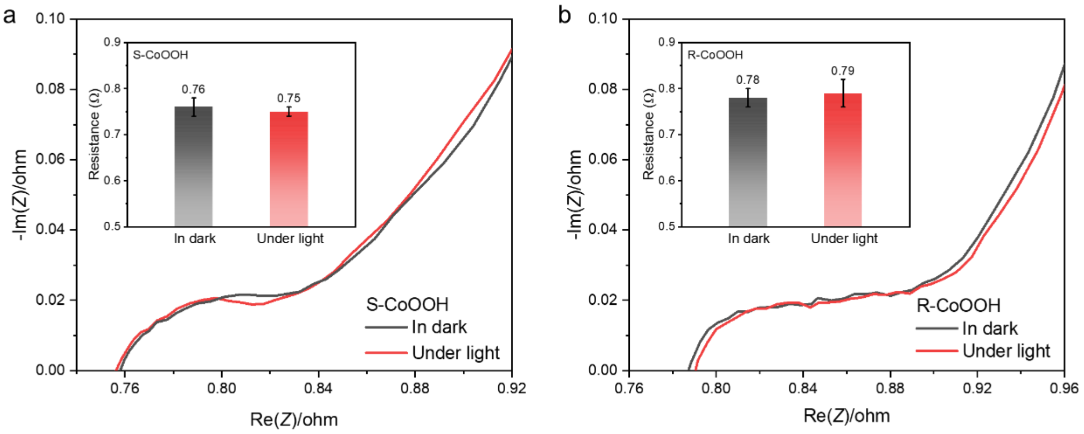

Supplementary Figure S16. Electrochemical impedance spectroscopy (EIS) data measured at open-circuit potential for S-CoOOH (**a**) and R-CoOOH (**b**) with and without light, where -Im(*Z*) and Re(*Z*) correspond to the imaginary and real parts of the impedance. Error bars in Supplementary Fig.S16a,b represent the standard deviation. Source data for Supplementary Fig.S16 are provided as a Source Data file.

The EIS data measured at open-circuit potential for both S-CoOOH and R-CoOOH in dark and under light are provided in Supplementary Fig.S16. It is found that the resistances for each sample in dark and under light are relatively similar, ruling out the possibility that photon influx reducing catalyst resistance contributes to the observed light-enhanced OER performance.

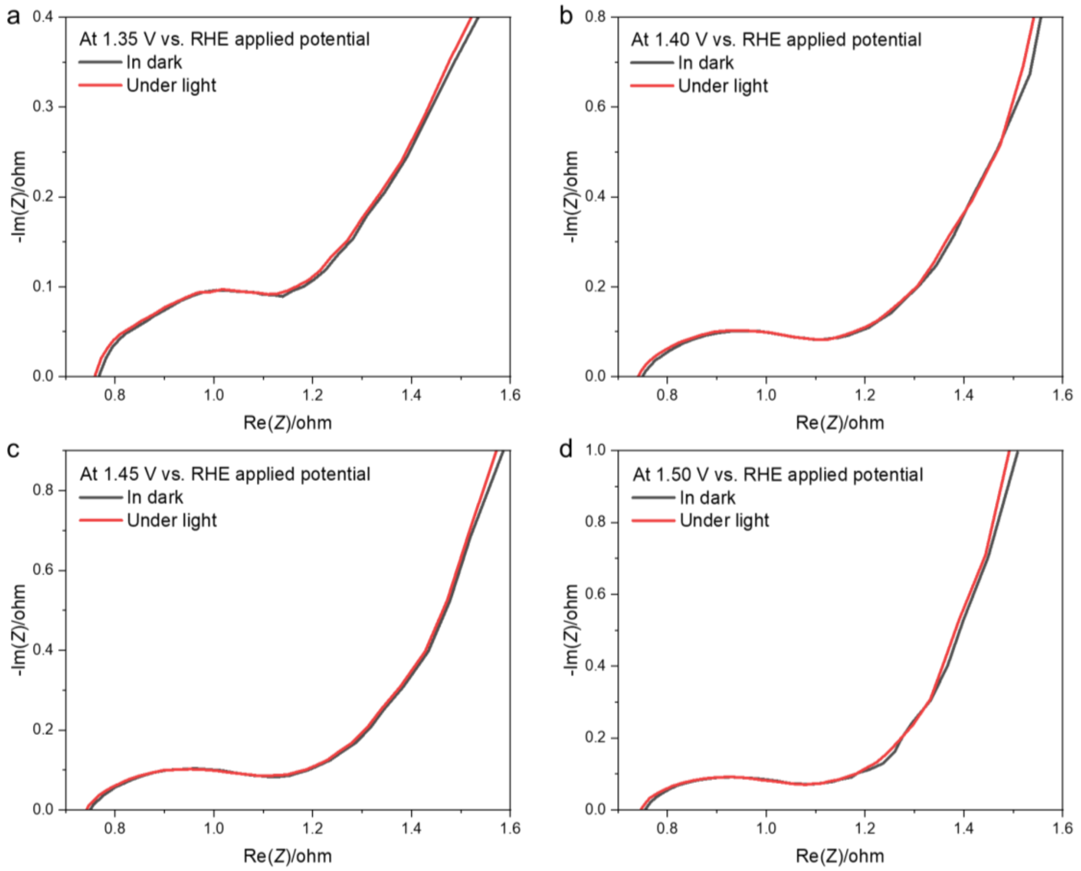

Supplementary Figure S17. Potentiostatic electrochemical impedance spectroscopy (PEIS) data for S-CoOOH in dark and under light, recorded at different applied potentials of 1.35 V (**a**), 1.40 V (**b**), 1.45 V (**c**), and 1.50 V (**d**) vs. RHE. Source data for Supplementary Fig.S17 are provided as a Source Data file.

As shown in Supplementary Figure S17, the potentiostatic electrochemical impedance spectroscopy (PEIS) results collected at different applied voltages (1.35 V, 1.40 V, 1.45 V, and 1.50 V vs. RHE) show no noticeable changes between dark and light conditions, indicating that the conductivity of catalyst is not improved under light. This rules out the possibility of photocurrent-induced conductivity improvement.

The slight difference in PEIS values between dark and illuminated conditions can be attributed to the fact that the effect of light on the adsorption configurations involves only a small fraction of the overall Co-O unit cells, due to the limited fraction of high-spin Co^{3+} species in S-CoOOH. Moreover, the effect of light on the adsorption configurations is limited to a specific step and is reversible, rather than affecting the whole OER process. Therefore, light

induces minimal alteration of the overall catalyst-electrolyte interface, resulting in similar resistance values under dark and illuminated conditions.

In addition, the above photocatalytic test (Supplementary Figure S15), which does not involve oxygen evolution, and light on/off current response curve (Supplementary Figure S3), which exhibits a gradual increase rather than an abrupt step-like increase, both further confirm that the observed current enhancement does not originate from a conventional photocurrent effect.

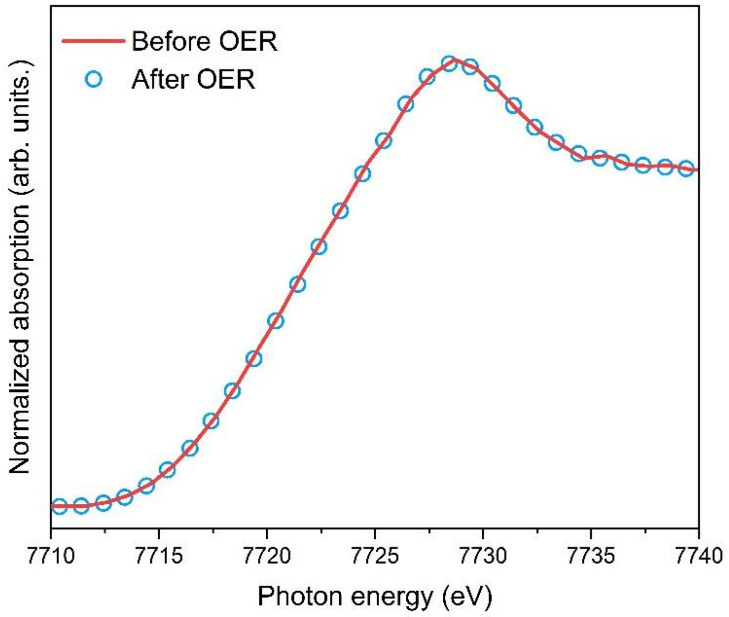

Supplementary Figure S18. Normalized Co *K*-edge XAS spectra of S-CoOOH before (solid line) and after OER (open circle) under light irradiation. Source data for Supplementary Fig.S18 are provided as a Source Data file.

Structural reconstruction is also excluded. As displayed in Supplementary Fig.S18, normalized Co *K*-edge XAS spectra of S-CoOOH before and after OER under light irradiation are nearly identical, indicating local structural stability under light. Besides, pre- and post-reaction XRD patterns and TEM images of S-CoOOH (Supplementary Figs.S19, S20) confirm that no phase transformation or surface reorganization occurs during OER under light. In addition, during the OER test under light irradiation, S-CoOOH exhibits reversible behavior in electrochemical performance, further confirming that no additional structural reconstruction occurs (Supplementary Fig.S3).

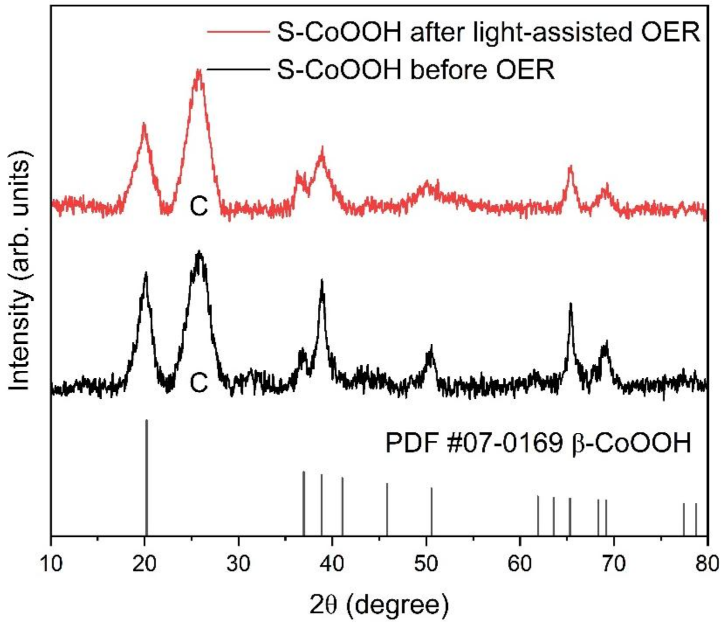

Supplementary Figure S19. XRD patterns of S-CoOOH based on carbon cloth before (bottom) and after (top) light-assisted OER test, with standard β-CoOOH listed (PDF #07-0169). Source data for Supplementary Fig.S19 are provided as a Source Data file.

Supplementary Figure S19 displays XRD patterns of S-CoOOH before and after light-assisted OER test. Prior to the OER test, S-CoOOH was subjected to a 10h electrochemical activation, during which cobalt sulfide pre-catalyst is fully reconstructed into rhombohedral β-CoOOH (space group R-3m, JCPDS No. 07-0169), as evidenced by the XRD pattern (bottom curve). The diffraction peaks at 20.24°, 36.98°, 38.89°, 50.58°, 65.34°, and 69.17° can be indexed to (003), (101), (012), (015), (110), and (113) crystal planes of β-CoOOH, respectively. The diffraction peak at 25.83° can be attributed to (002) crystal plane of carbon cloth substrate. After the light-assisted OER test, the XRD pattern (top curve) exhibits neither discernible shifts in peak positions nor the emergence of new diffraction peaks, indicating that the catalyst remains in β-CoOOH phase without detectable phase transformation.

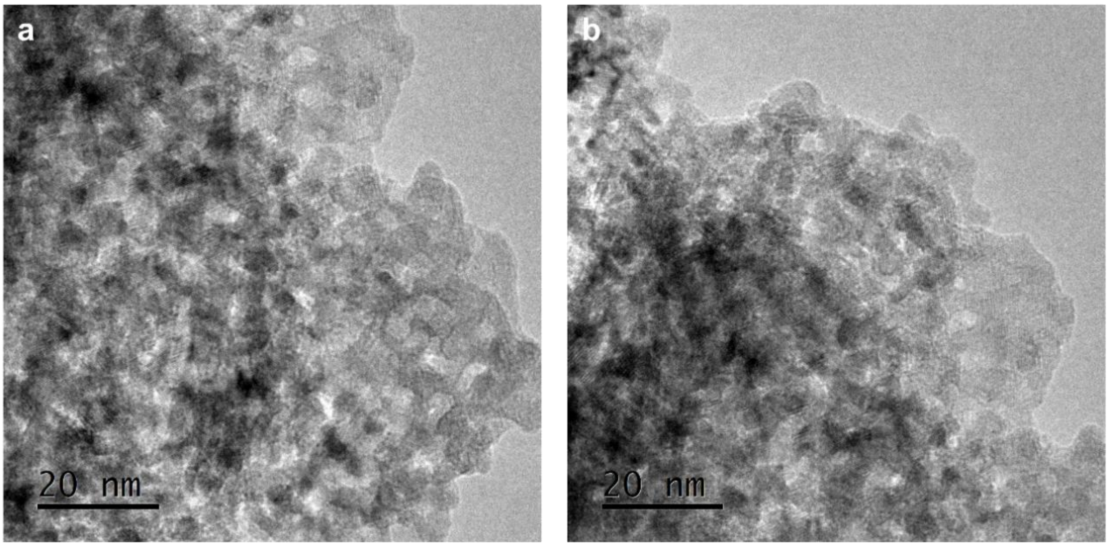

Supplementary Figure S20. The high-resolution transmission electron microscopy (HR-TEM) images of S-CoOOH before (**a**) and after (**b**) light-assisted OER test.

Supplementary Figure S20 presents the high-resolution transmission electron microscopy (HR-TEM) images of S-CoOOH collected before and after light-assisted OER process. The images show comparable particle sizes and morphologies before and after OER, without the appearance of any additional surface layer, thereby excluding significant surface reorganization.

Operando Co *K*-edge XAS experiments

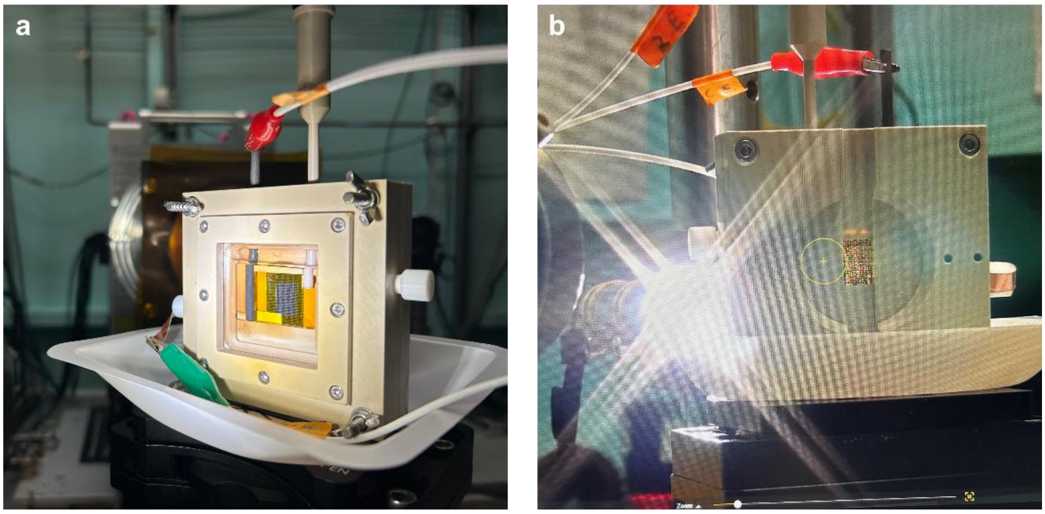

Supplementary Figure S21. Customized electrochemical device for *operando* X-ray absorption spectroscopy (XAS) measurements. Digital photographs of this device assembled on the XAS measurement instrument, employing the sample based on carbon cloth as the working electrode, Ag/AgCl as the reference electrode, along with carbon rod as the counter electrode.

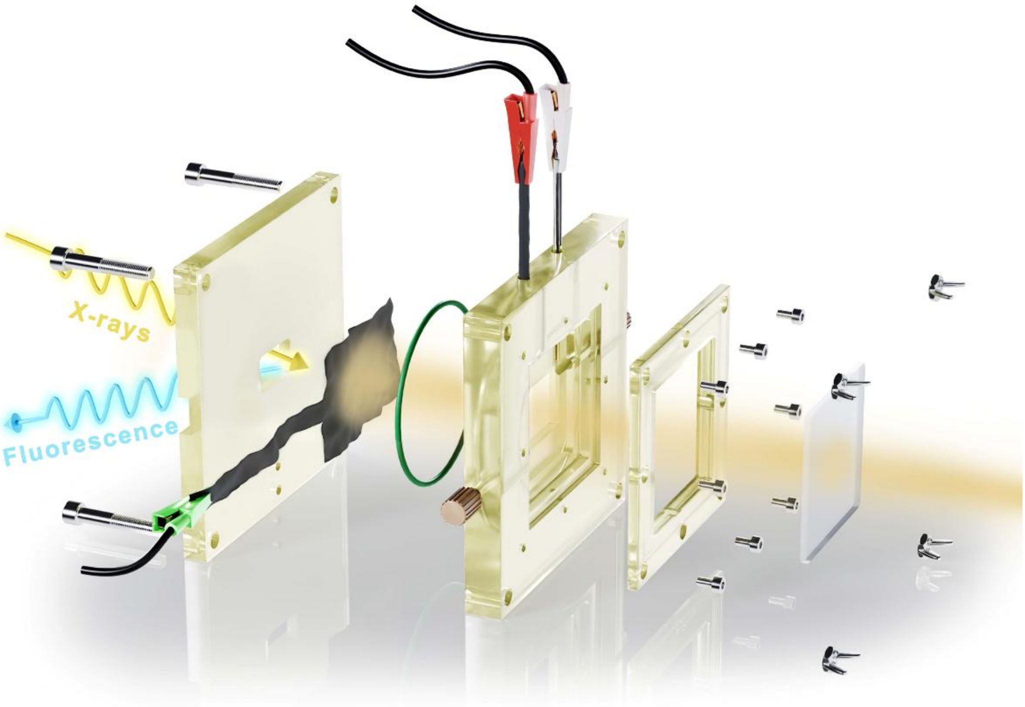

Supplementary Figure S22. Schematic illustration of the *operando* X-ray absorption spectroscopy (XAS) setup. The setup operates in fluorescence mode and is sealed with a 2×2 cm^2 Kapton window to allow light illumination on the sample. This three-electrode setup comprises a carbon cloth-supported working electrode, a carbon rod counter electrode, and an Ag/AgCl reference electrode.

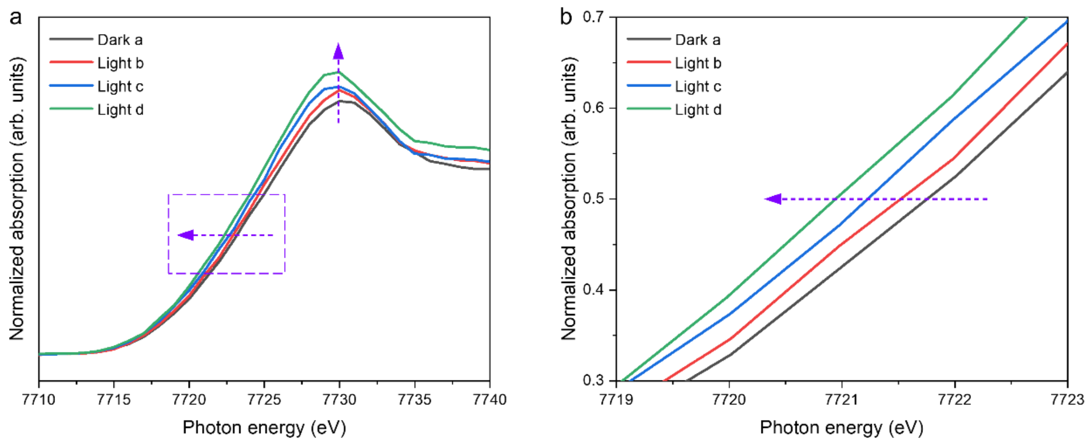

Supplementary Figure S23. *Operando* Co *K*-edge XAS spectra of S-CoOOH during OER under light. a Spectra recorded from dark to illumination at four selected points: Dark a, Light b, Light c, and Light d. The dashed purple lines reveal the shifting trends in the XAS spectra. **b** Enlarged *operando* Co *K*-edge XAS spectra near the absorption edge, extracted from **Fig.S23a.** Source data for Supplementary Fig.S23 are provided as a Source Data file.

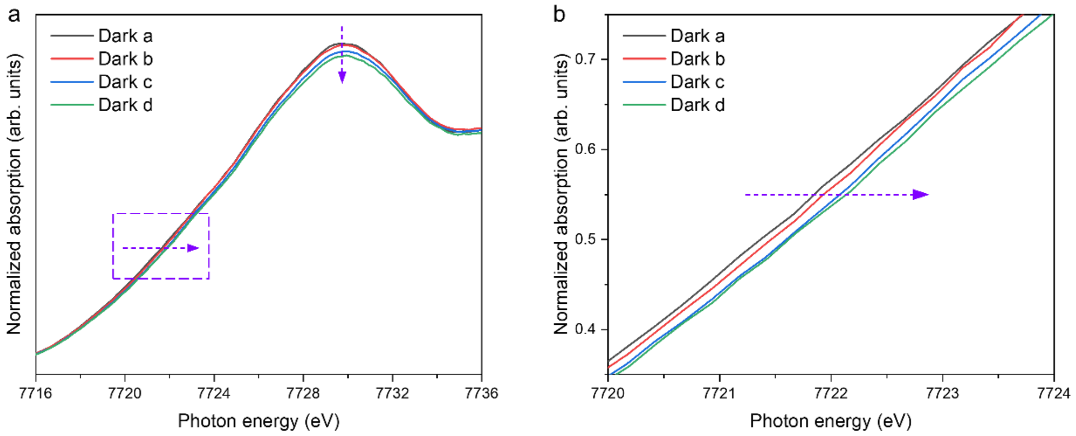

Supplementary Figure S24. *Operando* Co *K*-edge XAS spectra of S-CoOOH during OER in the dark. a Spectra successively recorded during OER in the dark at four selected points: Dark a, Dark b, Dark c, and Dark d. **b** Enlarged Co *K*-edge XAS absorption edge region, extracted from **Fig.S24a.** Source data for Supplementary Fig.S24 are provided as a Source Data file.

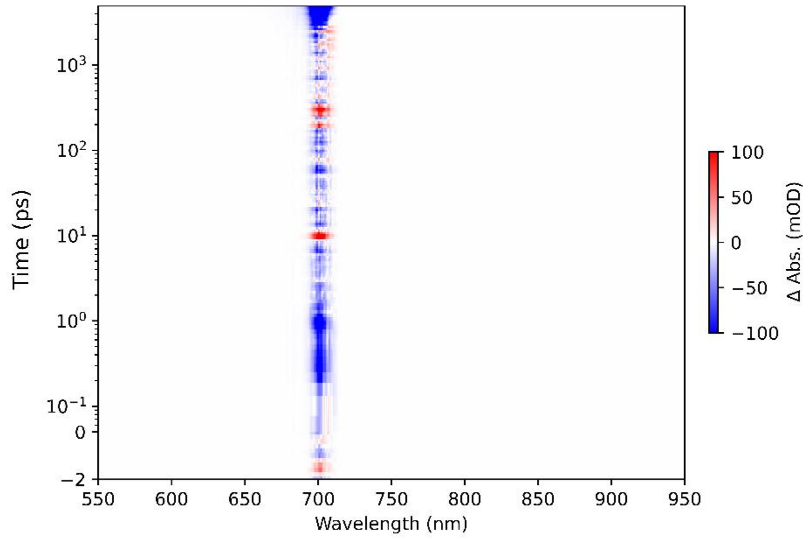

Supplementary Figure S25. Fs-transient absorption plot of S-CoOOH under 700 nm excitation. 2D-false color plot in 550-950 nm using 700 nm as an excitation source, with the signal around 700 nm arising from excitation light scattering. Source data for Supplementary Fig.S25 are provided as a Source Data file.

The transient absorption (TA) spectroscopy of S-CoOOH was performed using 700 nm as an excitation source (Supplementary Figure S25). No clear transient features are observed in the 2D TA map, except for a signal around 700 nm arising from excitation light scattering. This is reasonable because the light-induced electron transfer in our system is not a conventional photophysical excitation. In our proposed mechanism, S-CoOOH first adsorbs OH^- and undergoes deprotonation to form the $^*O$ intermediate under applied potential. Light then acts on this deprotonated $^*O$ states species, generating 7-electron oxygen ($^*O^-$). Two adjacent $^*O^-$ subsequently undergo direct O-O coupling, which stabilizes the photoinduced state. This light-induced electron transfer is coupled to the OER process and is regarded as a part of O-O coupling step. In the absence of bias, the deprotonated $^*O$ intermediate is not formed and no light-induced electron transfer is triggered, which explain the absence of observable TA signals.

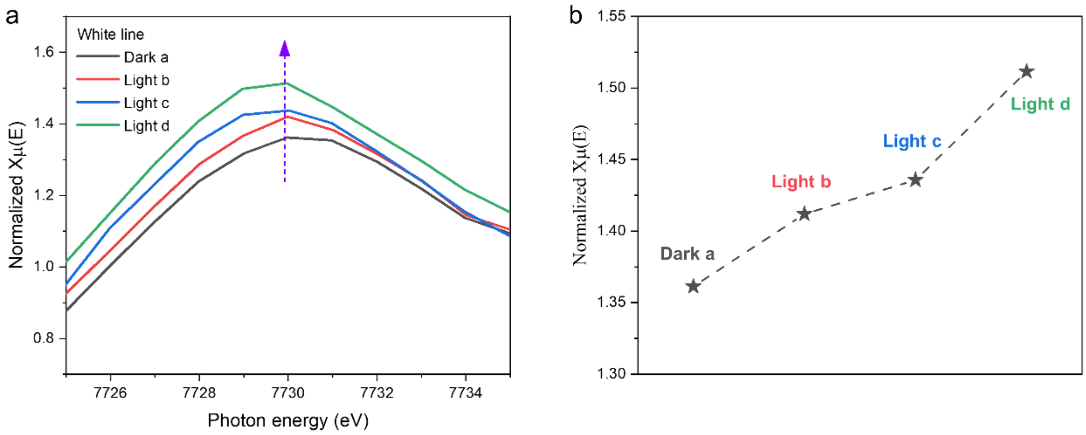

Supplementary Figure S26. Enlarged *operando* Co *K*-edge XAS spectra in white line region from 7725 to 7735 eV. a Spectra extracted from **Fig.2b,** recorded from dark to illumination at four selected points: Dark a, Light b, Light c, and Light d. The dashed purple arrows indicate the shifting direction of the white line peak with prolonged light illumination. **b** White line intensity of S-CoOOH subjected to light, recorded during OER from dark to illumination at four selected points: Dark a, Light b, Light c, and Light d. Source data for Supplementary Fig.S26 are provided as a Source Data file.

As shown in Supplementary Fig.S26a,b, *Operando* X-ray absorption spectroscopy (XAS) results for S-CoOOH demonstrate a progressive intensification of the white line with prolonged light irradiation. A higher white line intensity typically suggests a more regular CoO_6 octahedral structure[3]. Hence, this observation implies that during light irradiation, the Co centers retain their octahedral configuration. This result is contrary to the phenomenon observed in our prior work on NiOOH nanoribbon (NR-NiOOH)[2]. In that study, *operando* Ni *K*-edge XAS revealed weakening of the white line under light irradiation, indicating a structural transformation from octahedral NiO_6 to square planar NiO_4 geometry. This structural change led to the formation of two adjacent non-bonding oxygen (O_{NB}) states within the NiO_6 lattice, which combined to form an (O-O) coupling. In contrast, intensification of the white line typically suggests a more regular CoO_6 octahedral structure, rather than a transition to square planar geometry. The difference in white line intensity variations under light illumination between NiOOH and CoOOH stems from their 3*d* electron configurations, that is $3d^8$ in Ni^{2+} and $3d^7$ in Co^{2+}. Ni^{2+} favors a square planar geometry, whereas Co^{2+} does not due to its $3d^7$ electron configuration with unpaired

electrons, making the square planar geometry rare in Co^{2+} complexes[4].

Our work does not involve any change in translational or time-reversal symmetry. As shown in *operando* Co *K*-edge XAS spectra, under light irradiation, S-CoOOH only shows an increased white-line intensity (Supplementary Fig.S26), confirming that the octahedral structure of S-CoOOH remains intact under light irradiation. It indicates that during light irradiation, the translation symmetry is preserved without any structural reconstruction. Besides, the complete reversibility of the light-induced enhancement (Supplementary Fig.S3) demonstrates that the modulation of the electronic structure by light is fully reversible, indicating that no time-reversal symmetry breaking occurs under light irradiation.

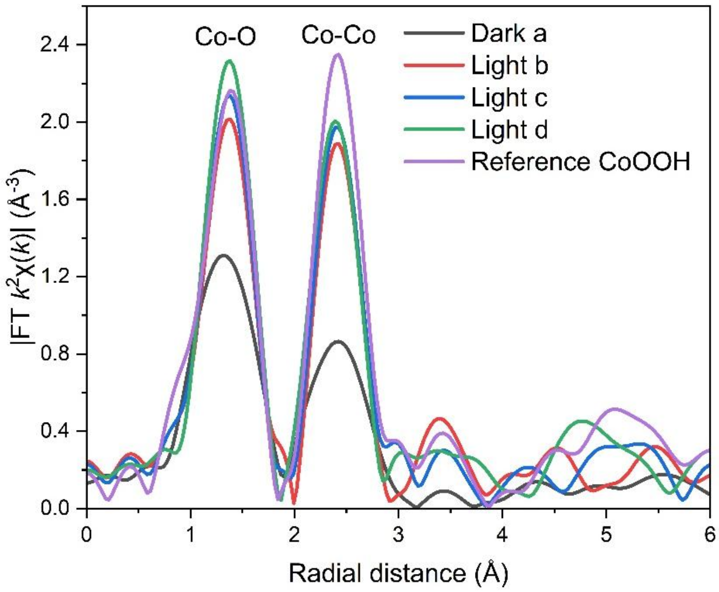

Supplementary Figure S27. Normalized Co *K*-edge Fourier transformed extended X-ray absorption fine structure (FT-EXAFS) spectra, collected during OER under light irradiation (Dark a, Light b, Light c, and Light d), with Reference CoOOH used as the standard. Source data for Supplementary Fig.S27 are provided as a Source Data file.

TMA$^+$ probe experiments in the dark

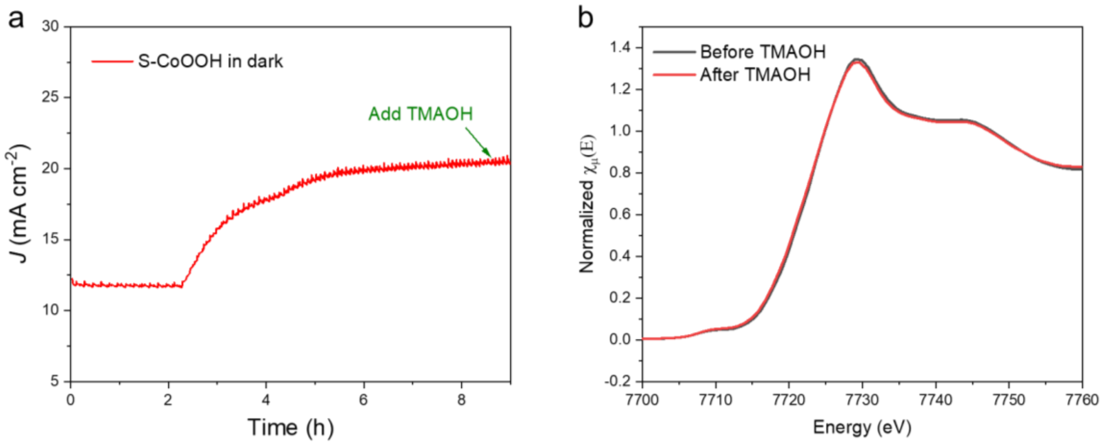

Supplementary Figure S28. Examining the presence of non-bonding oxygen (O_{NB}) states in S-CoOOH during OER in the dark using TMAOH. a The electrochemical curves of S-CoOOH in response to the addition of TMAOH in the dark. **b** Co *K*-edge XAS spectra of S-CoOOH before and after adding TMAOH in the dark. Source data for Supplementary Fig.S28 are provided as a Source Data file.

Operando DEMS with ^{18}O isotope measurements

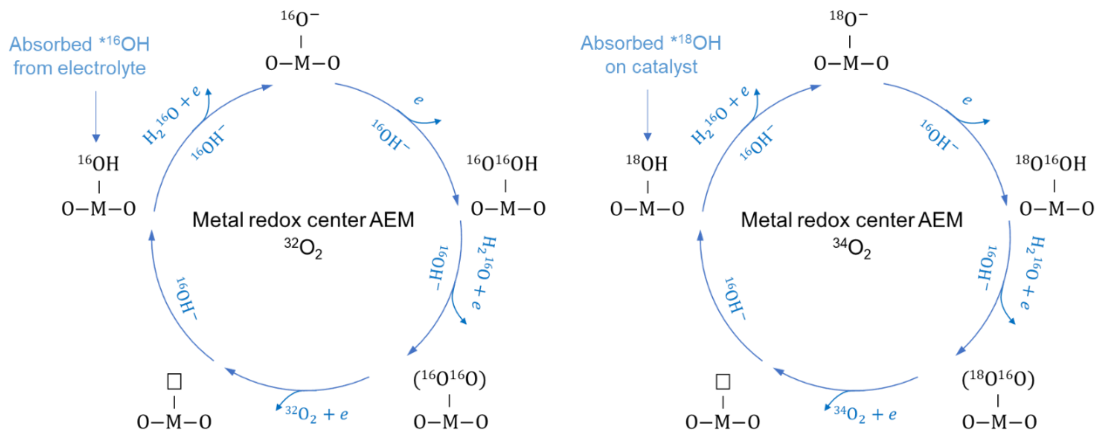

Supplementary Figure S29. Speculated mechanism of different ^{18}O isotope-labeled O_2 products in metal (oxy)hydroxide, which is predicted to follow conventional metal redox center adsorbate evolution mechanism (AEM). $^{34}O_2$ ($^{16}O^{18}O$) and $^{32}O_2$ ($^{16}O^{16}O$) evolution pathways in the electrolyte using $H_2{}^{16}O$ as solvent.

If metal Co exhibits redox activity, the catalyst would adsorb $^{*16}OH$ from $K^{16}OH$ electrolyte, leading to the formation of $^{*16}O^{16}OH$ ($^{*16}O^{18}OH$) intermediates and the eventual release of $^{32}O_2$ or $^{34}O_2$, with no detection of $^{36}O_2$ ($^{*18}O^{18}OH$) (Supplementary Fig.S29). In the dark, S-CoOOH exhibits no $^{36}O_2$ signal (Fig.4a), while $^{32}O_2$ and $^{34}O_2$ signals are present (Supplementary Fig.S30), which confirms that S-CoOOH follows metal redox center adsorbate evolution mechanism (AEM) under dark conditions.

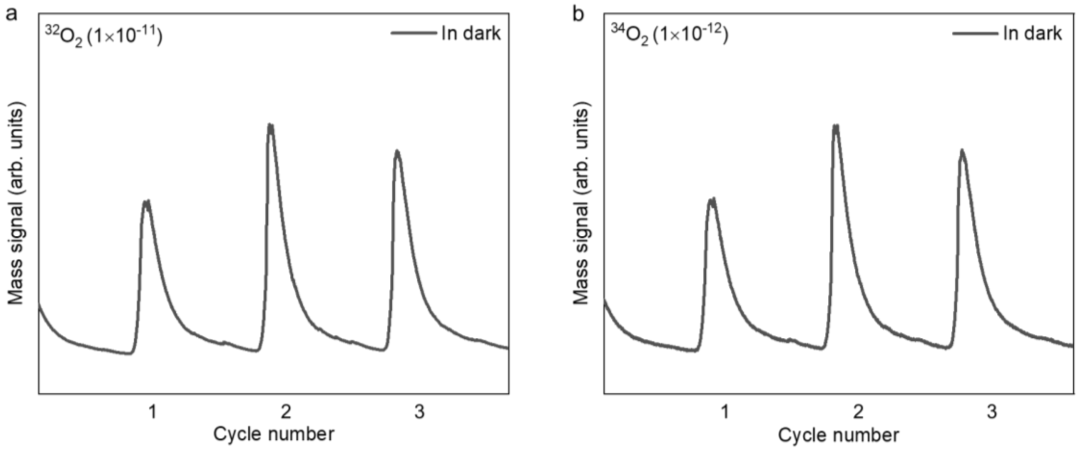

Supplementary Figure S30. OER mechanism analysis based on *operando* differential electrochemical mass spectrometry (DEMS) with ^{18}O isotope labeling measurements. DEMS signals of $^{32}O_2$ ($^{16}O^{16}O$) (a) and $^{34}O_2$ ($^{16}O^{18}O$) (b) products for S-CoOOH in the dark. Source data for Supplementary Fig.S30 are provided as a Source Data file.

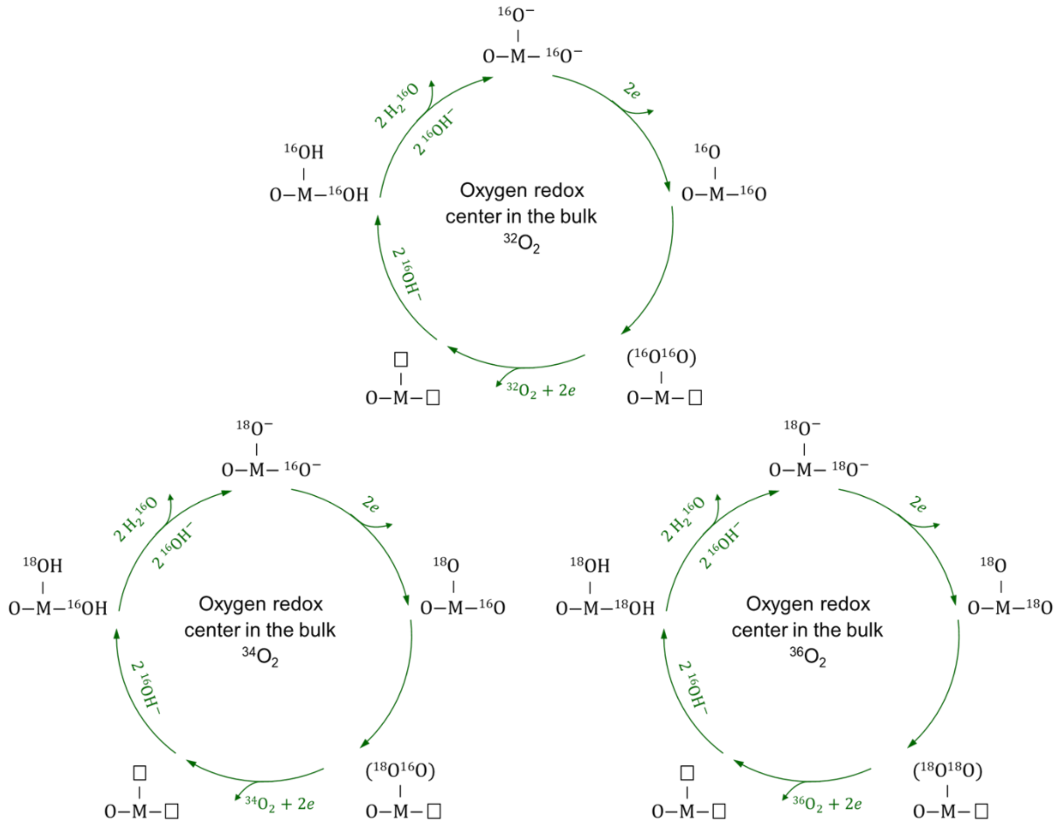

Supplementary Figure S31. Speculated mechanism of different ^{18}O isotope-labeled O_2 products in metal (oxy)hydroxide, which is predicted to enable oxygen redox activity in the bulk of metal (oxy)hydroxide. $^{36}O_2$ ($^{18}O^{18}O$), $^{34}O_2$ ($^{16}O^{18}O$), and $^{32}O_2$ ($^{16}O^{16}O$) evolution pathways in the electrolyte using $H_2{}^{16}O$ as solvent.

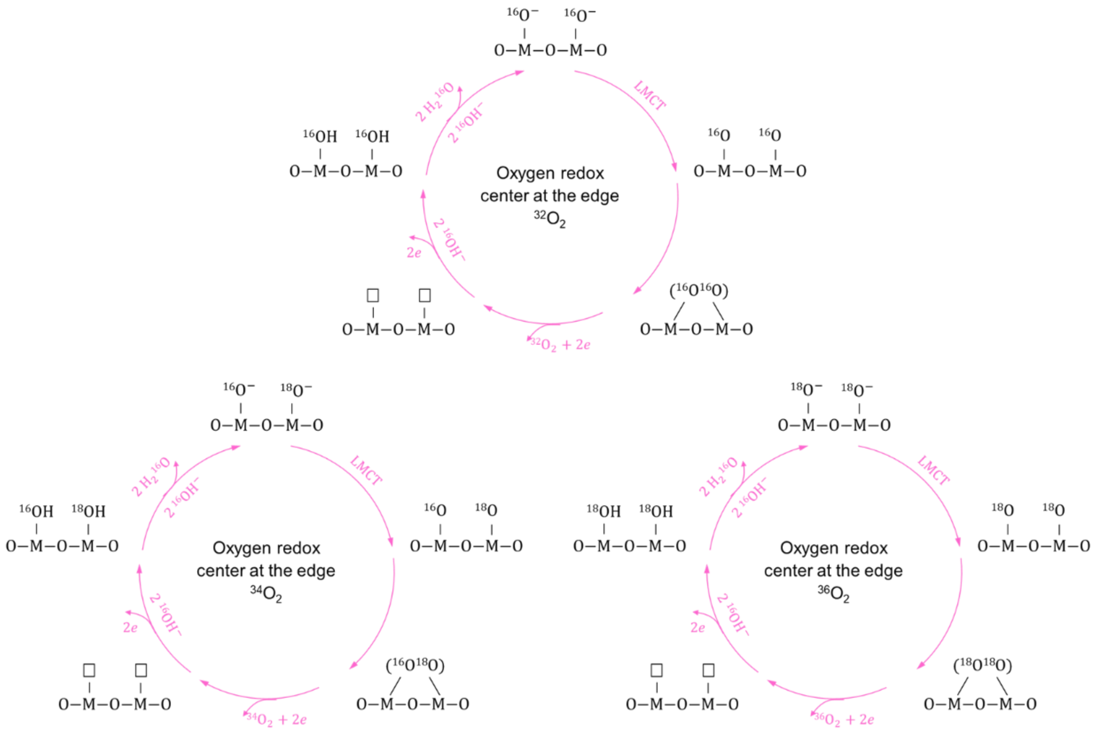

Supplementary Figure S32. Speculated mechanism of different ^{18}O isotope-labeled O_2 products in metal (oxy)hydroxide, which is predicted to enable oxygen redox activity at the edge of metal (oxy)hydroxide. $^{36}O_2$ ($^{18}O^{18}O$), $^{34}O_2$ ($^{16}O^{18}O$), and $^{32}O_2$ ($^{16}O^{16}O$) evolution pathways in the electrolyte using $H_2{}^{16}O$ as solvent.

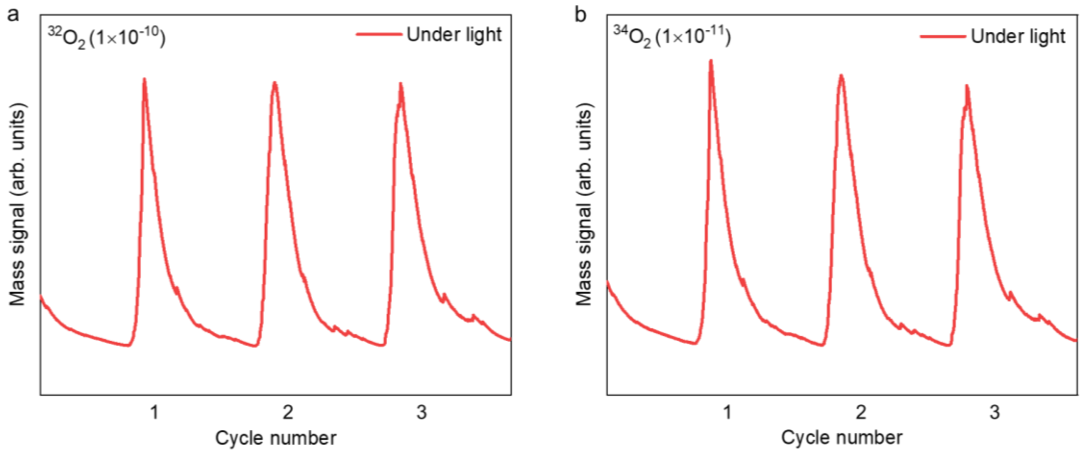

Supplementary Figure S33. OER mechanism analysis based on *operando* DEMS with ^{18}O isotope labeling measurements. DEMS signals of $^{32}O_2$ ($^{16}O^{16}O$) (a) and $^{34}O_2$ ($^{16}O^{18}O$) (b) products for S-CoOOH under light. Source data for Supplementary Fig.S33 are provided as a Source Data file.

Optimized model for simulations

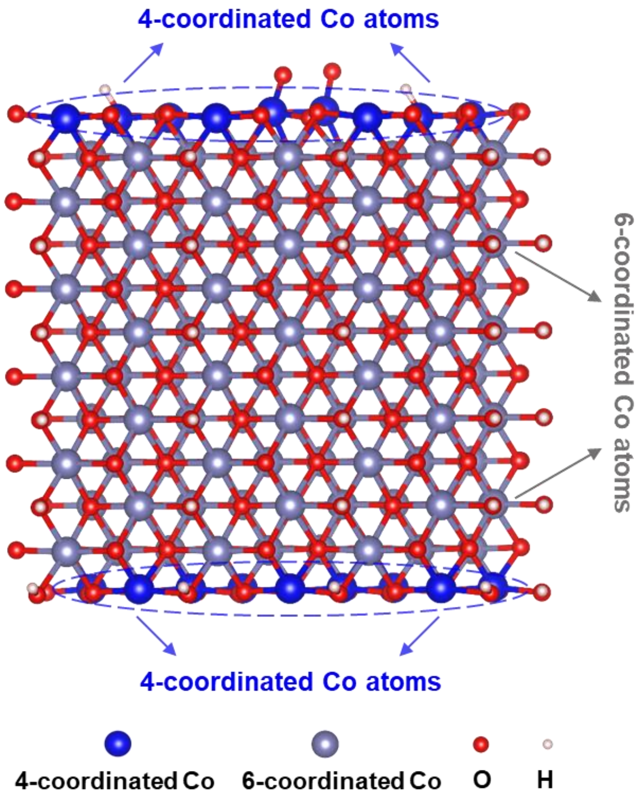

Supplementary Figure S34. The optimized model of S-CoOOH, with 4-coordinated Co atoms in blue and 6-coordinated ones in grey.

The S-CoOOH model was constructed following the procedure described in our previous work[3]. The CoOOH edge structure can be generated by cutting the CoOOH super cell along either the x-direction (XR) or the y-direction (YR), yielding two distinct edge structures: one with 3 or 5 coordination at edge and the other with 4 coordination at edge. Density functional theory (DFT) calculations were then performed to compare their energies, revealing that the structure with 4 coordination at edge has lower energy (Supplementary Figs.S34 and S35).

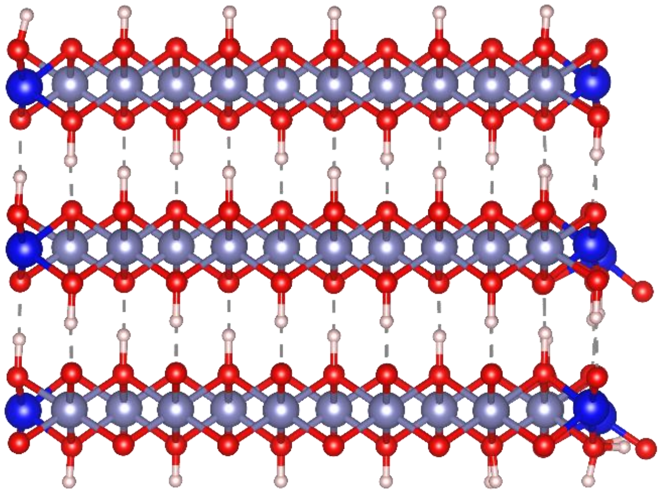

Supplementary Figure S35. Top view of the optimized S-CoOOH model, with 4-coordinated Co atoms in blue and 6-coordinated ones in grey.

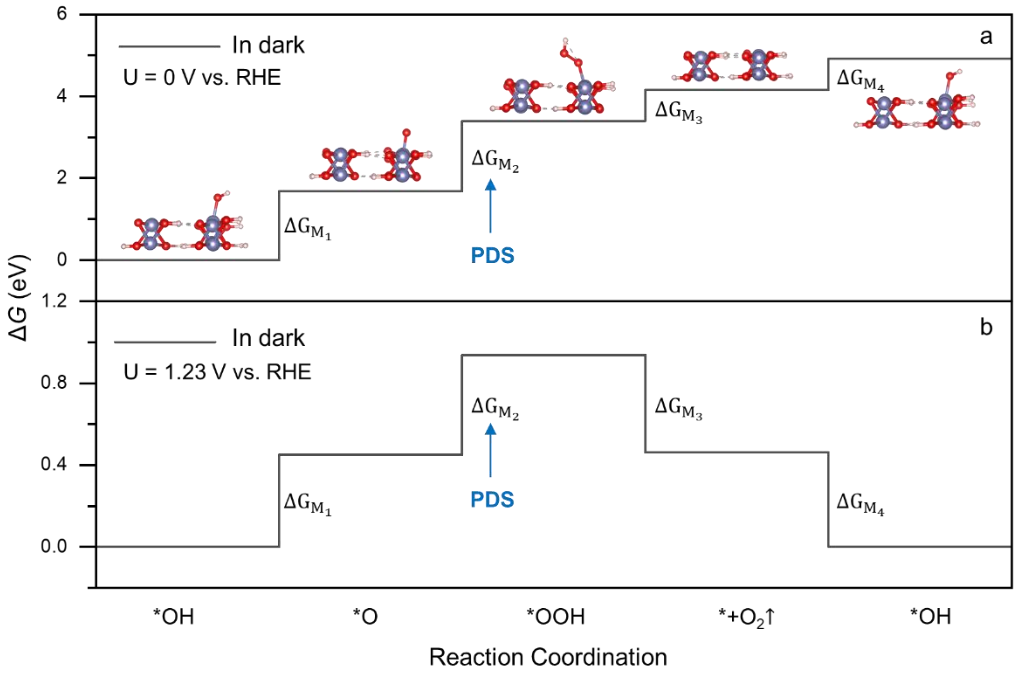

Supplementary Figure S36. Calculated reaction free energy diagrams of the OER pathway in dark (metal redox activity) on S-CoOOH at U = 0 V (**a**) and 1.23 V (**b**) vs. RHE, with the potential-determining step (PDS) indicated by a blue arrow (where * refers to the metal sites). Source data for Supplementary Fig.S36 are provided as a Source Data file.

The thermodynamic calculations were performed using the optPBE-vdW functional with the van der Waals (vdW) correction. The Gibbs free energy change (ΔG) for each elemental step in OER can be obtained by the following formula:

$$\Delta G(U_{\mathrm{RHE}}) = \Delta E + \Delta ZPE - T\Delta S$$

where ΔE is the electronic energy difference, ΔZPE is the zero-point energy difference, and ΔS is the entropy change. The relationship between ΔG referenced to standard hydrogen electrode (SHE) and reversible hydrogen electrode (RHE) can be described by a pH correction term: $\Delta G(U_{\mathrm{RHE}}) = \Delta G(U_{\mathrm{SHE}}) + \Delta G_{\mathrm{pH}}$, where $\Delta G_{\mathrm{pH}} = \frac{k_{\mathrm{B}}\mathrm{TIn10}}{e}\mathrm{pH}$, with k_{B} is the Boltzmann constant and T = 300 K.

The associated electron transfer reactions in dark and under light are composed of the following elementary steps, respectively:

In dark (metal redox activity):

$\Delta G_{\mathrm{M}_1} = \Delta G_{[*\mathrm{O}]} - \Delta G_{[*\mathrm{OH}]} - \mathrm{eU}$

$\Delta G_{\mathrm{M}_2} = \Delta G_{[*\mathrm{OOH}]} - \Delta G_{[*\mathrm{O}]} - \mathrm{eU}$

$\Delta G_{\mathrm{M}_3} = 4.92\ \mathrm{eV} - \Delta G_{[*]} + \Delta G_{[*\mathrm{OOH}]} - \mathrm{eU}$

$\Delta G_{\mathrm{M}_4} = \Delta G_{[*\mathrm{OH}]} - \Delta G_{[*]} - \mathrm{eU}$

Under light (oxygen redox activity):

$\Delta G_{\mathrm{O}_1} = \Delta G_{[*\mathrm{OH}+*\mathrm{O}]} - \Delta G_{[*\mathrm{OH}+*\mathrm{OH}]} - \mathrm{eU}$

$\Delta G_{\mathrm{O}_2} = \Delta G_{[*\mathrm{O}+*\mathrm{O}]} - \Delta G_{[*\mathrm{OH}+*\mathrm{O}]} - \mathrm{eU}$

$\Delta G_{\mathrm{O}_3} = \Delta G_{[*\mathrm{O}-\mathrm{O}]} - \Delta G_{[*\mathrm{O}+*\mathrm{O}]}$ (Light dominated process)

$\Delta G_{\mathrm{O}_4} = 4.92\ \mathrm{eV} - \Delta G_{[*\mathrm{OH}]} + \Delta G_{[*\mathrm{O}-\mathrm{O}]} - \mathrm{eU}$

$\Delta G_{\mathrm{O}_5} = \Delta G_{[*\mathrm{OH}+*\mathrm{OH}]} - \Delta G_{[*\mathrm{OH}]} - \mathrm{eU}$

To maintain numerical stability for the large system (over 400 atoms), the linear mixing scheme was enforced for electronic density, and the LMIXTAU tag was enabled to stabilize the density mixing in the presence of U parameters. To account for the electrolyte environment and long-range electrostatic screening, an implicit solvation model as implemented in VASPsol was employed[5]. The solvent was treated as a continuum dielectric medium with a relative permittivity of ε_{b} = 78.4, representing the aqueous phase. The solute-solvent interface was defined by a critical density threshold of n_{c} = 0.0025 Å[6]. To simulate the ionic screening effect in the electrolyte (e.g., 1.0 M KOH), the linearized Poisson-Boltzmann equation was solved by specifying a Debye screening length (λ_{D}) of 3.04 Å[7]. This parameter describes the characteristic thickness of the diffuse part of the electrochemical double layer (EDL), allowing for a more realistic description of the interfacial energetics during the oxygen evolution reaction process.

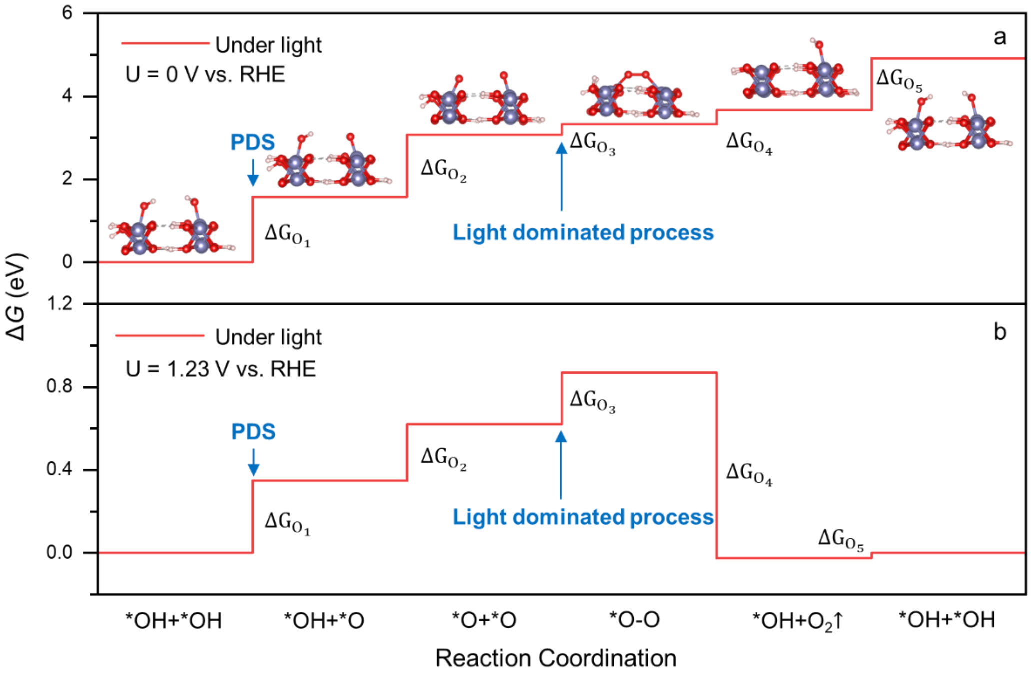

Supplementary Figure S37. Calculated reaction free energy diagrams of the OER pathway under light (oxygen redox activity) on S-CoOOH at U = 0 V (**a**) and 1.23 V (**b**) vs. RHE, with the potential-determining step (PDS) indicated by a blue arrow (where * refers to the metal sites). The light dominated process occurs at O-O coupling step. Source data for Supplementary Fig.S37 are provided as a Source Data file.

The role of light is manifested at the O-O coupling step (*O+*O→*O-O, marked in a blue arrow). Under light illumination, electrons are transferred from deprotonated *O to Co, generating 7-electron oxygen ($^*O^-$); then, two adjacent ones couple to form direct O-O coupling (*O-O), thereby stabilizing the oxygen intermediates. The thermodynamic shifting of the potential-determining step (PDS), accompanied by a decrease in overpotential from 0.49 V for *OOH formation in dark to 0.34 V for proton transfer under light, reflects that light favors a reaction pathway involving direct O-O coupling. This shift provides indirect computational evidence supporting light-triggered electron transfer.

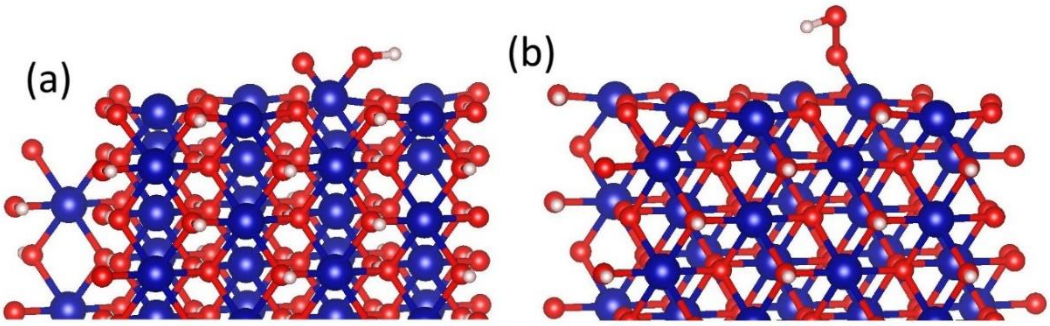

Supplementary Figure S38. The optimized models of *OH adsorption at Co site (**a**) and O_{NB} site (**b**).

Supplementary Fig.S38 presents the optimized models of *OH adsorption at Co site (**a**) and O_{NB} site (**b**). The calculations of *OH adsorption on cobalt and O_{NB} sites reveal that, the adsorption on the O_{NB} site (-0.649 eV) is more energetically favorable than on cobalt site (0 eV) based on the calculated relative energy.

PDOS results for Co 3*d* and O 2*p* orbitals

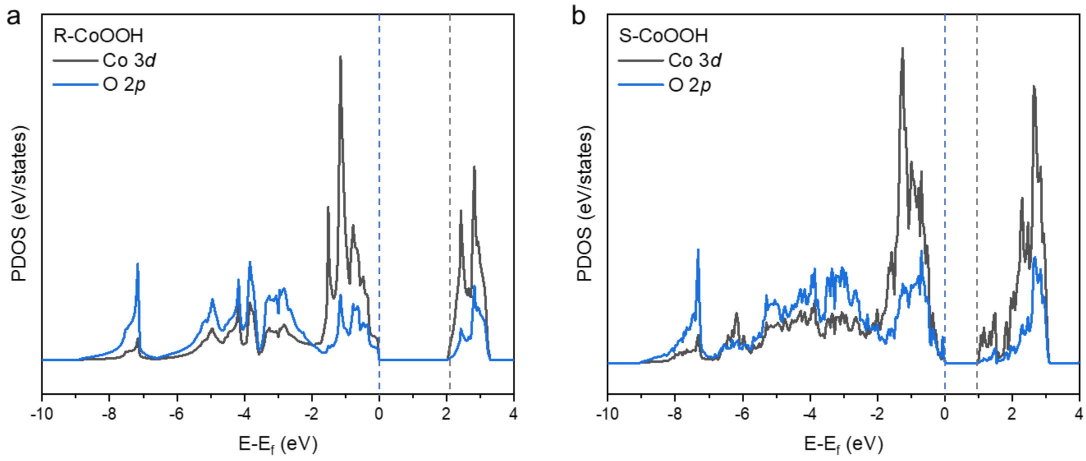

Supplementary Figure S39. PDOS of cobalt 3*d* and oxygen 2*p* orbitals in both R-CoOOH (**a**) and S-CoOOH (**b**). Source data for Supplementary Fig.S39 are provided as a Source Data file.

It is noted in the main text that enabling ligand-to-metal charge transfer (LMCT) in CoOOH necessitates the presence of empty antibonding orbitals near the Fermi level, which implies that Co 3*d* and 4*s* orbitals should not completely overlap. This can be explained by the fact that if Co 3*d* and 4*s* orbitals were to completely overlap, the electronic states within the overlapping region would primarily arise from the contributions of Co 4*s* orbitals. However, electron transfer from (M-O) bonds to 4*s* orbitals is improbable, as electrons preferentially occupy 3*d* orbital over 4*s* orbital.

Moreover, beyond whether non-overlapping regions exist between Co 3*d* and 4*s* orbitals, the feasibility of light-triggered LMCT is further assessed from an energetic perspective. If such a process can occur in both samples, the excitation energy required in each case is estimated. The LMCT in CoOOH corresponds to electron transfer from (M-O) to Co 3*d* orbitals. As shown in Supplementary Fig.S39, the energy barriers between (M-O) and Co 3*d* orbitals are 2.05 eV for R-CoOOH and 0.98 eV for S-CoOOH (marked with dashed lines). Accordingly, the excitation energy required for LMCT in S-CoOOH (0.98 eV) is significantly lower than that in R-CoOOH (2.05 eV).

Photophysical property of S-CoOOH

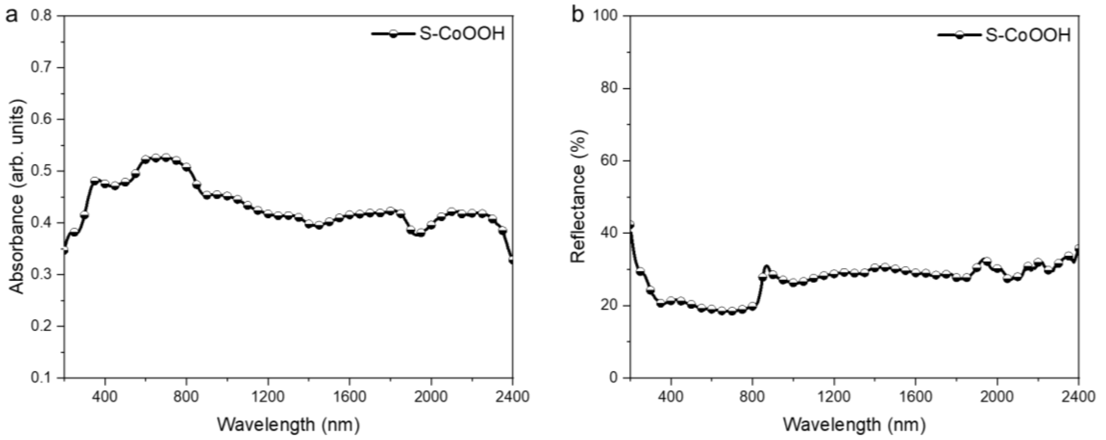

Supplementary Figure S40. UV-Vis absorption (a) and reflectance spectra (b) of S-CoOOH powder measured in the 200-2400 nm wavelength range. Source data for Supplementary Fig.S40 are provided as a Source Data file.

Schematic of DEMS electrochemical cell

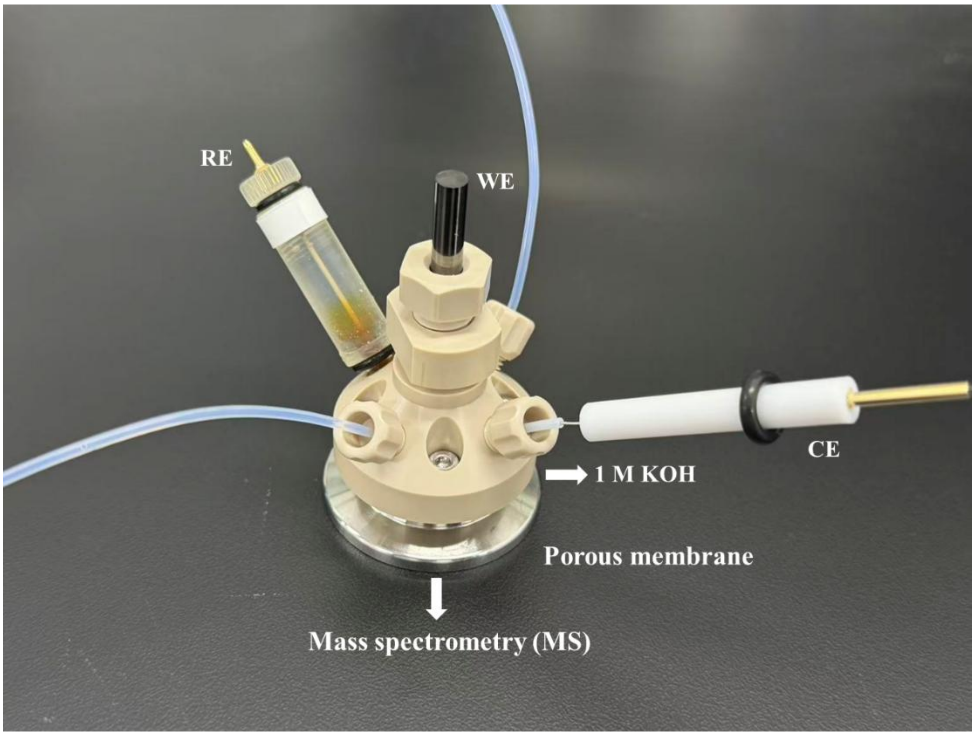

Supplementary Figure S41. Digital photograph of the DEMS electrochemical cell. CE: Pt wire counter electrode, WE: working electrode wrapped with a carbon rod, RE: Ag/AgCl reference electrode. The interface is constituted by a porous membrane supported on a stainless-steel frit.

Elemental analysis of S-CoOOH

Supplementary Table S1. Inductively coupled plasma (ICP) result of S-CoOOH, showing the corresponding mass/atomic ratios. Source data for Supplementary Table S1 are provided as a Source Data file.

Element	Co	S
Mass ratio (w/w%)	39.94	0.41
Atomic ratio (at%)	98.16	1.84

Summary of OER performance of cobalt-based catalysts

Supplementary Table S2. OER catalytic performance comparison among S-CoOOH subjected to light irradiation and previously reported cobalt-based catalysts. η stands for overpotential. WE, CE, and RE represent working, counter, and reference electrodes, respectively. Source data are provided.

Catalyst	Cell configuration (WE|CE|RE)	Electrolyte	Loading mass ($mg\cdot cm^{-2}$)	Electrolyte resistance (Ω)	η (mV) at 10 $mA\cdot cm^{-2}$	Substrate	Year	Ref
**S-CoOOH under light**	**CoOOH | Pt | Hg/HgO**	**1 M KOH**	**0.84**	**0.75**	**194**	**Carbon cloth**	**2025**	**Our work**
Co_3O_4	Co_3O_4 | carbon rod | Hg/HgO	1 M KOH	0.3	2.0 ± 0.5	230	Carbon cloth	2025	8
CoFe-NBs	CoFe-NBs | graphite rod | Hg/HgO	1 M KOH	0.05	6.62 ± 0.15	248	Carbon paper	2025	9
V-$Co_2P_4P_{12}$	V-$Co_2P_4P_{12}$ | Pt | Hg/HgO	1 M KOH	0.3	2.2	288	Carbon cloth	2024	10
CoFe@Co oxysulfide	CoFe@Co oxysulfide | Pt| Hg/HgO	1 M KOH	NA	3.01	280	Carbon cloth	2024	11
Co single atom	Co single atom | Graphite sheet | Ag/AgCl	1 M KOH	0.205	13	351	Carbon paper	2023	12
Co_3O_4-V_o	Co_3O_4-V_o | graphite rod | Hg/HgO	1 M KOH	0.34	6	262	Glassy carbon	2023	13
CoOOH	CoOOH | Carbon rod | Hg/HgO	1 M KOH	NA	6.1	355	Glassy carbon	2023	14

Catalyst	Cell configuration (WE|CE|RE)	Electrolyte	Loading mass ($mg \cdot cm^{-2}$)	Electrolyte resistance (Ω)	η (mV) at 10 $mA \cdot cm^{-2}$	Substrate	Year	Ref
Ru/Co_3O_{4-x}	Ru/Co_3O_{4-x} | Pt wire | Hg/HgO	1 M KOH	NA	NA	280	Glassy carbon	2023	15
$Co_{0.5}Fe_{0.5}$ LDH	$Co_{0.5}Fe_{0.5}$ LDH | graphitic rod | Hg/HgO	1 M KOH	NA	1.67	270	Carbon cloth	2023	16
$La_xSr_{1-x}CoO_{3-\delta}$	$La_xSr_{1-x}CoO_{3-\delta}$ | Pt foil | Hg/HgO	1 M KOH	0.028	NA	250	Glassy carbon	2023	17
$MoCo_xO_y$	$MoCo_xO_y$ | Graphite rod | HgO/Hg	1 M KOH	0.15	NA	282	Carbon cloth	2022	18
$Au\text{-}Co(OH)_2$	$Au\text{-}Co(OH)_2$ | Graphite rod | Ag/AgCl	1 M KOH	NA	NA	260	Glassy carbon	2022	19
CoOOH/CoS	CoOOH/CoS | carbon rod | Hg/HgO	1 M KOH	NA	3.35	273	Carbon cloth	2022	20
CoOOH nanosheet	CoOOH nanosheet | Pt foil | Hg/HgO	1 M KOH	0.0001-0.002	9.36	426	Carbon-coated chip	2022	21
$UCoO_4$	$UCoO_4$ | Pt wire | Hg/HgO	1 M KOH	NA	NA	250	Glassy carbon	2022	22
Co_3O_4/Mo_2N	Co_3O_4/Mo_2N | Graphite rod | Hg/HgO	1 M KOH	0.708	NA	300	Co PBA nanoframes	2022	23
Co_2FeO_4	Co_2FeO_4 | Pt wire | Ag/AgCl	1 M KOH	NA	NA	359	Glassy carbon	2022	24
IrO_2	IrO_2 | Carbon | Ag/AgCl	1M KOH	NA	NA	383	Carbon cloth	2022	25

Catalyst	Cell configuration (WE|CE|RE)	Electrolyte	Loading mass ($mg \cdot cm^{-2}$)	Electrolyte resistance (Ω)	η (mV) at 10 $mA \cdot cm^{-2}$	Substrate	Year	Ref
$Co(Fe)O_xH_y$	$Co(Fe)O_xH_y$ | Pt plate | Hg/HgO	1 M KOH	0.255	NA	350	Glassy carbon	2021	26
$LiCoO_{1.8}Cl_{0.2}$	$LiCoO_{1.8}Cl_{0.2}$ | coiled Pt wire | Ag/AgCl (ALS)	1 M KOH	0.4	15.8	276.8	Glassy carbon	2021	27
Co-Fe double-atom	Co-Fe double-atom | Pt plate | Hg/HgO	1 M KOH	NA	NA	319	Carbon cloth	2021	28
Co (High-spin)	Co (High-spin) | Graphite rod | Hg/Hg_2Cl_2 (saturated KCl)	1 M KOH	NA	NA	330	SiO_2/Si	2021	29
Co_3O_4@ZIF-67	Co_3O_4@ZIF-67 | Pt plate | Ag/AgCl (saturated)	1 M KOH	0.25	NA	262	ZIF-67 nanoplate	2020	30
$NiCo_2O_4$	$NiCo_2O_4$ | Pt wire | Ag/AgCl	1 M KOH	8	1.4	270 ± 3	Carbon paper	2020	31
$SrCoO_{3-\delta}$	$SrCoO_{3-\delta}$ | Pt wire | Ag/AgCl (4 M KCl)	1 M KOH	0.255	45	417	Glassy carbon	2020	32
$Fe-CoSe_2$	$Fe-CoSe_2$ | Graphite rod | Hg/HgO	1 M NaOH	NA	4.2	370	Glassy carbon	2020	33
$Zn_{0.2}Co_{0.8}OOH$	$Zn_{0.2}Co_{0.8}OOH$ | Graphite rod | Ag/AgCl (saturated KCl)	1 M KOH	0.204	5.4	241	Carbon paper	2019	34

Fitted atomic coordination number

Supplementary Table S3. FT-EXAFS fitting results of S-CoOOH collected during OER under light irradiation (Dark a, Light b, Light c, and Light d), with Reference CoOOH used as the standard. CN is the coordination number, σ^2 is the Debye-Waller factor, ΔE_0 is the shift in absorption edge energy, R is the bond length, and R-factor means Residual factor. Source data for Supplementary Table S3 are provided as a Source Data file.

Sample	path	CN	σ^2	ΔE_0	R	R-factor
Reference CoOOH	Co-O	6	0.0023	-3.4	1.90±0.01	0.0055
	Co-Co	6	0.0045	-4.5	2.85±0.01	
S-CoOOH at Dark a	Co-O	4.97±0.48	0.0130	-6.7	1.87±0.01	0.0183
	Co-Co	4.18±0.76	0.0170	3.9	2.91±0.04	
S-CoOOH at Light b	Co-O	5.78±0.58	0.0046	-2.2	1.89±0.02	0.0153
	Co-Co	4.43±0.55	0.0052	-2.0	2.85±0.02	
S-CoOOH at Light c	Co-O	5.90±0.42	0.0040	-3.5	1.88±0.01	0.0068
	Co-Co	4.83±0.42	0.0055	-1.4	2.84±0.02	
S-CoOOH at Light d	Co-O	5.97±0.50	0.0030	-4.3	1.88±0.02	0.0138
	Co-Co	5.16±0.57	0.0059	-3.7	2.83±0.02	

Calculated entropy and enthalpy values

Supplementary Table S4. Calculated entropy (T·S), enthalpy (H), and Gibbs free energy (G) values for the states before and after light-induced O-O coupling at 298.1 K. Source data for Supplementary Table S4 are provided as a Source Data file.

	entropy (T·S) J/mol	enthalpy (H) kcal/mol	Gibbs free energy (G) kcal/mol
Before light-induced O-O coupling (*O*O)	4443.833	8.942	7.879
After light-induced O-O coupling (*O-O)	8733.218	4.384	2.297
Δ	+4289.385	-4.559	-5.582

Light dominated process occurs at the O-O coupling step. To investigate the role of light, entropy (T·S), enthalpy (H), and Gibbs free energy (G) values were calculated for the states before (*O*O) and after (*O-O) this O-O coupling step. As displayed in Supplementary Table S4, *O-O exhibits a significant enthalpic stabilization relative to *O*O (ΔH = -4.558 kcal/mol), accompanied by an increased entropy contribution (ΔT·S = +4289.385 J/mol). Accordingly, the Gibbs free energy decreases by 5.582 kcal/mol, confirming that the transformation from *O*O to *O-O is thermodynamically favorable. Thus, these results suggest that light could affect the adsorption configuration, further altering the OER pathway.

Vibrational frequency analysis of S-CoOOH mode

Supplementary Table S5. The results of vibrational frequency analysis for identifying transition states. Source data for Supplementary Table S5 are provided as a Source Data file.

1 f	17.522950 THz	110.099942 2PiTHz	584.502699 cm^{-1}	72.469066 meV
2 f	13.881877 THz	87.222407 2PiTHz	463.049580 cm^{-1}	57.410805 meV
3 f	12.784254 THz	80.325839 2PiTHz	426.436823 cm^{-1}	52.871404 meV
4 f	11.620772 THz	73.015461 2PiTHz	387.627216 cm^{-1}	48.059628 meV
5 f	10.894075 THz	68.449493 2PiTHz	363.387230 cm^{-1}	45.054254 meV
6 f	10.142644 THz	63.728109 2PiTHz	338.322175 cm^{-1}	41.946585 meV
7 f	9.201811 THz	57.816682 2PiTHz	306.939367 cm^{-1}	38.055614 meV
8 f	8.203018 THz	51.541085 2PiTHz	273.623241 cm^{-1}	33.924943 meV
9 f	7.463638 THz	46.895418 2PiTHz	248.960154 cm^{-1}	30.867111 meV
10 f	5.787387 THz	36.363224 2PiTHz	193.046445 cm^{-1}	23.934698 meV
11 f	5.702479 THz	35.829732 2PiTHz	190.214225 cm^{-1}	23.583548 meV
**12 f/i=**	**11.818344 THz**	**74.256848 2PiTHz**	**394.217535 cm^{-1}**	**48.876723 meV**

Supplementary references

1 Fan, K. et al. Direct observation of structural evolution of metal chalcogenide in electrocatalytic water oxidation. *ACS Nano* **12**, 12369-12379 (2018).

2 Wang, X. et al. Pivotal role of reversible NiO_6 geometric conversion in oxygen evolution. *Nature* **611**, 702-708 (2022).

3 Zhang, X. et al. High-spin Co^{3+} in cobalt oxyhydroxide for efficient water oxidation. *Nat. Commun.* **15**, 1383 (2024).

4 Cibian, M., Derossi, S. & Hanan, G. S. Synthesis and crystal structure of a rare square-planar Co(II) complex of a hydroxyamidinate ligand. *Dalton. Trans.* **40**, 1038-1040 (2011).

5 Mathew, K. et al. Implicit self-consistent electrolyte model in plane-wave density-functional theory. *J. Chem. Phys.* **151**, 234101 (2019).

6 Cui, Q. Combining implicit solvation models with hybrid quantum mechanical/molecular mechanical methods: A critical test with glycine. *J. Chem. Phys.* **117**, 4720-4728 (2022).

7 Lebègue, E. Electrochemical methods: fundamentals and applications. *Transit. Met. Chem.* **48**, 433-436 (2023).

8 Bo, S. W. et al. Inorganic-organic hybrid cobalt spinel oxides for catalyzing the oxygen evolution reaction. *Nat. Commun.* **16**, 2483 (2025).

9 Zhao, Y. G. et al. Operando monitoring of the functional role of tetrahedral cobalt centers for the oxygen evolution reaction. *Nat. Commun.* **16**, 580 (2025).

10 Chang, X.-W. et al. Tuning morphology and electronic structure of cobalt metaphosphate via vanadium-doping for efficient water and urea splitting. *Adv. Funct. Mater.* **34**, 2313974 (2024).

11 Mariappan, A. et al. Novel heterostructure-based CoFe and cobalt oxysulfide nanocubes for effective bifunctional electrocatalytic water and urea oxidation. *Small* **20**, e2310112 (2024).

12 Kumar, P. et al. High-density cobalt single-atom catalysts for enhanced oxygen evolution reaction. *J. Am. Chem. Soc.* **145**, 8052-8063 (2023).

13 Zhang, R. R. et al. Tracking the role of defect types in Co_3O_4 structural evolution and active motifs during oxygen evolution reaction. *J. Am. Chem. Soc.* **145**, 2271-2281 (2023).

14 Jia, H. N. et al. Unveiling the electrolyte cations dependent kinetics on CoOOH-catalyzed oxygen evolution reaction. *Angew. Chem. Int. Ed.* **62**, e202313886 (2023).

15 Yuan, C.-Z. et al. In situ immobilizing atomically dispersed Ru on oxygen-defective Co_3O_4 for efficient oxygen evolution. *ACS Catal.* **13**, 2462-2471 (2023).

16 Naik, S. S. et al. Dual-cation-coordinated CoFe-layered double-hydroxide nanosheets using the pulsed laser ablation technique for efficient electrochemical water splitting: mechanistic screening by in situ/operando raman and density functional theory calculations. *ACS Catal.* **13**, 1477-1491 (2023).

17 Lu, M. et al. Artificially steering electrocatalytic oxygen evolution reaction mechanism by regulating oxygen defect contents in perovskites. *Sci. Adv.* **8**, eabq3563 (2022).

18 Wang, X. et al. Activating the lattice oxygen oxidation mechanism in amorphous molybdenum cobalt oxide nanosheets for water oxidation. *J. Mater. Chem. A* **10**, 3659-3666 (2022).

19 Cai, C. et al. Ultrahigh oxygen evolution reaction activity in Au doped co-based nanosheets. *RSC Adv.* **12**, 6205-6213 (2022).

20 Yao, N. et al. Intermolecular energy gap-induced formation of high-valent cobalt species in CoOOH surface layer on cobalt sulfides for efficient water oxidation. *Angew. Chem. Int. Ed.* **61**, e202117178 (2022).

21 Wang, S. H. et al. Identifying the geometric catalytic active sites of crystalline cobalt oxyhydroxides for oxygen evolution reaction. *Nat. Commun.* **13**, 6650 (2022).

22 Lin, X. et al. 5f covalency synergistically boosting oxygen evolution of $UCoO_4$ catalyst. *J. Am. Chem. Soc.* **144**, 416-423 (2022).

23 Wang, T. T. et al. Nanoframes of Co_3O_4–Mo_2N heterointerfaces enable high-performance bifunctionality toward both electrocatalytic HER and OER. *Adv. Funct. Mater.* **32**, 2108382 (2021).

24 Xiang, W. K. et al. 3D atomic-scale imaging of mixed Co-Fe spinel oxide nanoparticles during oxygen evolution reaction. *Nat. Commun.* **13**, 179 (2022).

25 Shah, K. et al. Cobalt single atom incorporated in ruthenium oxide sphere: a robust bifunctional electrocatalyst for HER and OER. *Angew. Chem. Int. Ed.* **61**, e202114951

(2022).

26 Wu, T. Z. et al. Spin pinning effect to reconstructed oxyhydroxide layer on ferromagnetic oxides for enhanced water oxidation. *Nat. Commun.* **12**, 3634 (2021).

27 Wang, J. et al. Redirecting dynamic surface restructuring of a layered transition metal oxide catalyst for superior water oxidation. *Nat. Catal.* **4**, 212-222 (2021).

28 Bai, L. C. et al. Double-atom catalysts as a molecular platform for heterogeneous oxygen evolution electrocatalysis. *Nat. Energy* **6**, 1054-1066 (2021).

29 Li, Z. J. et al. Tuning the spin density of cobalt single-atom catalysts for efficient oxygen evolution. *ACS Nano* **15**, 7105-7113 (2021).

30 Zhang, S. L. et al. Metal atom-doped Co_3O_4 hierarchical nanoplates for electrocatalytic oxygen evolution. *Adv. Mater.* **32**, e2002235 (2020).

31 Li, J. et al. Boosted oxygen evolution reactivity by igniting double exchange interaction in spinel oxides. *J. Am. Chem. Soc.* **142**, 50-54 (2020).

32 Pan, Y. L. et al. Direct evidence of boosted oxygen evolution over perovskite by enhanced lattice oxygen participation. *Nat. Commun.* **11**, 2002 (2020).

33 Dou, Y. H. et al. Approaching the activity limit of $CoSe_2$ for oxygen evolution via Fe doping and Co vacancy. *Nat. Commun.* **11**, 1664 (2020).

34 Huang, Z.-F. et al. Chemical and structural origin of lattice oxygen oxidation in Co–Zn oxyhydroxide oxygen evolution electrocatalysts. *Nat. Energy* **4**, 329-338 (2019).
