# Supplementary material for: Light-triggered oxygen redox activity at the edge of cobalt oxyhydroxide for superior water oxidation
Source: Nat Commun. 2026 Jun 11;17:7417. doi: 10.1038/s41467-026-74386-1 (PMC13408805; doi:10.1038/s41467-026-74386-1)
Supplement: Supplementary file 2 — Description of Additional Supplementary Files [file 41467_2026_74386_MOESM2_ESM.pdf]

Description of Additional Supplementary Files

File Name: Supplementary Data 1

Description: CONTCAR of the optimized R-CoOOH and S-CoOOH models.
