# Supplementary material for: Light-triggered oxygen redox activity at the edge of cobalt oxyhydroxide for superior water oxidation
Source: Nat Commun. 2026 Jun 11;17:7417. doi: 10.1038/s41467-026-74386-1 (PMC13408805; doi:10.1038/s41467-026-74386-1)
Supplement: Supplementary file 4 — Transparent Peer Review file [file 41467_2026_74386_MOESM4_ESM.pdf]

nature portfolio

Peer Review File

Light Triggered Oxygen Redox Activity at the Edge of Cobalt Oxyhydroxide for Superior Water Oxidation

Corresponding Author: Professor Junmin Xue

Parts of this Peer Review File have been redacted as indicated to remove third-party material.

This file contains all reviewer reports in order by version, followed by all author rebuttals in order by version.

Attachments originally included by the reviewers as part of their assessment can be found at the end of this file.

Version 0:

Reviewer comments:

Reviewer #1

(Remarks to the Author)
In this work, the authors disclose the contribution of light irradiation onto triggering a higher oxygen redox activity for water oxidation. Too many fundamental physical problems still be clarified as follows:
(1)Why the light can trigger an enhancement of catalytic activity? If the light-matter interaction can drive a structure transformation, what is the physical essential reason, structural phase transformation or thermal effect, or addition reaction? How to confirm this? How about removing the electrolyte?
(2)In all the experimental characterizations, we cannot find the reversible behavior of light irradiations. How to exclude the reconstruction process of photothermal effect or electrochemical-coupling behavior? If that's really the case, the actual contribution of light irradiation should be reconsidered.
(3)In fact, the light-matter interaction has not be discussed, such as how to separate electron-hole pair? how to affect the atomic reconfiguration? the relationship between wave length and structure response? What is the role of translational symmetry and time-reversal symmetry (or breaking) in light response? (see Eric V.Ansly nand Dennis A. Dougherty, Modern Physical Organic Chemistry)
(4)In addition, reconstruction process of light irradiations cannot be simple confirmed by changes in XAS. What is the atomic coordination number? How to affect the 3d orbital by light? How to understand interaction between boson (light) and fermion (electron)?
(5)In fact, several hours light irradiations may lead to a raid thermal enhancement, whether or not the high-temperature-induced electrochemical reconfiguration is the actual reason?
(6)If the contribution of light irradiations is the key factor, the catalytic activity will be significantly enhanced by regulating their light-matter interactions. However, this behavior cannot be observed or provided.

Reviewer #2

(Remarks to the Author)
Please see attachment.

Reviewer #3

(Remarks to the Author)
Zhang's study reported that the light irradiation triggers ligand-to-metal charge transfer in S-CoOOH catalyst, leading to formation of ONB states. The neighboring ONB states enable direct O-O coupling, namely coupled oxygen evolution mechanism, which achieves superior OER activity compared to the same catalysts under dark conditions, as well as R-CoOOH with weaker ligand-to-metal charge transfer properties. In my opinion, some experimental aspects of it are appealing, but significant concerns remain for the model construction, DFT computational details, and mechanistic discussion. These limitations compromise the study's conclusions. Therefore, I cannot recommend its publication in Nature Communications. Specific issues include:

1. Line 459, the authors claimed that the total of electrons in S-CoOOH supercell was adjusted to modify the oxidation state, please supplement the details and state the oxidation state of Co sites in optimized model.
2. Line 222, the authors claimed that S-CoOOH model feature 4-coordinated Co at edge and 6-coordinated Co in the bulk. Please highlight the locations of 4 and 6-coordinated Co in Figure S21.
3. The structural model in Figure S21 is hard to read. It seems the authors introduced several random protons on surface to model the S-CoOOH catalyst, right? Please supplement which oxygen atoms were terminated by protons, and why this.
4. All the calculations seem to be conducted in vacuum conditions. It is well established that the solvent environment plays an important role in electronic catalysis, especially considering there are many proton transfer steps in OER process. The authors are encouraged to consider the solvent conditions or at least need to rule out the effect on DFT conclusions.
5. Line 455. CI-NEB method was used to locate the transition state of O-O coupling. Were the transition states confirmed by imaginary vibrational frequency analysis?
6. Figure 5c. The authors performed density of state analysis for R-CoOOH catalyst. But I cannot find the descriptions for constructing this model.
7. Figure 4b only considered the O-O coupling step at edge or bulk sites. But in Line 250, the authors claimed that the *OOH (i.e., O2- adsorbs OH-) formation is the rate-determining step. The effect of edge and bulk O sites on other OER elementary steps, such as OH- adsorption, O release, need to be considered. Moreover, the calculations seem to be on an assumption that the OER on ONB sites is more favorable than that on metal sites. To clarify this, the authors need to perform DFT calculations about OER reaction on cobalt sites of S-CoOOH model.

Version 1:

Reviewer comments:

Reviewer #1

(Remarks to the Author)
Some problems have been addressed, but many points still need to be further clarified.
(1)In fact, the current density at many catalyst materials can be enhanced, which is completely not related with their reaction kinetics of OER. This may be induced by the photocurrent, because this light irradiation may directly enhance the conductivity of your catalysts without any involvements of electrochemical reaction when applying a special voltage. By the way, what is your working cell voltage?
(2)Why the charge transfer from oxygen to cobalt can occurs? Just light irradiation? What is the driving force? Transient absorption spectra should be provided to unveil this important process of light-induced carrier kinetic.
(3)The light adsorption response of different wavelength is directly related with their band gap, so the enhancement in OER current can be observed but it cannot directly confirm their intrinsic correlations to change the reaction pathway (or activity) of OER.
(4) The influence of light carrier onto OER reaction pathway should be carefully confirmed, involving DFT calculations and electrochemical experiments.
(5)The thermodynamics and kinetic processes should be simultaneously considered in your DFT calculations, especially for the contribution of electrolyte or adsorbates.
(6)In addition, if the reaction pathway of OER can be changed by the light irradiations, the adsorption configuration may be reflected by their changes in entropy or enthalpy values.
(7)How about the lifetime (ps or ns) of your light-induced carriers and OER reaction (ms)? How to couple together? Why they can occur? What is their effective distance of charge transfer? In a word, time-dependent spectral analysis and fundamental physical modes should be established.

Reviewer #2

(Remarks to the Author)
Overall, most of the reviewer’s questions have been addressed. However, two additional points should be clarified.
First, regarding the photophysical properties of SCoOOH, reflectance measurements would help clearly demonstrate the light absorption of S-CoOOH, and the details of the UV/Vis measurement conditions should be specified.
Second, the analysis of structural reconstruction of oxyhydroxides after the OER appears insufficient. Adding XRD analysis is recommended to strengthen this part.

Reviewer #3

(Remarks to the Author)
This manuscript presents a systematic study on the photo-assisted oxygen evolution reaction (OER) of cobalt oxyhydroxide, combining operando spectroscopic experiments with density functional theory (DFT) analysis. The work is overall comprehensive, and the integration of theory and experiment is commendable. However, several key aspects of the proposed mechanism and methodology remain insufficiently supported.

1. The authors attribute enhanced catalytic performance under illumination to a photo-induced LMCT process. However, the supporting evidence mainly relies on spectral intensity variations in XAS and photocurrent enhancement, both of which are indirect indicators. Such observations could also result from light-induced charge redistribution and electrochemical potential shifts. The manuscript would be significantly strengthened if the authors could provide direct experimental

evidence for LMCT, such as transient absorption spectroscopy or photoelectron spectroscopy data.

2. Although the authors state that illumination increases the reaction temperature by less than 2 °C, this measurement alone cannot conclusively exclude the influence of photothermal effects. Nanoscale "hot spots" or localized heating in the catalyst layer could alter reaction kinetics or electronic states even under seemingly mild macroscopic temperature changes. Furthermore, localized light absorption could induce structural strain or interfacial polarization, both of which can mimic photoelectronic enhancement. The method used for temperature monitoring is inadequate to assess microscale temperature gradients. Therefore, additional characterization such as photothermal IR mapping et.al would help to verify whether illumination triggers genuine electronic effects rather than subtle thermal or structural perturbations.

3. The theoretical calculations in the manuscript are presented as evidence supporting the experimentally observed light effects; however, they appear to be limited to ground-state DFT without explicit treatment of the photoexcited electronic structure. In this form, the calculations primarily reveal static charge distributions and adsorption energetics, which cannot directly account for transient electron transfer induced by illumination. To justify the link between LMCT and catalytic enhancement, the authors should explain how the computed charge density differences and reaction barriers reflect photoinduced electron migration.

4. In response to the first reviewer's first question, the authors claim that operando XAS confirms the structural stability of the Co oxyhydroxide catalyst under illumination. While this technique provides valuable information on the local coordination environment, it cannot entirely rule out surface amorphization or subtle lattice reconstruction, especially in the near-surface region where catalysis occurs. The observed similarity in average Co-O coordination number and bond length does not necessarily imply that the surface remains intact. It would be helpful if the authors could include or reference complementary structural characterizations such as post-reaction TEM, operando Raman, or XRD measurements to demonstrate that no phase transformation or surface reorganization accompanies the photo assisted process. Without such verification, it remains possible that part of the observed activity change arises from illumination-induced structural dynamics rather than pure electronic effects.

5. In response to the fourth question raised by the third reviewer, the authors repeatedly emphasize that CI-NEB and DOS analyses were performed based on first-principles calculations; however, all computations were conducted in vacuum, and the structural models employed are not fully consistent with those used in previous studies. The justification that "the differences from earlier results are negligible" is therefore unconvincing. For the oxygen evolution reaction (OER) system, solvent polarization and interfacial electric fields play a crucial role in stabilizing intermediates and influencing the O-O coupling barrier. Calculations performed under vacuum conditions may overestimate the energy barriers or misrepresent the reaction mechanism. It is thus recommended that the authors at least include an implicit solvation model (e.g., VASPsol) or apply surface charge corrections to validate their results and improve the physical reliability and comparability of the conclusions.

Version 2:

Reviewer comments:

Reviewer #1

(Remarks to the Author)
Most of problems have been addressed, but some points still need to be clarified.
(1) Based on your responses, the PEIS for S-CoOOH display similar results in dark and under light irradiation. It is difficult to understand this point. The adsorbate configuration at catalyst surface should be changed by light-driven transformation, why the PEIS values are so such alike?
(2) In addition, how to implant the light irradiations into the industrial electrolyzer?
(3) In the DFT calculation, the contribution of pH values should be considered. The U values should be reconsidered to reevaluate their rate-limiting steps.

Reviewer #2

(Remarks to the Author)
publish as is

Reviewer #3

(Remarks to the Author)
Authors have well readdressed the comments raised by the reviewers, now this manuscript can be accepted.

Version 3:

Reviewer comments:

Reviewer #1

(Remarks to the Author)
Great, can be accepted as it is

Open Access This Peer Review File is licensed under a Creative Commons Attribution 4.0 International License, which permits use, sharing, adaptation, distribution and reproduction in any medium or format, as long as you give appropriate credit to the original author(s) and the source, provide a link to the Creative Commons license, and indicate if changes were made.
In cases where reviewers are anonymous, credit should be given to 'Anonymous Referee' and the source.
The images or other third party material in this Peer Review File are included in the article's Creative Commons license, unless indicated otherwise in a credit line to the material. If material is not included in the article's Creative Commons license and your intended use is not permitted by statutory regulation or exceeds the permitted use, you will need to obtain permission directly from the copyright holder.
To view a copy of this license, visit https://creativecommons.org/licenses/by/4.0/

Response to referees

We thank the three referees for taking the time to carefully review the manuscript and for giving many useful suggestions. The manuscript has been revised according to their comments and the changes have been highlighted in the revised version. We feel the quality of the paper has been improved greatly thanks to the input from the referees. Below is a point-by-point response.

Reviewer #1

Remarks to the Author: In this work, the authors disclose the contribution of light irradiation onto triggering a higher oxygen redox activity for water oxidation. Too many fundamental physical problems still be clarified as follows:

1. Why the light can trigger an enhancement of catalytic activity? If the light-matter interaction can drive a structure transformation, what is the physical essential reason, structural phase transformation or thermal effect, or addition reaction? How to confirm this? How about removing the electrolyte?

Response: We are very grateful for the referee's comment. In our work, the mechanism of light triggering an enhancement of catalytic activity could be described as follows: light irradiation triggers electron transfer from oxygen (ligand) to cobalt (metal) in S-CoOOH, leading to the formation of non-bonding oxygen (O_{NB}) states; then, two neighboring O_{NB} states spontaneously couple to form direct O-O coupling, thereby bypassing the rate-determining step of *OOH formation in dark metal-redox pathway. **As such, light shifts the redox activity from metal to oxygen, which is the reason for the enhancement of catalytic activity.**

Meanwhile, the light-matter interaction does not induce structural transformation. It only triggers the electron transfer from oxygen (ligand) to cobalt (metal) in S-CoOOH, resulting in direct O-O coupling. This could be verified by *operando* X-ray absorption fine structure (XAFS) results, showing that during the light illumination process, S-CoOOH preserves its intact octahedral structure without any phase transformation (Fig.R1-1).

The possibility of other factors, such as thermal effect, has already been discussed in our Supplementary

Information. In our work, to avoid the thermal effect, electrochemical measurements were performed with a cell connected to a thermostatic bath. As stated in Fig.S1 (lines 1-4 in previous Supplementary Information): An electrochemical cell connected to a thermostatic bath was used for electrochemical testing under light illumination, whereby cooling water was used to keep the temperature of the system constant. The surface temperature of catalysts was monitored by a thermocouple and was found to increase by less than 2 ºC throughout the whole measurement process. Besides, there is no additional reaction under light illumination, as the light enhanced OER activity is fully reversible (Fig.R1-2). Instead, light illumination only induces electron transfer from oxygen to cobalt.

As discussed above, in fact the light-matter interaction was one of the parts of OER process. In other words, the light-matter interaction could only occur during OER process. **As such, removing the electrolyte is not feasible.**

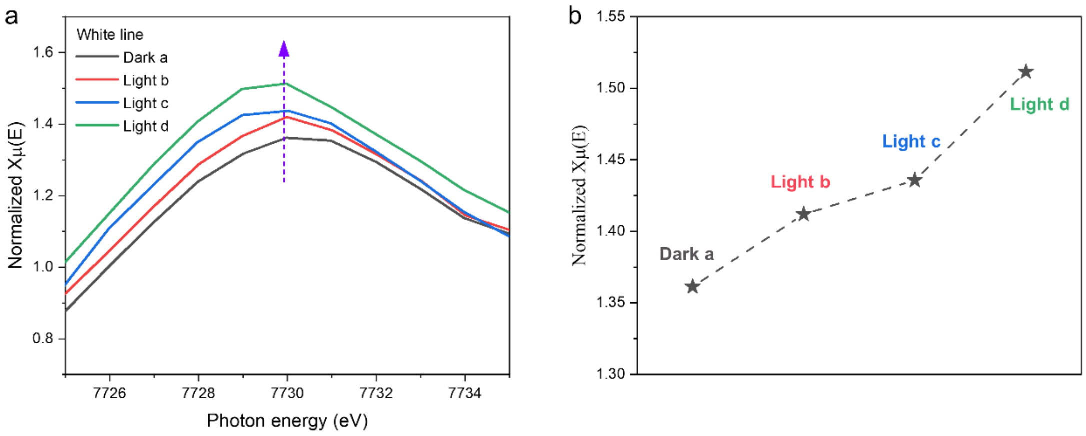

Figure R1-1. a Enlarged *operando* Co *K*-edge XAS spectra in white line region from 7725 to 7735 eV, extracted from Fig.2b **b** White line intensity of S-CoOOH subjected to light, recorded during OER from dark to illumination at four selected points: Dark a, Light b, Light c, and Light d. (Supplementary Fig.S14 in our previous Supplementary Information, Supplementary Fig.S21 in our revised Supplementary Information)

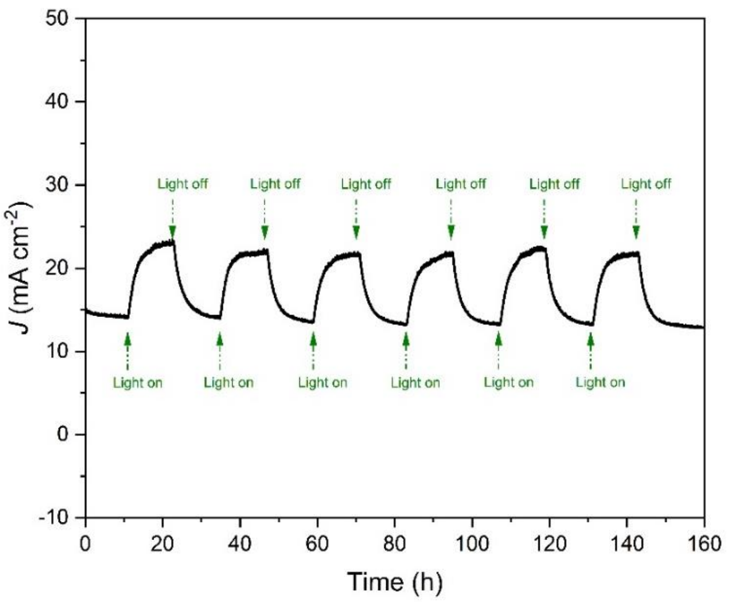

Fig.R1-2. The repeatability experiments for electrochemical measurements of S-CoOOH. (Supplementary Fig.S1 in our previous Supplementary Information, Supplementary Fig.S3 in our revised version)

To address the comment, we have added a paragraph into our manuscript describing the mechanism of light-triggered catalytic enhancement (lines 269-274, page 11, highlighted in yellow): Briefly, the mechanism of light-triggered catalytic enhancement in S-CoOOH is illustrated in Fig.4c,d. Light irradiation triggers electron transfer from oxygen (ligand) to cobalt (metal) in S-CoOOH, leading to the formation of O_{NB} states; then, two neighboring O_{NB} states spontaneously couple to form direct O-O coupling, thereby bypassing the rate-determining step of *OOH formation in dark metal-redox pathway. As such, light shifts the redox activity from metal to oxygen, which is the reason for the enhancement of catalytic activity.

Also, we have revised the relevant content in our manuscript, to emphasize that no phase transformation takes place (lines 146-149, page 6, highlighted in yellow): Moreover, *operando* XAS result for S-CoOOH demonstrates intensification of the while line with prolonged light irradiation (Fig.2b), suggesting a more regular CoO_6 octahedral structure under light without any phase transformation.

Besides, we have incorporated the proposed modifications into our manuscript (lines 108-111, page 4, highlighted in yellow): Initially, several potential reasons for the distinctive increase in OER performance under light illumination are excluded, including thermal effect, changes in ohmic resistance and grain size, generation of light-induced holes, or any additional reaction (Supplementary Figs.S9-S15).

2. In all the experimental characterizations, we cannot find the reversible behavior of light irradiations. How to exclude the reconstruction process of photothermal effect or electrochemical-coupling behavior? If that's really the case, the actual contribution of light irradiation should be reconsidered.

Response: We are very grateful for the referee's comment. In our previous manuscript (lines 94-96, page 4), we had included the following description: The repeatability of light-induced behavior in S-CoOOH is verified in Supplementary Fig.S1, demonstrating a prolonged operating duration of six consecutive on/off cycles spanning about 160 hours. In our previous Supplementary Information (Supplementary Fig.S1), we had included the reversible behavior of light irradiation, and for clarity this figure is also provided here as Fig.R1-3. Moreover, the following explanation was given below Fig.S1: The result indicates the long-term repeatability of S-CoOOH over six light turn-on/off cycles. The current after 160 hours of illumination cycling remains nearly identical to the initial value, indicating excellent operational stability under light irradiation. This clearly indicates that the light-enhanced OER activity is reversible behavior, rather than an irreversible process caused by reconstruction. As such, the possibility of reconstruction could be excluded.

Furthermore, photothermal effect was ruled out by controlling the catalyst surface temperature via connecting to a thermostatic bath. Besides, our work does not involve electrochemical-coupling behavior. Once the light is switched on, the current exhibits an immediate increase followed by a stabilization period; when light is turned off, the current fully returns to its initial baseline, indicating that the light-induced enhancement is completely reversible. Here, the role of light is solely to generate non-bonding oxygen (O_{NB}) states at the edge of S-CoOOH, via light-induced electron transfer from oxygen to cobalt. These O_{NB} states subsequently shift the electrochemical OER mechanism from metal to oxygen redox.

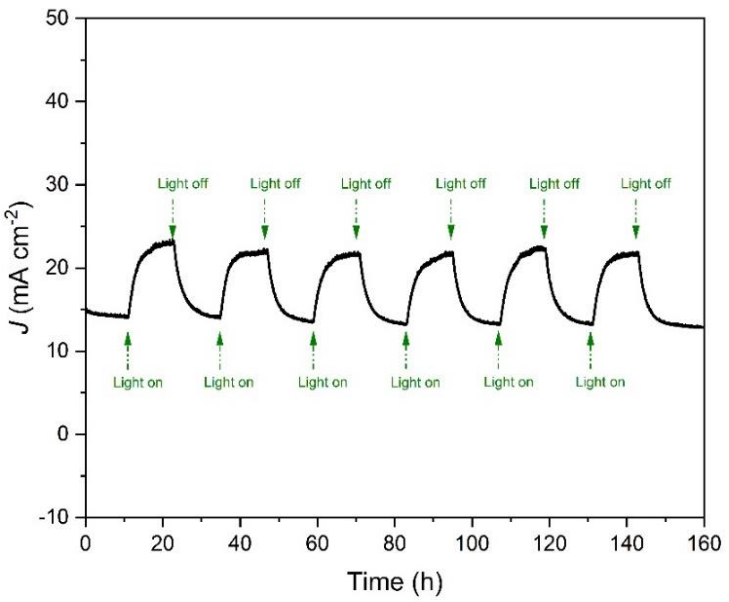

Fig.R1-3. The repeatability experiments for electrochemical measurements of S-CoOOH. (Supplementary Fig.S1 in our previous Supplementary Information, Supplementary Fig.S3 in our revised Supplementary Information)

To address the comment, we have incorporated the proposed modifications into our manuscript (lines 97-100, page 4, highlighted in yellow): Furthermore, the repeatability of light-induced behavior in S-CoOOH is verified in Supplementary Fig.S3, demonstrating a prolonged operating duration of six consecutive on/off cycles spanning about 160 hours. This long-term repeatability excludes the possibility of structural reconstruction.

Also, we have revised the content accompanying Supplementary Fig.S3 (previously Fig.S1) in our supplementary information: Supplementary Fig.S3 indicates the long-term repeatability of S-CoOOH over six light turn-on/off cycles, maintaining stability over 160 hours. The current after 160 hours of illumination cycling remains nearly identical to the initial value, indicating excellent operational stability under light irradiation. This clearly indicates that the light-enhanced OER activity is reversible behavior, rather than an irreversible process caused by reconstruction. As such, the possibility of reconstruction could be excluded.

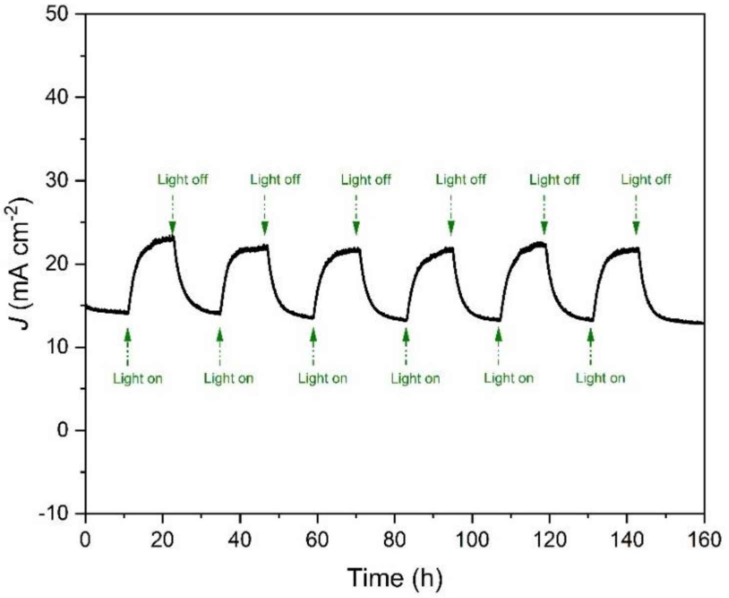

Supplementary Fig.S3. The repeatability experiments for electrochemical measurements of S-CoOOH. (Supplementary Fig.S1 in our previous Supplementary Information, Supplementary Fig.S3 in our revised Supplementary Information)

3. In fact, the light-matter interaction has not be discussed, such as how to separate electron-hole pair? how to affect the atomic reconfiguration? the relationship between wave length and structure response? What is the role of translational symmetry and time-reversal symmetry (or breaking) in light response? (see Eric V.Ansly nand Dennis A. Dougherty, Modern Physical Organic Chemistry)

Response: We are very grateful for the referee's comment. Discussion on the light-matter interaction is provided below. Briefly, light irradiation generates electron-hole pairs through electron transfer from oxygen to cobalt. The generated holes were the non-bonding oxygen species, which were then stabilized via direct O-O coupling with adjacent ones. Through this way, the electron-hole pair was separated.

Based on the referee's insightful advice, we have fitted the atomic coordination numbers (CN) of Co-O and Co-Co using the *operando* Co *K*-edge Fourier transformed extended X-ray near fine structure (FT-EXAFS) spectra of S-CoOOH (Fig.R1-4), collected during OER under light irradiation (Dark a, Light b, Light c, and Light d). As shown in Table R1-1, the CN of Co-O and Co-Co for S-CoOOH increase progressively with prolonged light irradiation, and this atomic configuration change is reversible.

In our photo response measurements under varying illumination wavelengths, the current enhancement is found to be strongly related to the incident light wavelength (Fig.R1-5). Illumination at 575 nm and 800 nm result in a pronounced increase in current. Meanwhile, the calculated energy barrier in S-CoOOH corresponds to about 1260 nm (0.98 eV), the response at longer wavelengths is expected to diminish. As shown in Fig.R1-5c and d, at 1200 nm (1.03 eV) the current increase is barely detectable, indicating only minimal LMCT activation, while illumination at 1310 nm (0.95 eV) produces no observable effect, which agrees well with our calculation results. These findings confirm that light-induced electron transfer in S-CoOOH is strongly governed by the illumination wavelength.

Moreover, as shown in *operando* Co *K*-edge XAS spectra, under light irradiation, S-CoOOH only shows an increased white-line intensity (Fig.R1-6a), confirming that the octahedral structure of S-CoOOH remains intact under light irradiation. It indicates that during light irradiation the translation symmetry is preserved without any structural reconstruction. Besides, the complete reversibility of the light-induced enhancement (Fig.R1-6b) demonstrates that the modulation of the electronic structure by light is fully reversible, indicating that no time-reversal symmetry breaking occurs under light irradiation. Hence, our work does not involve any change in translational or time-reversal symmetry.

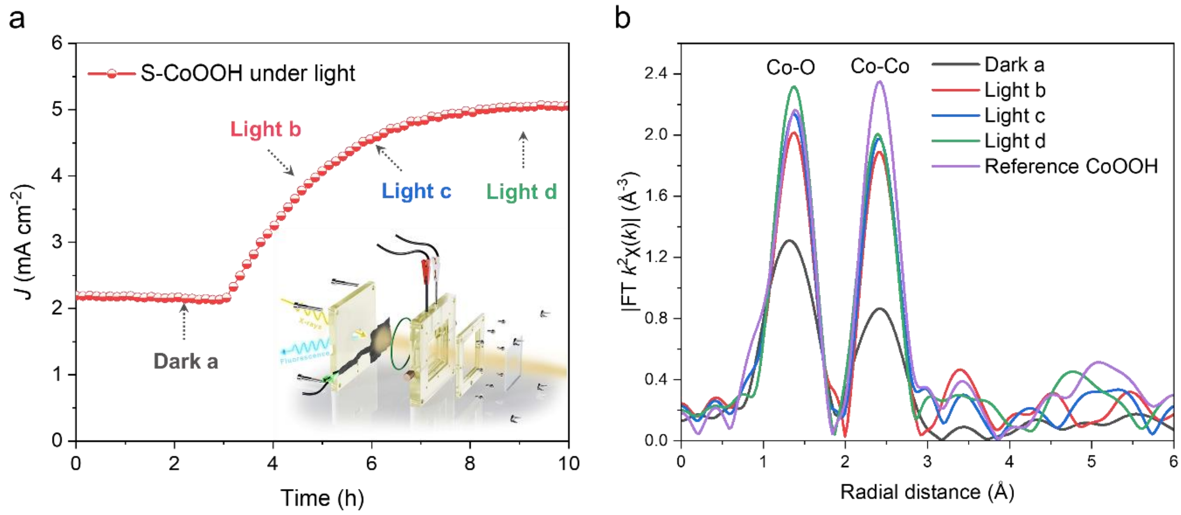

Fig.R1-4. a Electrochemical curve for operando Co K-edge XAS measurements recorded during OER from dark to illumination at four selected points: Dark a, Light b, Light c, and Light d. **b** Normalized Co *K*-edge Fourier transformed extended X-ray near fine structure (FT-EXAFS) spectra, collected during OER under light irradiation (Dark a, Light b, Light c, and Light d), with Reference CoOOH used as the standard. (Fig.2d and Supplementary Fig.S22 in our manuscript and Supplementary Information)

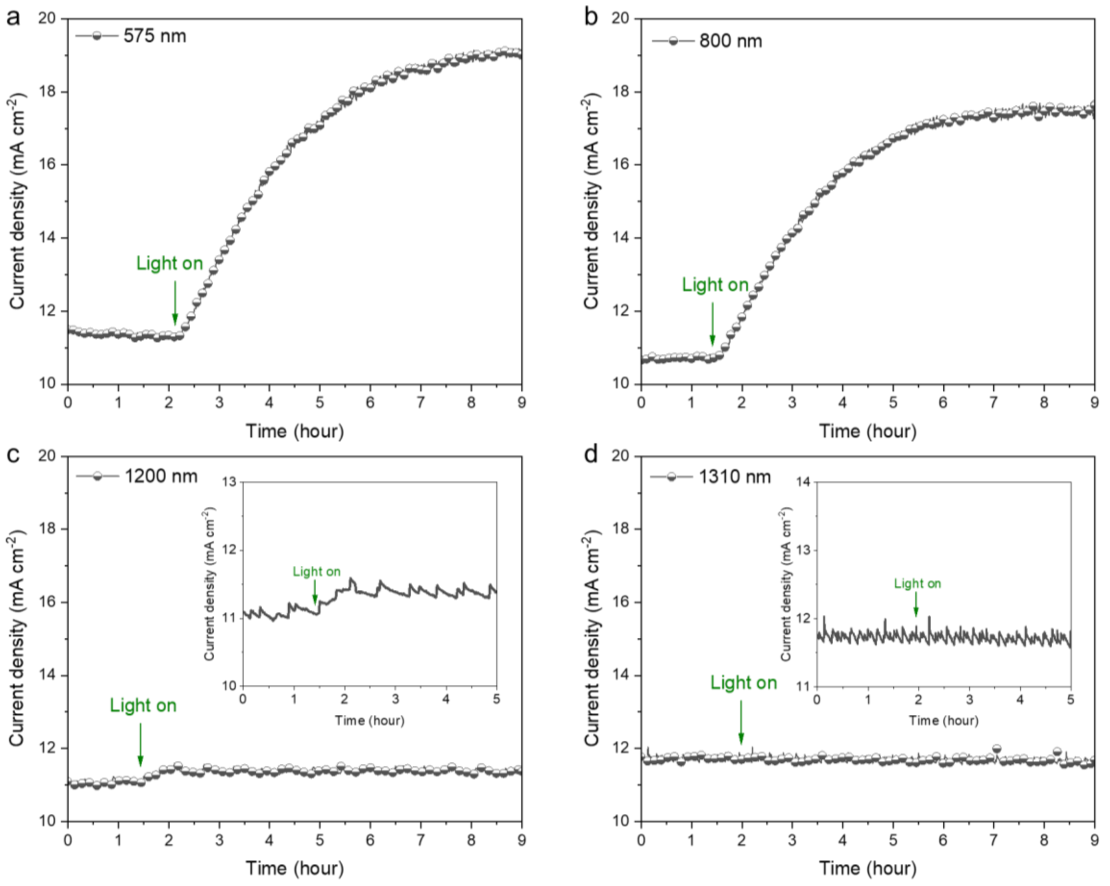

Fig.R1-5. Photon response measurements equipped with different wavelength filters of 575 nm (**a**), 800 nm (**b**), 1200 nm (**c**), and 1310 nm (**d**), respectively. (Supplementary Fig.S8 in our Supplementary Information)

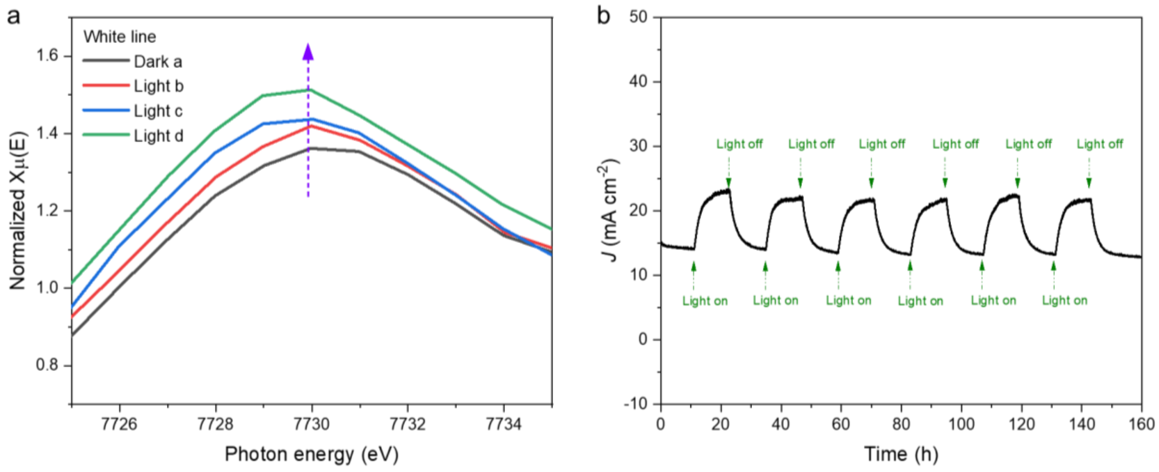

Fig.R1-6. a Enlarged *operando* Co *K*-edge XAS spectra in white line region from 7725 to 7735 eV, extracted from **Fig.2b** **b** The repeatability experiments for electrochemical measurements of S-CoOOH. (Supplementary Fig.S14a and Fig.S1 in our previous Supplementary Information, Supplementary Fig.S21a and Fig.S3 in our revised Supplementary Information)

Table R1-1. FT-EXAFS fitting results of S-CoOOH collected during OER under light irradiation (Dark a, Light b, Light c, and Light d), with Reference CoOOH used as the standard. CN is the coordination number, σ^2 is the Debye-Waller factor, ΔE_0 is the shift in absorption edge energy, R is the bond length, and R-factor means Residual factor. (Supplementary Table S3 in our Supplementary Information)

Sample	path	CN	σ^2	ΔE_0	R	R-factor
Reference	Co-O	6	0.0023	-3.4	1.90±0.01	0.0055
CoOOH	Co-Co	6	0.0045	-4.5	2.85±0.01	
S-CoOOH	Co-O	4.97±0.48	0.0130	-6.7	1.87±0.01	0.0183
at Dark a	Co-Co	4.18±0.76	0.0170	3.9	2.91±0.04	
S-CoOOH	Co-O	5.78±0.58	0.0046	-2.2	1.89±0.02	0.0153
at Light b	Co-Co	4.43±0.55	0.0052	-2.0	2.85±0.02	
S-CoOOH	Co-O	5.90±0.42	0.0040	-3.5	1.88±0.01	0.0068
at Light c	Co-Co	4.83±0.42	0.0055	-1.4	2.84±0.02	
S-CoOOH	Co-O	5.97±0.50	0.0030	-4.3	1.88±0.02	0.0138
at Light d	Co-Co	5.16±0.57	0.0059	-3.7	2.83±0.02	

To address the comment, we have incorporated the following description into our manuscript (lines 274-278, page 11, highlighted in yellow): To further clarify the light-matter interaction, specifically the interaction between light and electron, Fig.4d depicts the underlying photoexcitation: Light irradiation generates electron-hole pairs through electron transfer from oxygen to cobalt. The generated holes were the non-bonding oxygen species, which have then stabilized via direct O-O coupling with adjacent one. Through this way, the electron-hole pair was separated.

We have also added the relevant content to our manuscript (lines 153-156, page 6, highlighted in yellow): The atomic coordination number (CN) of Co-O and Co-Co are fitted based on the *operando* Co *K*-edge Fourier transformed extended X-ray near fine structure (FT-EXAFS) spectra of S-CoOOH. As illustrated in Supplementary Fig.S22 and Table S3, it is revealed that the CN of Co-O and Co-Co for S-CoOOH increase progressively with prolonged light irradiation.

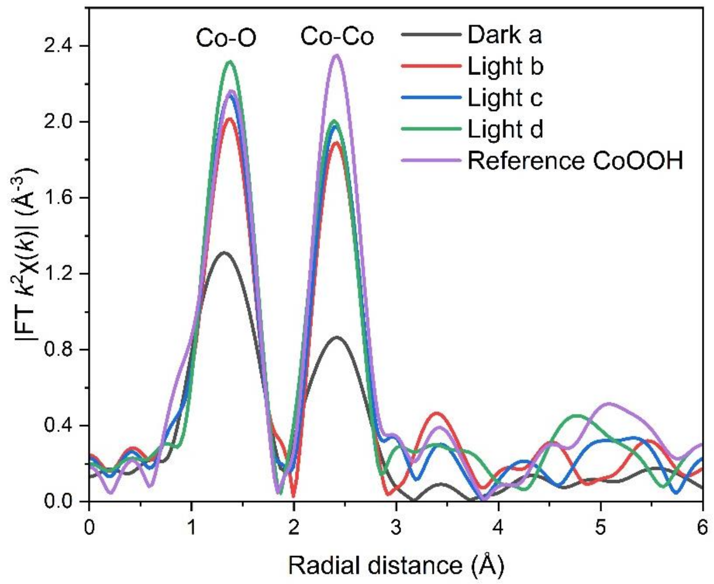

Supplementary Figure S22. Normalized Co *K*-edge Fourier transformed extended X-ray near fine structure (FT-EXAFS) spectra, collected during OER under light irradiation (Dark a, Light b, Light c, and Light d), with Reference CoOOH used as the standard.

Supplementary Table S3. FT-EXAFS fitting results of S-CoOOH collected during OER under light irradiation (Dark a, Light b, Light c, and Light d), with Reference CoOOH used as the standard. CN is the coordination number, σ^2 is the Debye-Waller factor, ΔE_0 is the shift in absorption edge energy, R is the bond length, and R-factor means Residual factor.

Sample	path	CN	σ^2	ΔE_0	R	R-factor
Reference	Co-O	6	0.0023	-3.4	1.90±0.01	0.0055
CoOOH	Co-Co	6	0.0045	-4.5	2.85±0.01	
S-CoOOH	Co-O	4.97±0.48	0.0130	-6.7	1.87±0.01	0.0183
at Dark a	Co-Co	4.18±0.76	0.0170	3.9	2.91±0.04	
S-CoOOH	Co-O	5.78±0.58	0.0046	-2.2	1.89±0.02	0.0153
at Light b	Co-Co	4.43±0.55	0.0052	-2.0	2.85±0.02	
S-CoOOH	Co-O	5.90±0.42	0.0040	-3.5	1.88±0.01	0.0068
at Light c	Co-Co	4.83±0.42	0.0055	-1.4	2.84±0.02	
S-CoOOH	Co-O	5.97±0.50	0.0030	-4.3	1.88±0.02	0.0138
at Light d	Co-Co	5.16±0.57	0.0059	-3.7	2.83±0.02	

Besides, we have incorporated the proposed modifications into our manuscript (lines 151-153, page 6, highlighted in yellow): The increased white-line intensity and reversibility indicate that there is no change in translational and time-reversal symmetry (Supplementary Fig.S21 and Fig.S3).

Also, we have implemented the suggested revisions into our manuscript (lines 107-108, page 4, highlighted in yellow): The photon response is related to the light intensity and wavelength (Supplementary Figs.S7-S8).

In addition, we have included a detailed explanation below Supplementary Fig.S21: Our work does not involve any change in translational or time-reversal symmetry. As shown in *operando* Co *K*-edge XAS spectra, under light irradiation, S-CoOOH only shows an increased white-line intensity (Supplementary Fig.S21), confirming that the octahedral structure of S-CoOOH remains intact under light irradiation. It indicates that during light irradiation the translation symmetry is preserved without any structural reconstruction. Besides, the complete reversibility of the light-induced enhancement (Supplementary Fig.S3) demonstrates that the modulation of the electronic structure by light is fully reversible, indicating that no time-reversal symmetry breaking occurs under light irradiation.

4. In addition, reconstruction process of light irradiations cannot be simple confirmed by changes in XAS. What is the atomic coordination number? How to affect the 3d orbital by light? How to understand interaction between boson (light) and fermion (electron)?

Response: We are very grateful for the referee's comment. According to the referee's insightful suggestion, the atomic coordination number (CN) of Co-O and Co-Co are fitted based on the *operando* Co *K*-edge FT-EXAFS spectra of S-CoOOH (Fig.R1-7), collected during OER under light irradiation (Dark a, Light b, Light c, and Light d). As shown in Table R1-2, it is revealed that the CN of Co-O and Co-Co for S-CoOOH increase progressively with expanded light irradiation.

According to *operando* XAS spectra, light irradiation induces a reduction in Co oxidation state, reflecting an increased occupancy in 3*d* orbital. Detailed discussion on the interaction between light and electron is provided below. Briefly, light irradiation triggers electron transfer from oxygen to cobalt, generating non-

bonding oxygen species and a decrease in Co valence. These unstable species spontaneously couple to form O-O bonds, thereby bypassing the rate-determining step *OOH formation and thus lowering the overpotential.

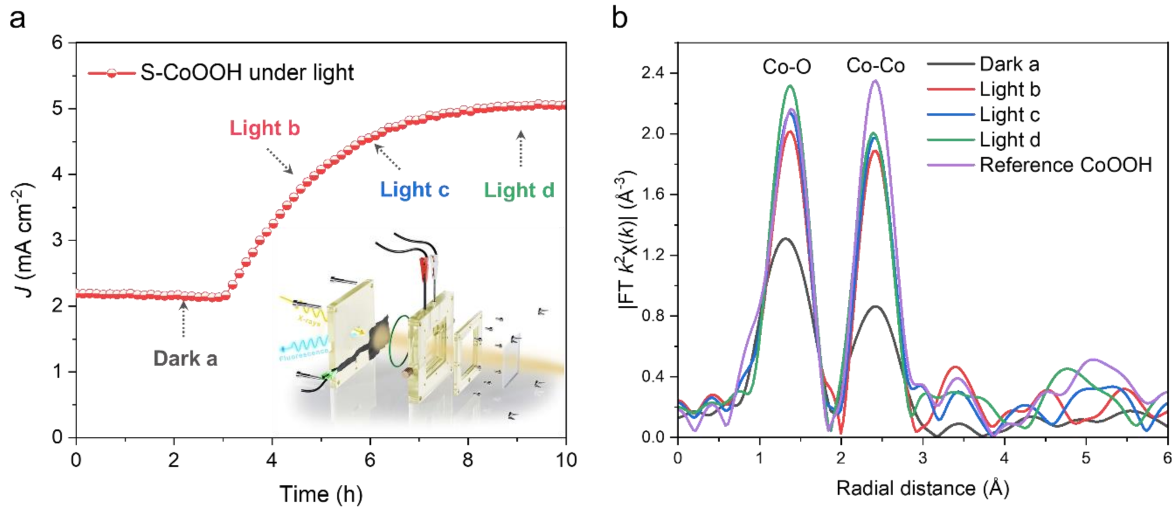

Fig.R1-7. a Electrochemical curve for operando Co K-edge XAS measurements recorded during OER from dark to illumination at four selected points: Dark a, Light b, Light c, and Light d. **b** Normalized Co *K*-edge Fourier transformed extended X-ray near fine structure (FT-EXAFS) spectra, collected during OER under light irradiation (Dark a, Light b, Light c, and Light d), with Reference CoOOH used as the standard. (Fig.2d and Supplementary Fig.S22 in our manuscript and Supplementary Information)

Table R1-2. FT-EXAFS fitting results of S-CoOOH collected during OER under light irradiation (Dark a, Light b, Light c, and Light d), with Reference CoOOH used as the standard. CN is the coordination number, σ^2 is the Debye-Waller factor, ΔE_0 is the shift in absorption edge energy, R is the bond length, and R-factor means Residual factor. (Supplementary Table S3 in our Supplementary Information)

Sample	path	CN	σ^2	ΔE_0	R	R-factor
Reference CoOOH	Co-O	6	0.0023	-3.4	1.90±0.01	0.0055
	Co-Co	6	0.0045	-4.5	2.85±0.01	
S-CoOOH at Dark a	Co-O	4.97±0.48	0.0130	-6.7	1.87±0.01	0.0183
	Co-Co	4.18±0.76	0.0170	3.9	2.91±0.04	
S-CoOOH at Light b	Co-O	5.78±0.58	0.0046	-2.2	1.89±0.02	0.0153
	Co-Co	4.43±0.55	0.0052	-2.0	2.85±0.02	

S-CoOOH at Light c	Co-O	5.90±0.42	0.0040	-3.5	1.88±0.01	0.0068
	Co-Co	4.83±0.42	0.0055	-1.4	2.84±0.02	
S-CoOOH at Light d	Co-O	5.97±0.50	0.0030	-4.3	1.88±0.02	0.0138
	Co-Co	5.16±0.57	0.0059	-3.7	2.83±0.02	

In response to the comment, we have incorporated the FT-EXAFS fitting results into our manuscript (lines 153-156, page 6, highlighted in yellow): The atomic coordination number (CN) of Co-O and Co-Co are fitted based on the *operando* Co *K*-edge Fourier transformed extended X-ray near fine structure (FT-EXAFS) spectra of S-CoOOH. As illustrated in Supplementary Fig.S22 and Table S3, it is revealed that the CN of Co-O and Co-Co for S-CoOOH increase progressively with prolonged light irradiation.

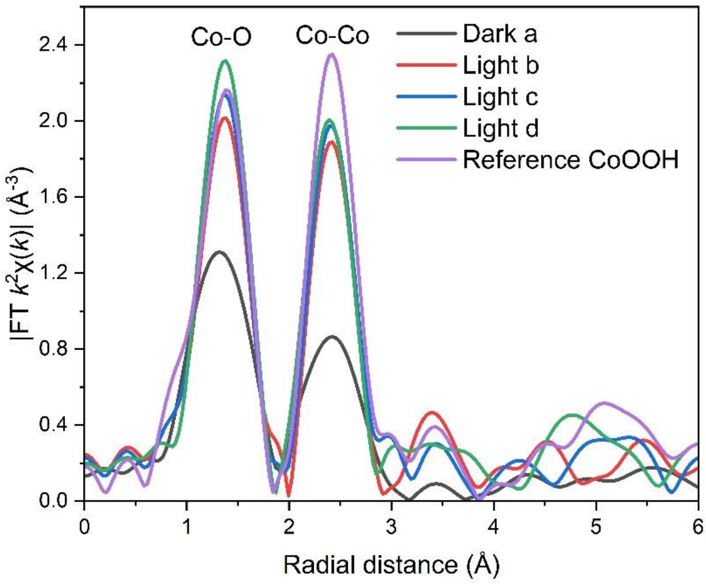

Supplementary Figure S22. Normalized Co *K*-edge Fourier transformed extended X-ray near fine structure (FT-EXAFS) spectra, collected during OER under light irradiation (Dark a, Light b, Light c, and Light d), with Reference CoOOH used as the standard.

Supplementary Table S3. FT-EXAFS fitting results of S-CoOOH collected during OER under light irradiation (Dark a, Light b, Light c, and Light d), with Reference CoOOH used as the standard. CN is the coordination number, σ^2 is the Debye-Waller factor, ΔE_0 is the shift in absorption edge energy, R is the bond length, and R-factor means Residual factor.

Sample	path	CN	σ^2	ΔE_0	R	R-factor
Reference	Co-O	6	0.0023	-3.4	1.90±0.01	0.0055
CoOOH	Co-Co	6	0.0045	-4.5	2.85±0.01	
S-CoOOH	Co-O	4.97±0.48	0.0130	-6.7	1.87±0.01	0.0183
at Dark a	Co-Co	4.18±0.76	0.0170	3.9	2.91±0.04	
S-CoOOH	Co-O	5.78±0.58	0.0046	-2.2	1.89±0.02	0.0153
at Light b	Co-Co	4.43±0.55	0.0052	-2.0	2.85±0.02	
S-CoOOH	Co-O	5.90±0.42	0.0040	-3.5	1.88±0.01	0.0068
at Light c	Co-Co	4.83±0.42	0.0055	-1.4	2.84±0.02	
S-CoOOH	Co-O	5.97±0.50	0.0030	-4.3	1.88±0.02	0.0138
at Light d	Co-Co	5.16±0.57	0.0059	-3.7	2.83±0.02	

Also, we have incorporated the proposed modifications into our manuscript (lines 275-279, page 11, highlighted in yellow): To further clarify the light-matter interaction, specifically the interaction between light and electron, Fig.4d depicts the underlying photoexcitation: Light irradiation generates electron-hole pairs through electron transfer from oxygen to cobalt. The generated holes were the non-bonding oxygen species, which have then stabilized via direct O-O coupling with adjacent one. Through this way, the electron-hole pair was separated.

5. In fact, several hours light irradiations may lead to a raid thermal enhancement, whether or not the high-temperature-induced electrochemical reconfiguration is the actual reason?

Response: We are very grateful for the referee's comment. In our work, to avoid the effect of thermal on the catalytic activities, the electrochemical cell was connected to a thermostatic bath with continuous water flow. The surface temperature of catalyst, monitored by a thermocouple, was found to increase by no more than 2 ºC throughout the whole process, which was provided in our initial supplementary information (under Fig.S1, lines 1-4). In addition, we further provide a photograph of thermostatic bath connected to an electrochemical cell (Fig.R1-8), and catalyst surface temperature recorded during chronoamperometry (CA) process under light irradiation (Fig.R1-9).

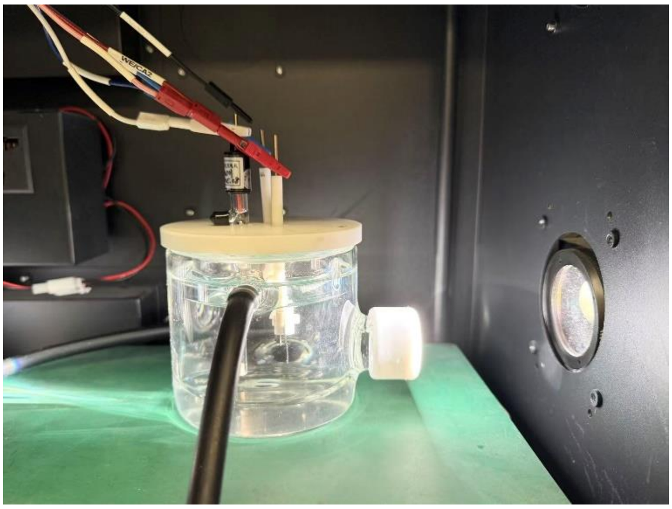

Fig.R1-8. Photograph of the actual thermostatic bath connected to an electrochemical cell. (Supplementary Fig.S9 in our Supplementary Information)

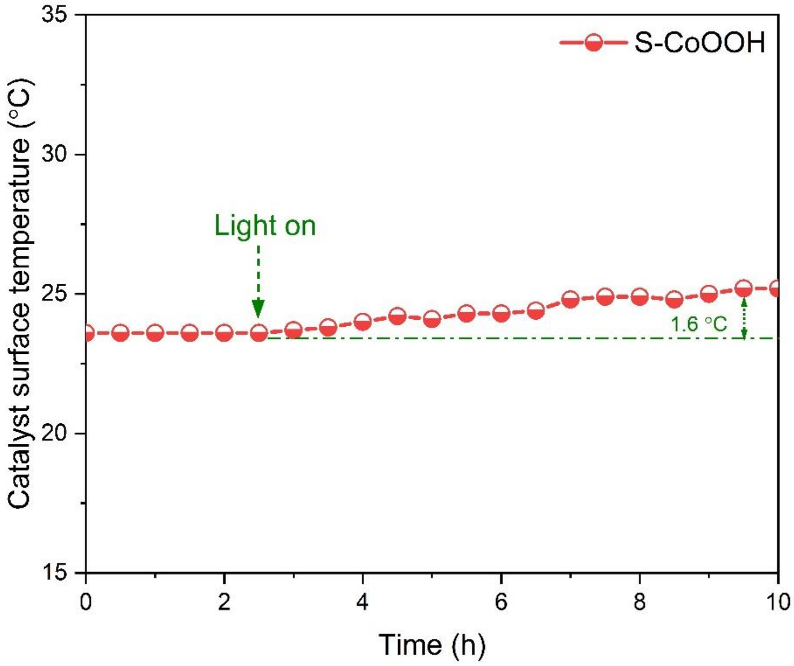

Fig.R1-9. Catalyst surface temperature recorded under light illumination during chronoamperometry (CA) process, corresponding to **Fig.1a**. (Supplementary Fig.S10 in our Supplementary Information)

To address the comment, we have incorporated the proposed modifications into our manuscript (lines 108-111, page 4, highlighted in yellow): Initially, several potential reasons for the distinctive increase in OER performance under light illumination are excluded, including thermal effect, changes in ohmic resistance and grain size, generation of light-induced holes, or any additional reaction (Supplementary Figs.S9-S15).

Also, we have included Supplementary Fig.S9 and Fig.S10 into our Supplementary Information:

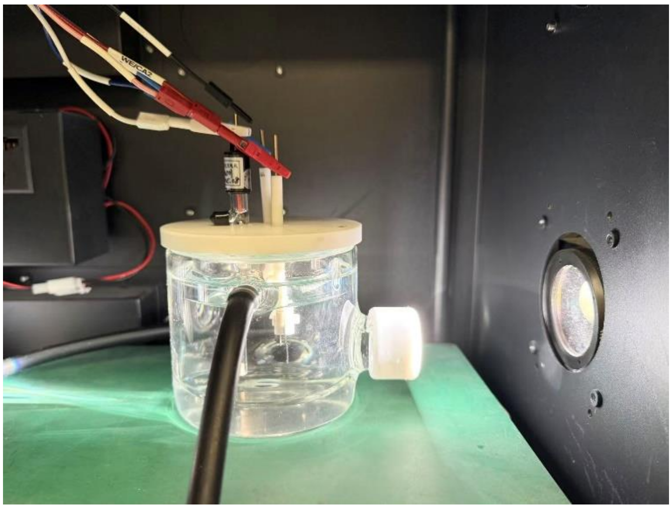

Supplementary Figure S9. Photograph of the actual thermostatic bath connected to an electrochemical cell.

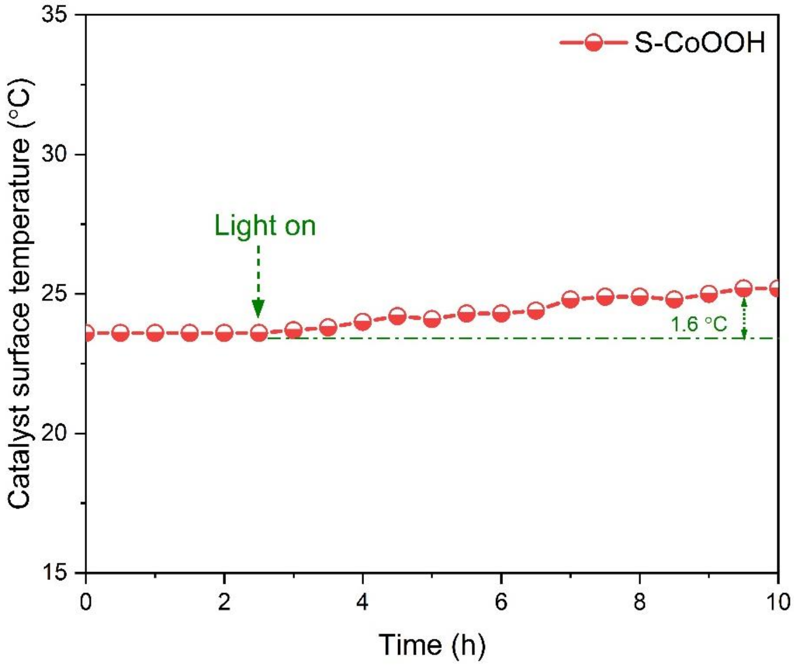

Supplementary Figure S10. Catalyst surface temperature recorded under light illumination during chronoamperometry (CA) process, corresponding to **Fig.1a**.

In addition, we have added a description of temperature control during the illumination process to the Methods section of our manuscript (lines 407-409, page 16, highlighted in yellow): Conversely, for those carried out under light conditions, the electrochemical cell was connected to a thermostatic bath with continuous water flow to maintain a constant catalyst surface temperature.

6. If the contribution of light irradiations is the key factor, the catalytic activity will be significantly enhanced by regulating their light-matter interactions. However, this behavior cannot be observed or provided.

Response: We sincerely appreciate the reviewer's insightful suggestion. In our group's previous work (*Nature* 2022, 611, 702-708; *Nature Communications* 2023, 14, 7488), we have shown that variations in light wavelength could lead to different degrees of enhancement in OER activity, suggesting a potential role of light-matter interactions. According to the reviewer's insightful suggestion, discussions on different intensities and wavelength filters of light irradiation have been provided below.

Fig.R1-10 presents the photon response measurements under varying illumination intensities, revealing that the light-induced behavior is closely related to light intensity. As the light intensity increases from 0.5633 to 0.7664, 1.0228 and 1.4901 Sun, the increase in current density becomes progressively more pronounced.

In addition, Fig.R1-11 provides the photo response measurements under different illumination wavelengths. It is shown that with a wavelength of 575 nm, the current increases significantly when light is switched on. As the wavelength increases to 800 nm, a current enhancement can still be observed. Since the calculated kinetic barrier is about 1260 nm (Fig.R1-12), it is expected that the current increment with light source of 1200 nm to be greatly weakened. As shown in Fig.R1-11, the current increment is barely detectable when a light source of 1200 nm is used. This result suggests that the wavelength of 1200 nm could trigger LMCT minimally, which is in good agreement with our calculations. Interestingly, switching the light source to a higher wavelength of 1310 nm does not trigger any observable change in the current, which suggests the inability of this wavelength to trigger LMCT. This result further validates our calculation of the kinetic energy barrier required to activate LMCT. Thus, based on these results, it can be concluded that the lowest wavelength that can trigger LMCT in S-CoOOH is about 1200 nm (1.03 eV), which is in close agreement with the calculated value (0.98 eV, 1260 nm).

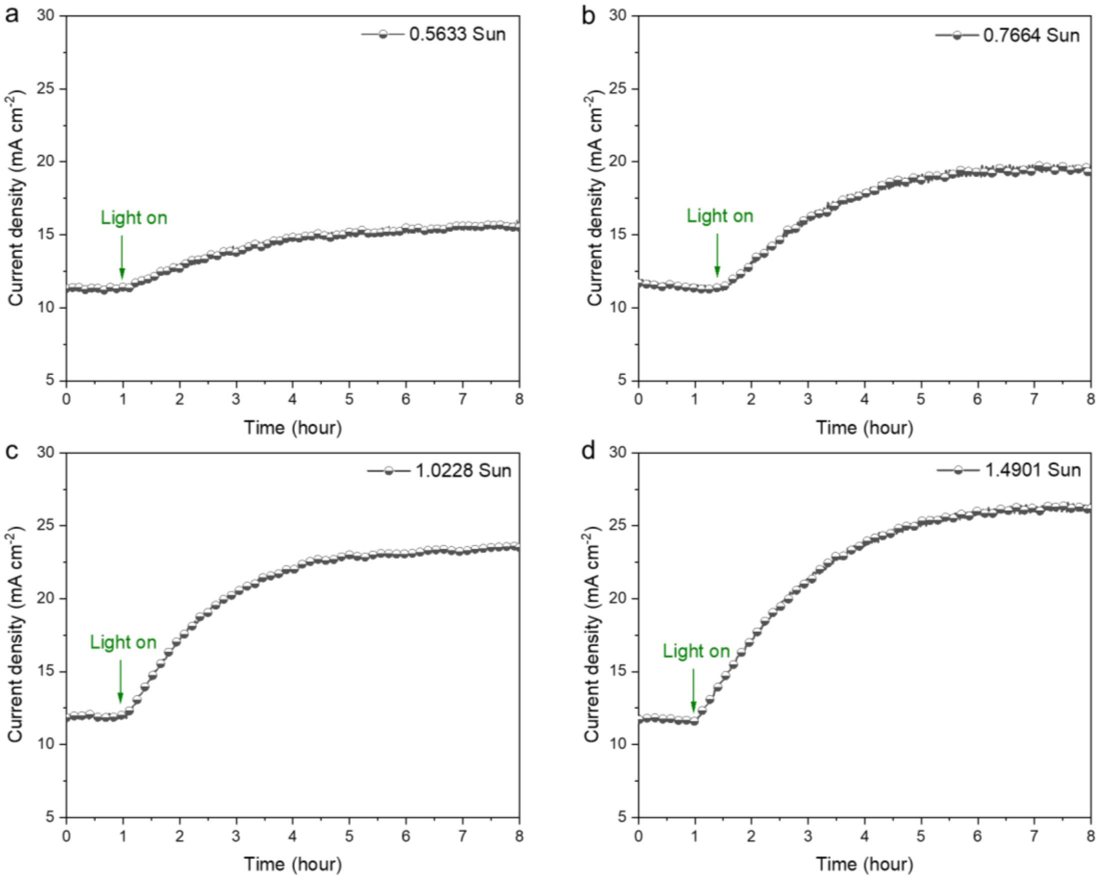

Fig.R1-10. Photon response measurements under different light intensities: **a** 0.5633 Sun (56.33 mW cm^{-2}), **b** 0.7664 Sun (76.64 mW cm^{-2}), **c** 1.0228 Sun (102.28 mW cm^{-2}), and **d** 1.4901 Sun (149.01 mW cm^{-2}). (Supplementary Fig.S7 in our Supplementary Information)

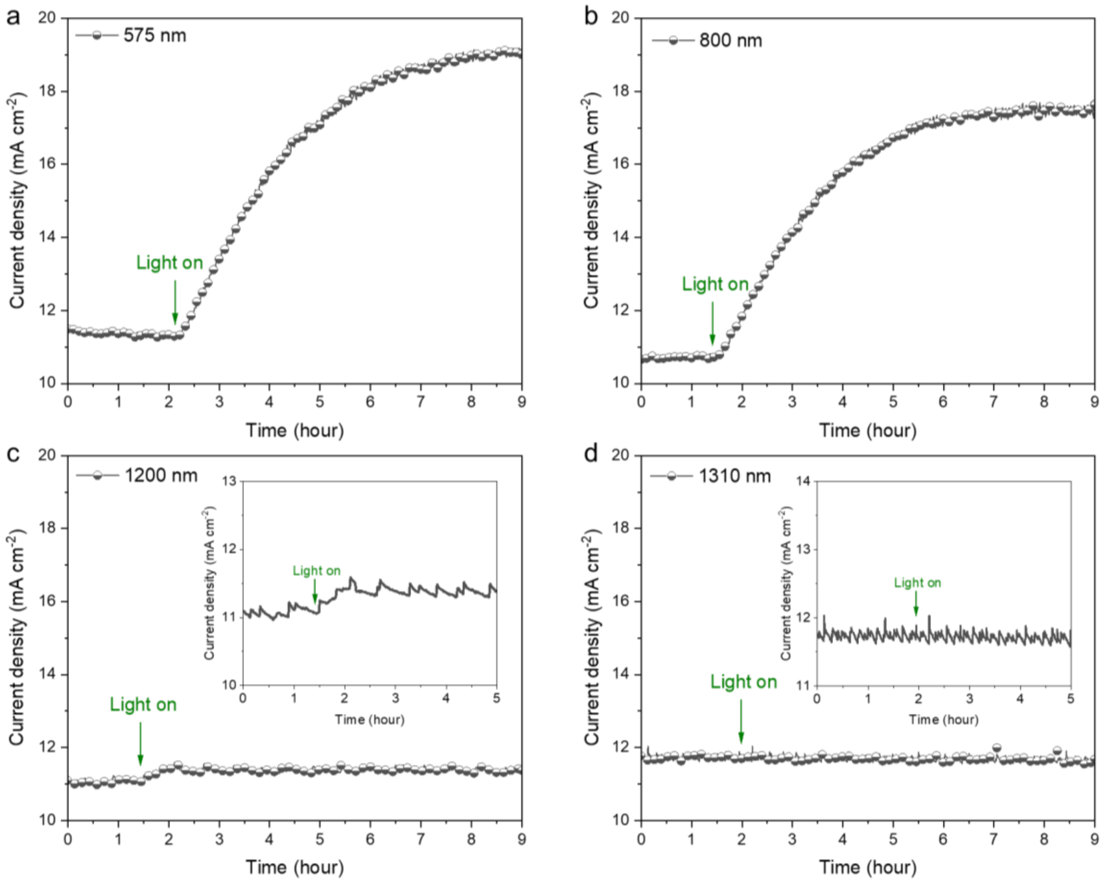

Fig.R1-11. Photon response measurements equipped with different wavelength filters of 575 nm (**a**), 800 nm (**b**), 1200 nm (**c**), and 1310 nm (**d**), respectively. (Supplementary Fig.S8 in our Supplementary Information)

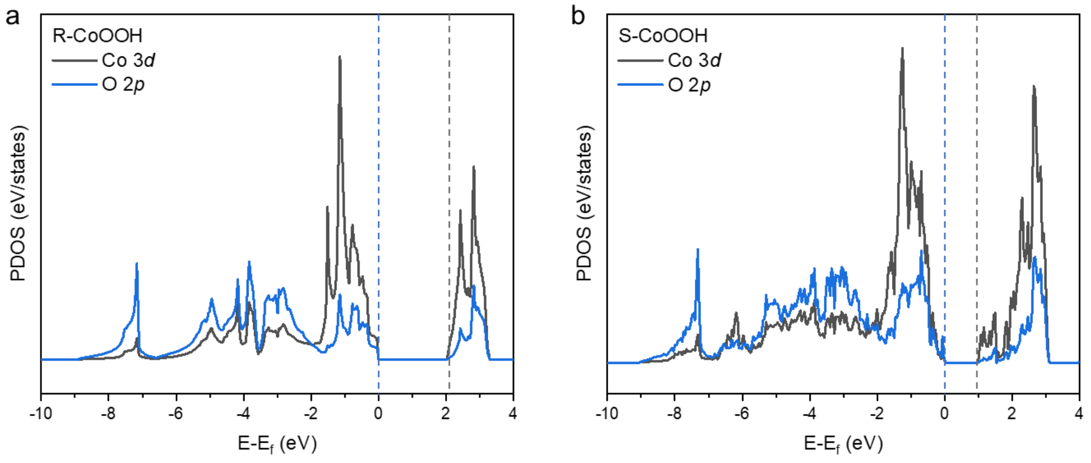

Fig.R1-12. PDOS of cobalt 3*d* and oxygen 2*p* orbitals in both R-CoOOH (**a**) and S-CoOOH (**b**). (Supplementary Fig.S23 in our previous Supplementary Information, Supplementary Fig.S32 in our revised Supplementary Information)

To address the comment, we have incorporated the proposed modifications into our manuscript (lines 107-108, page 4, highlighted in yellow): The photon response is related to the light intensity and wavelength (Supplementary Figs.S7-S8).

Besides, we have implemented the suggested versions into our manuscript (lines 322-327, page 14, highlighted in yellow): Our photon response measurements across different light wavelengths are consistent with this simulation result, showing pronounced current increase at 575 nm and 800 nm, a minimal increase at 1200 nm, and no detectable response at 1310 nm (Supplementary Fig.S8). These findings indicate that photons with an energy of over 1.03 eV ($\lambda < 1200$ nm) are required to activate LMCT in S-CoOOH, which is in close agreement with the calculated value of 0.98 eV (1260 nm).

Moreover, we have added a description of the illumination conditions to the Methods section of our manuscript (lines 410-414, page 17, highlighted in yellow): A solar simulator (NBeT Solar-500, 300 W) emulating AM 1.5 G (100 mW cm^{-2}) was used as the light source. The light intensity impinging on the electrocatalyst was regulated by adjusting the output current and irradiation distance of the solar simulator, and was monitored using a light power meter (Newport 91150). In addition, light filters with wavelengths of 575, 800, 1200, 1310 nm were applied for wavelength-dependent photon response measurements.

In addition, we have added Supplementary Fig.S7 and Fig.S8 to the Supplementary Information and included the corresponding descriptions below the figures.

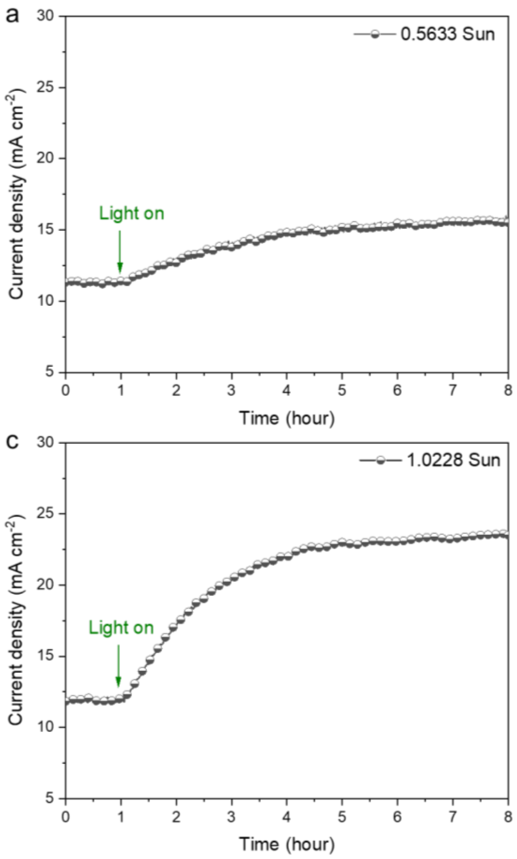

Supplementary Figure S7. Photon response measurements under different light intensities: **a** 0.5633 Sun (56.33 mW cm^{-2}), **b** 0.7664 Sun (76.64 mW cm^{-2}), **c** 1.0228 Sun (102.28 mW cm^{-2}), and **d** 1.4901 Sun (149.01 mW cm^{-2}).

Supplementary Figure S7 presents the photon response measurements under varying illumination intensities, revealing that the light-induced behavior is closely related to light intensity. As the light intensity increases from 0.5633 to 0.7664, 1.0228 and 1.4901 Sun, the increase in current density becomes progressively more pronounced.

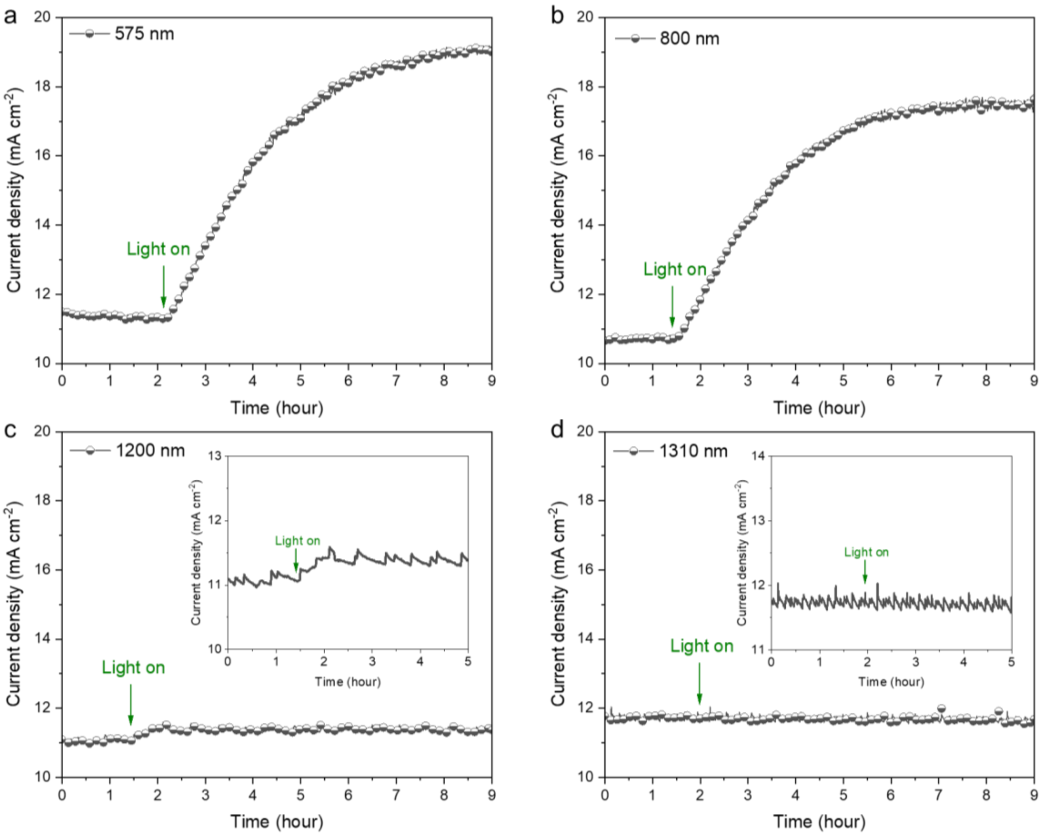

Supplementary Figure S8. Photon response measurements equipped with different wavelength filters of 575 nm (**a**), 800 nm (**b**), 1200 nm (**c**), and 1310 nm (**d**), respectively.

In addition, Supplementary Fig.S8 provides the photo response measurements under different illumination wavelengths. It is shown that with a wavelength of 575 nm, the current increases significantly when light is switched on. As the wavelength increases to 800 nm, a current enhancement can still be observed. Since the calculated kinetic barrier is about 1260 nm (Supplementary Fig.S32), it is expected that the current increment with light source of 1200 nm to be greatly weakened. As shown in Supplementary Fig.S8c, the current increment is barely detectable when a light source of 1200 nm is used. This result suggests that the wavelength of 1200 nm could trigger ligand to metal charge transfer (LMCT) minimally, which is in good agreement with our calculations. Interestingly, switching the light source to a higher wavelength of 1310 nm does not trigger any observable change in the current, which suggests the inability of this wavelength to trigger LMCT. This result further validates our calculation of the kinetic energy barrier required to activate LMCT. Thus, based on these results, it can be concluded that the lowest wavelength that can trigger LMCT in S-CoOOH is about 1200 nm (1.03 eV), which is in close agreement with the calculated value (0.98 eV, 1260 nm).

Reviewer #2

Remarks to the Author: The authors report a light-triggered LMCT from M–O to Co 3d orbitals at the edge sites of SCoOOH. This photoinduced LMCT is proposed to enhance OER activity by facilitating the participation of ONB sites in the formation of O–O intermediates during oxygen redox, effectively bypassing the rate-determining *OOH step in the conventional AEM. To elucidate the LMCT mechanism, the authors employed operando XAS and RIXS to probe the electronic structure at the edge of S-CoOOH. In addition, the OER mechanism involving O–O intermediate formation was investigated using 18O labeling combined with operando DEMS. Overall, this study presents interesting mechanism and provides comprehensive characterization. However, publication can be considered after the following revisions are addressed.

1. The authors argue that light-triggered LMCT (from M–O to O) enhances OER activity, with no evidence that electrons from the M–O bonds are photo-excited.

Response: We are very grateful for the referee's comment. When light-induced electron transfer from (M-O) to metal, the outer electron configuration of oxygen would become 7 and non-bonding oxygen (O_{NB}) species would appear. The presence of such O_{NB} species can be probed by TMAOH, as inhibition of OER kinetics upon adding TMA^+ indicates their formation (*Angew. Chem. Int. Ed.* 2017, 56, 8652-8656; *Nat. Energy* 2019, 4, 329-338).

In our work, TMAOH probing experiments were employed to identify the photo-excited O_{NB} species. The TMAOH experiment results show that, S-CoOOH exhibits a significant reduction in OER activity under light following the addition of TMA^+ (Fig.R2-1a), contrasting with its stable OER activity in dark after adding TMA^+ (Fig.R2-1b). These results demonstrate that electron transfer from M-O to Co occurs exclusively under illumination, with light acting as the driving force for this LMCT process.

To further identify this, TMA^+ probe measurements combined with Co *K*-edge XAS were employed, as shown in Fig.R2-2. Theoretically, the bonding of O_{NB} species with TMA^+ would trigger an inner electron transfer from Co atom to TMA^+, leading to an increase in cobalt valence. As shown in the Co *K*-edge spectra of S-CoOOH before and after adding TMA^+ (Fig.R2-2a), a blueshift occurs under light, signifying an increase in cobalt valence, while no detectable change is observed for S-CoOOH in dark upon TMA^+

addition (Fig.R2-2b). These results further support O_{NB} species are generated only under light, and therefore LMCT takes place exclusively under light.

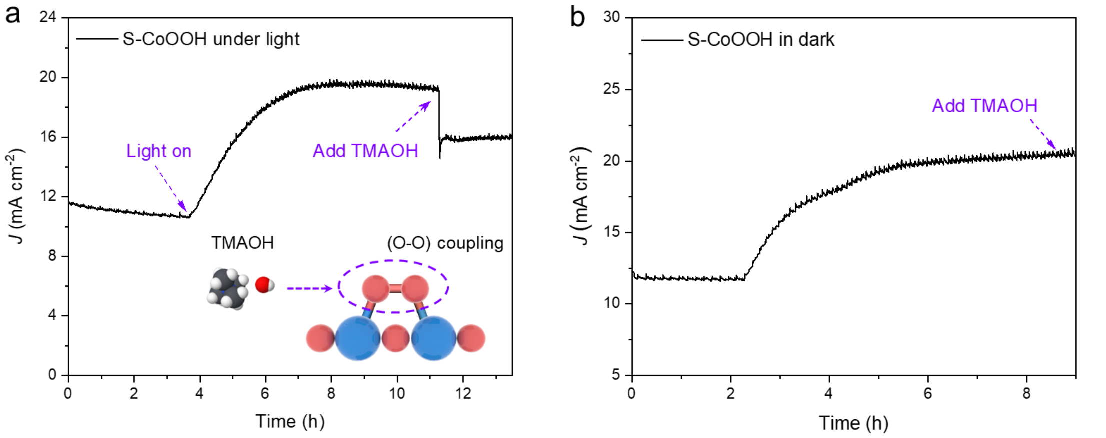

Fig.R2-1. The electrochemical curves respond to the addition of TMAOH under light (**a**) and in dark (**b**). (Fig.3a and Supplementary Fig.S15a in our previous manuscript and Supplementary Information, Fig.3a and Supplementary Fig.S23a in our revised manuscript and Supplementary Information)

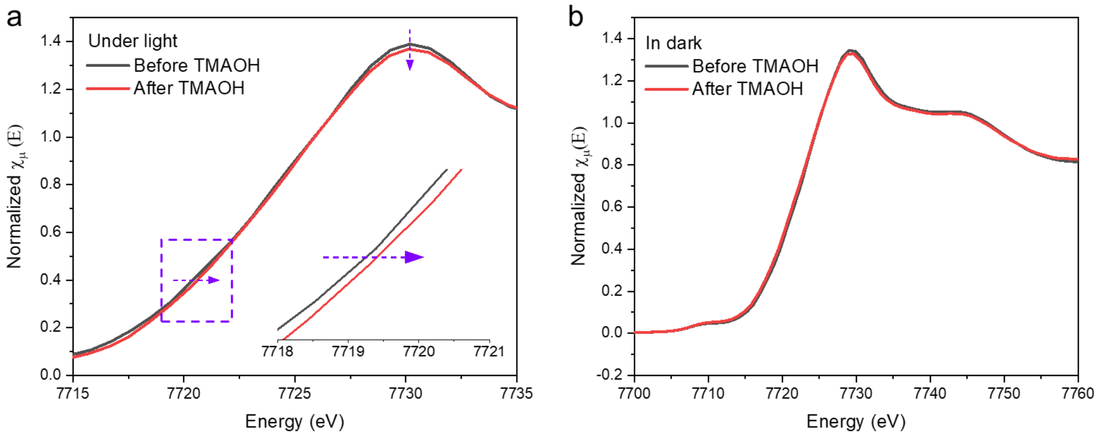

Fig.R2-2. Co *K*-edge spectra of S-CoOOH recorded under light (**a**) and in dark (**b**) conditions, before and after TMAOH addition. (Fig.3b and Supplementary Fig.S15b in our previous manuscript and Supplementary Information, Fig.3b and Supplementary Fig.S23b in our revised manuscript and Supplementary Information)

To address the comment, we have incorporated the proposed modifications into our manuscript (lines 184-185, page 8, highlighted in yellow): These results (Fig.3a,b and Supplementary Fig.S23) support the formation of O-O coupling exclusively under light, with light acting as the driving force for this light-induced LMCT process.

2. S-CoOOH was prepared by oxidizing cobalt sulfides, but cobalt sulfides are photoelectrode materials. Have authors considered the remaining cobalt sulfides?

Response: We sincerely appreciate the referee's valuable suggestion. In our work, we conducted a long-time electrochemical oxidation procedure on cobalt sulfides to ensure a complete conversion into CoOOH, leaving negligible residual cobalt sulfides, as evidenced by our prior work (Figs.S1-S5 in *Nat. Commun.* 2024, 15, 1383). X-ray diffraction (XRD), Raman spectra, and X-ray absorption spectroscopy (XAS) (Fig.R2-3), showing that cobalt sulfide is completely oxidized into CoOOH. Besides, the Co *K*-edge FT-EXAFS results indicate that all Co-S bonds have been broken in S-CoOOH after electrochemical oxidation (Fig.R2-3d).

[FIGURE REDACTED]

Fig.R2-3. Structural characterization of as-prepared Co_3S_4 and after activation S-CoOOH, where Co_3S_4 is marked in black and S-CoOOH is marked in red. **a** XRD patterns of Co_3S_4 and S-CoOOH with standard peaks of Co_3S_4 and β-CoOOH listed. **b** Raman spectra of Co_3S_4 and S-CoOOH. **c** Normalized Co *K*-edge XAS spectra of Co_3S_4 and S-CoOOH. **d** FT-EXAFS spectra of Co *K*-edge of Co_3S_4 and S-CoOOH. (Our prior work in *Nat. Commun.* 2024, 15, 1383)

Meanwhile, according to the reviewer's advice, X-ray photoelectron spectroscopy (XPS), Fourier transformed extended X-ray near fine structure (FT-EXAFS), and Inductively coupled plasma (ICP) characterizations are supplemented in our work (Fig.R2-4). In the high-resolution XPS spectra of S 2*p* for cobalt sulfides before and after activation, no peaks assigned to S 2*p* are observed in S-CoOOH, indicating that cobalt sulfides undergo complete conversion during OER process (Fig.R2-4a). As shown in the FT-EXAFS spectra, the Co-S bond peak disappears after electrochemical oxidation, indicating that all Co-S bonds are broken (Fig.R2-4b). In addition, the ICP result shows an almost negligible signal S element in S-CoOOH, which excludes the presence of residual cobalt sulfides (Table R2-1). Therefore, the presence of residual cobalt sulfides can be excluded, and thus the enhanced light response cannot originate from cobalt sulfides.

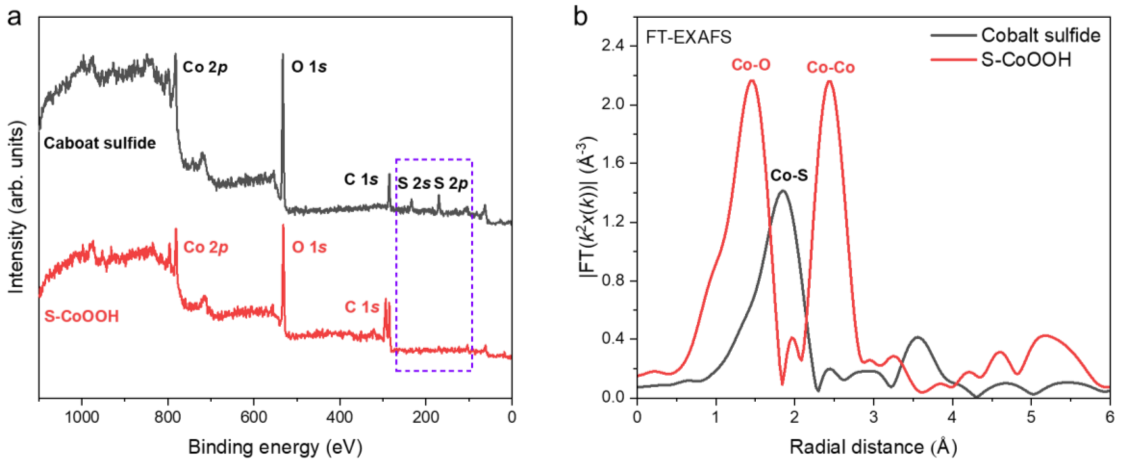

Fig.R2-4 a XPS spectra of sample before (cobalt sulfide) and after electro-oxidation (S-CoOOH) recorded over a wide range. **b** FT-EXAFS spectra of sample before (cobalt sulfide) and after electro-oxidation (S-CoOOH). (Supplementary Fig.S1b and Fig.S2b in our revised Supplementary Information)

Table R2-1. ICP results of S-CoOOH, showing the corresponding mass/atomic ratios

(Supplementary Table S1 in our revised Supplementary Information)

Element	Co	S
Mass ratio (w/w%)	39.94	0.41
Atomic ratio (at%)	98.16	1.84

To address the comment, we have incorporated the proposed modifications into our manuscript (lines 88-90, page 4, highlighted in yellow): The cobalt sulfides were completely reconstructed into β-CoOOH, leaving negligible residual sulfur, as confirmed by X-ray diffraction (XRD), X-ray photoelectron spectroscopy (XPS), Co *K*-edge XAS, and inductively coupled plasma (ICP) measurements (Supplementary Figs.S1-S2 and Table S1).

Besides, we have also added a section titled "Structural characterization of S-CoOOH" into our Supplementary Information (Supplementary Figs.S1-S2 and Table S1):

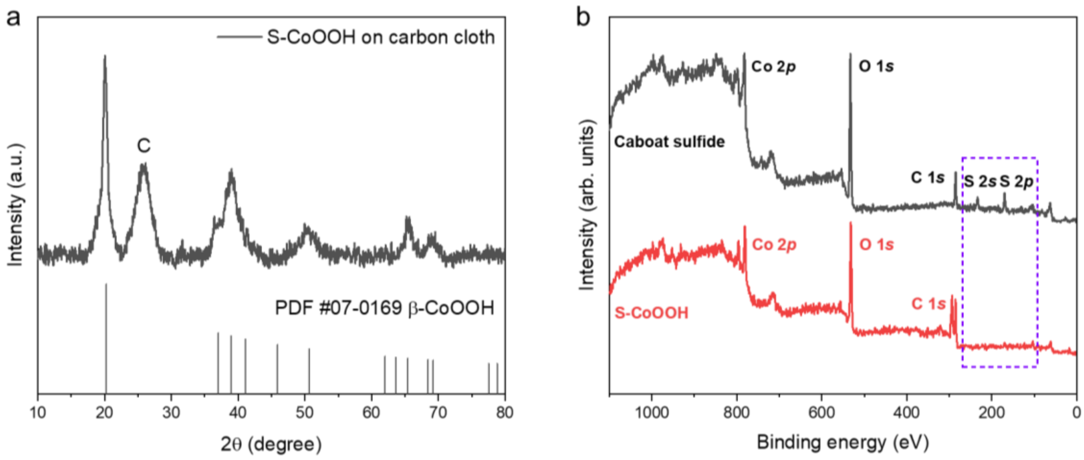

Supplementary Figure S1. a XRD pattern of S-CoOOH based on carbon cloth, with standard β-CoOOH listed (PDF #07-0169). **b** XPS spectra of cobalt sulfide before (top) and after activation (bottom) in a wide range.

In this section, after long-term chronopotentiometry (CP) treatment, cobalt sulfide is verified to convert into CoOOH with negligible residual sulfur, as confirmed by X-ray diffraction (XRD), X-ray photoelectron spectroscopy (XPS), X-ray absorption spectroscopy (XAS), and inductively coupled plasma (ICP) analysis (Supplementary Figs.S1-S2, Table S1). As shown in Supplementary Fig.S1a, after a long-time electro-oxidation process (10-hour CP), XRD result reveals that cobalt sulfide pre-catalyst is fully reconstructed into rhombohedral β-CoOOH (R-3m space group, JCPDS 07-0169). Besides, Supplementary Fig.S1b presents the wide-range XPS spectra of cobalt sulfide before and after activation. The peaks at binding energies of 160, 221, 279, 527, and 780 eV correspond to S 2*p*, S 2*s*, C 1*s*, O 1*s*, and Co 2*p*, respectively[1]. After long-time activation, the S 2*p* and S 2*s* signals disappear entirely in S-CoOOH.

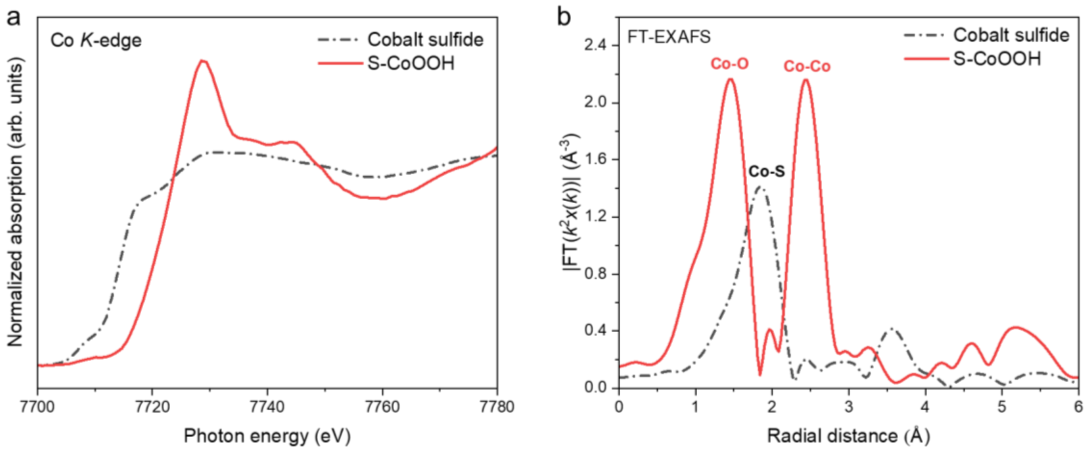

Supplementary Figure S2. a Normalized Co *K*-edge XAS spectra of cobalt sulfide and S-CoOOH. **b** FT-EXAFS spectra of Co *K*-edge of cobalt sulfide and S-CoOOH.

In addition, the structural characterization of cobalt sulfide before and after electro-oxidation is studied using XAS at Co *K*-edge. As shown in Supplementary Fig.S2a, cobalt sulfide undergoes electro-oxidation to S-CoOOH, exhibiting a red-shifted absorption edge and higher white-line intensity, which reflects a significant phase transformation. Supplementary Fig.S2b presents the corresponding Fourier-transformed extended X-ray near fine structure (FT-EXAFS) spectra. The peaks associated with Co-S bonds in cobalt sulfide disappear fully in S-CoOOH, indicating that all Co-S bonds are broken after electro-oxidation.

Supplementary Table S1. ICP results of S-CoOOH, showing the corresponding mass/atomic ratios

Element	Co	S
Mass ratio (w/w%)	39.94	0.41
Atomic ratio (at%)	98.16	1.84

3. The LMCT has been triggered in S-CoOOH. What is photophysical property of the SCoOOH to support the authors' explanation.

Response: We are grateful for the referee's valuable suggestion. According to the suggestion, we carried out UV-Vis absorption measurements to probe the photophysical properties of S-CoOOH. The black S-CoOOH powder exhibits intense light absorption across a broad spectral range of 200 to 2400 nm (Fig.R2-5). As discussed in the main text (lines 320-322, pages 12-13), our PDOS analysis indicates that light-induced

LMCT in S-CoOOH requires a minimum excitation energy of approximately 0.98 eV, corresponding to photons with wavelengths shorter than about 1260 nm. The measured UV-Vis spectrum therefore provides photophysical evidence that light-induced LMCT is feasible in S-CoOOH, as it exhibits sufficient absorption well within the required wavelength range.

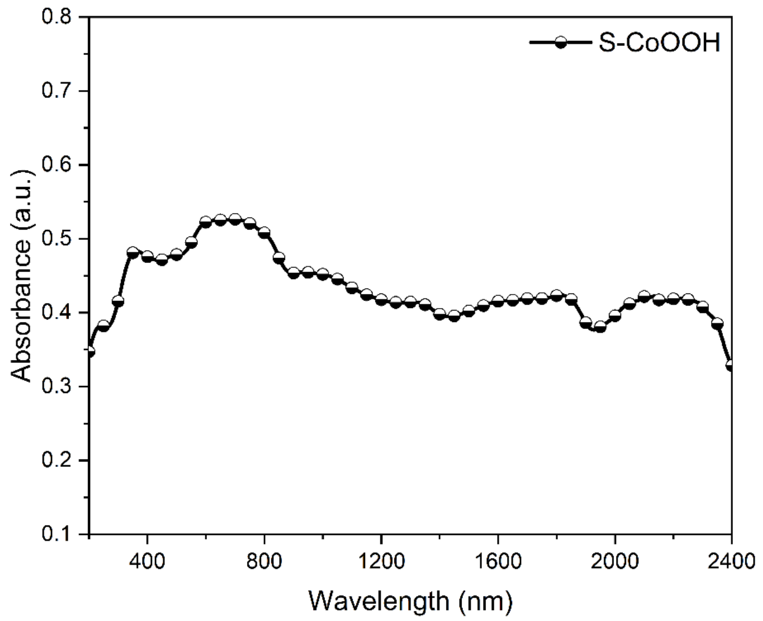

Fig.R2-5. UV-Vis absorption spectroscopy of S-CoOOH powder measured in the 200-2400 nm range. (Supplementary Fig.S33 in our revised Supplementary Information)

To address the comment, we have incorporated the proposed modifications into our manuscript (lines 327-329, page 14, highlighted in yellow): The photophysical property of S-CoOOH was further examined by UV-Vis absorption spectroscopy (Supplementary Fig.S33), which reveals intense light absorption over 200-2400 nm, confirming that light-induced LMCT in S-CoOOH is feasible.

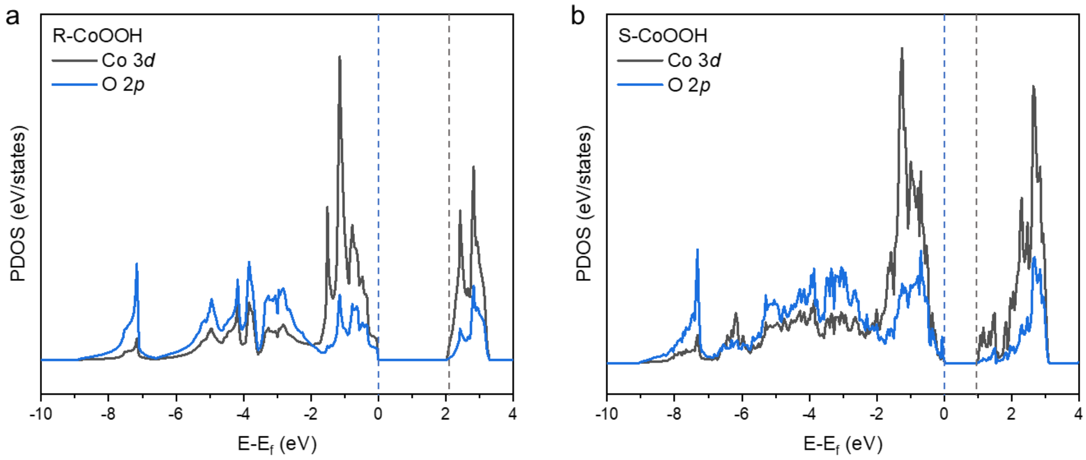

Supplementary Figure S32. PDOS of cobalt 3*d* and oxygen 2*p* orbitals in both R-CoOOH (**a**) and S-CoOOH (**b**). (Supplementary Fig.S23 in our previous Supplementary Information, Supplementary Fig.S32 in our revised Supplementary Information)

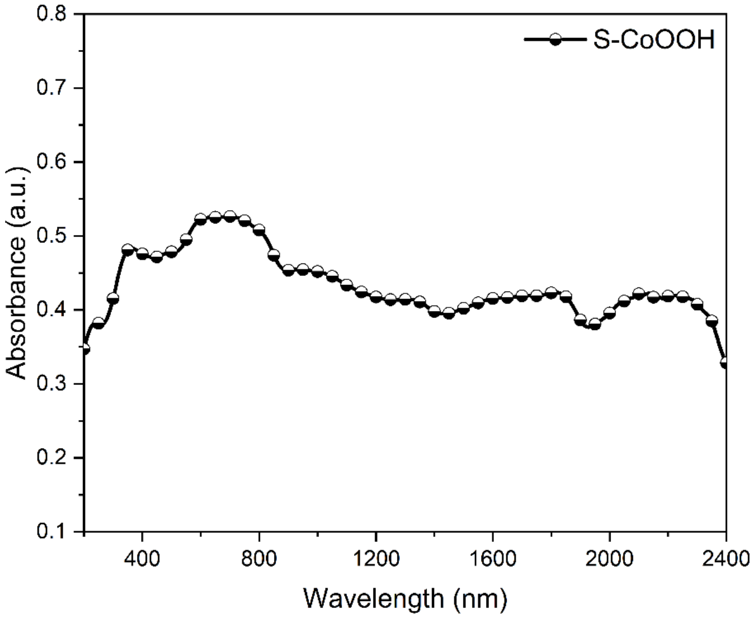

Supplementary Figure S33. UV-Vis absorption spectroscopy of S-CoOOH powder measured in the 200-2400 nm range. (Supplementary Fig.S33 in our revised Supplementary Information)

4. What potential was applied during the operando XAS? More experimental details needed.

Response: We sincerely appreciate the referee's useful suggestion. An applied potential of 1.43 V vs. RHE was used to maintain a constant current of approximately 2 mA. Accordingly, we have revised the description of *operando* X-ray absorption spectroscopy (XAS) in the Methods section.

To address the comment, we have incorporated the proposed modifications into our manuscript (lines 442-445, page 18, highlighted in yellow): During operation, to minimize interference from generated bubbles and avoid structural impacts from voltage variation, the electrochemical experiment was conducted at a constant low voltage of 1.43 V vs. RHE, with adjustments made to maintain an initial current of approximately 2 mA.

5. Authors mentioned changes in the valence states of Co sites during the OER process. Structural reconstruction commonly happens in oxyhydroxides during OER, causing alterations of crystal structure. The bulk structure should be analyzed before and after the OER.

Response: We are very grateful for the referee's insightful suggestion. We agree that structural reconstruction might happen during OER, leading to irreversible alterations in crystal structure. According to the referee's useful advice, we provide Co *K*-edge XAS spectra of S-CoOOH before and after OER under light irradiation (Fig.R2-6), which are nearly identical, indicating no structural reconstruction. At the same time, during OER test under light irradiation, our sample S-CoOOH exhibits reversible behavior in electrochemical performance, further confirming that no further structural reconstruction takes place (Fig.R2-7).

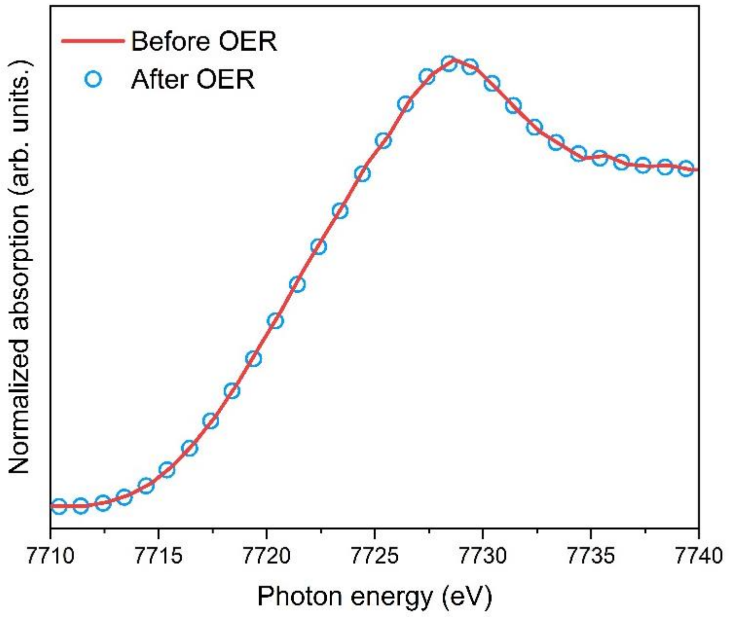

Fig.R2-6. Normalized Co *K*-edge XAS spectra of S-CoOOH before and after OER under light irradiation. (Supplementary Fig.S16 in our revised Supplementary Information)

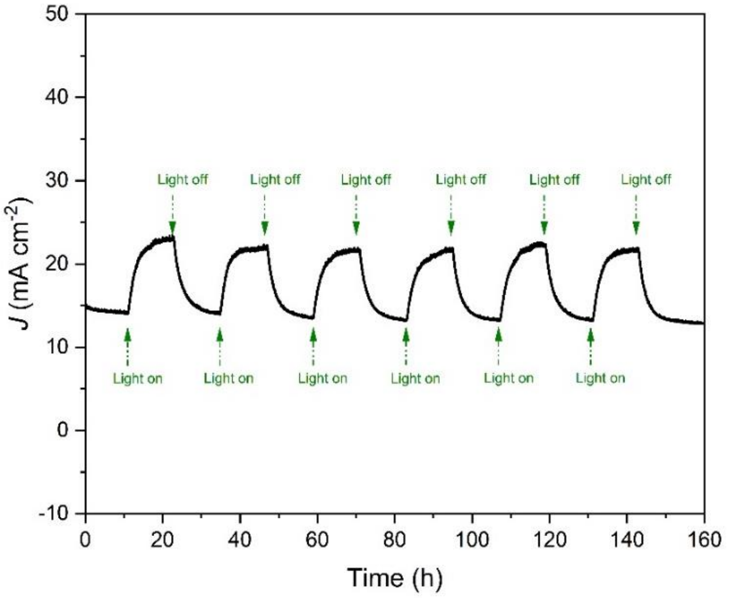

Fig.R2-7. The repeatability experiments for electrochemical measurements of S-CoOOH. (Supplementary Fig.S1 in our previous Supplementary Information, Supplementary Fig.S3 in our revised version)

To address the comment, we have incorporated the proposed modifications into our manuscript (lines 111-112, page 4, highlighted in yellow): Besides, structural reconstruction is also excluded, as the Co *K*-edge XAS before and after OER under light irradiation remain nearly identical (Supplementary Figure S16).

Also, we have added the relevant description below Supplementary Fig.S16:

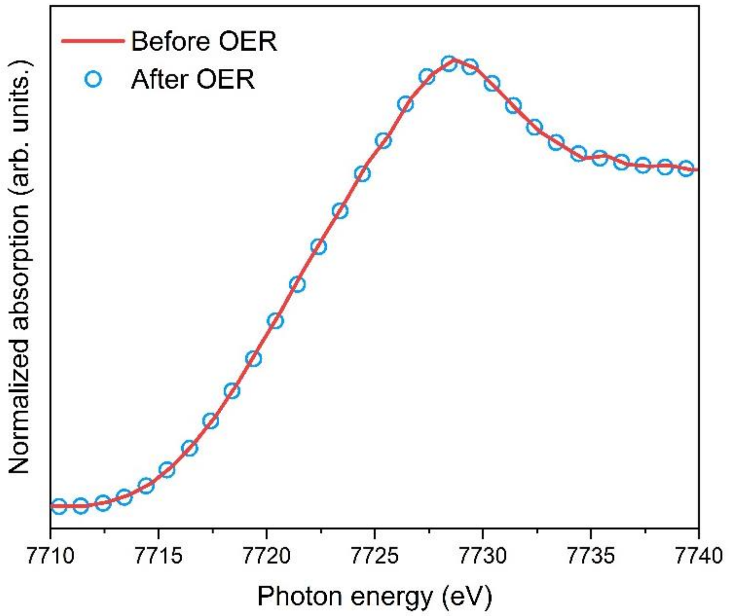

Fig.S16. Normalized Co *K*-edge XAS spectra of S-CoOOH before and after OER under light irradiation.

Structural reconstruction is also excluded. As displayed in Supplementary Fig.S16, normalized Co *K*-edge XAS of S-CoOOH before and after OER under light irradiation are nearly identical, indicating no structural changes. Also, during the OER test under light irradiation, S-CoOOH exhibits reversible behavior in electrochemical performance, further confirming that no additional structural reconstruction occurs (Supplementary Fig.S3).

6. In Figure 2d, changes in the absorption edge are not properly presented. need to show clearly this region and the shifts.

Response: We sincerely appreciate the referee's useful suggestion. According to the referee's advice, we have enlarged the absorption edge region, as shown in Fig.R2-8, to clearly present the changes and highlight the shifts. In addition, in the supplementary information, we provided a further enlarged Co *K*-edge XAS absorption edge region, as shown in Supplementary Fig.S20 (previously Fig.S13; corresponding to Fig.R2-9 below).

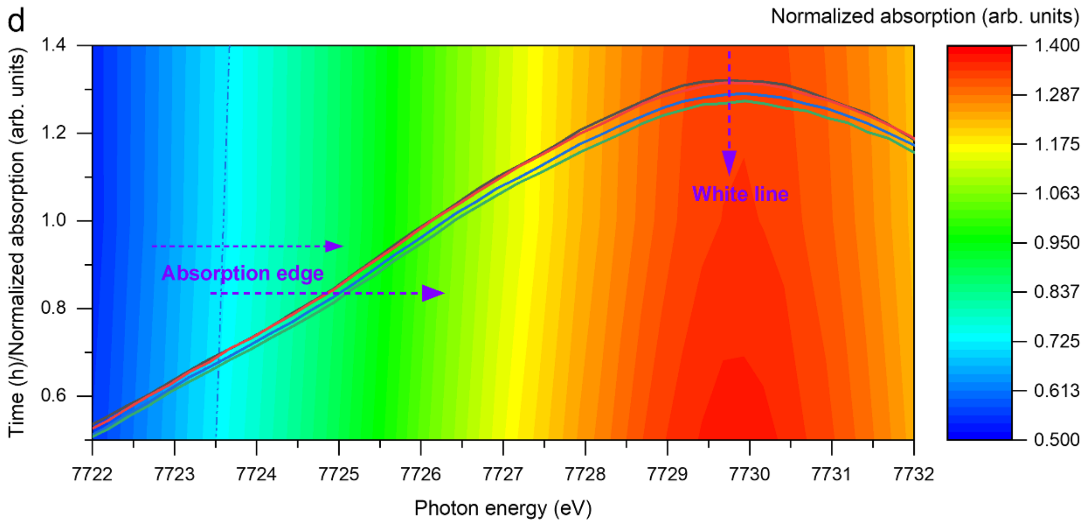

Fig.R2-8. Revised version of Fig.2d in the original manuscript. (Fig.2d in our previous manuscript, Fig.2d in our revised manuscript)

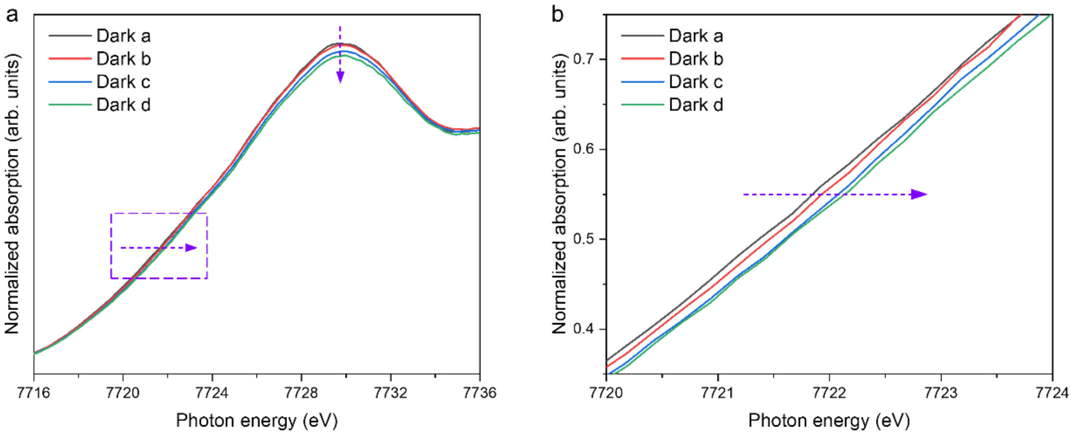

Fig.R2-9. a *Operando* Co *K*-edge XAS spectra of S-CoOOH during OER in the dark. Dark a, Dark b, Dark c, and Dark d spectra were recorded successively during OER. **b** Enlarged Co *K*-edge XAS absorption edge region, extracted from Fig.R2-9a. (Supplementary Fig.S13 in our previous Supplementary Information, Supplementary Fig.S20 in our revised Supplementary Information)

To address the comment, we have revised Fig.2d accordingly in our revised manuscript (lines 115-123, page 5, highlighted in yellow):

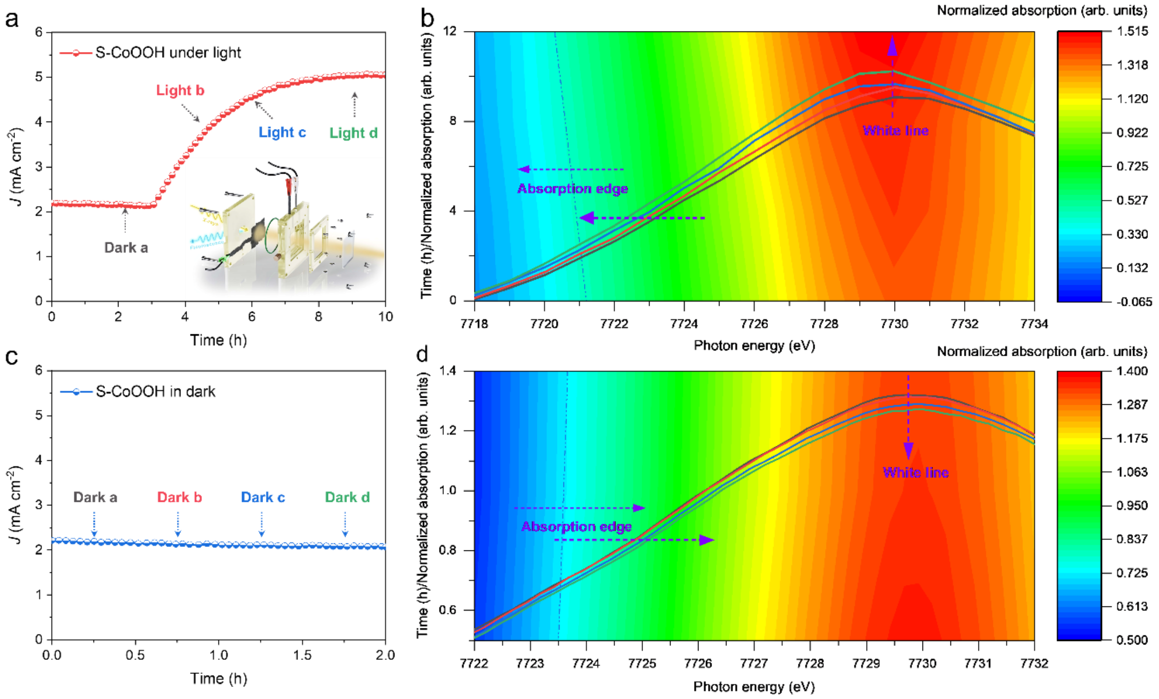

Figure 2. Identification of O_{NB} states formation in S-CoOOH subjected to light irradiation. a Electrochemical curve for *operando* Co *K*-edge XAS measurements recorded during OER from dark to illumination at four selected points: Dark a, Light b, Light c, and Light d. The inset is a schematic of

operando XAS setup, with carbon rod as the counter electrode and Ag/AgCl as the reference electrode. **b** *Operando* Co *K*-edge XAS spectra of S-CoOOH during OER under light, with dashed purple lines revealing spectral shifts. **c** Electrochemical curve for *operando* Co *K*-edge XAS measurements in the dark, recorded during the chronoamperometry (CA) process at four selected points: Dark a, Dark b, Dark c, and Dark d. **d** *Operando* Co *K*-edge XAS spectra of S-CoOOH during OER in the dark, with dashed purple lines indicating spectral shifts. (Fig.2 in our previous manuscript, Fig.2 in our revised manuscript)

7. TMA+ was used as the O–O intermediate probe and intended to interact with O–O intermediates. TMA^+ likely alters the valence state of Co. Have direct reactivities of TMA+ with surficial Co ions been considered?

Response: We agree with the reviewer's comment that TMA^+ alters the valence state of Co. Theoretically, the bonding of O_{NB} species with TMA^+ would trigger an inner electron transfer from Co atom to TMA^+, leading to an increase in cobalt valence (*Nature* 2022, 611, 702-708). As shown in the Co *K*-edge XAS spectra of S-CoOOH before and after TMA^+ (Fig.R2-10a), a blueshift occurs under light, signifying an increase in cobalt valence, while no detectable change is observed for S-CoOOH in dark upon TMA^+ addition (Fig.R2-10b).

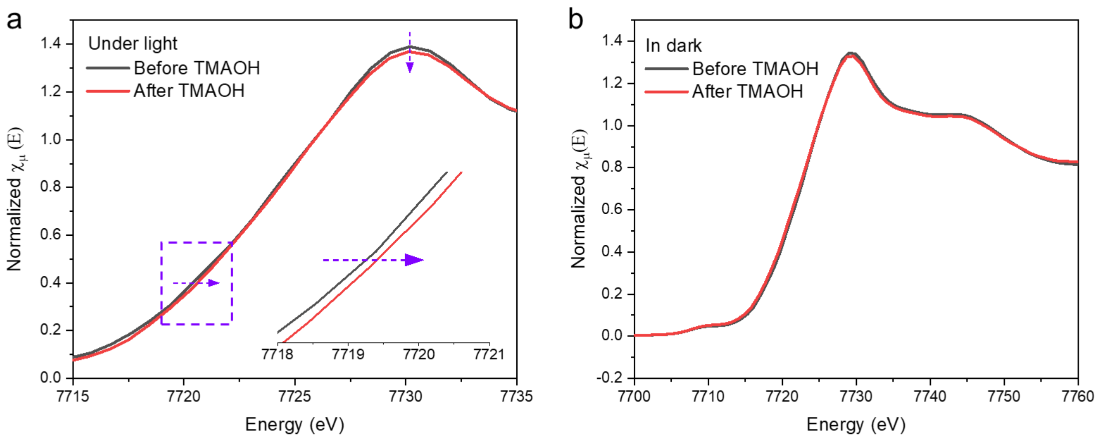

Fig.R2-10. Co *K*-edge spectra of S-CoOOH recorded under light (**a**) and in dark (**b**) conditions, before and after TMAOH addition. (Fig.3b and Supplementary Fig.S15b in our previous manuscript and Supplementary Information, Fig.3b and Supplementary Fig.S23b in our revised manuscript and Supplementary Information)

To address the comment, we have incorporated the proposed modifications into our manuscript (lines 178-184, page 8, highlighted in yellow): Besides, TMA^+ probe combined with Co *K*-edge XAS can reveal changes in the valence state of Co upon adding TMA^+. When O-O coupling is present, the addition of TMA^+

triggers an inner electron transfer from Co atom to TMA^+, leading to an increase in cobalt valence[22]. As shown in the Co *K*-edge spectra of S-CoOOH before and after adding TMA^+ (Fig.3b), a blueshift occurs under light, signifying an increase in cobalt valence, while no detectable change is observed for S-CoOOH in the dark upon TMA^+ addition (Supplementary Fig.S23b).

8. In Figure 3d, the light was turned on after 10 minutes and 32O2 increased after 20 minutes. Clarify discrepancy from the 18O experiment.

Response: We are very grateful for the referee's comment. The ratio of $^{32}O_2$ to $^{28}N_2$ shows a slight increase from 10 minutes onwards, which is consistent with the time when the light is turned on. The misunderstanding may arise from the chosen y-axis range, which we have narrowed to present the trend more clearly (Fig.R2-11).

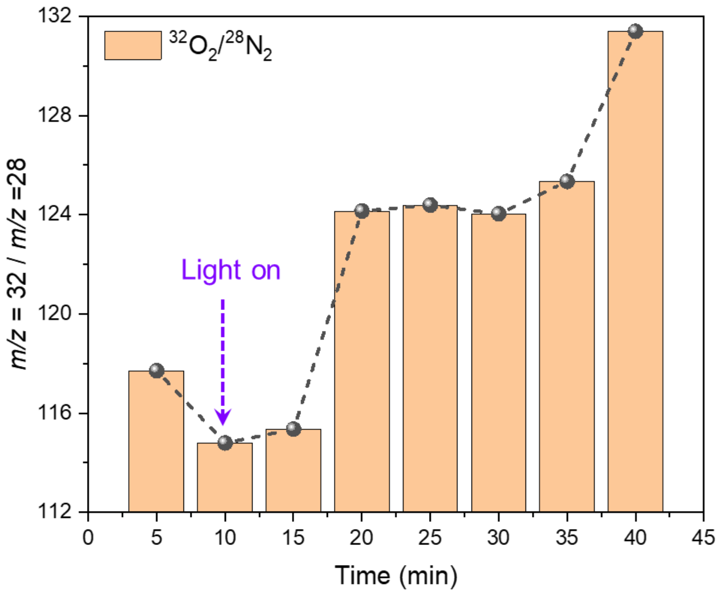

Fig.R2-11. The average ratio of $^{32}O_2$ to $^{28}N_2$ measured every 5 min (300 s). (Fig.3d in our previous manuscript, Fig.3d in our revised manuscript)

To address the comment, we have revised Fig.3d in our revised manuscript accordingly (lines 159-166, page 7, highlighted in yellow):

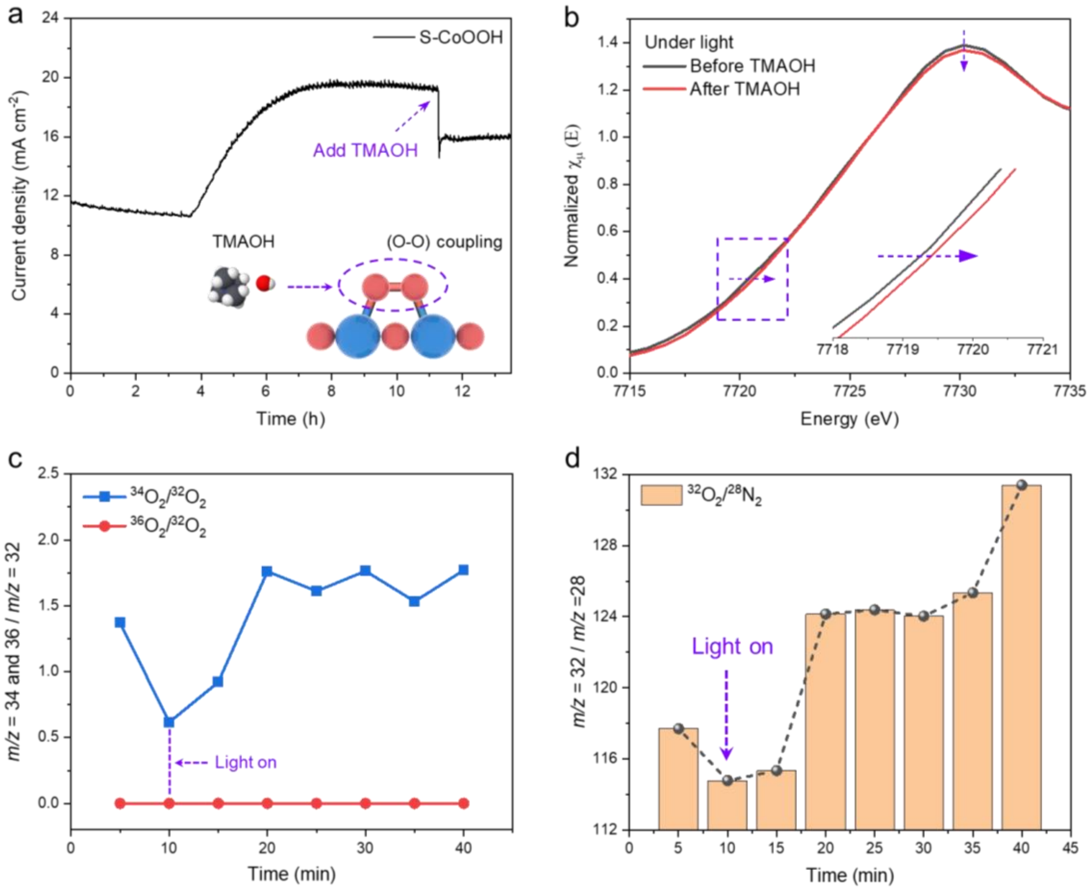

Figure 3. Confirmation of O_{NB} states participation in the redox activity subjected to light irradiation. **a** The electrochemical curves respond to the addition of TMAOH under light, where the inset is the schematic diagram of the interaction between TMA^+ cations and O_{NB} states. **b** Co *K*-edge spectra of S-CoOOH subjected to light irradiation before and after adding TMAOH. **c, d** ^{18}O isotope labeling experiments. To minimize the impacts of residual gas in the electrochemical cell and tube on the gas chromatography-mass spectrometry (GC-MS) analysis, the average ratio of $^{34}O_2$ to $^{32}O_2$ and $^{36}O_2$ to $^{32}O_2$ (**c**), as well as $^{32}O_2$ to $^{28}N_2$ (**d**) were measured every 300 s. (Fig.3 in our previous manuscript, Fig.3 in our revised manuscript)

9. In Figure 4, the color choice for atoms is confusing.

Response: We sincerely appreciate the referee's useful suggestion. According to the referee's advice, we have adjusted the atom colors in Fig.4b and 4c to ensure consistency (Fig.R2-12).

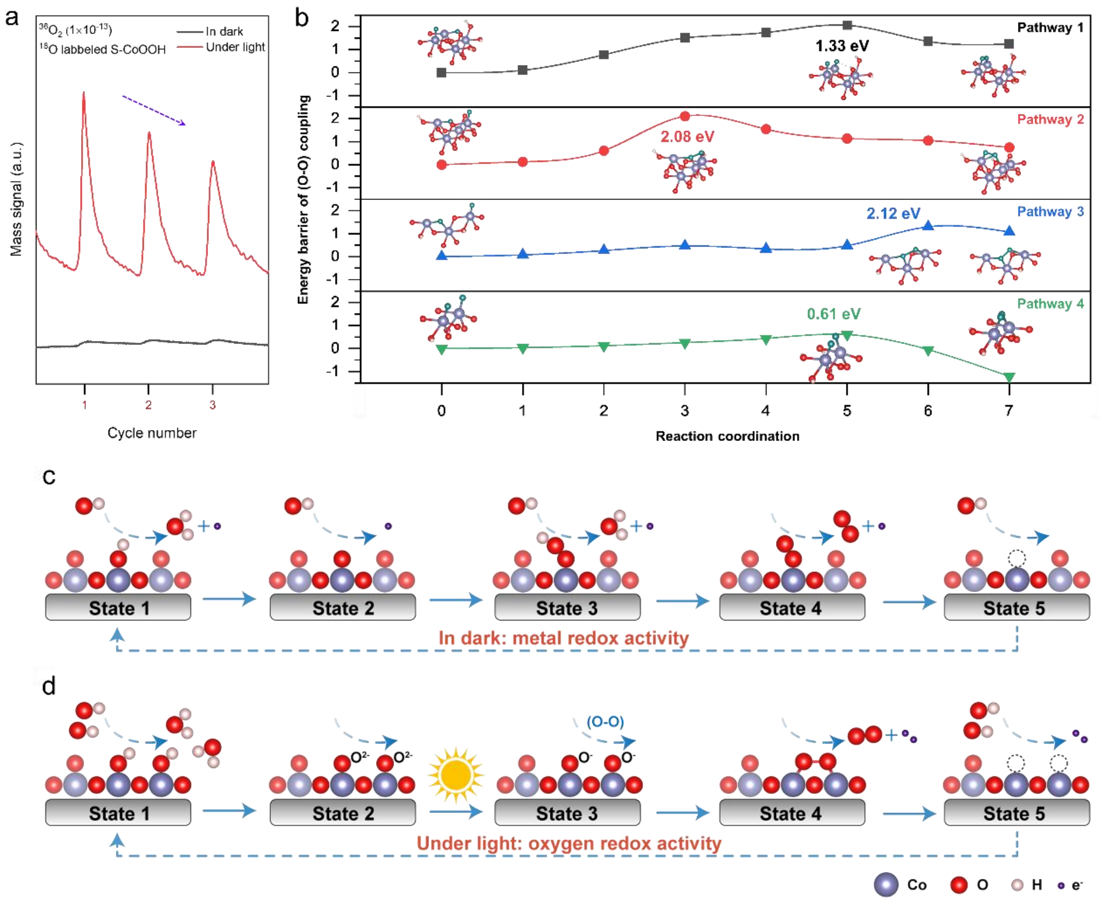

Fig.R2-12. Determination of sites of oxygen redox activity. a Differential electrochemical mass spectrometry (DEMS) signals of O_2 products for S-CoOOH measured with and without light irradiation. **b** Energy profile diagram of O-O coupling involving oxygen atoms located either in the bulk or at the edge. The inset shows the proposed O-O coupling occurring at different oxygen sites, referred to as Pathway 1-4, where oxygen atoms marked in green represent the atoms involved in O-O coupling. **c** Electron transfer pathway of S-CoOOH in the dark, where cobalt acts as a redox center. **d** Electron transfer pathway of S-CoOOH under light, where oxygen serves as a redox center with LMCT triggered O_{NB} states formation. (Fig.4 in our previous manuscript, Fig.4 in our revised manuscript)

To address the comment, we have revised the color of atoms in Figure 4 accordingly in our revised manuscript (lines 208-215, page 9, highlighted in yellow):

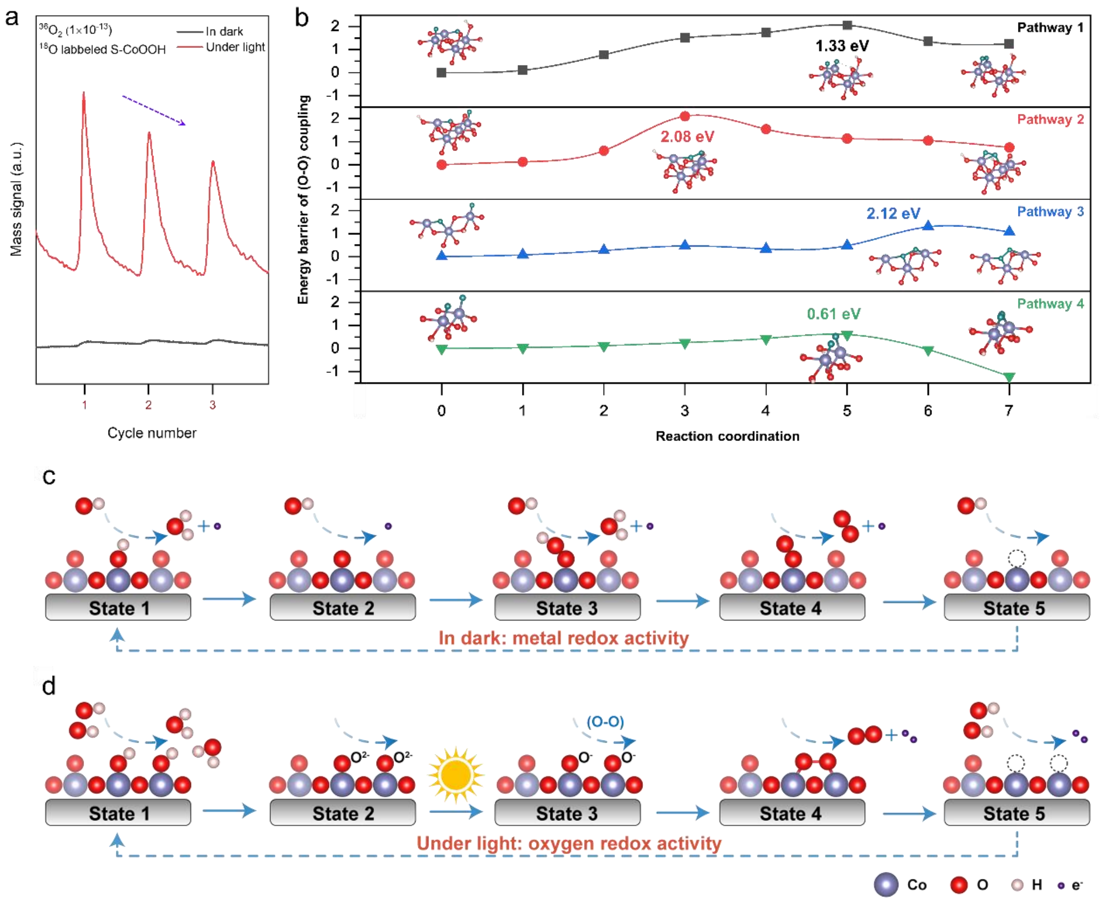

Figure 4. Determination of sites of oxygen redox activity. a Differential electrochemical mass spectrometry (DEMS) signals of O_2 products for S-CoOOH measured with and without light irradiation. **b** Energy profile diagram of O-O coupling involving oxygen atoms located either in the bulk or at the edge. The inset shows the proposed O-O coupling occurring at different oxygen sites, referred to as Pathway 1-4, where oxygen atoms marked in green represent the atoms involved in O-O coupling. **c** Electron transfer pathway of S-CoOOH in the dark, where cobalt acts as a redox center. **d** Electron transfer pathway of S-CoOOH under light, where oxygen serves as a redox center with LMCT triggered O_{NB} states formation. (Fig.4 in our previous manuscript, Fig.4 in our revised manuscript)

10. The proposed reaction mechanism suggests that hydroxyl plays a crucial role. Is this role specific to alkaline conditions? A comparative study with acidic conditions could enrich the paper further.

Response: We are very grateful for the referee's comment. Until now, the light-induced new mechanism has mainly been found in transitional metal oxyhydroxides, i.e., CoOOH for our work, NiOOH for coupled oxygen evolution mechanism (COM, *Nature* 2022, 611, 702-708). Yet, the transitional metal oxyhydroxides were unstable in acidic condition. As such, for the CoOOH species the proposed mechanism might be

difficult to realize in acidic condition. Yet, the widely used acidic OER electrocatalysts were RuO_2 and IrO_2, which have the same octahedral geometry as CoOOH. Under light irradiation, there might be a similar electron transfer from (M-O) to metal, leading to a new OER mechanism. Extending the role of light to acidic catalysts is a meaningful direction and this will be the focus of our future work.

To address the comment, we have incorporated the proposed modifications into our manuscript (lines 351-354, page 15, highlighted in yellow): This work elucidates the role of light in enhancing the OER performance of metal oxyhydroxide-based alkaline catalysts, and such light-induced effect shows promising potential for extension to acidic catalysts that share a similar octahedral structure.

Reviewer #3

Remarks to the Author: Zhang's study reported that the light irradiation triggers ligand-to-metal charge transfer in S-CoOOH catalyst, leading to formation of ONB states. The neighboring ONB states enable direct O-O coupling, namely coupled oxygen evolution mechanism, which achieves superior OER activity compared to the same catalysts under dark conditions, as well as R-CoOOH with weaker ligand-to-metal charge transfer properties. In my opinion, some experimental aspects of it are appealing, but significant concerns remain for the model construction, DFT computational details, and mechanistic discussion. These limitations compromise the study's conclusions. Therefore, I cannot recommend its publication in Nature Communications. Specific issues include:

1. Line 459, the authors claimed that the total of electrons in S-CoOOH supercell was adjusted to modify the oxidation state, please supplement the details and state the oxidation state of Co sites in optimized model.

Response: We sincerely appreciate the referee's important suggestion. According to the reviewer's advice, we have added the relevant description to the Methods section in the revised manuscript, as follows: In this case, we changed the total number of electrons in the system by setting NELECT in INCAR. Such as, we add one more electron into the supercell model for 7-electron O.

To address the comment, we have incorporated the proposed modifications into the Methods section of our manuscript (lines 501-503, page 20, highlighted in yellow): In this case, we changed the total number of electrons in the system by setting NELECT in INCAR. Such as, we add one more electron into the supercell model for 7-electron O.

2. Line 222, the authors claimed that S-CoOOH model feature 4-coordinated Co at edge and 6-coordinated Co in the bulk. Please highlight the locations of 4 and 6-coordinated Co in Figure S21.

Response: We sincerely appreciate the referee's insightful suggestion. According to the reviewer's advice, we have now clearly labeled the Co atom with 4- and 6-fold coordination in the structural model shown in the revised supplementary Fig.S29 (previously Fig.S21), with 4-coordinate Co atoms at the edge of CoOOH shown in blue, and 6-coordinated ones in the bulk shown in grey.

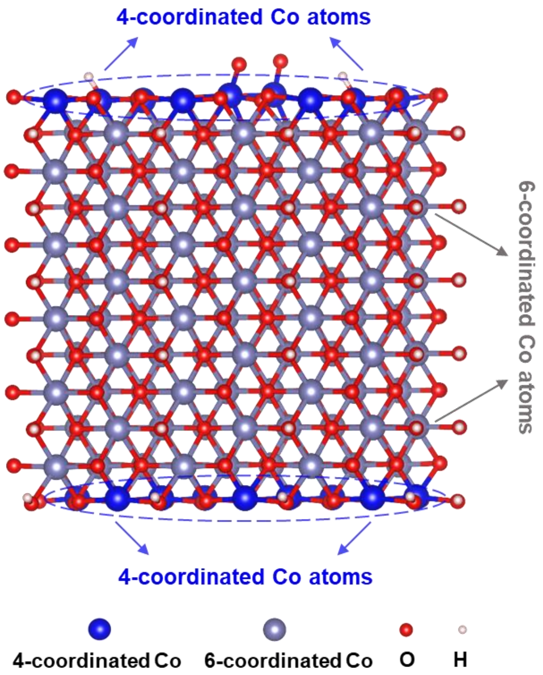

Fig.R3-1. The optimized model of S-CoOOH, with 4-coordinated Co atoms in blue and 6-coordinated ones in grey. (Supplementary Fig.S21 in our previous Supplementary Information, Fig.S29 in our revised version)

To address the comment, we have replaced the original Supplementary Fig.S21 (Fig.S29 in the revised version) with an updated one:

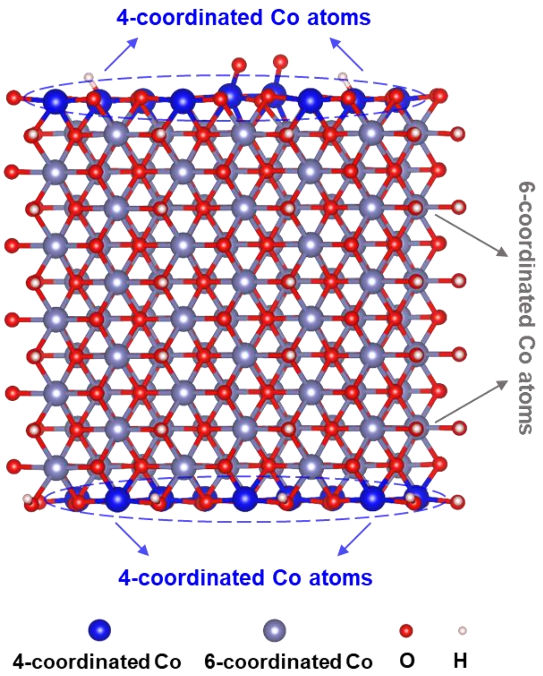

Supplementary Figure S29. The optimized model of S-CoOOH, with 4-coordinated Co atoms in blue and 6-coordinated ones in grey.

3. The structural model in Figure S21 is hard to read. It seems the authors introduced several random protons on surface to model the S-CoOOH catalyst, right? Please supplement which oxygen atoms were terminated by protons, and why this.

Response: We sincerely apologize for any potential misunderstanding that may have arisen from the original view direction of the structural model in the previous Figure S21. We would like to clarify that in our work, we did not introduce random protons on the surface to model S-CoOOH, nor were any oxygen atoms terminated by additional protons. All protons originate from the structure of CoOOH itself and follow a periodic arrangement. We have now provided a top-view model that better illustrates this configuration shown below (Fig.R3-2). We believe that the additional view offers a clearer visualization and can address the reviewer's concern regarding the construction of our S-CoOOH model.

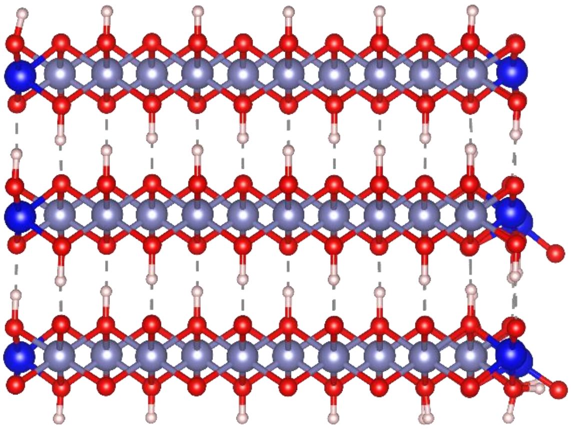

Fig.R3-2. Top view of the optimized S-CoOOH model, with 4-coordinated Co atoms in blue and 6-coordinated ones in grey. (Supplementary Fig.S30 in our revised Supplementary Information)

To address the comment, we have added a top view of our optimized S-CoOOH model to our Supplementary Information as Supplementary Fig.S30:

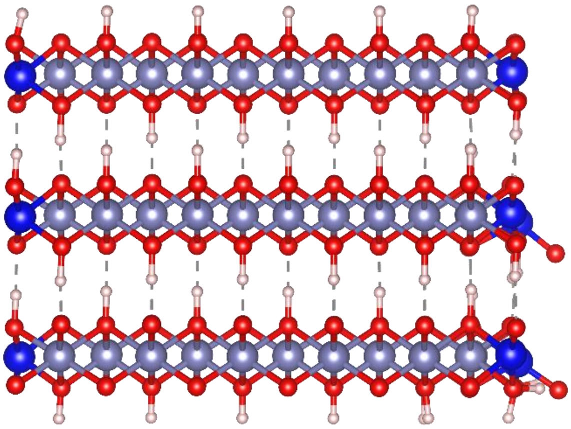

Supplementary Figure S30. Top view of the optimized S-CoOOH model, with 4-coordinated Co atoms in blue and 6-coordinated ones in grey.

4. All the calculations seem to be conducted in vacuum conditions. It is well established that the solvent environment plays an important role in electronic catalysis, especially considering there are many proton transfer steps in OER process. The authors are encouraged to consider the solvent conditions or at least need to rule out the effect on DFT conclusions.

Response: We are very grateful to the reviewer's insightful suggestion. In our previous work (Figure R47 in peer review file of *Nature* 2022, 611, 702-708), we have compared the free energy diagrams calculated without (GGA) and with solvent effects (solvent), as shown below (Fig.R3-3), and found negligible differences. In addition, neglecting solvent effects is consistent with the standard practice widely used in most DFT calculations on OER studies, as it significantly improves computational efficiency without impacting accuracy (*Science* 2025, 388, 430-436; *Science* 2025, 387, 791-796; *Science* 2025, 387, 48-55; *Nat. Catal.* 2025, 8, 523-535). Based on these considerations, the solvent conditions were not considered in our work.

[FIGURE REDACTED]

Fig.R3-3. Calculated reaction free energy change of the LOM route. (from our previous work in *Nature* 2022, 611, 702-708)

5. Line 455. CI-NEB method was used to locate the transition state of O-O coupling. Were the transition states confirmed by imaginary vibrational frequency analysis?

Response: We sincerely appreciate the reviewer's valuable suggestion. According to the reviewer's advice, we have added the results of the vibrational frequency analysis to confirm the identified transition states. The calculated results are shown below; it can be seen that only one imaginary frequency, further confirming the saddle point. We have added the results into the revised supplementary information and below description has also been added in the revised manuscript.

"We performed a vibrational frequency calculation at this saddle point. The presence of a single imaginary frequency confirms that the structure is indeed a first-order saddle point."

1 f	17.522950 THz	110.099942 2PiTHz	584.502699 cm^{-1}	72.469066 meV
2 f	13.881877 THz	87.222407 2PiTHz	463.049580 cm^{-1}	57.410805 meV
3 f	12.784254 THz	80.325839 2PiTHz	426.436823 cm^{-1}	52.871404 meV
4 f	11.620772 THz	73.015461 2PiTHz	387.627216 cm^{-1}	48.059628 meV
5 f	10.894075 THz	68.449493 2PiTHz	363.387230 cm^{-1}	45.054254 meV
6 f	10.142644 THz	63.728109 2PiTHz	338.322175 cm^{-1}	41.946585 meV
7 f	9.201811 THz	57.816682 2PiTHz	306.939367 cm^{-1}	38.055614 meV
8 f	8.203018 THz	51.541085 2PiTHz	273.623241 cm^{-1}	33.924943 meV
9 f	7.463638 THz	46.895418 2PiTHz	248.960154 cm^{-1}	30.867111 meV

10 f	5.787387 THz	36.363224 2PiTHz	193.046445 cm^{-1}	23.934698 meV
11 f	5.702479 THz	35.829732 2PiTHz	190.214225 cm^{-1}	23.583548 meV
**12 f/i=**	**11.818344 THz**	**74.256848 2PiTHz**	**394.217535 cm^{-1}**	**48.876723 meV**

To address the comment, we have incorporated the proposed modifications into our manuscript (lines 494-496, page 19, highlighted in yellow): We performed a vibrational frequency calculation at this saddle point. The presence of a single imaginary frequency confirms that the structure is indeed a first-order saddle point (Supplementary Table S4).

Supplementary Table S4. The results of vibrational frequency analysis for identifying transition states.

1 f	17.522950 THz	110.099942 2PiTHz	584.502699 cm^{-1}	72.469066 meV
2 f	13.881877 THz	87.222407 2PiTHz	463.049580 cm^{-1}	57.410805 meV
3 f	12.784254 THz	80.325839 2PiTHz	426.436823 cm^{-1}	52.871404 meV
4 f	11.620772 THz	73.015461 2PiTHz	387.627216 cm^{-1}	48.059628 meV
5 f	10.894075 THz	68.449493 2PiTHz	363.387230 cm^{-1}	45.054254 meV
6 f	10.142644 THz	63.728109 2PiTHz	338.322175 cm^{-1}	41.946585 meV
7 f	9.201811 THz	57.816682 2PiTHz	306.939367 cm^{-1}	38.055614 meV
8 f	8.203018 THz	51.541085 2PiTHz	273.623241 cm^{-1}	33.924943 meV
9 f	7.463638 THz	46.895418 2PiTHz	248.960154 cm^{-1}	30.867111 meV
10 f	5.787387 THz	36.363224 2PiTHz	193.046445 cm^{-1}	23.934698 meV
11 f	5.702479 THz	35.829732 2PiTHz	190.214225 cm^{-1}	23.583548 meV
**12 f/i=**	**11.818344 THz**	**74.256848 2PiTHz**	**394.217535 cm^{-1}**	**48.876723 meV**

6. Figure 5c. The authors performed density of state analysis for R-CoOOH catalyst. But I cannot find the descriptions for constructing this model.

Response: We are very grateful for the reviewer's important suggestion. R-CoOOH refers to the reference CoOOH, representing the standard bulk structure. According to the reviewer's advice, we have added the construction process of R-CoOOH model to Methods section in our revised manuscript as follows: For R-CoOOH model construction, first the standard CoOOH structure adopted from the experimental result underwent an optimization process. And then the optimized model was used to calculate the density of states.

To address the comment, we have incorporated the proposed modifications into the Methods section of our manuscript (lines 498-500, pages 19-20, highlighted in yellow): For R-CoOOH model construction, first the standard CoOOH structure adopted from the experimental result underwent an optimization process. And then the optimized model was used to calculate the density of states.

7. Figure 4b only considered the O-O coupling step at edge or bulk sites. But in Line 250, the authors claimed that the *OOH (i.e., O2- adsorbs OH-) formation is the rate-determining step. The effect of edge and bulk O sites on other OER elementary steps, such as OH- adsorption, O release, need to be considered. Moreover, the calculations seem to be on an assumption that the OER on ONB sites is more favorable than that on metal sites. To clarify this, the authors need to perform DFT calculations about OER reaction on cobalt sites of S-CoOOH model.

Response: We sincerely appreciate the reviewer's valuable comments. We agree that if Pathways 1-4 were all considered feasible, our simulations should be necessary to evaluate the other three elementary steps, such as OH^- adsorption and O_2 release. For O-O coupling to be accessible, two criteria must be met: First, as a spontaneous chemical process, it must be exothermic, meaning the energy of the initial state (reaction coordination 0 in Fig.R3-4) must be higher than that of the final state (reaction coordination 7) (*Nat. energy*, 2019, 4, 329-338). Second, the surmountable barrier for O-O coupling is 0.75 eV, therefore the activation energy must be lower than this value (*Chem. Rev.* 2019, 119, 7610-7672). As displayed in Fig.R3-4, Pathways 1-3 are all excluded because they are endothermic and have energy barriers exceeding 0.75 eV, whereas only Pathway 4 is likely to occur for O-O coupling. Hence, other steps on Pathways 1-3 were not conducted.

We also agree with the reviewer that *OOH formation occurring at cobalt site in adsorbate evolution mechanism (AEM) needs to be considered. However, *OOH formation is an electrochemical process that requires an applied potential to overcome its kinetic barrier, whereas O-O coupling is a spontaneous chemical process governed by thermodynamics. Their energy diagrams are therefore not directly comparable. Hence, the calculation on AEM process was not conducted in this work.

Meanwhile, we have conducted the calculations of *OH adsorption on both cobalt and O_{NB} sites. The results show that the adsorption on the O_{NB} site is more energetically favorable based on the calculated relative

energy. The optimized models of *OH adsorption at Co site (a) and O_{NB} site (b) are provided below (Fig.R3-5). The calculated relative energy for *OH adsorption at the O_{NB} site (−0.649 eV) is lower than that at the cobalt site (0 eV), indicating stronger adsorption at the O_{NB} site.

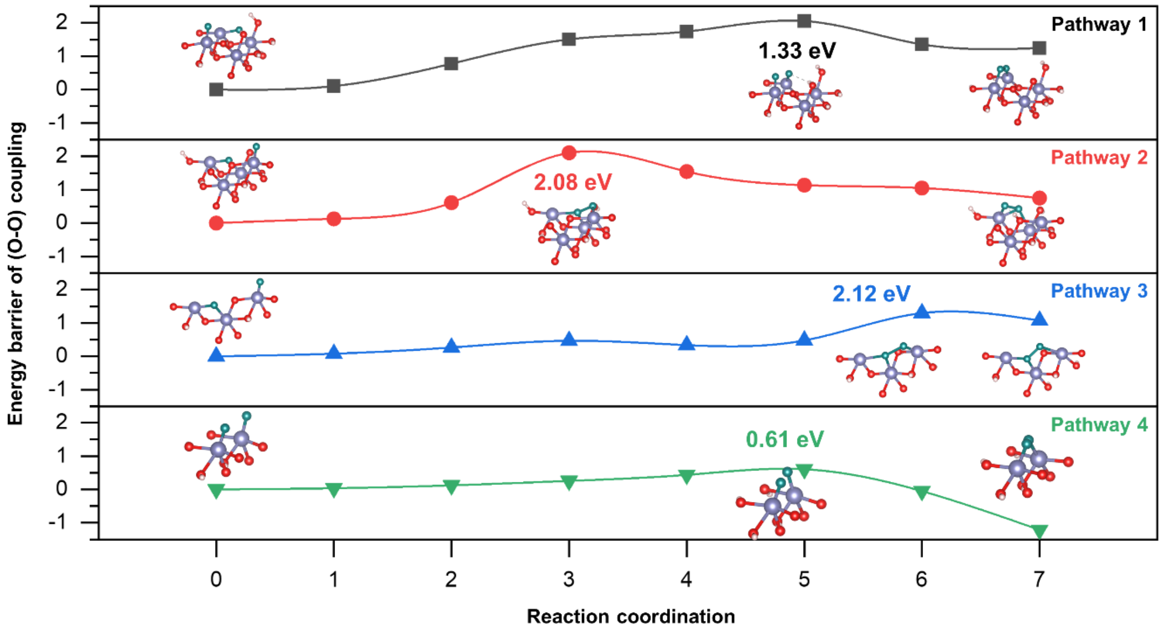

Fig.R3-4. Energy profile diagram of O-O coupling involving oxygen atoms located either in the bulk or at the edge. The inset shows the proposed O-O coupling occurring at different oxygen sites, referred to as Pathway 1-4, where oxygen atoms marked in green represent the atoms involved in O-O coupling. (Fig.4b in our previous manuscript, Fig.4b in our revised manuscript)

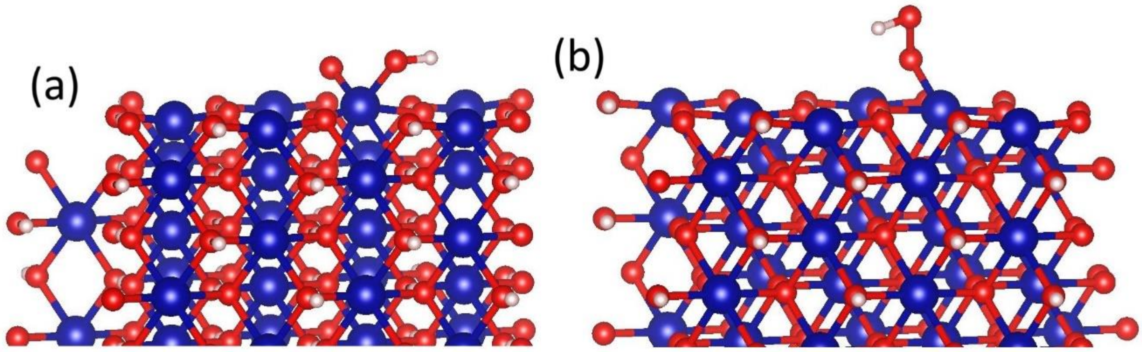

Fig.R3-5. The optimized models of *OH adsorption at Co site (**a**) and O_{NB} site (**b**).

(Supplementary Fig.S31 in our revised Supplementary Information)

To address the comment, we have incorporated the proposed modifications into our manuscript (lines 243-247, page 10, highlighted in yellow): In addition, calculations of $^{*}OH$ adsorption on cobalt and O_{NB} sites reveal that, the adsorption on the O_{NB} site (-0.649 eV) is more energetically favorable than on cobalt site (0 eV) based on the calculated relative energy. The optimized models of $^{*}OH$ adsorption at Co site (a) and O_{NB} site (b) are provided in Supplementary Fig.S31.

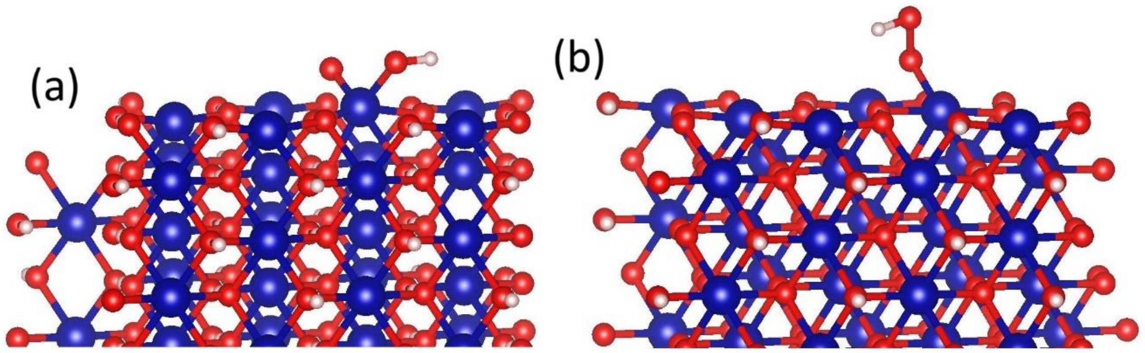

Supplementary Figure S31. The optimized models of $^{*}OH$ adsorption at Co site (**a**) and O_{NB} site (**b**).

Response to referees

We thank the three referees for taking the time to carefully review the manuscript and for giving many useful suggestions. The manuscript has been revised according to their comments and the changes have been highlighted in the revised version. We feel the quality of the paper has been improved greatly thanks to the input from the referees. Below is a point-by-point response.

Reviewer #1

Remarks to the Author: Some problems have been addressed, but many points still need to be further clarified.

1. In fact, the current density at many catalyst materials can be enhanced, which is completely not related with their reaction kinetics of OER. This may be induced by the photocurrent, because this light irradiation may directly enhance the conductivity of your catalysts without any involvements of electrochemical reaction when applying a special voltage. By the way, what is your working cell voltage?

Response: According to the reviewer's comments, we have conducted the conductivity measurements. The potentiostatic electrochemical impedance spectroscopy (PEIS) results collected at different applied voltages (1.35 V, 1.40 V, 1.45 V, and 1.50 V vs. RHE) show no noticeable changes between dark and light conditions (Fig.R1-1), indicating that the conductivity of catalyst is not improved under light.

Moreover, the observed current enhancement does not originate from conventional photocurrent. If the current enhancement is caused by photocurrent, the current response upon light on/off would be expected to exhibit an abrupt, step-like change, reflecting the instantaneous generation of free carriers and the corresponding increase in conductivity. In contrast, our measurements show a gradual increase when the illumination is switched on or off (Fig.R1-2). Such a slow and continuous evolution is incompatible with photocurrent-driven effect.

In addition, to further verify other possibility of photocurrent-related contributions, such as photocatalytic activities, in our work standard photocatalytic oxygen evolution measurements were conducted using $K_2S_2O_8$ as sacrificial electron acceptors under AM 1.5 G illumination, which were included in our previous

Supplementary Information. The potential of holes was assessed using gas chromatography, and no oxygen was detected over a 4-hour light irradiation test (Fig.R1-3), indicating that no light-induced holes with sufficient oxidative capability were generated. This outcome thus rules out any photocurrent-related contribution to the observed improvement in current density of S-CoOOH.

The working cell voltage is set above the OER onset potential, which is approximately 1.35 V vs. RHE for our sample S-CoOOH.

We shall state that the increase in current density is related to the change in OER reaction mechanism. *Operando* X-ray absorption spectroscopy (XAS), differential electrochemical mass spectrometry (DEMS) coupled with ^{18}O isotope labeling, and TMAOH chemical probe results collectively demonstrate that light irradiation induces electron transfer from oxygen to cobalt, leading to a reduction in cobalt valence and formation of oxidized oxygen species (non-bonding oxygen O_{NB}). Then, these two neighboring O_{NB} states couple to form direct O-O coupling, thereby bypassing the rate-determining step of $^{*}OOH$ formation in dark metal-redox pathway. As such, light shifts the redox activity from metal to oxygen, which is the reason for the enhancement of catalytic activity. More detailed discussion of the experimental evidence is provided in the response to Question R1-3.

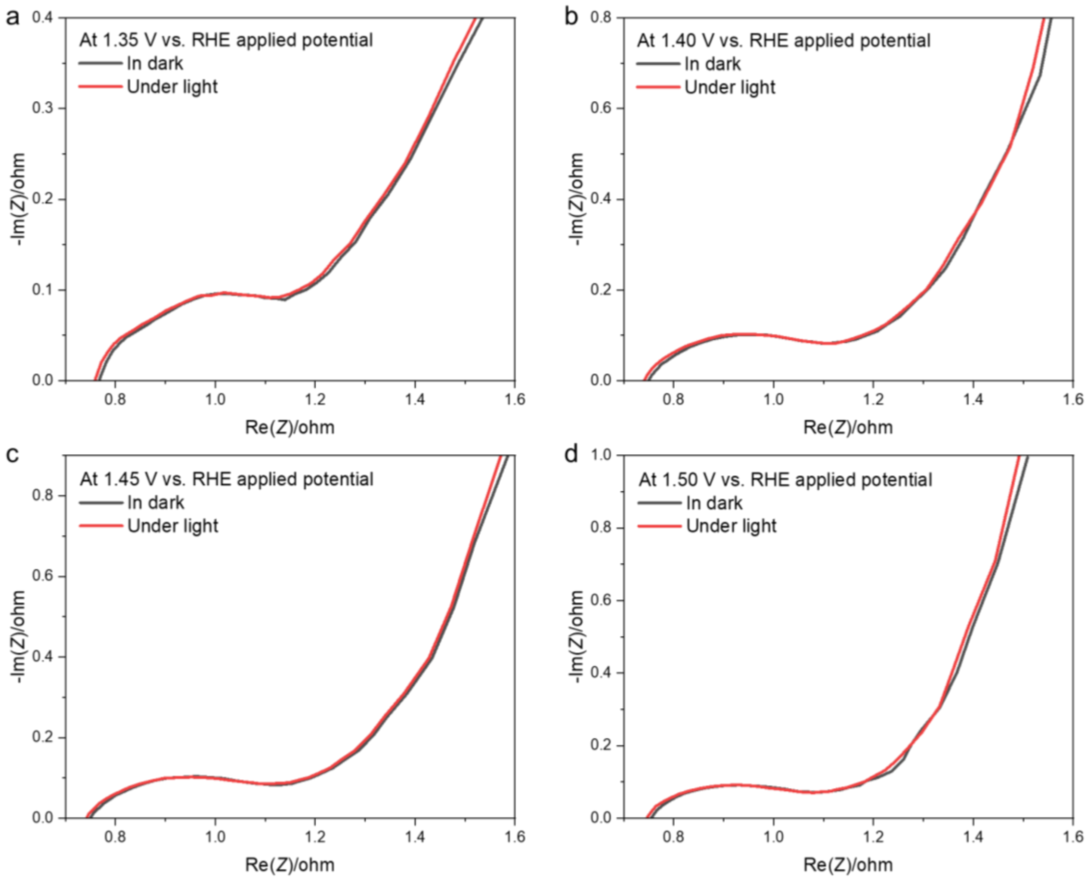

Fig.R1-1. Potentiostatic electrochemical impedance spectroscopy (PEIS) data for S-CoOOH in dark and under light, recorded at different applied potentials of 1.35 V (**a**), 1.40 V (**b**), 1.45 V (**c**), and 1.50 V (**d**) vs. RHE. (Supplementary Fig.S17 in our revised Supplementary Information)

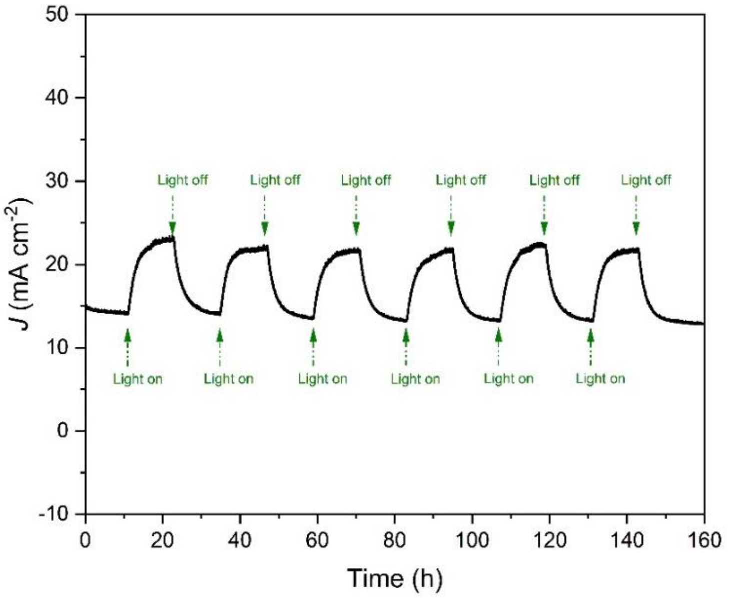

Fig.R1-2. The light-response repeatability experiments for electrochemical measurements of S-CoOOH.

Chronoamperometry (CA) curve for S-CoOOH at a constant potential of 1.45 V versus a reversible hydrogen electrode (RHE), with multiple cycles of turning light on and off. (Supplementary Fig.S3 in our previous Supplementary Information, Supplementary Fig.S3 in our revised Supplementary Information)

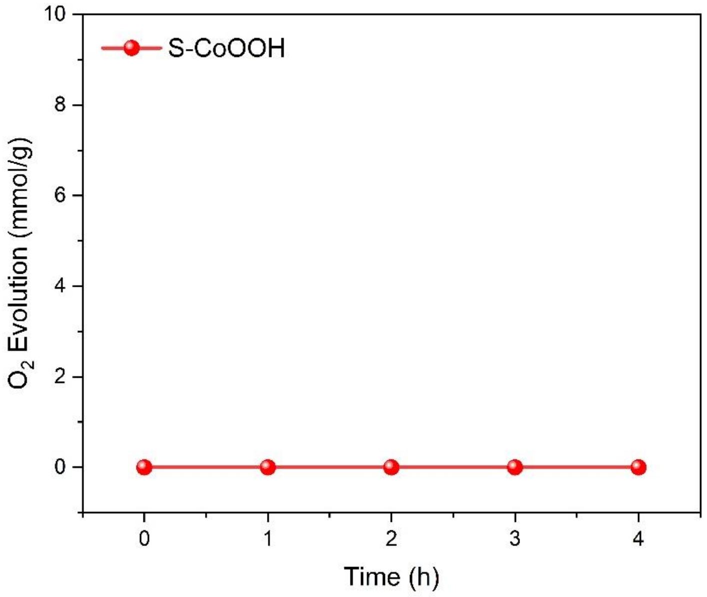

Fig.R1-3. Photocatalytic oxygen generation as a function of irradiation time. The gas chromatograph measurement of S-CoOOH, using $K_2S_2O_8$ as electron acceptor under AM 1.5G condition. (Supplementary Fig.S15 in our previous Supplementary Information, Supplementary Fig.S15 in our revised Supplementary Information)

To address the comment, we have incorporated the proposed modifications into our manuscript (lines 115-118, page 5, highlighted in yellow): Furthermore, the enhancement of conductivity induced by photocurrent is also ruled out based on potentiostatic electrochemical impedance spectroscopy (PEIS) results, showing no noticeable changes between dark and light conditions collected at different applied voltages (1.35 V, 1.40 V, 1.45 V, and 1.50 V vs. RHE) (Supplementary Fig.S17).

Moreover, we have revised the content accompanying Supplementary Fig.S17 in our supplementary information: As shown in Supplementary Figure S17, the potentiostatic electrochemical impedance spectroscopy (PEIS) results collected at different applied voltages (1.35 V, 1.40 V, 1.45 V, and 1.50 V vs. RHE) show no noticeable changes between dark and light conditions, indicating that the conductivity of

catalyst is not improved under light. This rules out the possibility of photocurrent-induced conductivity improvement. In addition, the above photocatalytic test (Supplementary Figure S15), which does not involve oxygen evolution, and light on/off current response curve (Supplementary Figure S3), which exhibits a gradual increase rather than an abrupt step-like increase, both further confirm that the observed current enhancement does not originate from a conventional photocurrent effect.

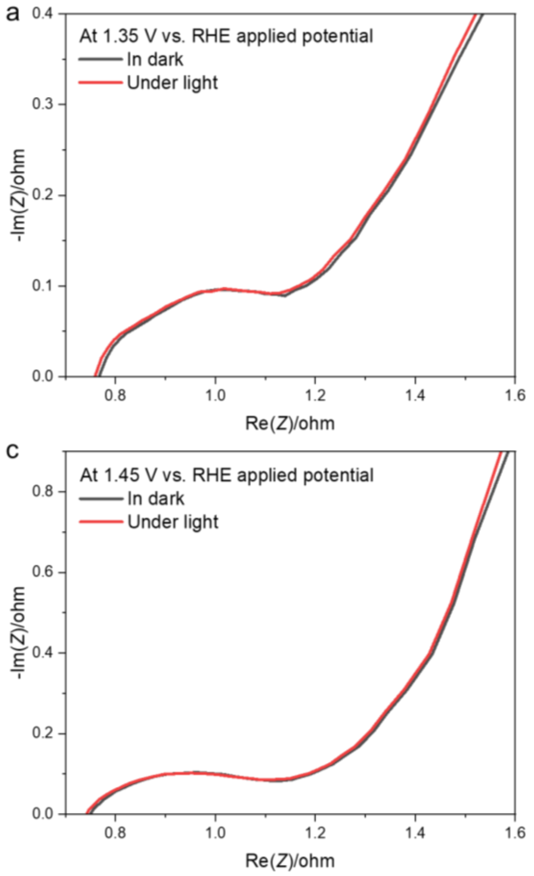

Supplementary Figure S17. Potentiostatic electrochemical impedance spectroscopy (PEIS) data for S-CoOOH in dark and under light, recorded at different applied potentials of 1.35 V (**a**), 1.40 V (**b**), 1.45 V (**c**), and 1.50 V (**d**) vs. RHE.

In addition, we have clarified in the manuscript that the increase in current density is attributed to a change in OER reaction pathway, rather than to a photocurrent effect (lines 291-297, page 12, highlighted in yellow): Briefly, the mechanism of light-triggered catalytic enhancement in S-CoOOH is related to the change in OER reaction mechanism, illustrated in Fig.4c,d. Light irradiation triggers electron transfer from oxygen (ligand) to cobalt (metal) in S-CoOOH, leading to a reduction in cobalt valence and the formation of O_{NB} states; then, two neighboring O_{NB} states couple to form direct O-O coupling, thereby bypassing the rate-

determining step of $^*OOH$ formation in dark metal-redox pathway. As such, light shifts the redox activity from metal to oxygen, which is the reason for the enhancement of catalytic activity.

2. Why the charge transfer from oxygen to cobalt can occurs? Just light irradiation? What is the driving force? Transient absorption spectra should be provided to unveil this important process of light-induced carrier kinetic.

Response: In our work, the charge transfer from oxygen to cobalt in S-CoOOH is triggered solely by light irradiation. The electronic structure of S-CoOOH showcases a distinct non-overlapping region between Co 3*d* and 4*s* orbitals around the Fermi level, providing available unoccupied states that can accommodate excited electrons (Fig.R1-4). Upon illumination, photons provide the required energy to promote electron transfer from oxygen 2*p* to cobalt 3*d* orbitals, generating non-bonding oxygen (O_{NB}) states and a decrease in Co valence. Then, these unstable O_{NB} species couple to form direct O-O coupling that stabilizes the photoinduced intermediates. In this way, the light-induced electron transfer process in our sample S-CoOOH is feasible and coupled to the OER processes (as marked in State 3, Fig.R1-5).

At first, our experiments demonstrate that the adsorbed photon energy is sufficient to drive this process. The UV-Vis absorption spectrum (Fig.R1-6) shows that S-CoOOH exhibits intense light absorption over 200-2400 nm (0.52-6.20 eV). Combined DFT calculations and photon response measurements (Fig.R1-7 and Fig.R1-8), photons with energies of over 1.03 eV (λ <1200 nm) are required to activate LMCT in S-CoOOH. Therefore, under AM 1.5G light irradiation, the absorbed photons provide sufficient energy to promote the LMCT process.

Then, to verify that light irradiation is the only driving force for this charge transfer process, we conducted a series of controlled comparative experiments under dark and light conditions, with light illumination as the sole variable. *Operando* Co *K*-edge XAS measurements of S-CoOOH during OER confirm that cobalt valence decreases exclusively upon light irradiation (Fig.R1-9). Furthermore, TMAOH probing experiments (Fig.R1-10 and Fig.R1-11) and *operando* differential electrochemical mass spectrometry (DEMS) coupled with ^{18}O isotope experiments (Fig.R1-12) all identify the formation of oxidized oxygen species only under

light. In contrast, neither reduction in cobalt valence nor formation of oxidized oxygen species occurs during otherwise identical dark OER conditions. These results collectively demonstrate that light irradiation is the sole driving force triggering electron transfer from oxygen to cobalt.

Moreover, our previous supplemental information has already excluded alternative contributions such as photothermal effect, grain growth, photocurrent, and changes in conductivity resistance. The only driving force is the energy of photons absorbed by S-CoOOH.

In addition, we have performed transient absorption (TA) spectroscopy of S-CoOOH using 700 nm as an excitation source (Fig.R1-13). No clear transient features are observed in the 2D TA map, except for a signal around 700 nm arising from excitation light scattering, which is consistent with our proposed mechanism. As discussed above, the light-induced electron transfer in our work is coupled to the OER processes and is regarded as a part of O-O coupling step, rather than a conventional photophysical excitation (Fig.R1-5).

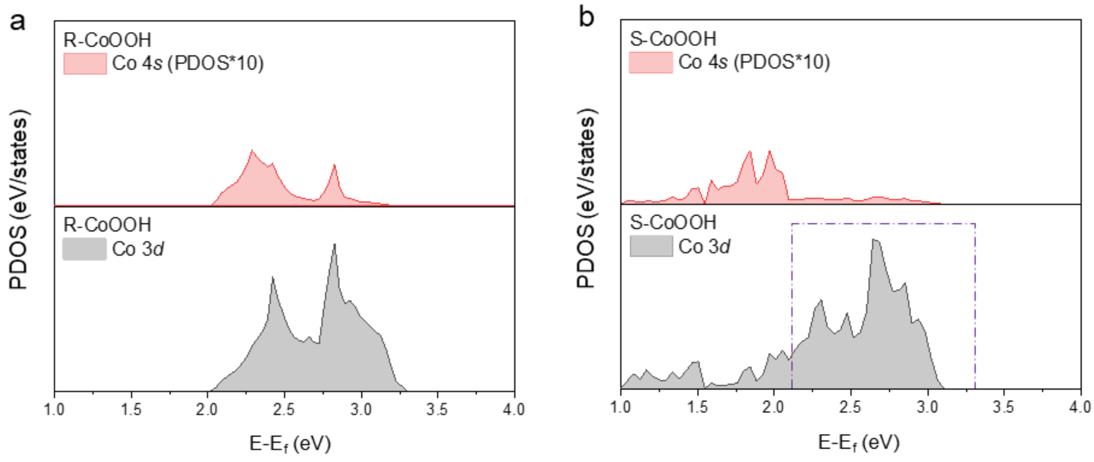

Fig.R1-4. PDOS of cobalt 3*d* and 4*s* orbitals in both R-CoOOH (**a**) and S-CoOOH (**b**). (Fig.5c,d in our previous manuscript, Fig.5c,d in our revised manuscript)

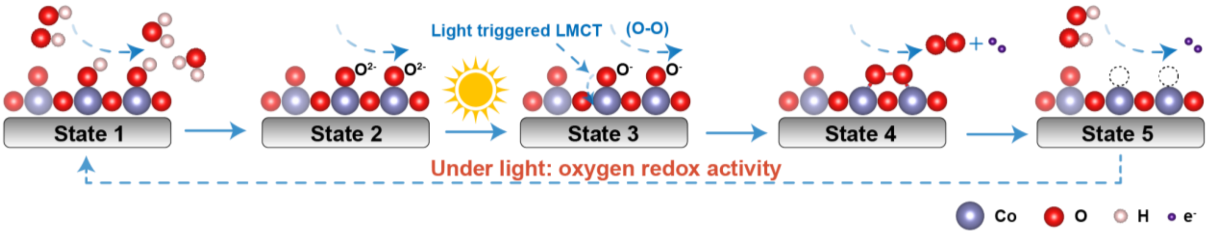

Fig.R1-5. Electron transfer pathway of S-CoOOH under light, with light-triggered electron transfer

occurring during O-O coupling (State 3). (Fig.4d in our previous manuscript, Fig.4d in our revised manuscript)

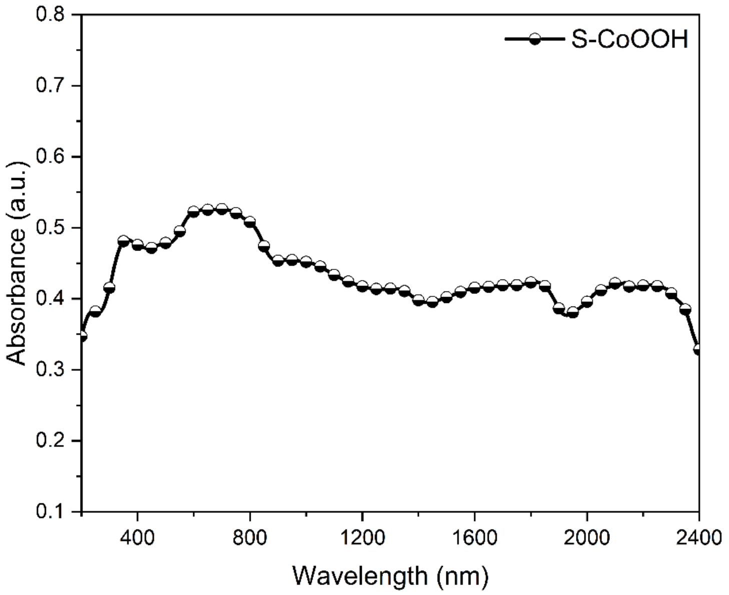

Fig.R1-6. UV-Vis absorption spectrum of S-CoOOH powder measured in the 200-2400 nm wavelength range. (Supplementary Fig.S33 in our previous Supplementary Information, Supplementary Fig.S39a in our revised Supplementary Information)

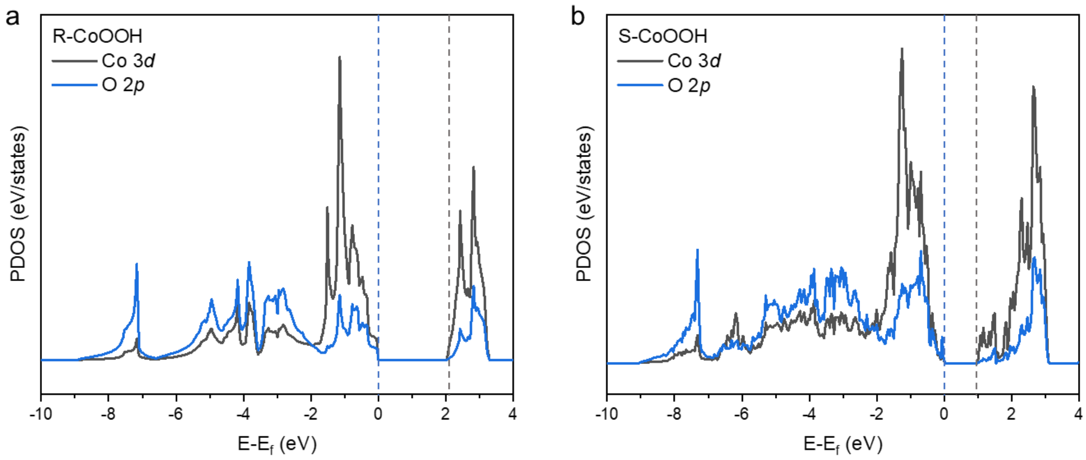

Fig.R1-7. PDOS of cobalt 3*d* and oxygen 2*p* orbitals in both R-CoOOH (**a**) and S-CoOOH (**b**). (Supplementary Fig.S32 in our previous Supplementary Information, Supplementary Fig.S38 in our revised Supplementary Information)

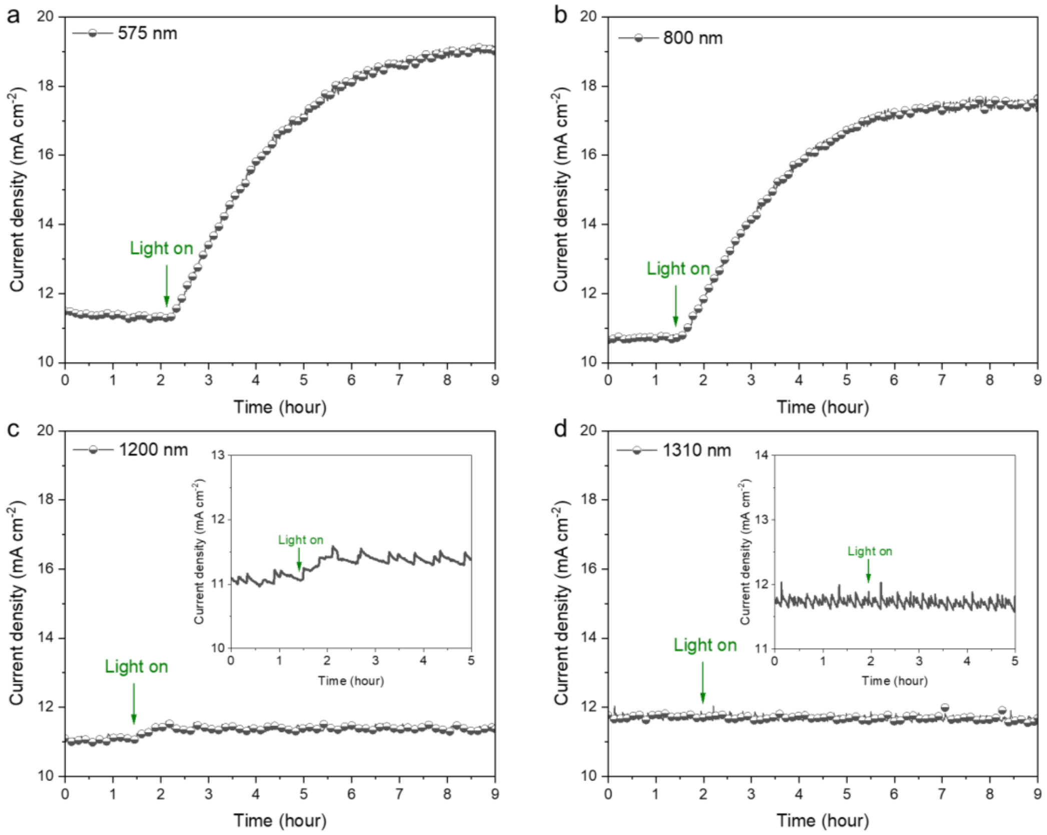

Fig.R1-8. Photon response measurements equipped with different wavelength filters of 575 nm (**a**), 800 nm (**b**), 1200 nm (**c**), and 1310 nm (**d**), respectively. (Supplementary Fig.S8 in our previous Supplementary Information, Supplementary Fig.S8 in our revised Supplementary Information)

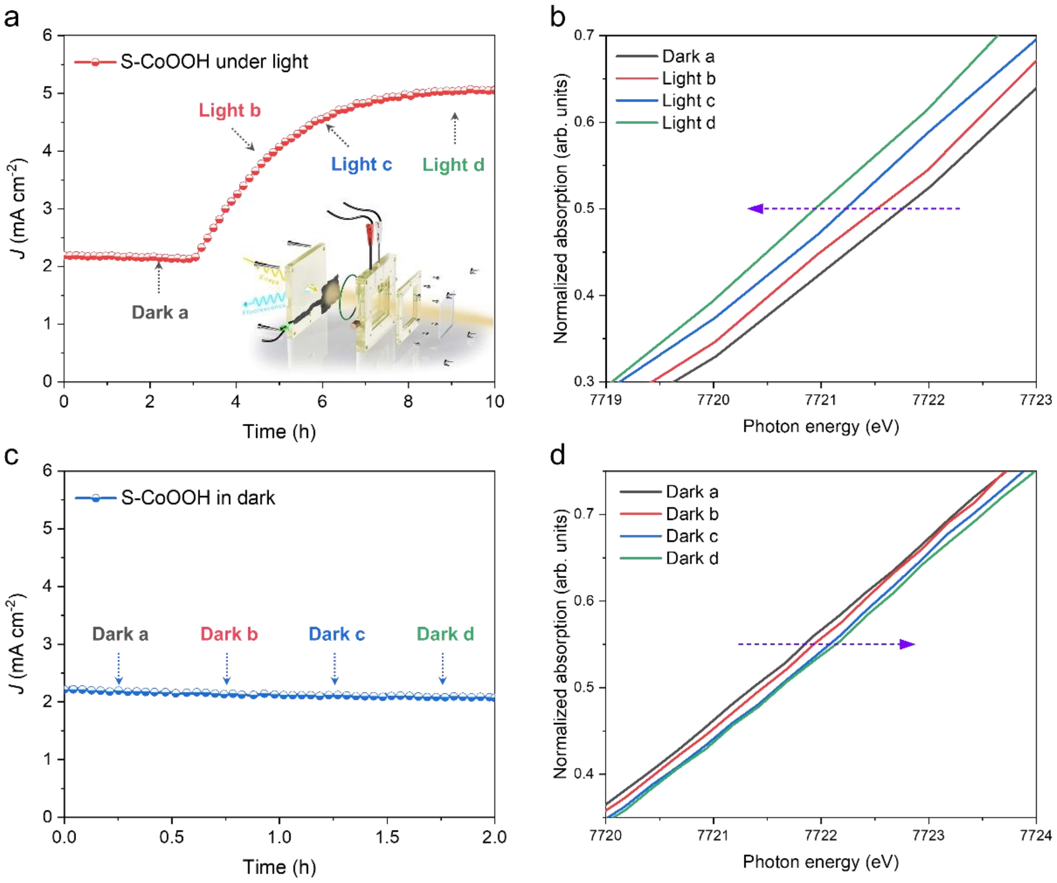

Fig.R1-9. *Operando* Co *K*-edge XAS measurements of S-CoOOH during OER under light and dark conditions. a Electrochemical curve for *operando* Co *K*-edge XAS measurements recorded during OER from dark to illumination at four selected points: Dark a, Light b, Light c, and Light d. **b** Enlarged *operando* Co *K*-edge XAS spectra of S-CoOOH during OER under light near the absorption edge, with dashed purple lines revealing spectral shifts. **c** Electrochemical curve for *operando* Co *K*-edge XAS measurements in the dark, recorded during the chronoamperometry (CA) process at four selected points: Dark a, Dark b, Dark c, and Dark d. **d** Enlarged *operando* Co *K*-edge XAS spectra of S-CoOOH during OER in dark near the absorption edge, with dashed purple lines indicating spectral shifts. (Fig.2a,c and Supplementary Figs.S19b, S20b in our previous manuscript and Supplementary Information, Fig.2a,c and Supplementary Figs.S23b, S24b in our revised manuscript and Supplementary Information)

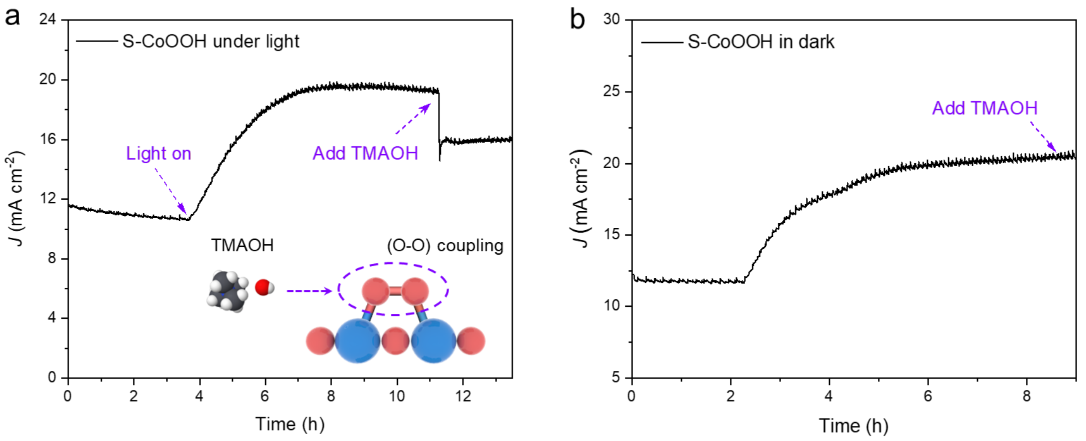

Fig.R1-10. The electrochemical curves respond to the addition of TMAOH under light (**a**) and in dark (**b**). (Fig.3a and Supplementary Fig.S23a in our previous manuscript and Supplementary Information, Fig.3a and Supplementary Fig.S28a in our revised manuscript and Supplementary Information)

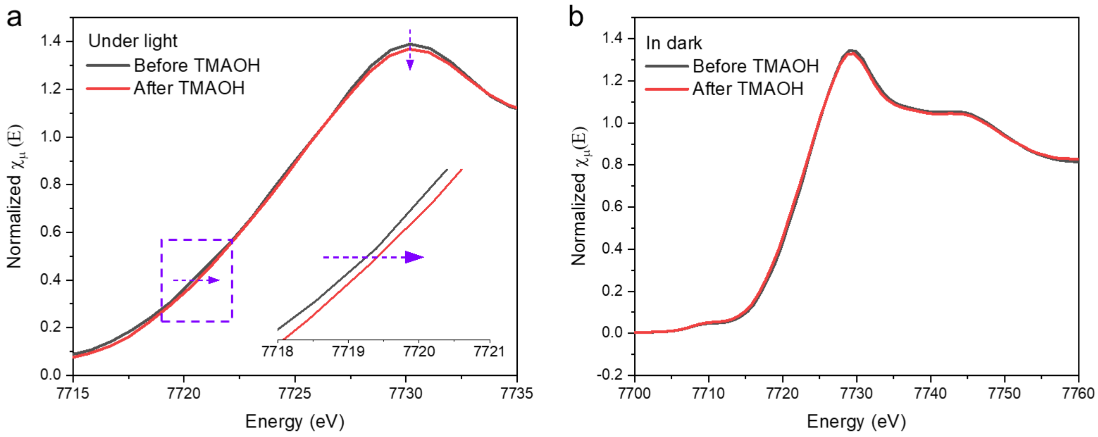

Fig.R1-11. Co *K*-edge XAS spectra of S-CoOOH recorded under light (**a**) and in dark (**b**) conditions, before and after TMAOH addition. (Fig.3b and Supplementary Fig.S23b in our previous manuscript and Supplementary Information, Fig.3b and Supplementary Fig.S28b in our revised manuscript and Supplementary Information)

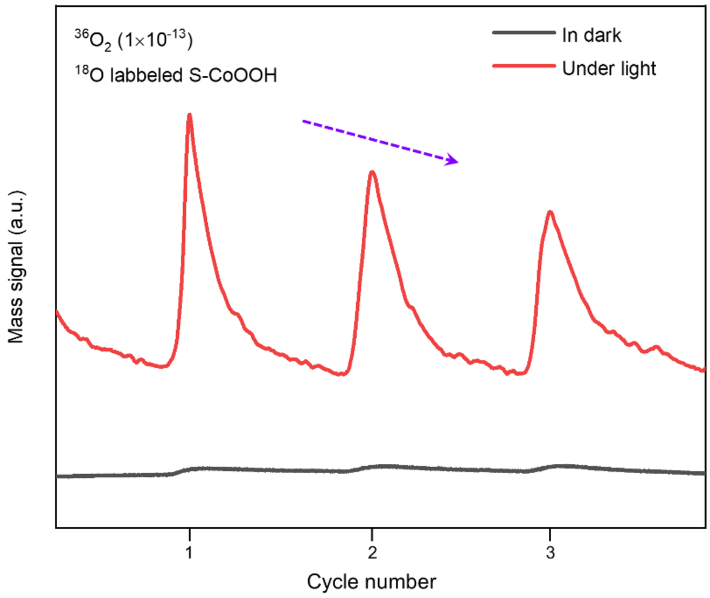

Fig.R1-12. Differential electrochemical mass spectrometry (DEMS) signals of O_2 products ($^{36}O_2$) for S-CoOOH measured with and without light irradiation. (Fig.4a in our previous manuscript, Fig.4a in our revised manuscript)

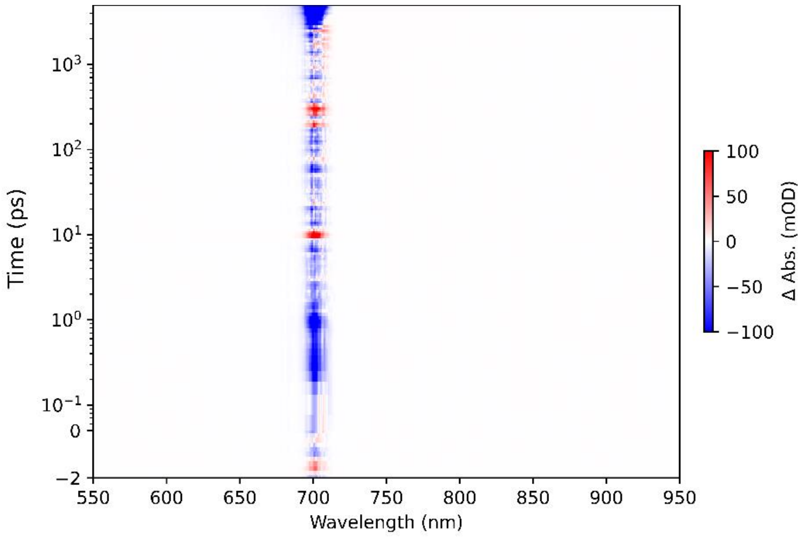

Fig.R1-13. 2D-false color plot of fs-transient absorption in 550-950 nm for S-CoOOH using 700 nm as an excitation source, with the signal around 700 nm arising from excitation light scattering. (Supplementary Fig.S25 in our revised manuscript and Supplementary Information)

To address the comment, we have incorporated the proposed modifications into our manuscript (lines 353-356, page 15, highlighted in yellow): The photophysical property of S-CoOOH was further examined by UV-Vis absorption and diffuse reflectance spectra (Supplementary Fig.S39a, b), revealing intense light

absorption over 200-2400 nm (0.52-6.20 eV), which provides the required energy to promote electron transfer from (M-O) to Co 3*d* orbitals (LMCT) in S-CoOOH.

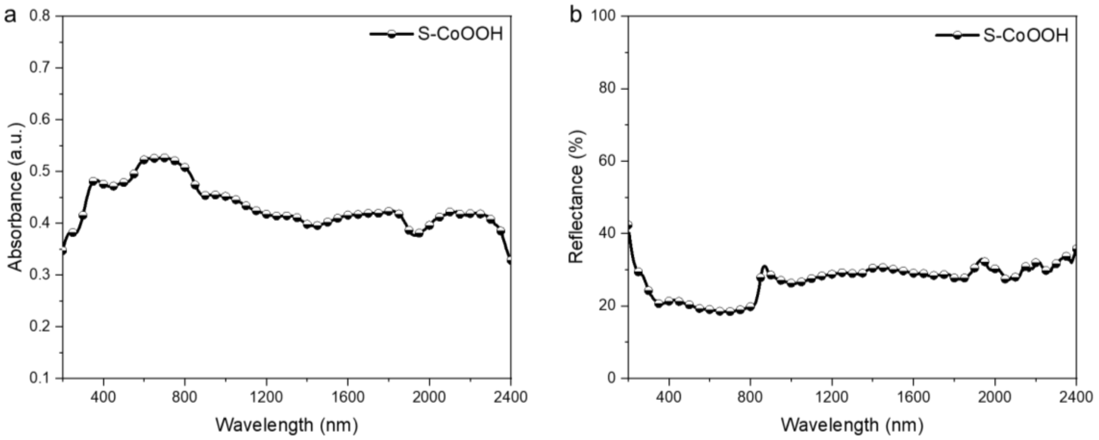

Supplementary Figure S39. UV-Vis absorption (**a**) and reflectance spectra (**b**) of S-CoOOH powder measured in the 200-2400 nm wavelength range.

Besides, we have revised the legends for Fig.5e and 5f as follows (lines 309-310, page 14, highlighted in yellow): **Fig.5e** Schematic diagram of infeasible electron transfer in R-CoOOH, with a complete overlapping region between Co 3*d* and 4*s* orbitals. **f** Schematic diagram of feasible electron transfer in S-CoOOH, with a distinct non-overlapping region between Co 3*d* and 4*s* orbitals.

Moreover, we have incorporated the proposed modifications into our manuscript (lines 270-274, page 12, highlighted in yellow): Based on the aforementioned *operando* XAS, *operando* DEMS, and TMAOH chemical probe experiments, the OER electron pathway of S-CoOOH shifts from metal redox activity in dark to oxygen redox activity under light, with neighboring O_{NB} states at its edge undergo O-O coupling. The above comparative experiments under both dark and light conditions confirm that light is the sole driving force for LMCT.

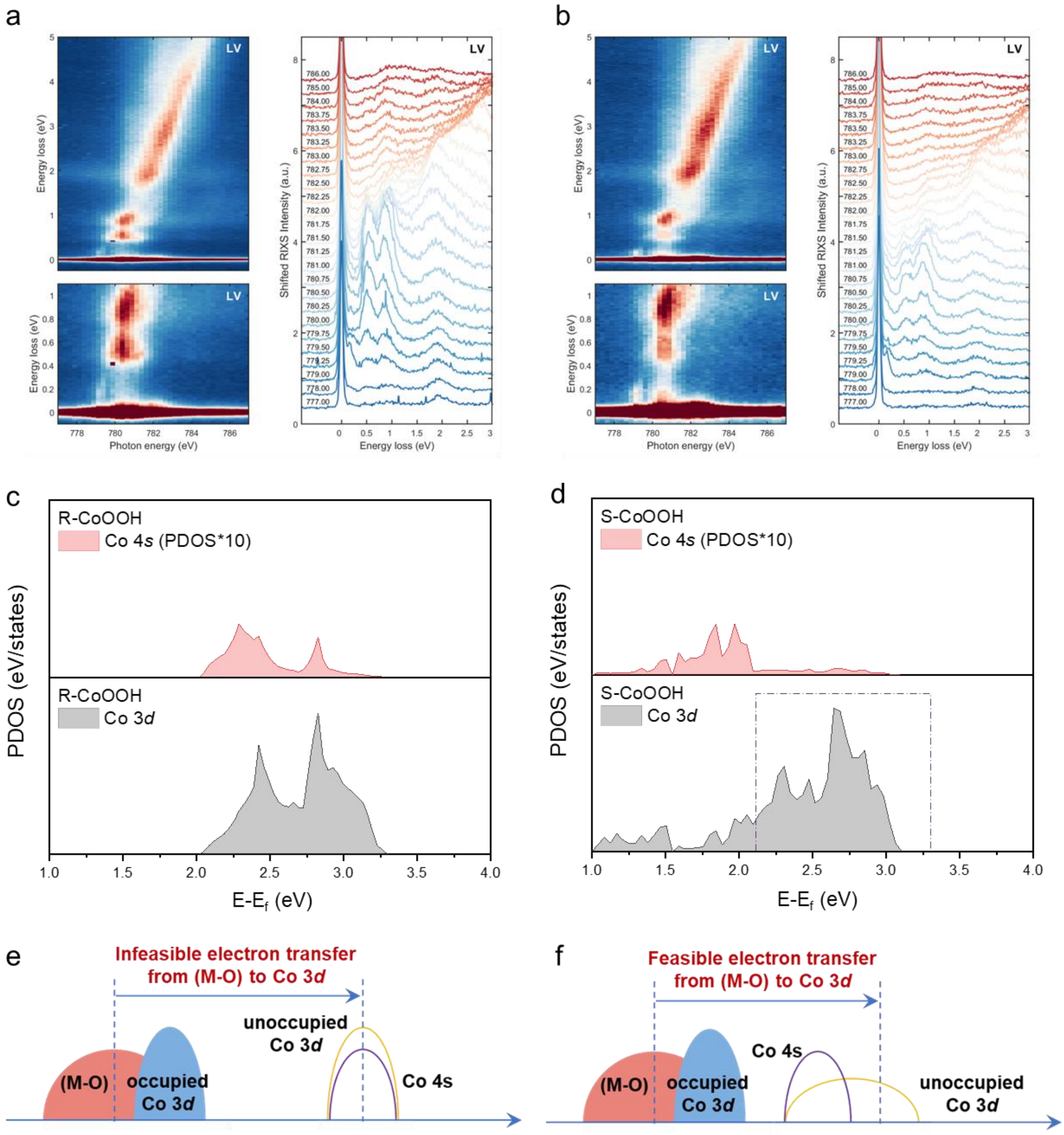

Figure 5. Differences in the electronic structures of R-CoOOH and S-CoOOH. **a** Co 2p3d RIXS spectra of R-CoOOH. **b** Co 2p3d RIXS spectra of S-CoOOH. **c** PDOS of cobalt 3*d* and 4*s* orbitals in R-CoOOH. **d** PDOS of cobalt 3*d* and 4*s* orbitals in S-CoOOH. **e** Schematic diagram of infeasible electron transfer in R-CoOOH, with a complete overlapping region between Co 3*d* and 4*s* orbitals. **f** Schematic diagram of feasible electron transfer in S-CoOOH, with a distinct non-overlapping region between Co 3*d* and 4*s* orbitals.

In addition, we have incorporated the proposed modifications into our manuscript (lines 154-159, page 6, highlighted in yellow) and added further explanation accompanying Supplementary Fig.S25: Importantly, the LMCT involved here is not a conventional photophysical LMCT, but rather a step of OER processes that

is coupled to chemical bond formation, and thus cannot be detected by transient absorption spectroscopy (Supplementary Fig.S25). Further evidence for LMCT is provided by combination of *operando* Co *K*-edge XAS and subsequent TMAOH chemical probe experiments, which together reveal a decrease of cobalt (electron transfer to metal) and the presence of O_{NB} species (electron transfer from ligand).

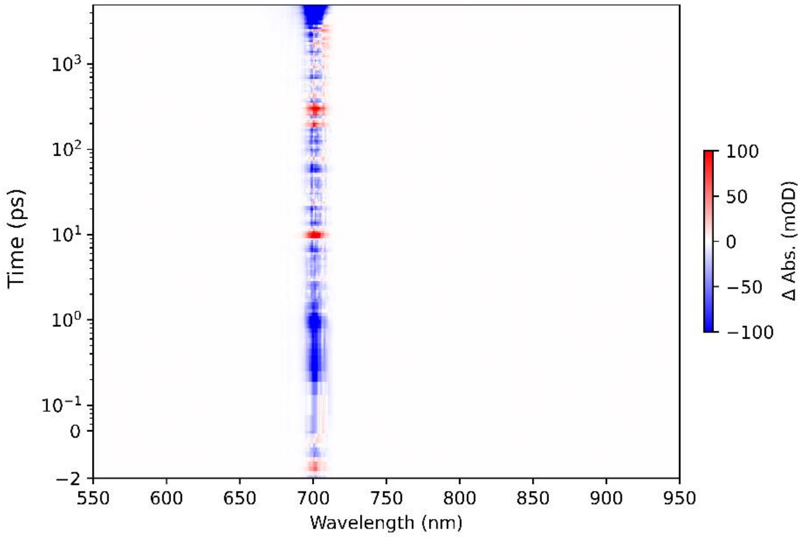

Supplementary Figure S25. 2D-false color plot of fs-transient absorption in 550-950 nm for S-CoOOH using 700 nm as an excitation source, with the signal around 700 nm arising from excitation light scattering.

The transient absorption (TA) spectroscopy of S-CoOOH was performed using 700 nm as an excitation source (Supplementary Figure S25). No clear transient features are observed in the 2D TA map, except for a signal around 700 nm arising from excitation light scattering. This is reasonable because the light-induced electron transfer in our system is not a conventional photophysical excitation. In our proposed mechanism, S-CoOOH first adsorbs OH^- and undergoes deprotonation to form the $^*O$ intermediate under applied potential. Light then acts on this deprotonated $^*O$ species, generating 7-electron oxygen ($^*O^-$). Two adjacent $^*O^-$ subsequently undergo direct O-O coupling, which stabilizes the photoinduced state. This light-induced electron transfer is coupled to the OER process and is regarded as a part of O-O coupling step. In the absence of bias, the deprotonated $^*O$ intermediate is not formed and no light-induced electron transfer is triggered, which explain the absence of observable TA signals.

3. The light adsorption response of different wavelength is directly related with their band gap, so the enhancement in OER current can be observed but it cannot directly confirm their intrinsic correlations to

change the reaction pathway (or activity) of OER.

Response: As discussed in response to Questions 1 and 2, in our work the observed photon response of different light wavelengths is not related to a conventional band gap excitation, but is instead associated with light-induced electron transfer from occupied (M-O) bond to unoccupied Co 3*d* orbitals (Fig.R1-14).

As illustrated in the PDOS of cobalt 3*d* and oxygen 2*p* orbitals, the energy barrier between (M-O) to Co 3*d* orbital in S-CoOOH is 0.98 eV, corresponding to a light wavelength of about 1260 nm (Fig.R1-15). Consistent with this prediction, photon response measurements under varying illumination wavelengths show good agreement with the calculated energy threshold (Fig.R1-16). Illumination at 575 nm and 800 nm result in a pronounced increase in current (Fig.R1-16a and b). In contrast, only a minimal current increase is detected at 1200 nm (1.03 eV), while no observable response is observed at 1310 nm (0.95 eV) (Fig.R1-16c and d), suggesting that a minimum photon energy of approximately 0.95 eV ($\lambda < 1310$ nm) is required to activate such electron transfer. **These photon response of different light wavelengths experimental results agree well with our calculations, which provide strong validation that photon response measurements under varying illumination wavelengths in our work are closely associated with electron transfer from (M-O) bond to Co 3*d* orbitals.**

Moreover, a change in reaction pathway of OER was verified using *operando* X-ray absorption spectroscopy (XAS), *operando* Differential electrochemical mass spectrometry (DEMS) coupled with ^{18}O isotope labeling, and TMAOH chemical probe measurements.

a) *Operando* DEMS coupled with ^{18}O isotope labeling (Fig.R1-17).

This approach provides direct evidence for the origin of the reaction product O_2 and reveals a light-induced change in reaction pathway. A clear $^{36}O_2$ signal is only detected under light and gradually decreases over time, indicating that oxygen redox activity takes place at the edge of S-CoOOH. In contrast, no $^{36}O_2$ signal is observed in dark, reflecting that S-CoOOH follows metal redox pathway in dark.

b) *Operando* Co *K*-edge XAS measurements (Fig.R1-18).

Under light irradiation, the absorption edge (7718 to 7725 eV) exhibits a redshift compared with that in dark, and this redshift intensifies with prolonged light exposure. Such a redshift in photon energy reflects

a reduction in Co valence states. In contrast, *operando* XAS measurement conducted under otherwise identical OER conditions in dark exhibits a gradual blueshift in absorption edge, consistent with the conventional Co^{3+}/Co^{4+} transition.

c) TMA^{+} chemical probe experiments (Fig.R1-19 and Fig.R1-20).

Both TMA^{+} chemical probe experiments and TMA^{+} probe measurements combined with Co *K*-edge XAS confirm the generation of oxidized oxygen species under light, which tend to undergo direct O-O coupling. In contrast, in the absence of oxidized oxygen species in dark, OER proceeds via the conventional pathway, in which two adjacent absorbed $^{*}OH$ undergo deprotonation to form $^{*}OOH$ intermediate.

Taken together, *operando* XAS, DEMS, and TMAOH results demonstrate that light irradiation induces electron transfer from oxygen to cobalt, leading to a reduction in cobalt valence and formation of oxidized oxygen species. These light-induced oxidized oxygen species undergo direct O-O coupling, thereby bypassing the rate-determining step $^{*}OOH$ formation in dark OER. As a result, light irradiation alters the OER reaction pathway from metal-centered redox in dark to edge oxygen-centered redox under light, as summarized in Fig.R1-21.

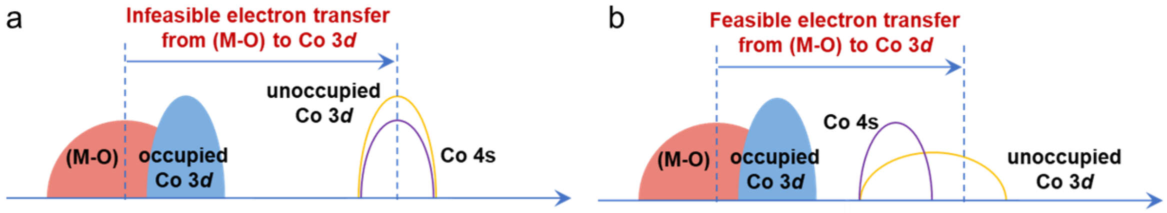

Fig.R1-14. Schematic diagram of light-induced electron transition from (M-O) bond to Co 3*d* orbitals: **a** infeasible electron transfer in R-CoOOH, with a complete overlapping region between Co 3*d* and 4*s* orbitals. **b** feasible electron transfer in S-CoOOH, with a distinct non-overlapping region between Co 3*d* and 4*s* orbitals. (Fig.5e,f in our previous manuscript, Fig.5e,f in our revised manuscript)

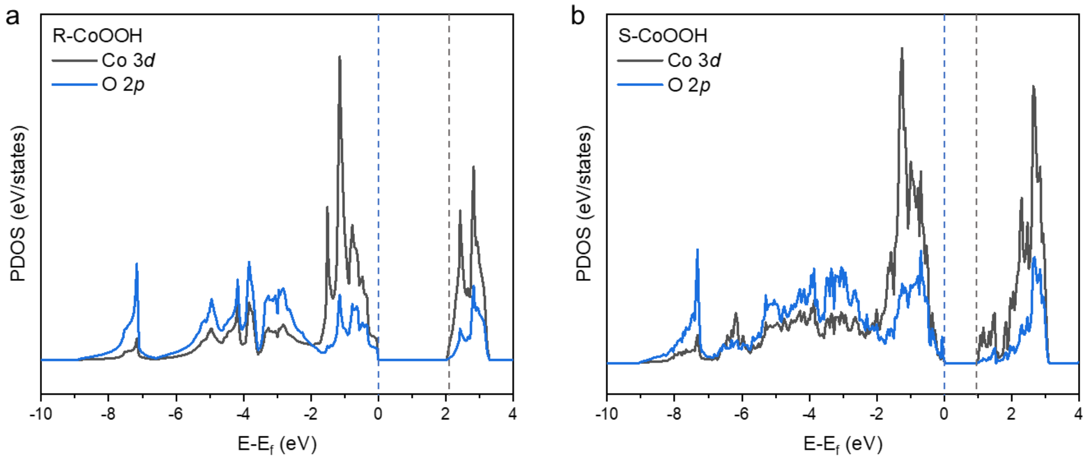

Fig.R1-15. PDOS of cobalt 3*d* and oxygen 2*p* orbitals in both R-CoOOH (**a**) and S-CoOOH (**b**). (Supplementary Fig.S32 in our previous Supplementary Information, Supplementary Fig.S38 in our revised Supplementary Information)

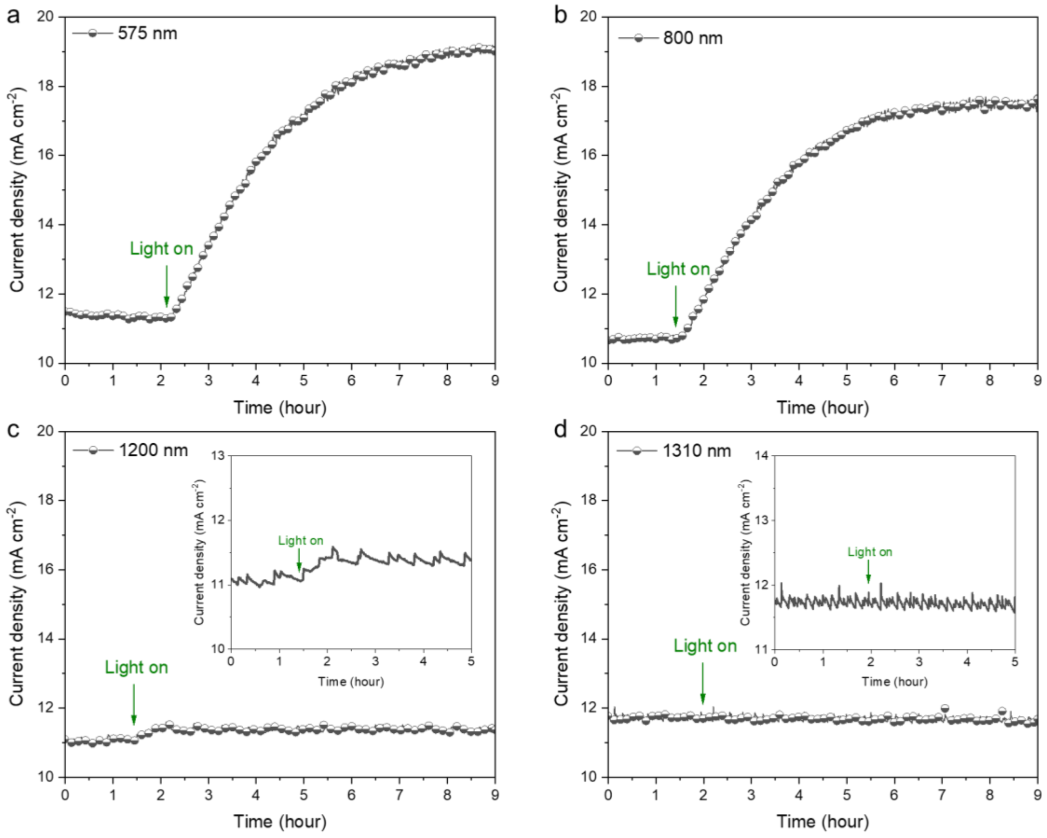

Fig.R1-16. Photon response measurements equipped with different wavelength filters of 575 nm (**a**), 800 nm (**b**), 1200 nm (**c**), and 1310 nm (**d**), respectively. (Supplementary Fig.S8 in our previous Supplementary Information, Supplementary Fig.S8 in our revised Supplementary Information)

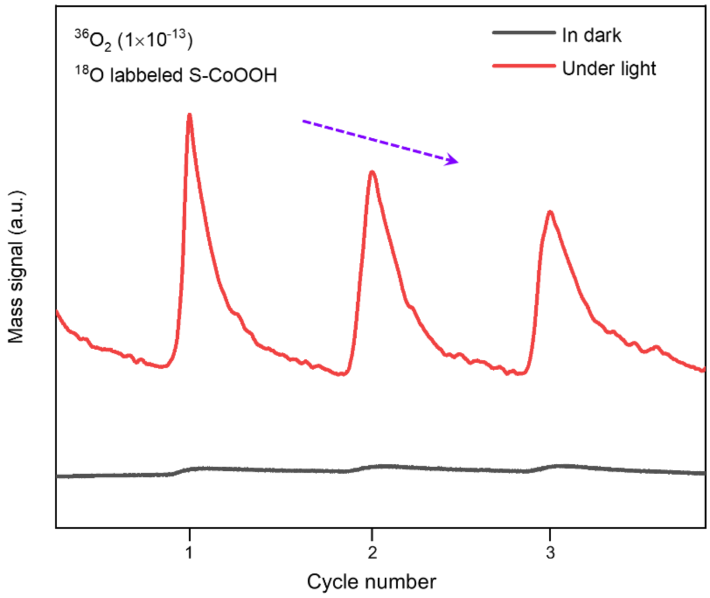

Fig.R1-17. Differential electrochemical mass spectrometry (DEMS) signals of O_2 products ($^{36}O_2$) for S-CoOOH measured with and without light irradiation. (Fig.4a in our previous manuscript, Fig.4a in our revised manuscript)

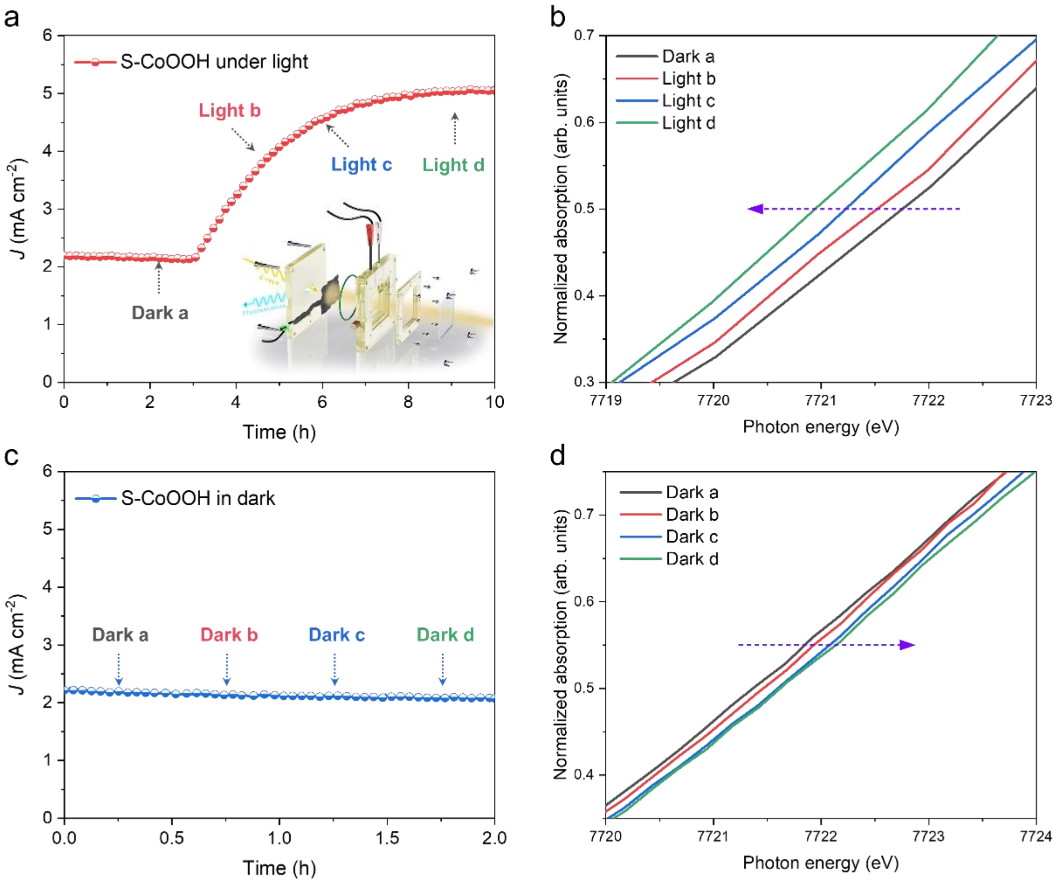

Fig.R1-18. ***Operando*** **Co** ***K*****-edge XAS measurements of S-CoOOH during OER under light and dark**

conditions. a Electrochemical curve for *operando* Co *K*-edge XAS measurements recorded during OER from dark to illumination at four selected points: Dark a, Light b, Light c, and Light d. **b** Enlarged *operando* Co *K*-edge XAS spectra of S-CoOOH during OER under light near the absorption edge, with dashed purple lines revealing spectral shifts. **c** Electrochemical curve for *operando* Co *K*-edge XAS measurements in the dark, recorded during the chronoamperometry (CA) process at four selected points: Dark a, Dark b, Dark c, and Dark d. **d** Enlarged *operando* Co *K*-edge XAS spectra of S-CoOOH during OER in dark near the absorption edge, with dashed purple lines indicating spectral shifts. (Fig.2a,c and Supplementary Figs.S19b, S20b in our previous manuscript and Supplementary Information, Fig.2a,c and Supplementary Figs.S23b, S24b in our revised manuscript and Supplementary Information)

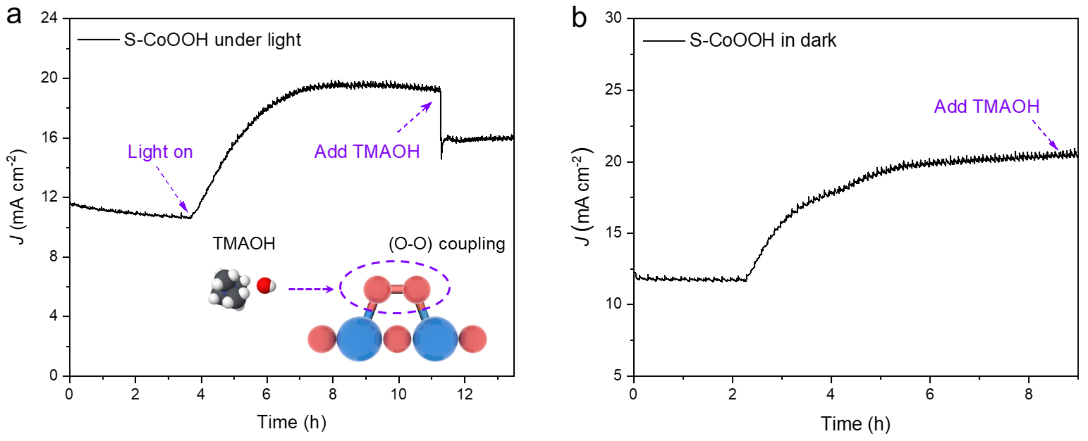

Fig.R1-19. The electrochemical curves respond to the addition of TMAOH under light (**a**) and in dark (**b**). (Fig.3a and Supplementary Fig.S23a in our previous manuscript and Supplementary Information, Fig.3a and Supplementary Fig.S28a in our revised manuscript and Supplementary Information)

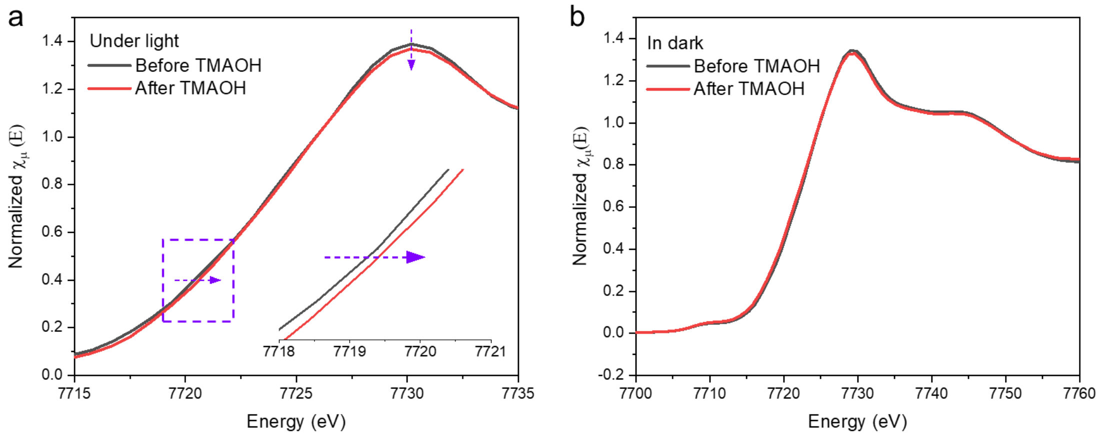

Fig.R1-20. Co *K*-edge XAS spectra of S-CoOOH recorded under light (**a**) and in dark (**b**) conditions, before

and after TMAOH addition. (Fig.3b and Supplementary Fig.S23b in our previous manuscript and Supplementary Information, Fig.3b and Supplementary Fig.S28b in our revised manuscript and Supplementary Information)

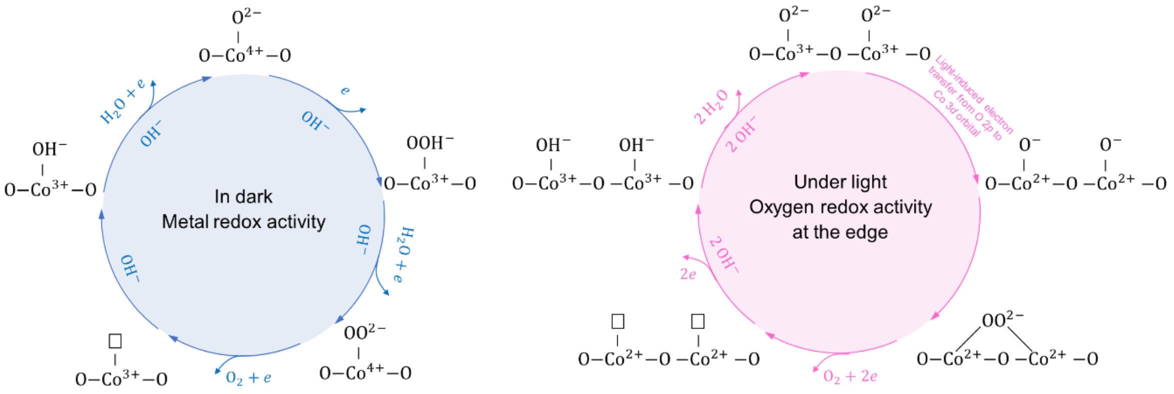

Fig.R1-21. Schematic illustration of OER reaction pathways of S-CoOOH in dark (left) and under light (right). In dark, cobalt acts as a redox center, whereas under light, edge oxygen serves as a redox center with LMCT triggered O_{NB} states formation. (Supplementary Figs.S24 and S27 in our previous Supplementary Information, Supplementary Figs.S29 and S32 in our revised Supplementary Information)

To address the comment, we have incorporated the proposed modifications into our manuscript (lines 107-109, page 4, highlighted in yellow): The photon response of different light intensities and wavelengths is not related to a conventional bandgap excitation, as further detailed in Supplementary Figs.S7-S8.

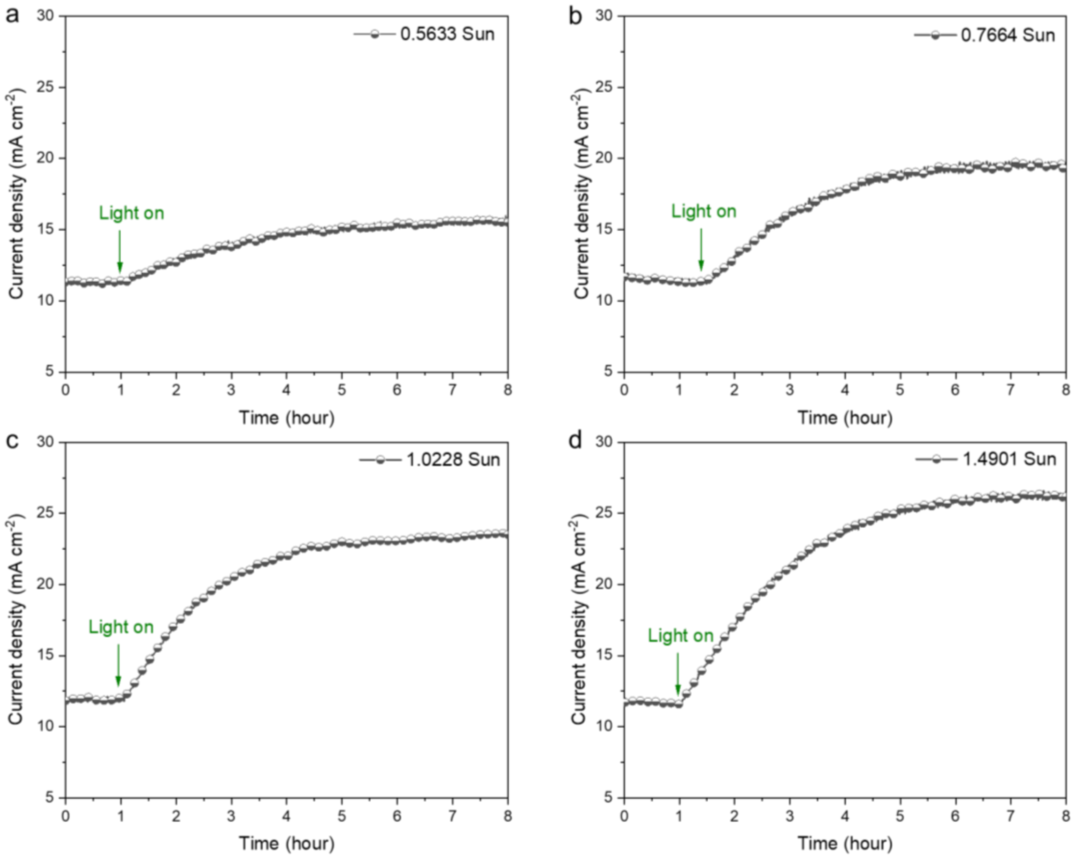

Supplementary Figure S7. Photon response measurements under different light intensities: **a** 0.5633 Sun (56.33 mW cm^{-2}), **b** 0.7664 Sun (76.64 mW cm^{-2}), **c** 1.0228 Sun (102.28 mW cm^{-2}), and **d** 1.4901 Sun (149.01 mW cm^{-2}).

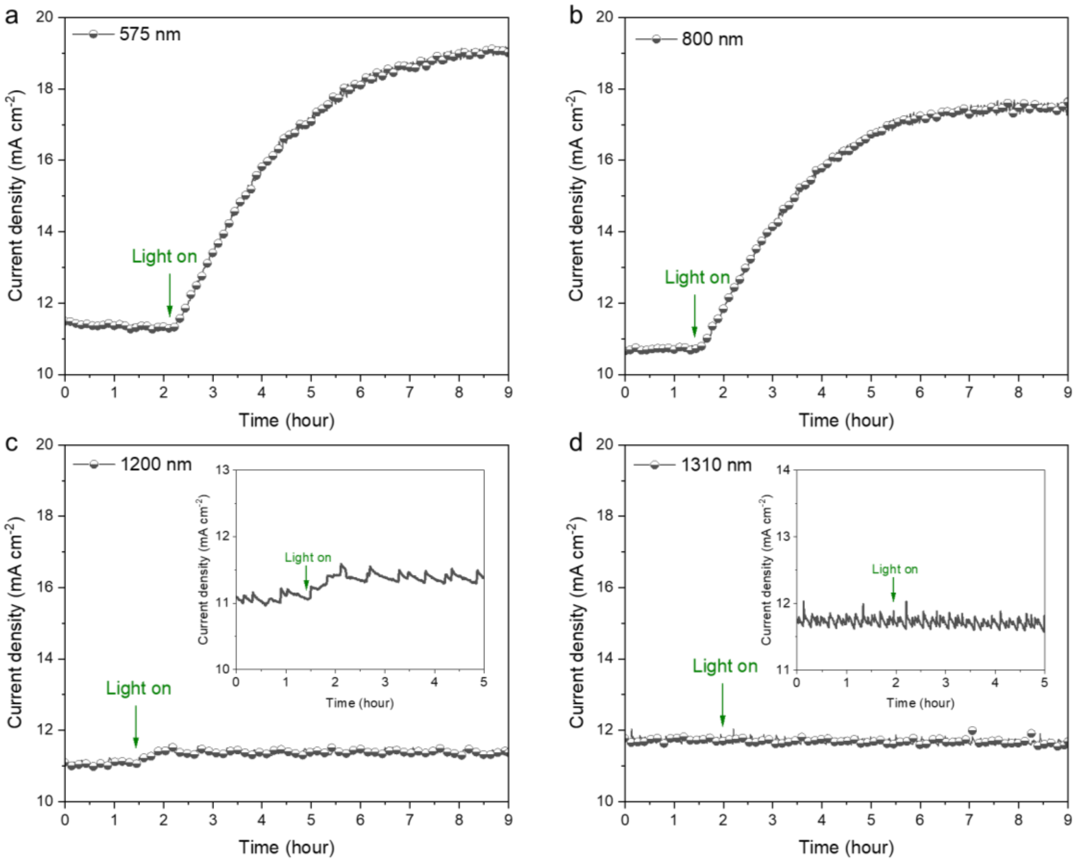

Supplementary Figure S8. Photon response measurements equipped with different wavelength filters of 575 nm (**a**), 800 nm (**b**), 1200 nm (**c**), and 1310 nm (**d**), respectively.

Moreover, we have revised the content accompanying Supplementary Fig.S8 in our supplementary information: Supplementary Fig.S8 provides the photon response measurements under different illumination wavelengths. It is shown that with a wavelength of 575 nm, the current increases significantly when light is switched on (Supplementary Fig.8a). As the wavelength increases to 800 nm, a noticeable current enhancement can still be observed (Supplementary Fig.8b). The PDOS of cobalt 3*d* and oxygen 2*p* orbitals reveals an energy barrier of 0.98 eV between (M-O) to Co 3*d* orbital in S-CoOOH, corresponding to a light wavelength of about 1260 nm (Supplementary Fig.S38). When a light source of 1200 nm is used, the current increment is barely detectable (Supplementary Fig.S8c), indicating that the wavelength of 1200 nm could trigger ligand to metal charge transfer (LMCT) minimally, which aligns with our calculations. Interestingly, switching the light source to a higher wavelength of 1310 nm does not trigger any observable change in the current (Supplementary Fig.8d), which suggests the inability of this wavelength to trigger LMCT due to the kinetic energy barrier. Thus, based on these results, it can be concluded that the lowest wavelength that can

trigger LMCT in S-CoOOH is about 1200 nm (1.03 eV), which is in close agreement with the calculated value (0.98 eV, 1260 nm).

In our work, the observed photon response of different light wavelengths is not related to a conventional band gap excitation, but is instead associated with light-induced electron transfer from occupied (M-O) bond to unoccupied Co 3*d* orbitals (Fig.5e,f). As discussed above, these photon response measurements show good agreement with the calculated energy barrier between (M-O) to Co 3*d* orbital (Supplementary Fig.S8). These findings strongly validate that photon response measurements under varying illumination wavelengths in our work are closely associated with electron transfer from (M-O) bond to Co 3*d* orbitals.

In addition, we have incorporated the proposed modifications into our manuscript (lines 270-272, page 12, highlighted in yellow): Based on the aforementioned *operando* XAS, *operando* DEMS, and TMAOH chemical probe experiments, the OER electron pathway of S-CoOOH shifts from metal redox activity in dark to oxygen redox activity under light, with neighboring O_{NB} states at its edge undergoing O-O coupling.

4. The influence of light carrier onto OER reaction pathway should be carefully confirmed, involving DFT calculations and electrochemical experiments.

Response: As discussed in response to Question 2, this light-induced electron transfer in our system is coupled to the OER process and can be regarded as a part of the O-O coupling step. To be more specific, this light-triggered electron transfer is feasible because S-CoOOH showcases a distinct non-overlapping region between Co 3*d* and 4*s* orbitals around the Fermi level, providing available unoccupied states that can accommodate excited electrons. Upon light illumination, electrons are transferred from oxygen to cobalt, generating non-bonding oxygen (O_{NB}) states, which are then stabilized via coupling with the neighboring O_{NB}. Based on these discussions, the light-induced carriers mentioned in our work do not refer to conventional mobile free charge carriers generated via a conventional bandgap excitation, but refer to a special Co-O octahedral framework, composing of reduced Co centers and O-O coupled oxygen species, which are spatially confined and do not exhibit mobility.

In the DFT calculations, the influence of this special Co-O octahedron on OER reaction pathway has been carefully considered. We have supplemented thermodynamic free-energy diagrams of S-CoOOH, showing the OER pathway in dark (without O-O coupling) and under light (with O-O coupling), as depicted in Fig.R1-22. These results indicate that the OER pathway under light exhibits a smaller theoretical overpotential (1.57 V) compared to that in dark (1.72 V), with the rate-determining step shifting from *OOH formation in dark to proton transfer under light. This suggests that light could alter the OER pathway, involving light-induced O-O coupling.

Moreover, electrochemical measurements were further conducted to experimentally confirm the influence of light carrier on OER reaction pathway. To this end, electrochemical measurements were combined with *operando* Co *K*-edge XAS and TMAOH chemical probe experiments to identify the light-induced special Co-O octahedral configuration, composing of reduced Co centers and O-O coupled oxygen species.

a) *Operando* Co *K*-edge XAS measurements were measured in a customized electrochemical cell to simulate the OER conditions (Fig.R1-23). Under light irradiation, the absorption edge (7718 to 7725 eV) of S-CoOOH exhibits a redshift compared with that in dark, and this redshift intensifies with prolonged light exposure. Such a redshift in photon energy reflects a reduction in Co valence states. In contrast, *operando* XAS measurement conducted under otherwise identical OER conditions in dark exhibits a gradual blueshift in absorption edge, consistent with the conventional Co^{3+}/Co^{4+} transition. The measurements monitor real-time changes in cobalt valence during the OER process, showing a decrease under light and an increase in dark, reflecting that light alters OER reaction pathway.

b) Here, whether O-O coupling actually takes place was investigated using tetraalkylammonium (TMA^+) probe combined with chronoamperometry (CA) measurements. As depicted in the inset of Fig.R1-24a, TMA^+ cation exhibits a specific interaction with O_{NB}, thereby blocking the formation of O-O coupling. If OER kinetics are inhibited upon adding TMA^+, it signifies the presence of O_{NB}-mediated O-O coupling. Fig.R1-24 demonstrates a significant reduction in the OER activity under light following the addition of TMA^+, contrasting with the stable OER activity of S-CoOOH in dark after adding TMA^+. These results support that O_{NB} is generated only under light, leading to a change in OER reaction pathway from *OOH formation in dark to direct O-O coupling under light.

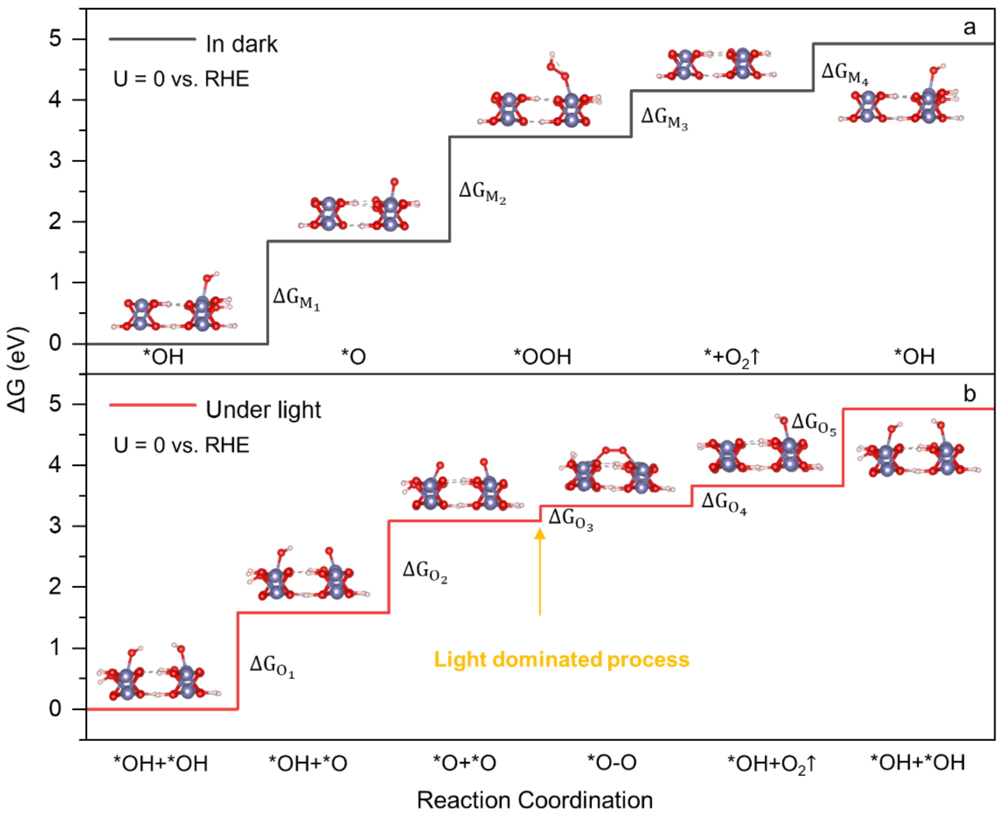

Fig.R1-22. Calculated reaction free energy diagrams of S-CoOOH in dark (a) and under light (b) on 0 V vs. RHE (where * refers to the metal sites). (Supplementary Fig.S37 in our revised Supplementary Information)

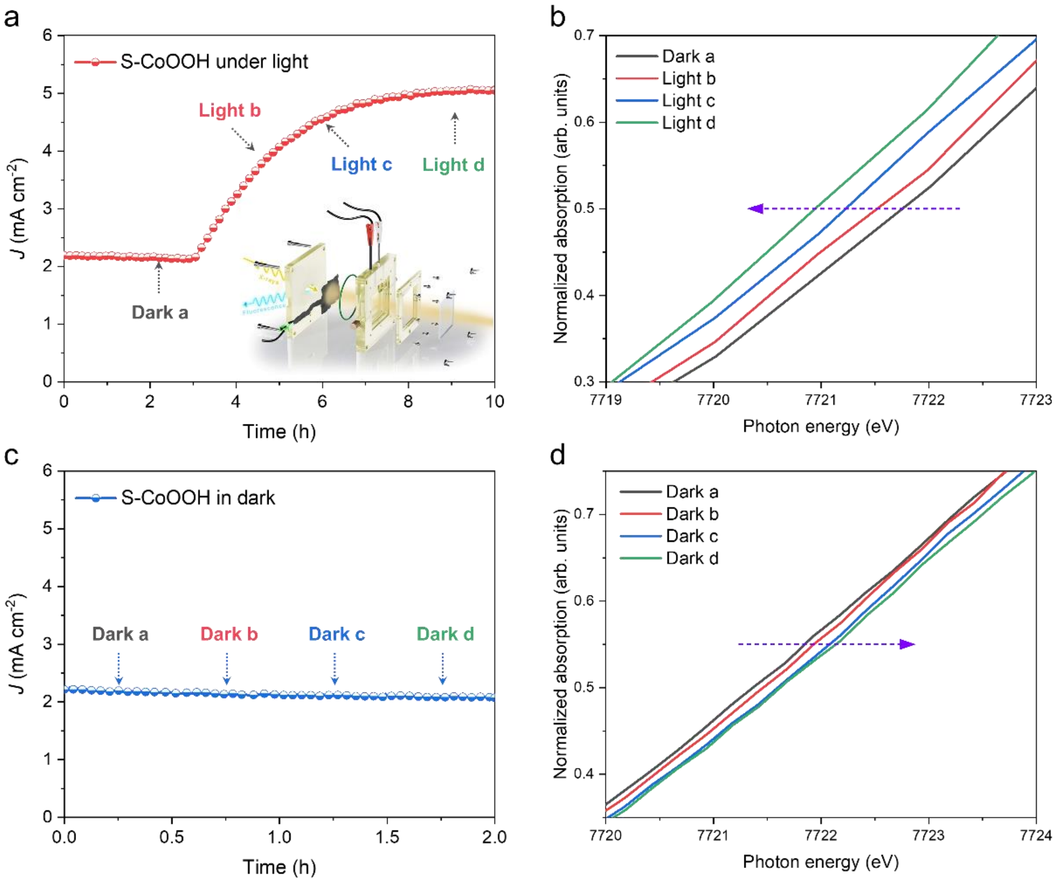

Fig.R1-23. ***Operando*** **Co** ***K*****-edge XAS measurements of S-CoOOH during OER under light and dark conditions.** **a** Electrochemical curve for *operando* Co *K*-edge XAS measurements recorded during OER from dark to illumination at four selected points: Dark a, Light b, Light c, and Light d. **b** Enlarged *operando* Co *K*-edge XAS spectra of S-CoOOH during OER under light near the absorption edge, with dashed purple lines revealing spectral shifts. **c** Electrochemical curve for *operando* Co *K*-edge XAS measurements in the dark, recorded during the chronoamperometry (CA) process at four selected points: Dark a, Dark b, Dark c, and Dark d. **d** Enlarged *operando* Co *K*-edge XAS spectra of S-CoOOH during OER in dark near the absorption edge, with dashed purple lines indicating spectral shifts. (Fig.2a, c and Supplementary Figs.S19b, S20b in our previous manuscript and Supplementary Information, Fig.2a, c and Supplementary Figs.S23b, S24b in our revised manuscript and Supplementary Information)

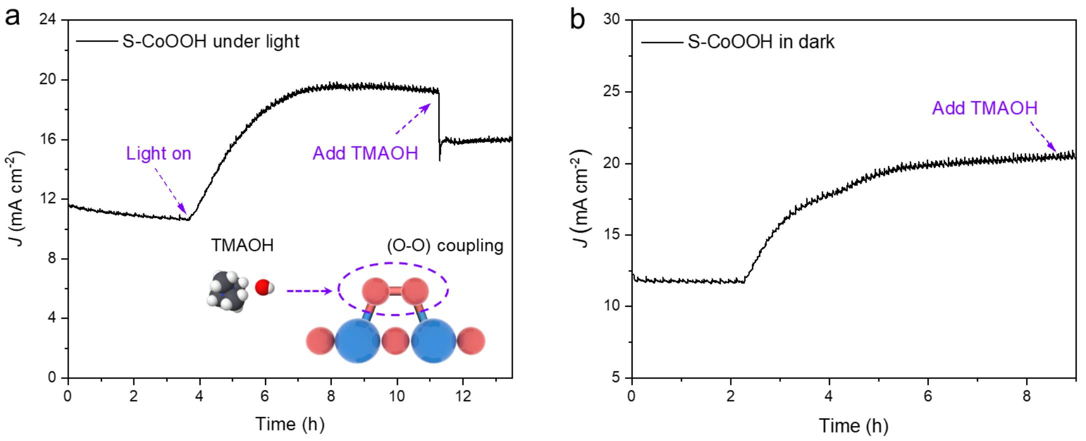

Fig.R1-24. The electrochemical curves respond to the addition of TMAOH under light (**a**) and in dark (**b**). (Fig.3a and Supplementary Fig.S23a in our previous manuscript and Supplementary Information, Fig.3a and Supplementary Fig.S28a in our revised manuscript and Supplementary Information)

To address the comment, we have incorporated the proposed modifications into our manuscript (lines 297-302, pages 12-13, highlighted in yellow): To further clarify the light-matter interaction, Fig.4d (State 3) depicts the underlying photoexcitation that in our work is coupled to OER: Upon light irradiation, electrons are transferred from oxygen to cobalt, generating O_{NB} states that are stabilized via coupling with the neighboring O_{NB} during O-O bond formation. Accordingly, the light carriers represent a special Co-O octahedral framework, composing of reduced Co centers and O-O coupled oxygen species, which are spatially confined and do not exhibit mobility.

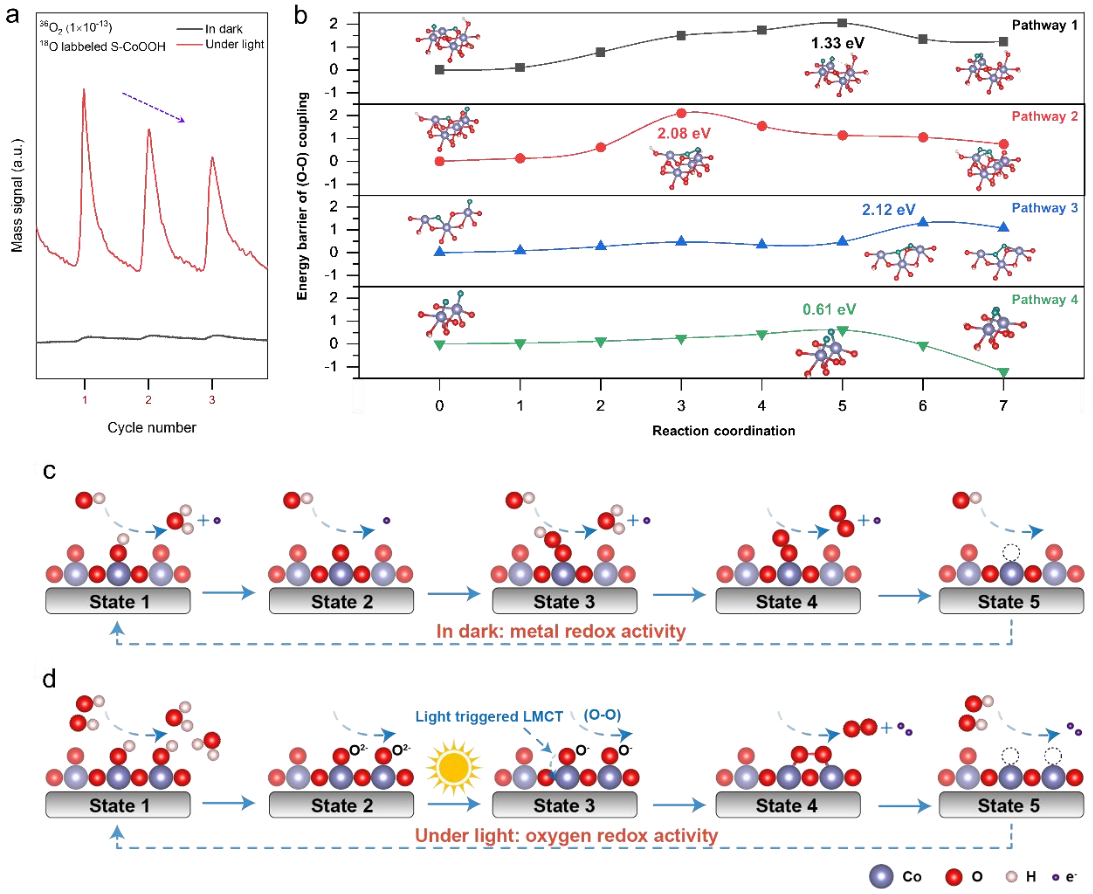

Figure 4. Determination of sites of oxygen redox activity. **a** Differential electrochemical mass spectrometry (DEMS) signals of O_2 products for S-CoOOH measured with and without light irradiation. **b** Energy profile diagram of O-O coupling involving oxygen atoms located either in the bulk or at the edge. The inset shows the proposed O-O coupling occurring at different oxygen sites, referred to as Pathway 1-4, where oxygen atoms marked in green represent the atoms involved in O-O coupling. **c** Electron transfer pathway of S-CoOOH in the dark, where cobalt acts as a redox center. **d** Electron transfer pathway of S-CoOOH under light, where oxygen serves as a redox center with LMCT triggered O_{NB} states formation.

Besides, we have incorporated the proposed modifications into our manuscript (lines 264-268, pages 11-12, highlighted in yellow): In addition to the kinetic processes discussed above, the thermodynamic processes under dark and light conditions are compared (Supplementary Fig.S37). These results indicate that the OER pathway under light exhibits a smaller overpotential (1.57 V) compared to that in dark (1.72 V), with the rate-determining step shifting from *OOH formation in dark to proton transfer under light.

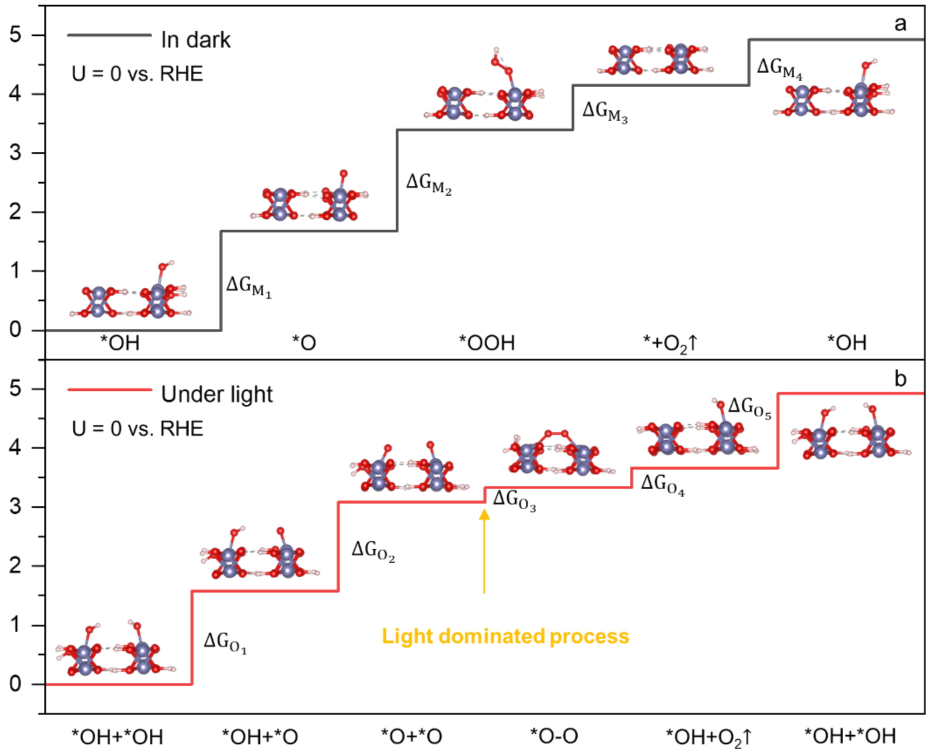

Supplementary Figure S37. Calculated reaction free energy diagrams of S-CoOOH in dark (**a**) and under light (**b**) on 0 V vs. RHE (where * refers to the metal sites).

5. The thermodynamics and kinetic processes should be simultaneously considered in your DFT calculations, especially for the contribution of electrolyte or adsorbates.

Response: Following the reviewer's valuable suggestion, we have supplemented thermodynamic free-energy diagrams of S-CoOOH under both dark and light conditions, incorporating an implicit solvation model to account for the contribution of electrolyte or adsorbates (Fig.R1-25). These results indicate that the OER pathway under light exhibits a smaller theoretical overpotential (1.57 V) compared to that in dark (1.72 V), with the rate-determining step shifting from *OOH formation in dark to proton transfer under light.

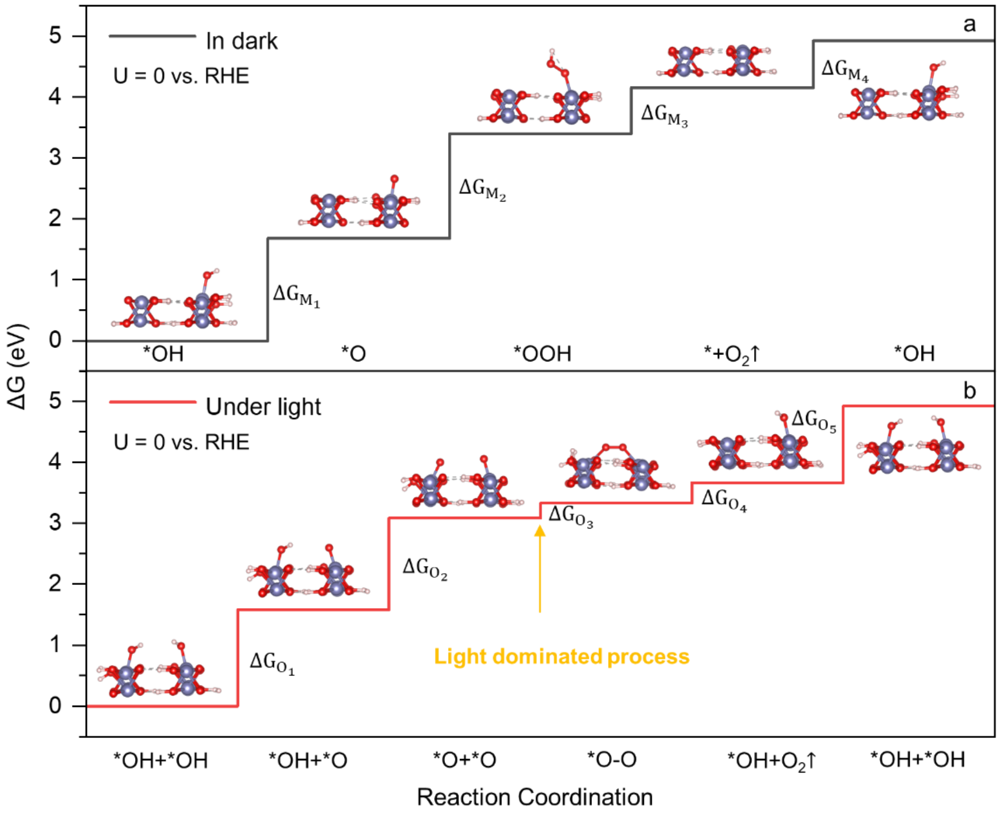

Fig.R1-25. Calculated reaction free energy diagrams of S-CoOOH in dark (a) and under light (b) on 0 V vs. RHE (where * refers to the metal sites). (Supplementary Fig.S37 in our revised Supplementary Information)

To address the comment, we have incorporated the proposed modifications into our manuscript (lines 264-268, pages 11-12, highlighted in yellow): In addition to the kinetic processes discussed above, the thermodynamic processes under dark and light conditions are compared (Supplementary Fig.S37). These results indicate that the OER pathway under light exhibits a smaller overpotential (1.57 V) compared to that in dark (1.72 V), with the rate-determining step shifting from *OOH formation in dark to proton transfer under light.

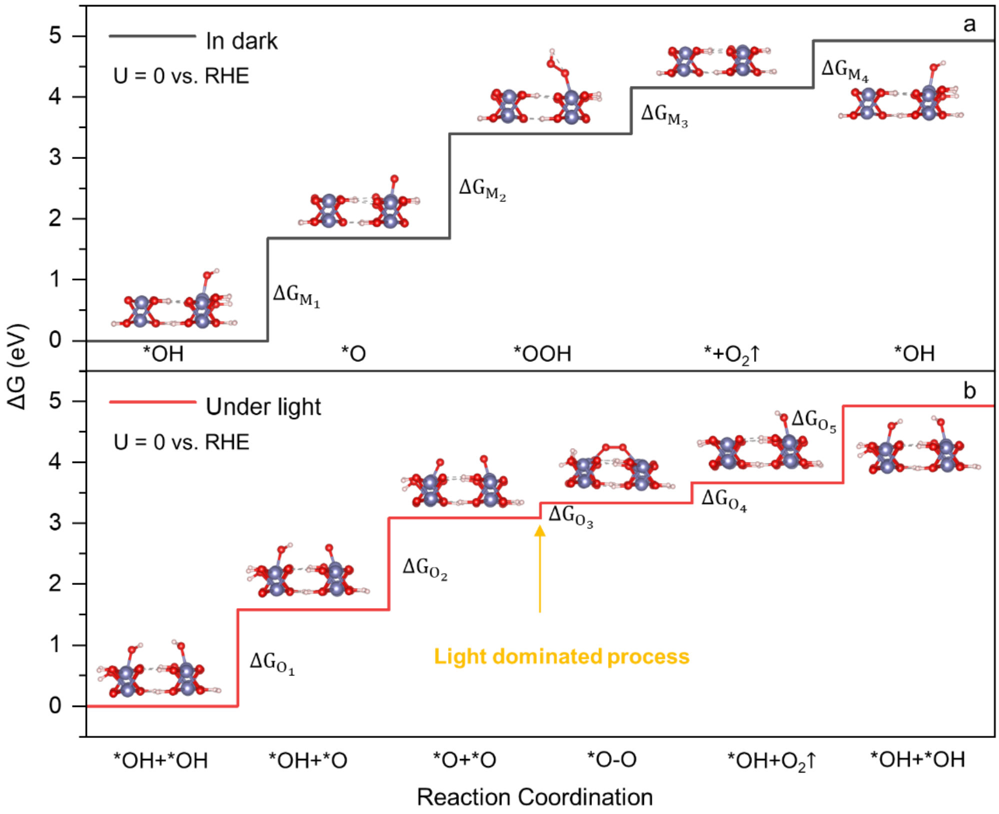

Supplementary Figure S37. Calculated reaction free energy diagrams of S-CoOOH in dark (**a**) and under light (**b**) on 0 V vs. RHE (where * refers to the metal sites).

Besides, we have supplemented the corresponding computational details in the Methods section of manuscript (lines 531-534, page 21, highlighted in yellow): Thermodynamics calculations were performed using the optPBE-vdW functional with the van der Waals (vdW) correction. The solvent effect was considered using the implicit solvation model implemented in the VASP model, with the free energies referenced to U = 0 V vs. RHE. More detailed computational parameters are provided in Supplementary Information.

In addition, more detailed parameters regarding the solvent effect are provided following Supplementary Figure S37: The thermodynamics calculations were performed using the optPBE-vdW functional with the van der Waals (vdW) correction. The Gibbs free energy change (ΔG) for each elemental step in OER can be obtained by the following formula: $\Delta G = \Delta E + \Delta ZPE - T\Delta S$

In dark (metal redox activity):

$\Delta G_{\mathrm{M_1}} = \Delta G_{[*\mathrm{O}]} - \Delta G_{[*\mathrm{OH}]} - \mathrm{eU}$

$\Delta G_{M_2} = \Delta G_{[*OOH]} - \Delta G_{[*O]} - eU$

$\Delta G_{M_3} = 4.92\ \text{eV} - \Delta G_{[*]} + \Delta G_{[*OOH]} - eU$

$\Delta G_{M_4} = \Delta G_{[*OH]} - \Delta G_{[*]} - eU$

Under light (oxygen redox activity):

$\Delta G_{O_1} = \Delta G_{[*OH+*O]} - \Delta G_{[*OH+*OH]} - eU$

$\Delta G_{O_2} = \Delta G_{[*O+*O]} - \Delta G_{[*OH+*O]} - eU$

$\Delta G_{O_3} = \Delta G_{[*O-O]} - \Delta G_{[*O+*O]} - eU$ (Light dominated process)

$\Delta G_{O_4} = 4.92\ \text{eV} - \Delta G_{[*OH]} + \Delta G_{[*O-O]} - eU$

$\Delta G_{O_5} = \Delta G_{[*OH+*OH]} - \Delta G_{[*OH]} - eU$

To maintain numerical stability for the large system (over 400 atoms), the linear mixing scheme was enforced for electronic density, and the LMIXTAU tag was enabled to stabilize the density mixing in the presence of U parameters. To account for the electrolyte environment and long-range electrostatic screening, an implicit solvation model as implemented in VASPsol was employed[5]. The solvent was treated as a continuum dielectric medium with a relative permittivity of ε_b = 78.4, representing the aqueous phase. The solute-solvent interface was defined by a critical density threshold of n_c = 0.0025 Å[6]. To simulate the ionic screening effect in the electrolyte (e.g., 1.0 M KOH), the linearized Poisson-Boltzmann equation was solved by specifying a Debye screening length (λ_D) of 3.04 Å[7]. This parameter describes the characteristic thickness of the diffuse part of the electrochemical double layer (EDL), allowing for a more realistic description of the interfacial energetics during the oxygen evolution reaction process.

6. In addition, if the reaction pathway of OER can be changed by the light irradiations, the adsorption configuration may be reflected by their changes in entropy or enthalpy values.

Response: As discussed in response to Question 5, in our work light dominated process occurs at the O-O coupling step. Accordingly, we have calculated the entropy (T·S), enthalpy (H), and Gibbs free energy (G) values before (State 3 in Fig.R1-26) and after (State 4 in Fig.R1-26) this O-O coupling step. As displayed in Table R1-1, State 4 exhibits a significant enthalpic stabilization relative to State 3 (ΔH = -4.558 kcal/mol), accompanied by an increased entropy contribution (ΔT·S = +4289.385 J/mol). Accordingly, the Gibbs free

energy decreases by 5.582 kcal/mol, confirming that the transformation from State 3 to State 4 is thermodynamically favorable. Thus, these results suggest that light could affect the adsorption configuration, further altering the OER pathway.

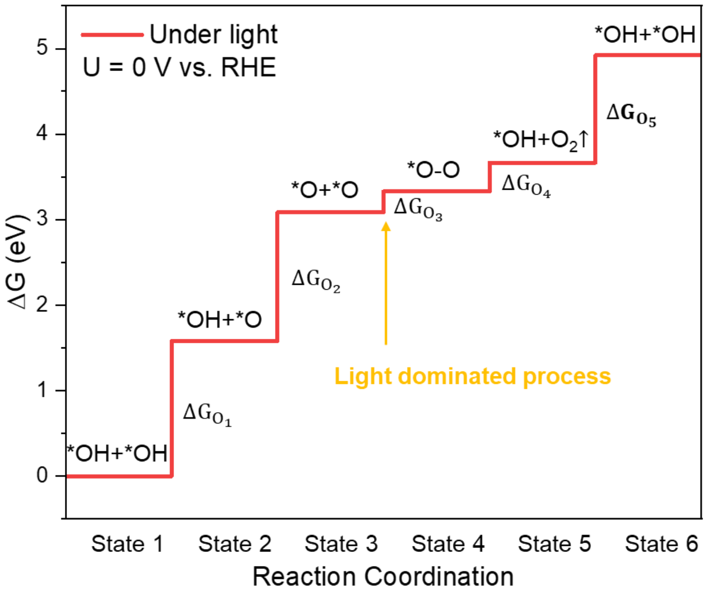

Fig.R1-26. Calculated reaction free energy diagram of S-CoOOH under light on 0 V vs. RHE, showing that light dominated process occurs at the O-O coupling step. (Supplementary Fig.S37 in our revised Supplementary Information)

Table R1-1. Calculated entropy (T·S), enthalpy (H), and Gibbs free energy (G) values for State 3 ($^*O^*O$, before light-induced O-O coupling) and State 4 ($^*O$-O, after light-induced O-O coupling) at 298.1 K. (Supplementary Table S4 in our revised Supplementary Information)

	entropy (T·S) J/mol	enthalpy (H) kcal/mol	Gibbs free energy (G) kcal/mol
State 3 $^*O^*O$	4443.833	8.942	7.879
State 4 $^*O$-O	8733.218	4.384	2.297
Δ	+4289.385	-4.558	-5.582

To address the comment, we have incorporated the proposed modifications into our manuscript (lines 268-269, page 12, highlighted in yellow): The effect of light is reflected by the changes in entropy, enthalpy, and

Gibbs free energy values (Supplementary Table S4).

Supplementary Table S4. Calculated entropy (T·S), enthalpy (H), and Gibbs free energy (G) values for the states before and after light-induced O-O coupling at 298.1 K.

	entropy (T·S) J/mol	enthalpy (H) kcal/mol	Gibbs free energy (G) kcal/mol
Before light-induced O-O coupling ($^{*}O^{*}O$)	4443.833	8.942	7.879
After light-induced O-O coupling ($^{*}O$-O)	8733.218	4.384	2.297
Δ	+4289.385	-4.558	-5.582

Moreover, we have revised the content accompanying Supplementary Table S4 in our supplementary information: Light dominated process occurs at the O-O coupling step. To investigate the role of light, entropy (T·S), enthalpy (H), and Gibbs free energy (G) values were calculated for the states before ($^{*}O^{*}O$) and after ($^{*}O$-O) this O-O coupling step. As displayed in Supplementary Table S4, $^{*}O$-O exhibits a significant enthalpic stabilization relative to $^{*}O^{*}O$ (ΔH = -4.558 kcal/mol), accompanied by an increased entropy contribution (ΔT·S = +4289.385 J/mol). Accordingly, the Gibbs free energy decreases by 5.582 kcal/mol, confirming that the transformation from $^{*}O^{*}O$ to $^{*}O$-O is thermodynamically favorable. Thus, these results suggest that light could affect the adsorption configuration, further altering the OER pathway.

7. How about the lifetime (ps or ns) of your light-induced carriers and OER reaction (ms)? How to couple together? Why they can occur? What is their effective distance of charge transfer? In a word, time-dependent spectral analysis and fundamental physical modes should be established.

Response: As discussed in response to Question 4, the light-induced carriers refer to a special Co-O octahedral framework, composing of reduced Co centers and O-O coupled oxygen species, which are spatially confined and do not exhibit mobility. Hence, this light-induced electron transfer in our system is coupled to the OER process and can be regarded as a part of the O-O coupling step. To be more specific, this

light-triggered electron transfer is feasible because S-CoOOH showcases a distinct non-overlapping region between Co 3*d* and 4*s* orbitals around the Fermi level, providing available unoccupied states that can accommodate excited electrons. Upon light illumination, electrons are transferred from oxygen to cobalt, generating non-bonding oxygen (O_{NB}). Although an isolated O_{NB} is thermodynamically unstable, two adjacent O_{NB} can couple to form direct O-O coupling, thereby stabilizing the photoinduced intermediate. Hence, the lifetime of the light carriers should be in the scale of ms.

Regarding the effective distance of charge transfer, the light carriers in our work represent a special Co-O octahedral framework, composing of reduced Co centers and O-O coupled oxygen species. Because these states do not undergo long-range charge migration, the concept of an effective charge transfer distance is not applicable in this case.

Moreover, we have performed transition absorption (TA) spectroscopy of S-CoOOH using 700 nm as an excitation source (Fig.R1-27). No clear transient features are observed in the 2D TA map, except for a signal around 700 nm arising from excitation light scattering. This is consistent with our above discussion light-induced electron transfer is coupled to OER process, rather than a conventional photophysical excitation.

In addition, we now provide an improved and clearer fundamental physical model underlying the electron transfer pathway under light, highlighting how the LMCT process couples with OER steps, as illustrated in Fig.R1-28.

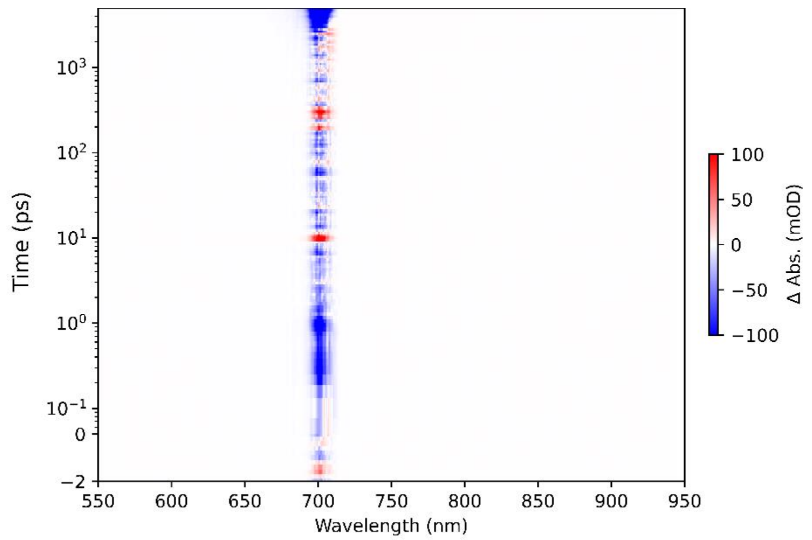

Fig.R1-27. 2D-false color plot of fs-transient absorption in 550-950 nm for S-CoOOH using 700 nm as an

excitation source, with the signal around 700 nm arising from excitation light scattering. (Supplementary Fig.S25 in our revised Supplementary Information)

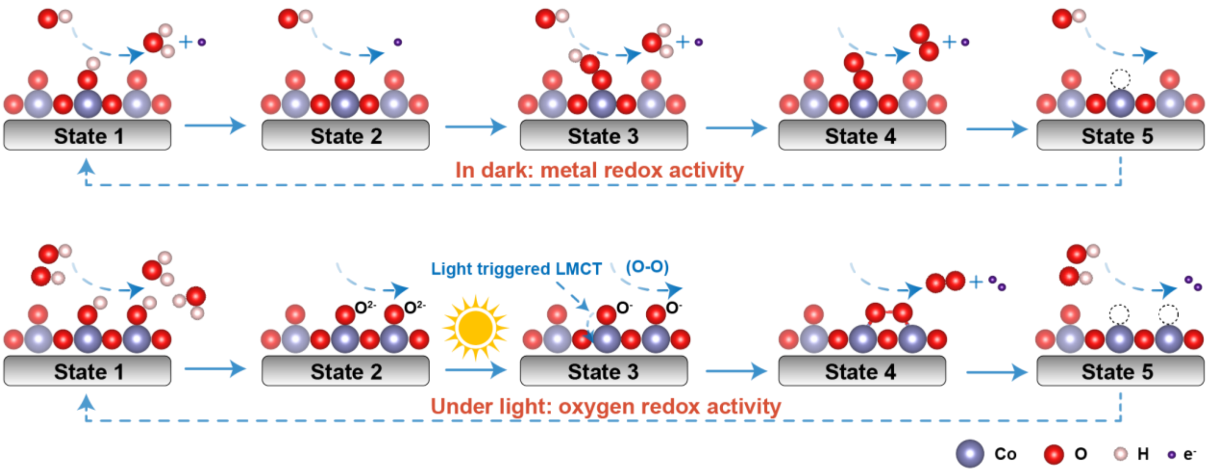

Fig.R1-28. Electron transfer pathways of S-CoOOH in dark (top) and under light (bottom). The light triggered LMCT occurs at State 3 (bottom) and is coupled to OER, where light induced electron transfer generates 7-electron oxygen (O^-), leading to the formation of O-O coupling. (Fig.4c,d in our previous manuscript, Fig.4d,c in our revised manuscript)

To address the comment, we have incorporated the proposed modifications into our manuscript (lines 297-302, pages 12-13, highlighted in yellow): To further clarify the light-matter interaction, Fig.4d (State 3) depicts the underlying photoexcitation that in our work is coupled to OER: Upon light illumination, electrons are transferred from oxygen to cobalt, generating O_{NB} states that are stabilized via coupling with the neighboring O_{NB} during O-O bond formation. Accordingly, the light carriers represent a special Co-O octahedral framework, composing of reduced Co centers and O-O coupled oxygen species, which are spatially confined and do not exhibit mobility.

Besides, we have incorporated the proposed modifications into our manuscript (lines 154-156, page 6, highlighted in yellow): Importantly, the LMCT involved here is not a conventional photophysical LMCT, but rather a step of OER processes that is coupled to chemical bond formation, and thus cannot be detected by transient absorption spectroscopy (Supplementary Fig.S25).

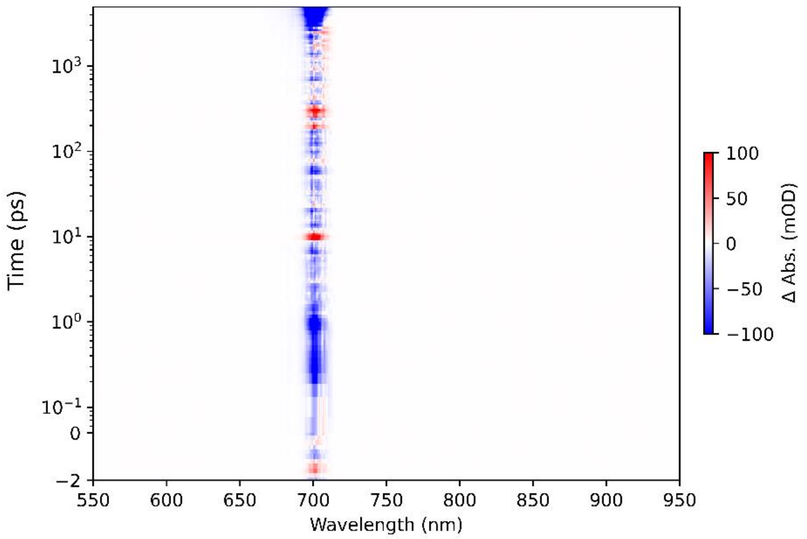

Supplementary Figure S25. 2D-false color plot of fs-transient absorption in 550-950 nm for S-CoOOH using 700 nm as an excitation source, with the signal around 700 nm arising from excitation light scattering.

The transient absorption (TA) spectroscopy of S-CoOOH was performed using 700 nm as an excitation source (Supplementary Figure S25). No clear transient features are observed in the 2D TA map, except for a signal around 700 nm arising from excitation light scattering. This is reasonable because the light-induced electron transfer in our system is not a conventional photophysical excitation. In our proposed mechanism, S-CoOOH first adsorbs OH^- and undergoes deprotonation to form the $^*O$ intermediate under applied potential. Light then acts on this deprotonated $^*O$ species, generating 7-electron oxygen ($^*O^-$). Two adjacent $^*O^-$ subsequently undergo direct O-O coupling, which stabilizes the photoinduced state. This light-induced electron transfer is coupled to the OER process and is regarded as a part of O-O coupling step. In the absence of bias, the deprotonated $^*O$ intermediate is not formed and no light-induced electron transfer is triggered, which explain the absence of observable TA signals.

In addition, we have revised the fundamental physical model and clearly marked light induced LMCT process (lines 278-280, page 12, highlighted in yellow): 2) Next, a light-triggered LMCT coupled to OER occurs (State 3 in Fig.4d), involving an electron transfer from (M-O) to Co 3*d* orbitals, thereby generating O_{NB} states with seven outermost electrons (O^-).

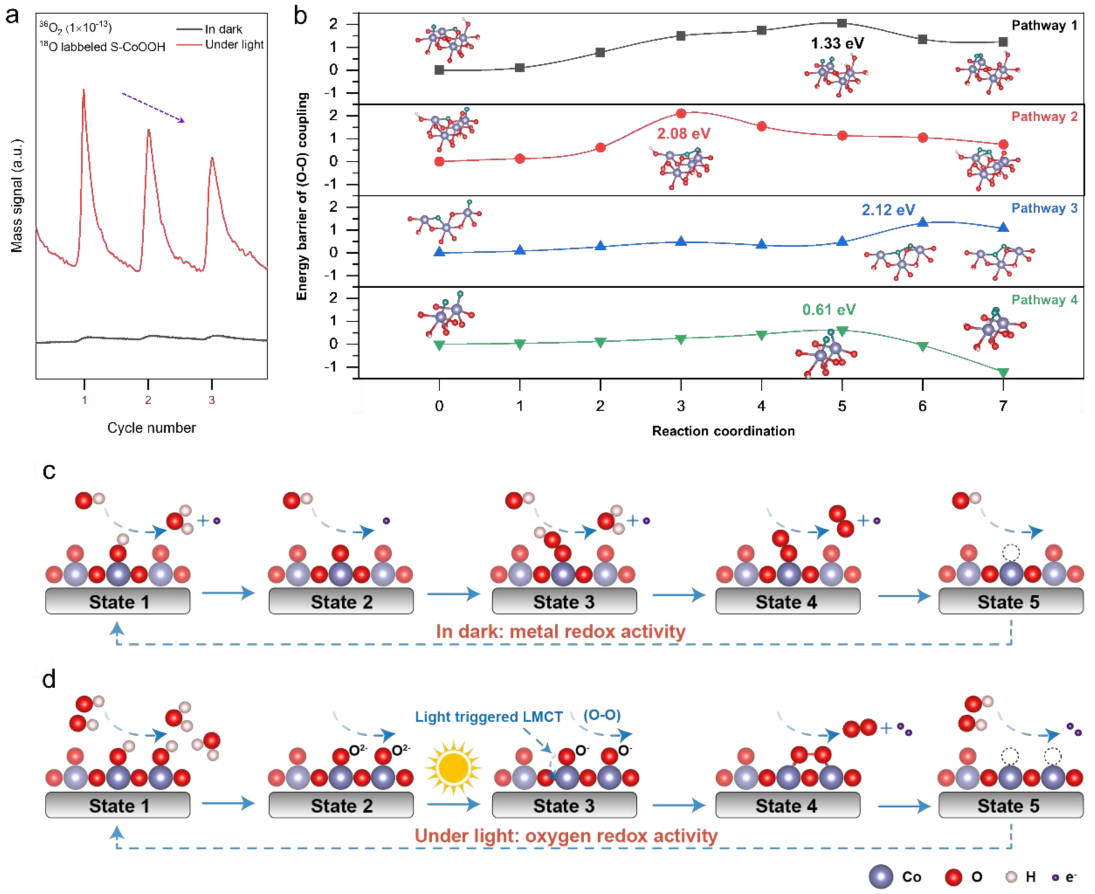

Figure 4. Determination of sites of oxygen redox activity. **a** Differential electrochemical mass spectrometry (DEMS) signals of O_2 products for S-CoOOH measured with and without light irradiation. **b** Energy profile diagram of O-O coupling involving oxygen atoms located either in the bulk or at the edge. The inset shows the proposed O-O coupling occurring at different oxygen sites, referred to as Pathway 1-4, where oxygen atoms marked in green represent the atoms involved in O-O coupling. **c** Electron transfer pathway of S-CoOOH in the dark, where cobalt acts as a redox center. **d** Electron transfer pathway of S-CoOOH under light, where oxygen serves as a redox center with LMCT triggered O_{NB} states formation.

Reviewer #2

Remarks to the Author: Overall, most of the reviewer's questions have been addressed. However, two additional points should be clarified.

1. First, regarding the photophysical properties of SCoOOH, reflectance measurements would help clearly demonstrate the light absorption of S-CoOOH, and the details of the UV/Vis measurement conditions should be specified.

Response: Following the reviewer's valuable suggestions, we have performed additional reflectance measurements on S-CoOOH (Fig.R2-1). The diffuse reflectance spectroscopy exhibits weak reflection and strong light absorption across 200-2400 nm, consistent with the absorption spectrum in this spectral range (Fig.R2-2).

Also, we have added the details of UV-Vis measurements conditions into the Methods section of our revised manuscript, as follows: UV-Vis absorption and reflectance measurements. UV-Vis absorption and diffuse reflectance spectra of S-CoOOH powder were collected on a spectrophotometer (LAMBDA 950, PerkinElmer, USA) equipped with an integrating sphere. The measurements were performed in the wavelength range of 200-2400 nm with a step size of 5 nm, using $BaSO_4$ as the reflectance reference. A deuterium lamp was used for the 200-312 nm region, while a tungsten lamp was employed for the 312-2400 nm region.

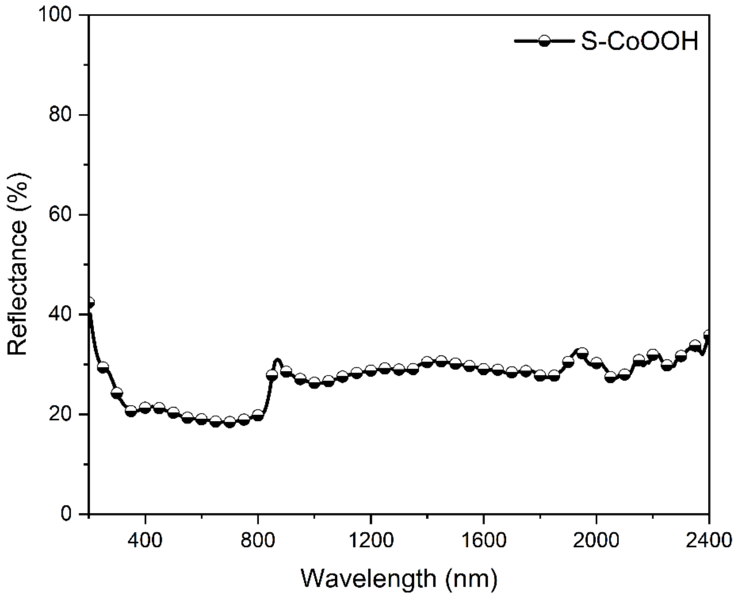

Fig.R2-1. UV-Vis reflectance spectrum of S-CoOOH powder measured in the 200-2400 nm wavelength range. (Supplementary Fig.S39b in our revised Supplementary Information)

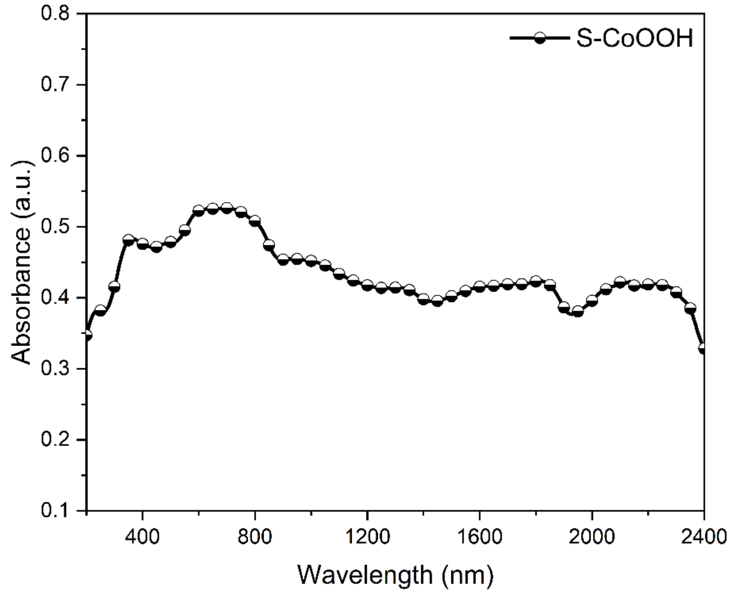

Fig.R2-2. UV-Vis absorption spectrum of S-CoOOH powder measured in the 200-2400 nm wavelength range. (Supplementary Fig.S33 in our previous Supplementary Information, Supplementary Fig.S39a in our revised Supplementary Information)

To address the comment, we have implemented suggested revisions into Methods section of our manuscript (lines 545-549, pages 21-22, highlighted in yellow): UV-Vis absorption and reflectance measurements. UV-Vis absorption and diffuse reflectance spectra of S-CoOOH powder were collected on a spectrophotometer

(LAMBDA 950, PerkinElmer, USA) equipped with an integrating sphere. The measurements were performed in the wavelength range of 200-2400 nm with a step size of 5 nm, using $BaSO_4$ as the reflectance reference. A deuterium lamp was used for the 200-312 nm region, while a tungsten lamp was employed for the 312-2400 nm region.

Besides, we have incorporated the proposed modifications into our manuscript (lines 353-356, page 15, highlighted in yellow): The photophysical property of S-CoOOH was further examined by UV-Vis absorption and diffuse reflectance spectra (Supplementary Figs.S39a,b), revealing intense light absorption over 200-2400 nm (0.52-6.20 eV), which provides the required energy to promote electron transfer from (M-O) to Co 3*d* orbitals (LMCT) in S-CoOOH.

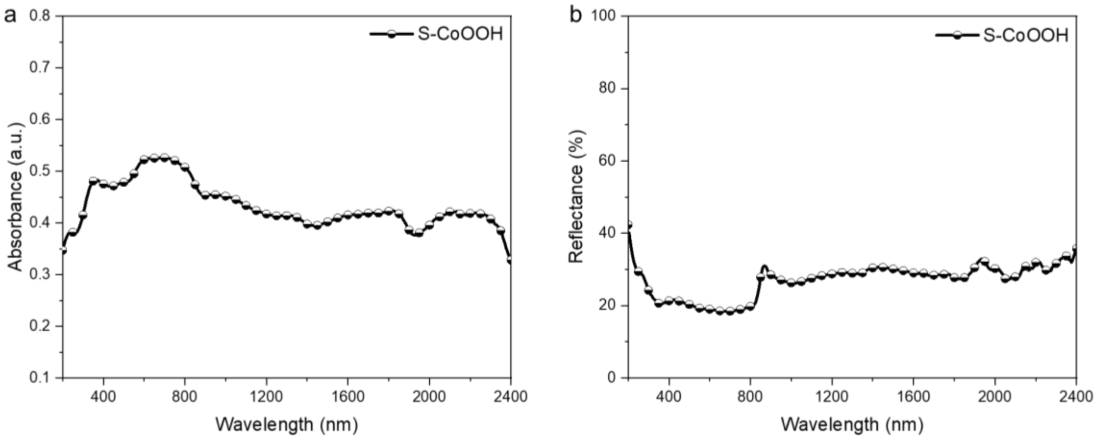

Supplementary Figure S39. UV-Vis absorption (**a**) and reflectance spectra (**b**) of S-CoOOH powder measured in the 200-2400 nm wavelength range.

2. Second, the analysis of structural reconstruction of oxyhydroxides after the OER appears insufficient. Adding XRD analysis is recommended to strengthen this part.

Response: Following the reviewer's insightful suggestion, we have added a detailed XRD analysis below the XRD pattern, as follows: As displayed in Fig.R2-3, after a long-time electro-oxidation process (10-hour CP), the XRD pattern reveals that cobalt sulfide pre-catalyst is fully reconstructed into rhombohedral β-CoOOH (R-3m space group, JCPDS 07-0169). The diffraction peaks at 20.24°, 36.98°, 38.89°, 50.58°, 65.34°, and 69.17° can be indexed to (003), (101), (012), (015), (110), and (113) crystal planes of β-CoOOH,

respectively. The diffraction peak at 25.83° can be attributed to (002) crystal plane of carbon cloth substrate. No additional reflections associated with other crystalline phases are detected after electro-oxidation, suggesting a complete structural reconstruction.

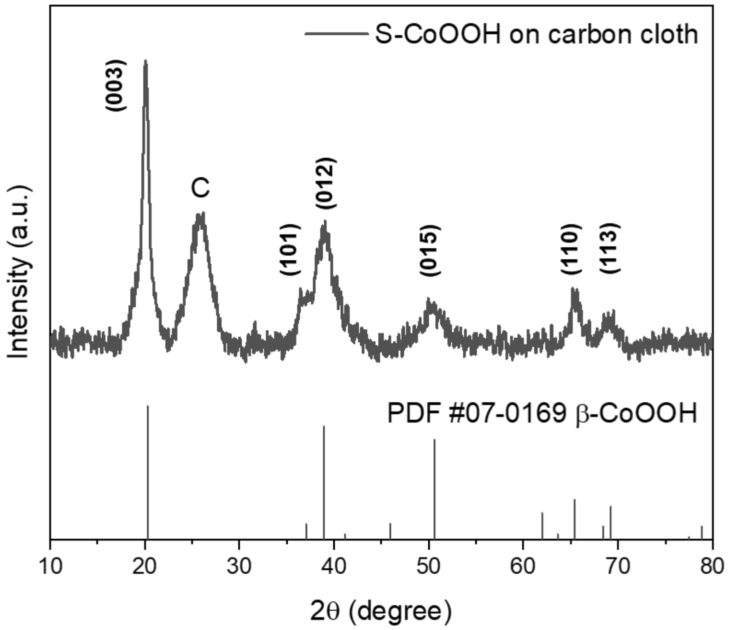

Fig.R2-3. XRD pattern of S-CoOOH based on carbon cloth, with standard β-CoOOH listed (PDF #07-0169). (Supplementary Fig.S1a in our previous Supplementary Information, Supplementary Fig.S1a in our revised Supplementary Information)

To address the comment, we have incorporated the proposed modifications into our manuscript (lines 88-91, page 4, highlighted in yellow): The cobalt sulfides were completely reconstructed into β-CoOOH (R-3m space group, JCPDS 07-0169), leaving negligible residual sulfur, as confirmed by X-ray diffraction (XRD), X-ray photoelectron spectroscopy (XPS), Co *K*-edge XAS, and inductively coupled plasma (ICP) measurements (Supplementary Figs.S1-S2 and Table S1).

Furthermore, a more detailed structural analysis of XRD pattern of oxyhydroxides after OER has been included below Supplementary Figure S1a: As shown in Supplementary Fig.S1a, after a long-time electro-oxidation process (10-hour CP), XRD result reveals that cobalt sulfide pre-catalyst is fully reconstructed into rhombohedral β-CoOOH (R-3m space group, JCPDS 07-0169). The diffraction peaks at 20.24°, 36.98°, 38.89°, 50.58°, 65.34°, and 69.17° can be indexed to (003), (101), (012), (015), (110), and (113) crystal

planes of β-CoOOH, respectively. The diffraction peak at 25.83° can be attributed to (002) crystal plane of carbon cloth substrate. No additional reflections associated with other crystalline phases are detected after electro-oxidation, suggesting a complete structural reconstruction.

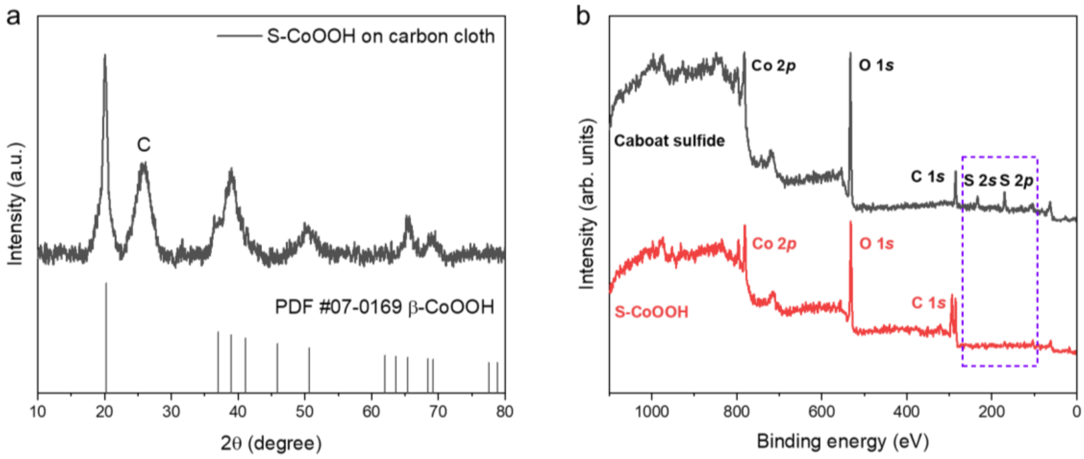

Supplementary Figure S1. a XRD pattern of S-CoOOH based on carbon cloth, with standard β-CoOOH listed (PDF #07-0169). **b** XPS spectra of cobalt sulfide before (top) and after activation (bottom) in a wide range.

Reviewer #3

Remarks to the Author: This manuscript presents a systematic study on the photo-assisted oxygen evolution reaction (OER) of cobalt oxyhydroxide, combining operando spectroscopic experiments with density functional theory (DFT) analysis. The work is overall comprehensive, and the integration of theory and experiment is commendable. However, several key aspects of the proposed mechanism and methodology remain insufficiently supported.

1. The authors attribute enhanced catalytic performance under illumination to a photo-induced LMCT process. However, the supporting evidence mainly relies on spectral intensity variations in XAS and photocurrent enhancement, both of which are indirect indicators. Such observations could also result from light-induced charge redistribution and electrochemical potential shifts. The manuscript would be significantly strengthened if the authors could provide direct experimental evidence for LMCT, such as transient absorption spectroscopy or photoelectron spectroscopy data.

Response: In our work, the mentioned photon induced LMCT is not a conventional LMCT, which is a step of OER process. Hence, the presence of non-bonding oxygen (O_{NB}) species (electron transfer from ligand) and decrease of cobalt (electron transfer to metal) could be verified by combination of TMAOH chemical probe and *operando* Co *K*-edge XAS experiments.

a) **First, *operando* Co *K*-edge XAS measurements of S-CoOOH reveal a light-induced reduction in cobalt valence.** During the OER, a distinct redshift of the Co *K*-edge adsorption energy (7718 to 7725 eV) is observed under light (Fig.R3-1a,b), whereas no such redshift appears in dark (Fig.R3-1c,d). This light-induced redshift reflects a reduction in Co valence state under light.

b) **Second, the presence of O_{NB} states can be probed by TMAOH, as inhibition of OER kinetics upon adding TMA^+ indicates their formation** (*Angew. Chem. Int. Ed.* 2017, 56, 8652-8656; *Nat. Energy* 2019, 4, 329-338). Upon the addition of TMAOH, S-CoOOH exhibits a significant reduction in OER activity under light (Fig.R3-2a), contrasting with its stable OER activity in dark (Fig.R3-2b). These results demonstrate that the O_{NB} species are generated under light, consistent with light-induced LMCT.

c) Third, to further identify the formation of O_{NB} under light, TMA^+ probe measurements combined with Co *K*-edge XAS were employed. Theoretically, the bonding of oxidized oxygen species with TMA^+ would trigger an inner electron transfer from Co atom to TMA^+, leading to an increase in cobalt valence.

As shown in the Co *K*-edge spectra of S-CoOOH before and after adding TMA^+, a blueshift occurs under light (Fig.R3-3a), signifying an increase in cobalt valence, while no detectable change is observed for S-CoOOH in dark upon TMA^+ addition (Fig.R3-3b). These results further support O_{NB} species are generated under light, and therefore LMCT takes place under light.

Taken together, the observed reduction in cobalt valence and the formation of O_{NB} species during OER under light constitute direct and compelling evidence for light-induced LMCT.

Moreover, both light-induced charge redistribution and electrochemical potential shifts have been excluded. As discussed above, in our work the photon-induced LMCT process also involves the chemical bonding, *i.e.* O-O coupling. Hence, the possibilities of light-induced charge distribution could be excluded, which is usually a charge transfer process. Besides, the possibility of electrochemical potential shifts could be studied using electrochemical measurement. Fig.R3-4 displays the potentiostatic electrochemical impedance spectroscopy (PEIS) measurements for S-CoOOH separately employed in dark and under light. The Nyquist plots recorded in dark and under light nearly overlap, indicating similar interfacial impedance responses. These results demonstrate that light illumination does not induce any electrochemical potential shifts.

In addition, following the reviewer's valuable suggestion, we have additionally performed transient absorption (TA) spectroscopy of S-CoOOH using 700 nm as an excitation source (Fig.R3-5). No clear transient features are observed in the 2D TA map, except for a signal around 700 nm arising from excitation light scattering. This is reasonable because the light-induced electron transfer in our system is not a conventional photophysical excitation. In our proposed mechanism, S-CoOOH first adsorbs OH^- and undergoes deprotonation to form the $^*O$ intermediates under applied potential. Light then acts on this deprotonated $^*O$ species, generating 7-electron oxygen ($^*O^-$) (State 3, Fig.R3-6). Two adjacent $^*O^-$ subsequently undergo direct O-O coupling, which stabilizes the photoinduced state. This light-induced electron transfer is coupled to the OER process and is regarded as a part of O-O coupling (Fig.R3-6). In the absence of bias, the deprotonated $^*O$ intermediate is not formed and no light-induced electron transfer is triggered, which explain the absence of observable TA signals.

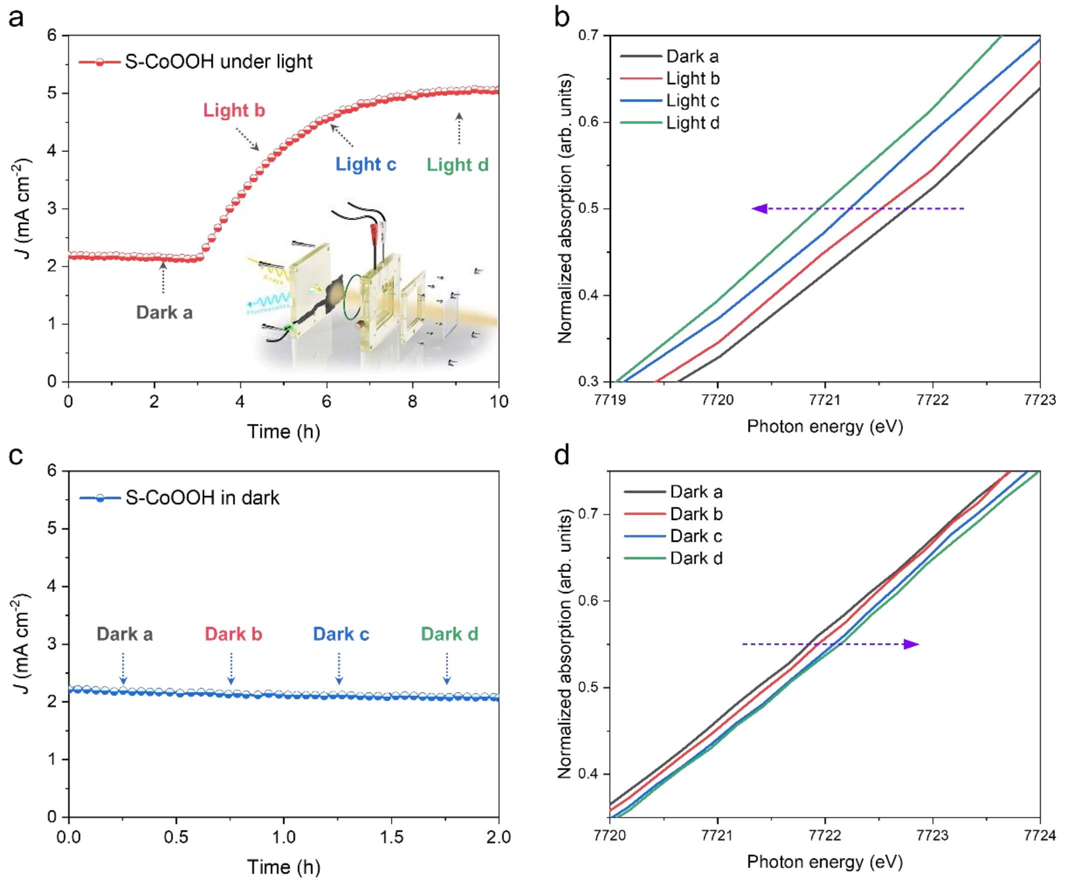

Fig.R3-1. ***Operando*** **Co** ***K*****-edge XAS measurements of S-CoOOH during OER under light and dark conditions. a** Electrochemical curve for *operando* Co *K*-edge XAS measurements recorded during OER from dark to illumination at four selected points: Dark a, Light b, Light c, and Light d. **b** Enlarged *operando* Co *K*-edge XAS spectra of S-CoOOH during OER under light near the absorption edge, with dashed purple lines revealing spectral shifts. **c** Electrochemical curve for *operando* Co *K*-edge XAS measurements in the dark, recorded during the chronoamperometry (CA) process at four selected points: Dark a, Dark b, Dark c, and Dark d. **d** Enlarged *operando* Co *K*-edge XAS spectra of S-CoOOH during OER in dark near the absorption edge, with dashed purple lines indicating spectral shifts. (Fig.2a,c and Supplementary Figs.S19b, S20b in our previous manuscript and Supplementary Information, Fig.2a,c and Supplementary Figs.S23b, S24b in our revised manuscript and Supplementary Information)

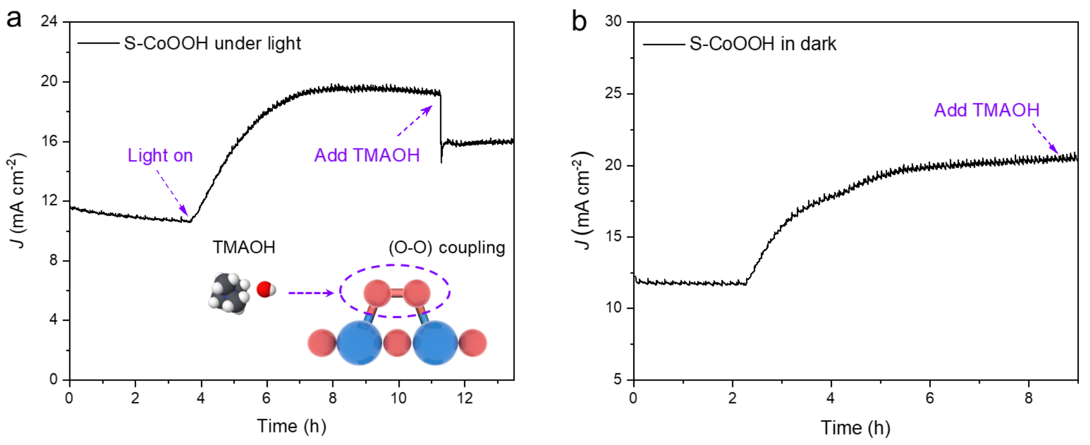

Fig.R3-2. The electrochemical curves respond to the addition of TMAOH under light (**a**) and in dark (**b**). (Fig.3a and Supplementary Fig.S23a in our previous manuscript and Supplementary Information, Fig.3a and Supplementary Fig.S28a in our revised manuscript and Supplementary Information)

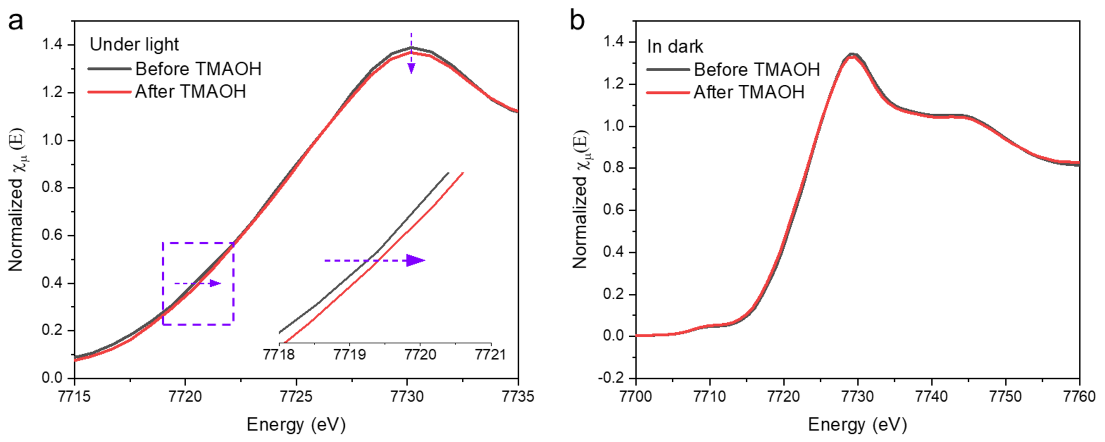

Fig.R3-3. Co *K*-edge XAS spectra of S-CoOOH recorded under light (**a**) and in dark (**b**) conditions, before and after TMAOH addition. (Fig.3b and Supplementary Fig.S23b in our previous manuscript and Supplementary Information, Fig.3b and Supplementary Fig.S28b in our revised manuscript and Supplementary Information)

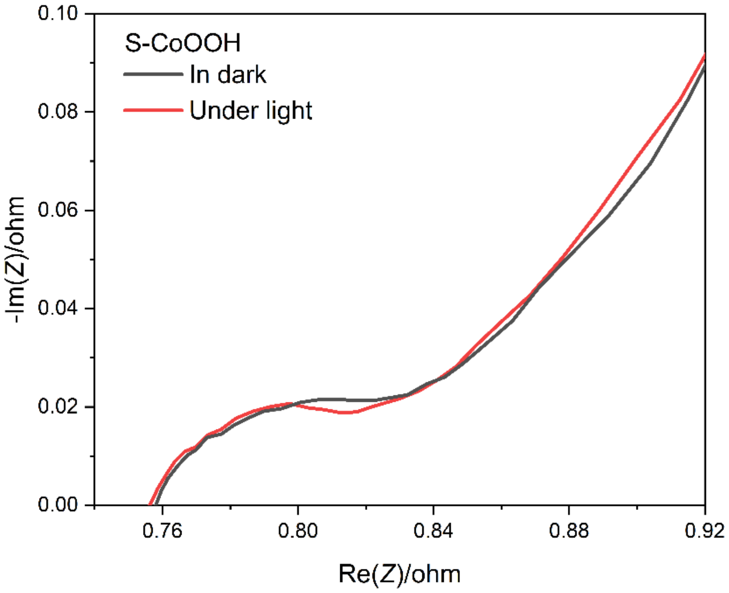

Fig.R3-4. Potentiostatic electrochemical impedance spectroscopy (PEIS) data for S-CoOOH with and without light. (Supplementary Fig.S11a in our previous Supplementary Information, Supplementary Fig.S16a in our revised Supplementary Information)

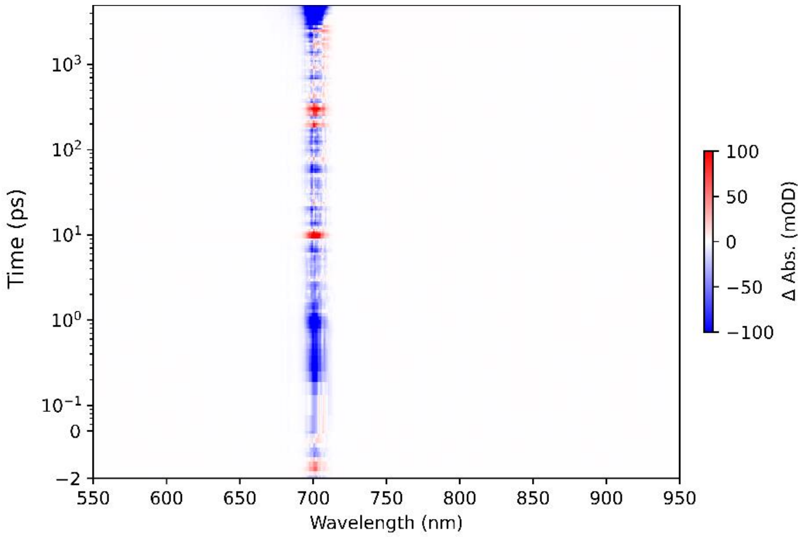

Fig.R3-5. 2D-false color plot of fs-transient absorption in 550-950 nm for S-CoOOH using 700 nm as an excitation source, with the signal around 700 nm arising from excitation light scattering. (Supplementary Fig.S25 in our revised Supplementary Information)

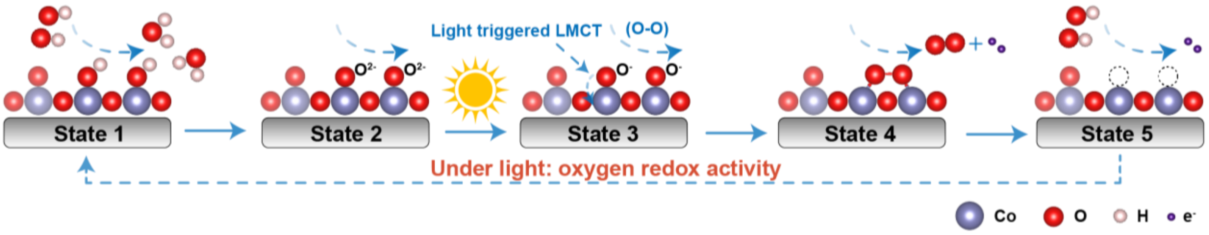

Fig.R3-6. Electron transfer pathway of S-CoOOH under light, with light-triggered electron transfer occurring during O-O coupling (State 3). (Fig.4d in our previous manuscript, Fig.4d in our revised manuscript)

To address the comment, we have incorporated the proposed modifications into our manuscript (lines 156-159, page 6, highlighted in yellow): Further evidence for LMCT is provided by combination of *operando* Co *K*-edge XAS and subsequent TMAOH chemical probe experiments, which together reveal a decrease of cobalt (electron transfer to metal) and the presence of O_{NB} states (electron transfer from ligand).

Besides, we have incorporated the proposed modifications into our manuscript (lines 113-115, pages 4-5, highlighted in yellow): Besides, the Nyquist plots recorded under light and dark nearly overlap, ruling out any electrochemical potential shifts or changes in the equilibrium interfacial potential (Supplementary Fig.S16).

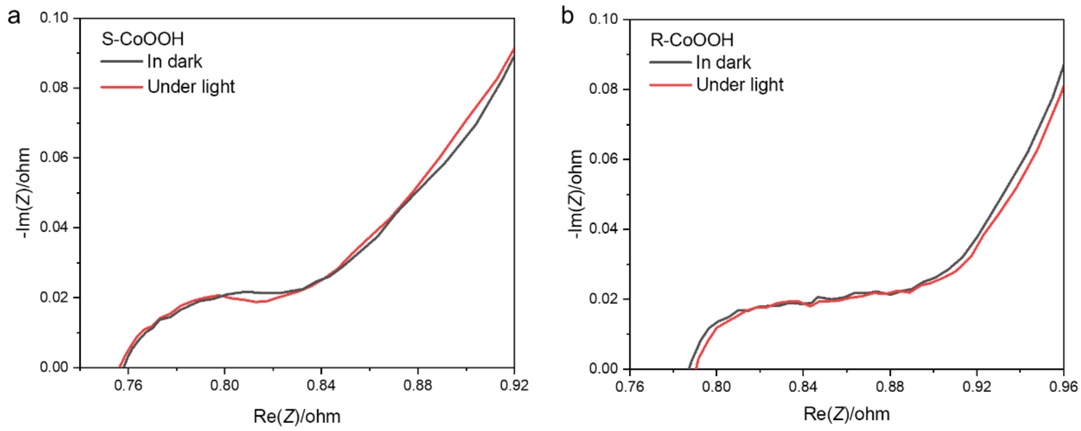

Supplementary Figure S16. Electrochemical impedance spectroscopy (EIS) data for S-CoOOH (**a**) and R-CoOOH (**b**) with and without light.

In addition, we have incorporated the proposed modifications into our manuscript (lines 154-156, page 6, highlighted in yellow): Importantly, the LMCT involved here is not a conventional photophysical LMCT,

but rather a step of OER processes that is coupled to chemical bond formation, and thus cannot be detected by transient absorption spectroscopy (Supplementary Fig.S25).

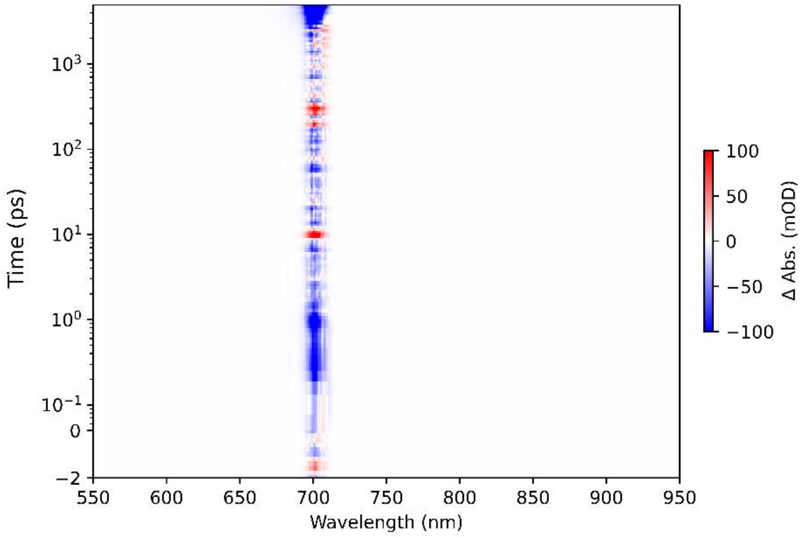

Supplementary Figure S25. 2D-false color plot of fs-transient absorption in 550-950 nm for S-CoOOH using 700 nm as an excitation source, with the signal around 700 nm arising from excitation light scattering.

The transient absorption (TA) spectroscopy of S-CoOOH was performed using 700 nm as an excitation source (Supplementary Figure S25). No clear transient features are observed in the 2D TA map, except for a signal around 700 nm arising from excitation light scattering. This is reasonable because the light-induced electron transfer in our system is not a conventional photophysical excitation. In our proposed mechanism, S-CoOOH first adsorbs OH^- and undergoes deprotonation to form the $^*O$ intermediates under applied potential. Light then acts on this deprotonated $^*O$ species, generating 7-electron oxygen ($^*O^-$). Two adjacent $^*O^-$ subsequently undergo direct O-O coupling, which stabilizes the photoinduced state. This light-induced electron transfer is coupled to the OER process and is regarded as a part of O-O coupling step. In the absence of bias, the deprotonated $^*O$ intermediate is not formed and no light-induced electron transfer is triggered, which explain the absence of observable TA signals.

2. Although the authors state that illumination increases the reaction temperature by less than 2 °C, this measurement alone cannot conclusively exclude the influence of photothermal effects. Nanoscale “hot spots” or localized heating in the catalyst layer could alter reaction kinetics or electronic states even under seemingly mild macroscopic temperature changes. Furthermore, localized light absorption could induce

structural strain or interfacial polarization, both of which can mimic photoelectronic enhancement. The method used for temperature monitoring is inadequate to assess microscale temperature gradients. Therefore, additional characterization such as photothermal IR mapping et.al would help to verify whether illumination triggers genuine electronic effects rather than subtle thermal or structural perturbations.

Response: In response to the reviewer's insightful suggestion, we have added photothermal IR mapping of S-CoOOH during OER under light (Fig.R3-7), in which the sample region is marked by a blue box. The corresponding temperature histogram of this region (Fig.R3-8) reveals a spatially uniform temperature distribution across the entire catalyst layer (22.7-23.3 °C), with no discernible localized "hot spots" or localized heating. Current characterization techniques make it challenging to direct probe nanoscale "hot spots"; however, the photothermal IR mapping shows no detectable microscale thermal inhomogeneity, which partially suggests that illumination does not induce significant localized heating.

Moreover, if the observed current enhancement is due to localized photothermal heating, a continuous increase in temperature and correspondingly gradual changes in catalytic performance would be expected. However, this behavior is inconsistent with our chronoamperometric (CA) results under light (Fig.R3-9). After stabilization, the catalytic performance remains nearly unchanged during continuous light irradiation. Besides, upon switching off light, the current immediately exhibits a downward trend, and following a stabilization period, gradually returns to its original pre-illumination value. This timely and reversible response is inconsistent with a photothermal effect, as the photothermal effect typically involves slower thermal accumulation, diffusion, and relaxation processes.

In addition, no evidence of structural strain or interfacial polarization is observed in our sample S-CoOOH during OER under light, as supported by *operando* Co *K*-edge X-ray absorption spectra (XAS) and potentiostatic electrochemical impedance spectroscopy (PEIS) measurements between dark and light conditions. Because no structural strain or interfacial polarization is detected, the presence of localized heating in our sample can be indirectly excluded.

a) No structural strain is detected during illumination, as supported by *operando* Co *K*-edge XAS showing nearly no distinct changes in Co-O or Co-Co bond lengths upon light irradiation. The *operando* Co *K*-edge Fourier transformed extended X-ray absorption fine structure (FT-EXAFS) spectra of S-CoOOH

were collected during OER under light irradiation, at four selected points marked in the electrochemical curves (Fig.R3-10a), denoted as Dark a, Light b, Light c, and Light d. As displayed in Fig.R3-10b, no detectable shift is observed for the Co-O and Co-Co bond peaks, indicating that the corresponding bond lengths remain negligible changes. This is further supported by the fitted bond length values (R) summarized in Table R3-1, which show no significant variation under light.

b) Interfacial polarization is also excluded by potentiostatic electrochemical impedance spectroscopy (PEIS) measurements for S-CoOOH separately employed in dark and under light (Fig.R3-11). The PEIS spectra obtained under both dark and light conditions nearly overlap. These results indicate that light irradiation does not induce a change in the equilibrium interfacial potential.

In this regard, we sincerely appreciate the reviewer for this valuable comment. Based on our current experimental evidence, we consider it unlikely that illumination generates localized heating within our sample in our electrochemical cell connected to a thermostatic bath (Fig.R3-12). However, whether nanoscale hot spots exist remains an ongoing question and warrants further investigation in the future.

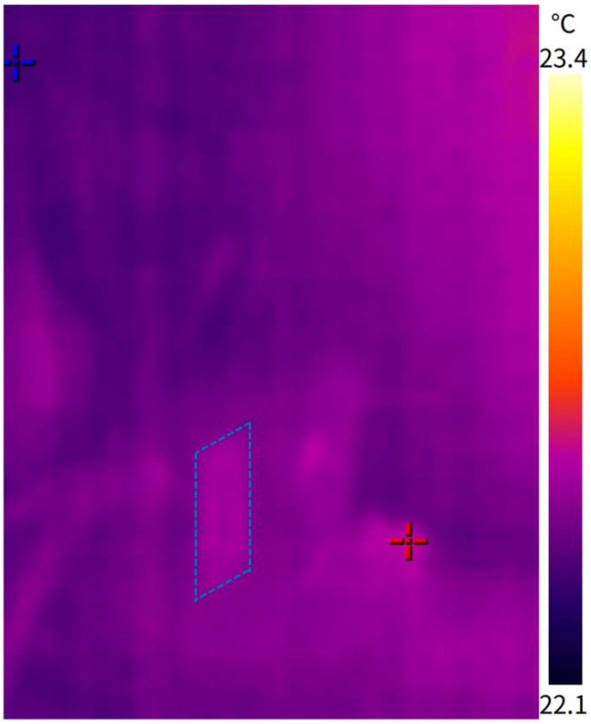

Fig.R3-7. Photothermal IR mapping of S-CoOOH during OER under light, with the region enclosed by blue box corresponding to S-CoOOH sample based on carbon cloth substrate. (Supplementary Fig.S11a in our revised Supplementary Information)

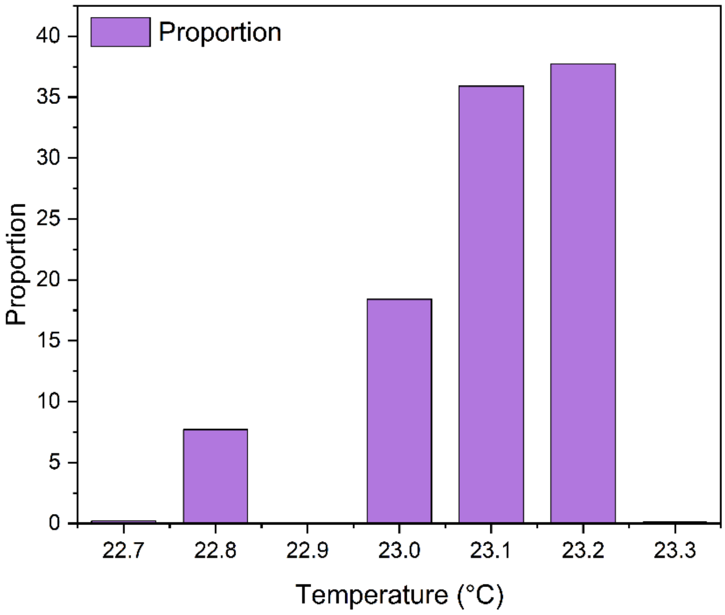

Fig.R3-8. Temperature histogram of S-CoOOH based on carbon cloth, highlighted by blue box in Fig.R3-7. (Supplementary Fig.S11b in our revised Supplementary Information)

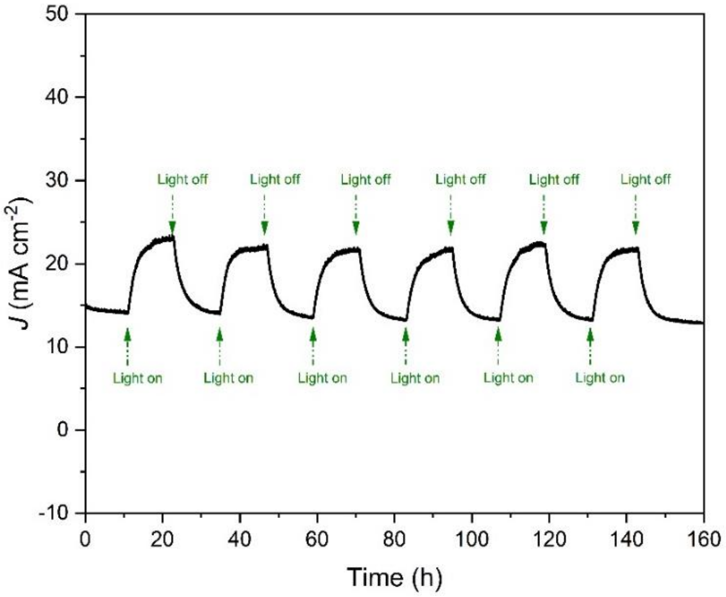

Fig.R3-9. Repeatability of the light on/off response behavior during chronoamperometric (CA) measurements of S-CoOOH. (Supplementary Fig.S3 in our previous Supplementary Information, Supplementary Fig.S3 in our revised Supplementary Information)

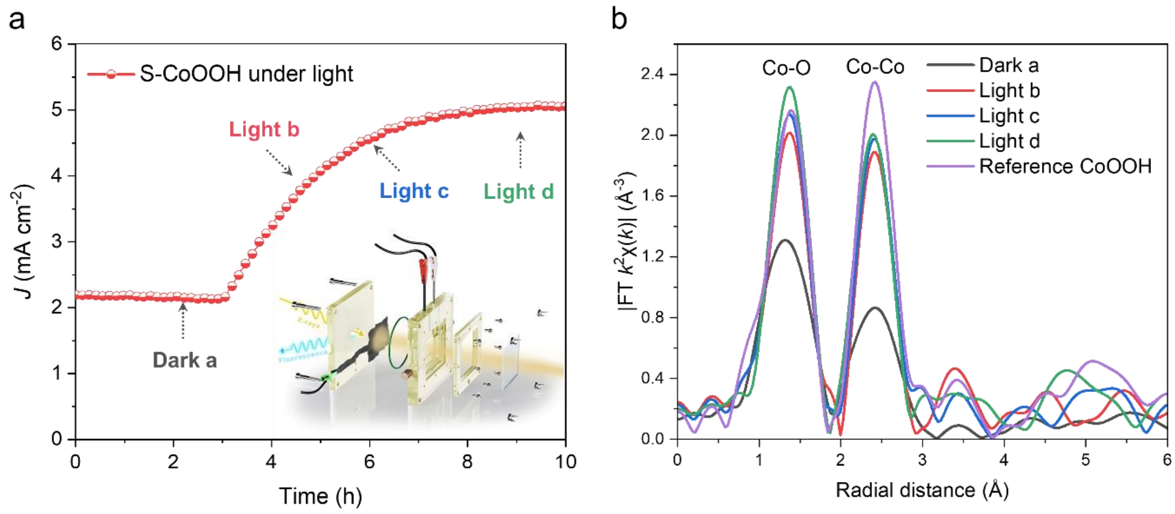

Fig.R3-10. a Electrochemical curve for *operando* Co *K*-edge XAS measurements recorded during OER from dark to illumination at four selected points: Dark a, Light b, Light c, and Light d. **b** Normalized Co *K*-edge Fourier transformed extended X-ray absorption fine structure (FT-EXAFS) spectra, collected during OER under light irradiation (Dark a, Light b, Light c, and Light d), with Reference CoOOH used as the standard. (Fig.2a and Supplementary Fig.S22 in our previous manuscript and Supplementary Information, Fig.2a and Supplementary Fig.S27 in our revised manuscript and Supplementary Information)

Table R3-1. FT-EXAFS fitting results of S-CoOOH collected during OER under light irradiation (Dark a, Light b, Light c, and Light d), with Reference CoOOH used as the standard. CN is the coordination number, σ^2 is the Debye-Waller factor, ΔE_0 is the shift in absorption edge energy, R is the bond length, and R-factor means Residual factor. (Supplementary Table S3 in our previous Supplementary Information, Supplementary Table S3 in our revised Supplementary Information)

Sample	path	CN	σ^2	ΔE_0	R	R-factor
Reference CoOOH	Co-O	6	0.0023	-3.4	1.90±0.01	0.0055
	Co-Co	6	0.0045	-4.5	2.85±0.01	
S-CoOOH at Dark a	Co-O	4.97±0.48	0.0130	-6.7	1.87±0.01	0.0183
	Co-Co	4.18±0.76	0.0170	3.9	2.91±0.04	
S-CoOOH at Light b	Co-O	5.78±0.58	0.0046	-2.2	1.89±0.02	0.0153
	Co-Co	4.43±0.55	0.0052	-2.0	2.85±0.02	
S-CoOOH	Co-O	5.90±0.42	0.0040	-3.5	1.88±0.01	0.0068

at Light c	Co-Co	4.83±0.42	0.0055	-1.4	2.84±0.02	
S-CoOOH	Co-O	5.97±0.50	0.0030	-4.3	1.88±0.02	0.0138
at Light d	Co-Co	5.16±0.57	0.0059	-3.7	2.83±0.02	

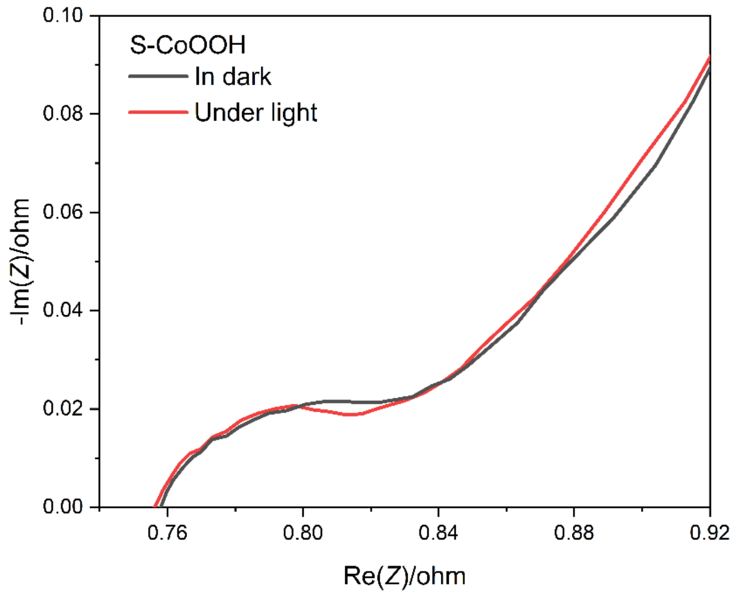

Fig.R3-11. Potentiostatic electrochemical impedance spectroscopy (PEIS) data for S-CoOOH with and without light. (Supplementary Fig.S11a in our previous Supplementary Information, Supplementary Fig.S16a in our revised Supplementary Information)

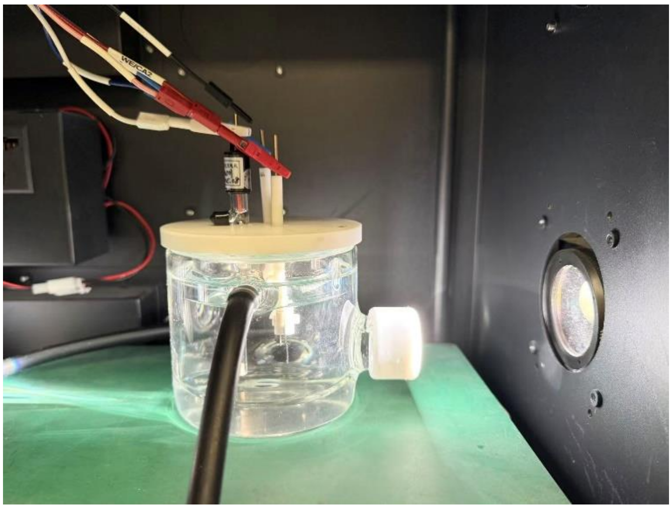

Fig.R3-12. Photograph of the actual thermostatic bath connected to an electrochemical cell. (Supplementary Fig.S9 in our previous Supplementary Information, Supplementary Fig.S9 in our revised Supplementary Information)

To address the comment, we have incorporated the proposed modifications into our manuscript (lines 110-115, pages 4-5, highlighted in yellow): Initially, several potential reasons for the distinctive increase in OER performance under light illumination are excluded, including thermal effect (22.7-23.3 °C across the entire catalyst layer), changes in grain size, generation of light-induced holes, or any additional reaction (Supplementary Figs.S9-S15). Besides, the Nyquist plots recorded under light and dark nearly overlap, ruling out any electrochemical potential shifts or changes in the equilibrium interfacial potential (Supplementary Fig.S16).

Moreover, we have included thermal IR mapping and the accompanying content in our supplementary information (Supplementary Figure S11): Supplementary Figure S11a presents photothermal IR mapping of S-CoOOH during OER under light, in which the sample region is marked by a blue box. The corresponding temperature histogram of this region (Supplementary Figure S11b) reveals a spatially uniform temperature distribution across the entire catalyst layer (22.7-23.3 °C), with no discernible localized "hot spots" or localized heating. Current characterization techniques make it challenging to direct probe nanoscale "hot spots"; however, the photothermal IR mapping shows no detectable microscale thermal inhomogeneity, which partially suggests that illumination does not induce significant localized heating. The existence of nanoscale hot spots remains an ongoing question and warrants further investigation in the future.

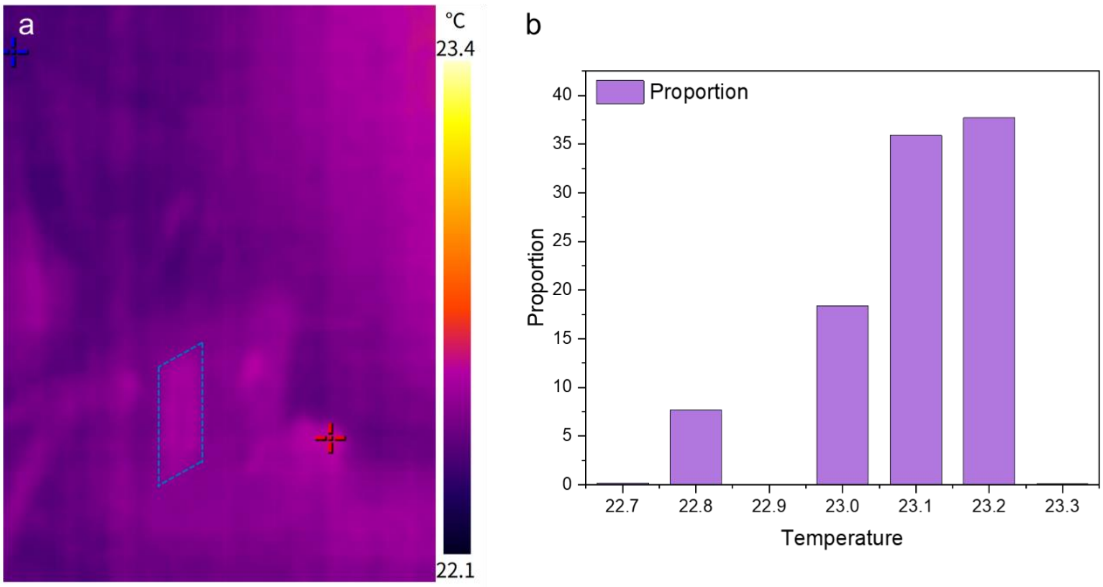

Supplementary Figure S11. a Photothermal IR mapping of S-CoOOH during OER under light, with the region enclosed by blue box corresponding to S-CoOOH sample based on carbon cloth substrate. **b** Temperature histogram of S-CoOOH based on carbon cloth, highlighted by blue box in Supplementary

Figure S11a.

In addition, we have incorporated the proposed modifications into our manuscript (lines 170-171, page 7, highlighted in yellow): Besides, no distinct changes in Co-O or Co-Co bond lengths are observed upon irradiation, suggesting no structural strain.

3. The theoretical calculations in the manuscript are presented as evidence supporting the experimentally observed light effects; however, they appear to be limited to ground-state DFT without explicit treatment of the photoexcited electronic structure. In this form, the calculations primarily reveal static charge distributions and adsorption energetics, which cannot directly account for transient electron transfer induced by illumination. To justify the link between LMCT and catalytic enhancement, the authors should explain how the computed charge density differences and reaction barriers reflect photoinduced electron migration.

Response: We have attempted to explore several excited-state computational approaches, including linear-response time-dependent DFT (LR-TDDFT) and constrained DFT. However, achieving a quantitatively reliable description of the excited states in our sample S-CoOOH remains highly challenging due to the large periodic supercell (434 atoms, comprising 108 Co, 108 H, and 218 O atoms). Such excited-state methods are typically applicable to models containing fewer than ~50 atoms. Besides, the intrinsic magnetic nature of the Co centers introduces multiple competing spin configurations, which further compromise the robustness and reliability of the excited-state calculations.

On this basis, we adopted a simplified approach to perform approximate excited-state calculations for evaluating the energy barrier of O-O coupling. In this case, we changed the total number of electrons in the system. Such as, we add one more electron into the supercell model for 7-electron O. As confirmed by ^{18}O isotope labeling and TMAOH experiments, such 7-electron O atoms are generated during light-assisted OER and undergo direct O-O coupling. Based on this model, our calculations further elucidate the specific sites where O-O coupling preferentially occurs following the formation of light-induced 7-electron O, which is in good agreement with our differential electrochemical mass spectrometry (DEMS) experimental results.

As discussed above, excited-state DFT methods are not applicable to our system; therefore, direct computation of excited-state charge density differences and reaction barriers is not feasible. Nevertheless, in order to address the reviewer's important suggestions, we have calculated the Gibbs free energy profiles under both dark and light conditions, using the described above simplified computational approach (Fig.R3-13). These results indicate that the OER pathway under light exhibits a smaller theoretical overpotential (1.57 V) compared to that in dark (1.72 V), with the rate-determining step shifting from *OOH formation in dark to proton transfer under light. It suggests that oxygen redox activity becomes thermodynamically more favorable and thus more readily proceeds when exposed to light.

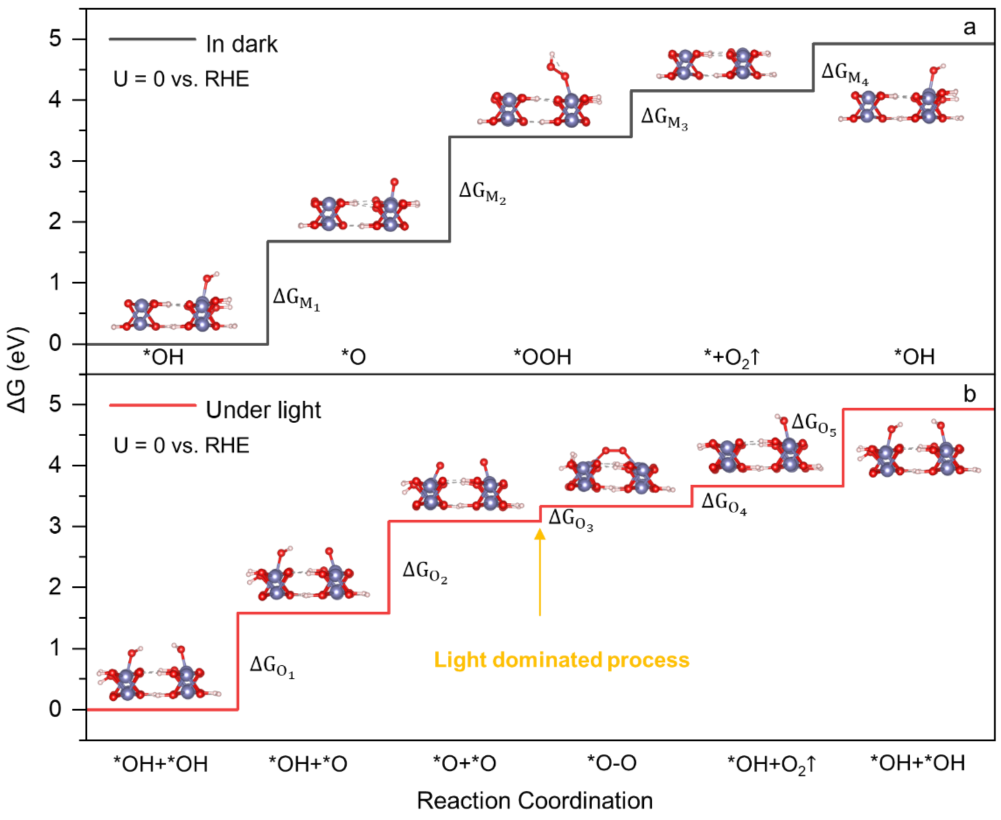

Fig.R3-13. Calculated reaction free energy diagrams of S-CoOOH in dark (a) and under light (b) on 0 V vs. RHE (where * refers to the metal sites). (Supplementary Fig.S37 in our revised Supplementary Information)

To address the comment, we have marked the light dominated process with a yellow arrow in Supplementary Fig.S37b, and have added the accompanying content below this figure: The role of light is manifested at the O-O coupling step (*O+*O→*O-O, marked in a yellow arrow). Under light illumination, electrons are transferred from deprotonated *O to Co, generating 7-electron oxygen ($^*O^-$); then, two adjacent ones couple to form direct O-O coupling (*O-O), thereby stabilizing the oxygen intermediates. The thermodynamic

lowering of the rate-determining step, from 1.72 V for *OOH formation in dark to 1.57 V for proton transfer under light, reflects that light favors a reaction pathway involving direct O-O coupling. This shift provides indirect computational evidence supporting light-triggered electron transfer.

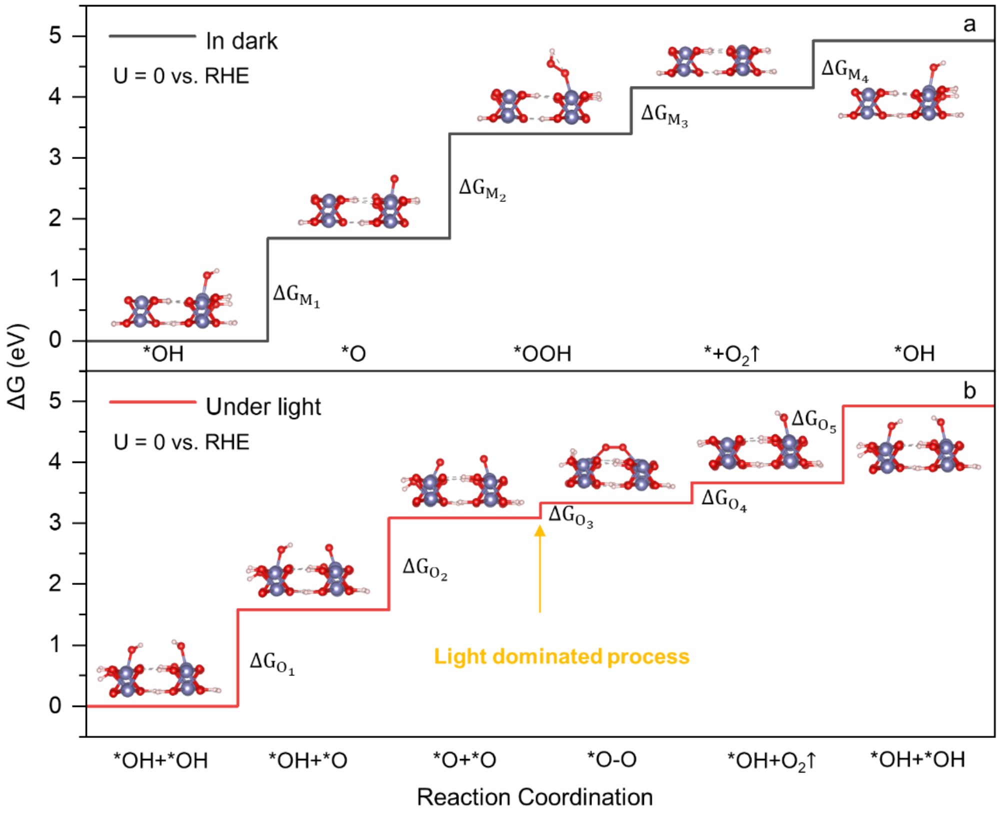

Supplementary Figure S37. Calculated reaction free energy diagrams of S-CoOOH in dark (**a**) and under light (**b**) on 0 V vs. RHE (where * refers to the metal sites).

4. In response to the first reviewer's first question, the authors claim that operando XAS confirms the structural stability of the Co oxyhydroxide catalyst under illumination. While this technique provides valuable information on the local coordination environment, it cannot entirely rule out surface amorphization or subtle lattice reconstruction, especially in the near-surface region where catalysis occurs. The observed similarity in average Co-O coordination number and bond length does not necessarily imply that the surface remains intact. It would be helpful if the authors could include or reference complementary structural characterizations such as post-reaction TEM, operando Raman, or XRD measurements to demonstrate that no phase transformation or surface reorganization accompanies the photo assisted process. Without such

verification, it remains possible that part of the observed activity change arises from illumination-induced structural dynamics rather than pure electronic effects.

Response: Both surface amorphization and subtle lattice reconstruction are irreversible, whereas the light-induced enhancement in electrochemical performance and the associated structural changes observed in our work are completely reversible, indicating that the observed behavior cannot be attributed to these irreversible transformations. Following the reviewer's suggestions, we have supplemented post-reaction TEM and XRD measurements, which further confirm that no phase transformation or surface reorganization occurs during OER under light.

Fig.R3-14 displays XRD patterns of S-CoOOH before and after light-assisted OER test. Prior to the OER test, S-CoOOH was subjected to a 10h electrochemical activation, during which cobalt sulfide pre-catalyst is fully reconstructed into rhombohedral β-CoOOH (space group *R-3m*, JCPDS No. 07-0169), as evidenced by the XRD pattern (bottom black curve). The diffraction peaks at 20.24°, 36.98°, 38.89°, 50.58°, 65.34°, and 69.17° can be indexed to (003), (101), (012), (015), (110), and (113) crystal planes of β-CoOOH, respectively. The diffraction peak at 25.83° can be attributed to (002) crystal plane of carbon cloth substrate. After the light-assisted OER test, the XRD pattern (top red curve) exhibits neither discernible shifts in peak positions nor the emergence of new diffraction peaks, indicating that the catalyst remains in β-CoOOH phase without detectable phase transformation.

Besides, we now provide the high-resolution transmission electron microscopy (HR-TEM) images of S-CoOOH collected before and after light-assisted OER process (Fig.R3-15). The images show comparable particle sizes and morphologies before and after OER, without the appearance of any additional surface layer, thereby excluding significant surface reorganization.

In addition, we appreciate the reviewer's suggestion regarding *operando* Raman measurements. However, in our work light-assisted OER process, *operando* Raman measurement is not experimentally feasible. First, the coexistence of the Raman excitation laser and continuous external illumination may introduce unavoidable optical interference and elevated background signals, making reliable Raman signal extraction highly challenging. Second, the geometric constraints of *operando* electrochemical Raman cells make it

challenging to simultaneously maintain stable electrolysis and homogeneous illumination. Therefore, *operando* Raman is not suitable for investigating the light-assisted OER process in our work.

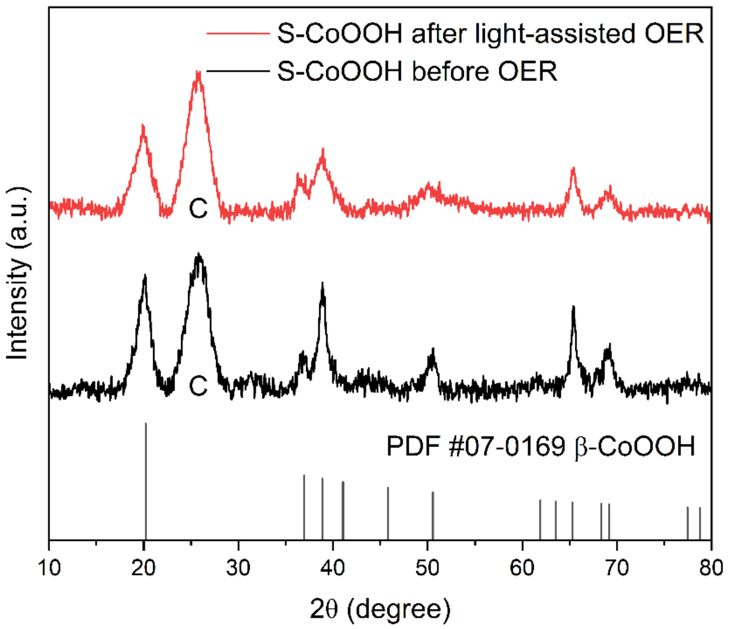

Fig.R3-14. XRD patterns of S-CoOOH based on carbon cloth before (bottom) and after (top) light-assisted OER test, with standard β-CoOOH listed (PDF #07-0169). (Supplementary Fig.S19 in our revised Supplementary Information)

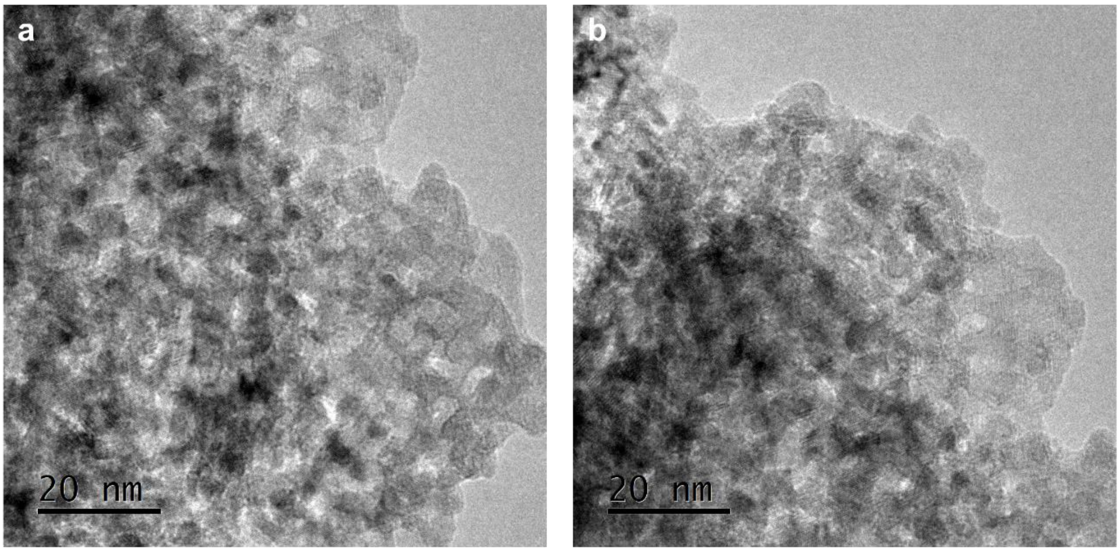

Fig.R3-15. The high-resolution transmission electron microscopy (HR-TEM) images of S-CoOOH before (**a**) and after (**b**) light-assisted OER test. (Supplementary Fig.S20 in our revised Supplementary Information)

To address the comment, we have incorporated the proposed modifications into our manuscript (lines 118-121, page 5, highlighted in yellow): Additionally, structural reconstruction is also excluded based on pre- and post-reaction Co *K*-edge XAS spectra, XRD patterns, and TEM images, as all results before and after OER under light irradiation remain nearly identical (Supplementary Figs.S18-20).

Moreover, we have revised the content accompanying Supplementary Fig.S18 (previously Supplementary Fig.S16) in our supplementary information: Structural reconstruction is also excluded. As displayed in Supplementary Fig.S18, normalized Co *K*-edge XAS spectra of S-CoOOH before and after OER under light irradiation are nearly identical, indicating local structural stability under light. Besides, pre- and post-reaction XRD patterns and TEM images of S-CoOOH (Supplementary Figs.S19, S20) confirm that no phase transformation or surface reorganization occurs during OER under light. In addition, during the OER test under light irradiation, S-CoOOH exhibits reversible behavior in electrochemical performance, further confirming that no additional structural reconstruction occurs (Supplementary Fig.S3).

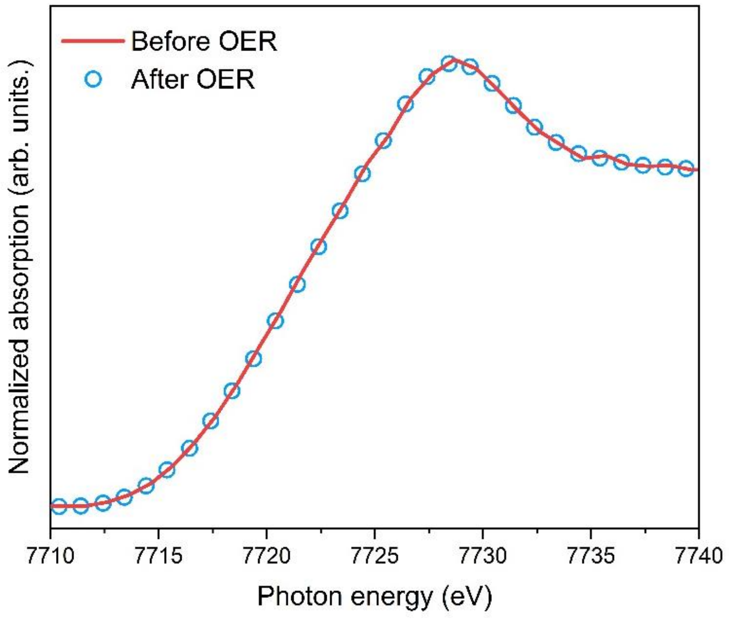

Supplementary Figure S18. Normalized Co *K*-edge XAS spectra of S-CoOOH before and after OER under light irradiation.

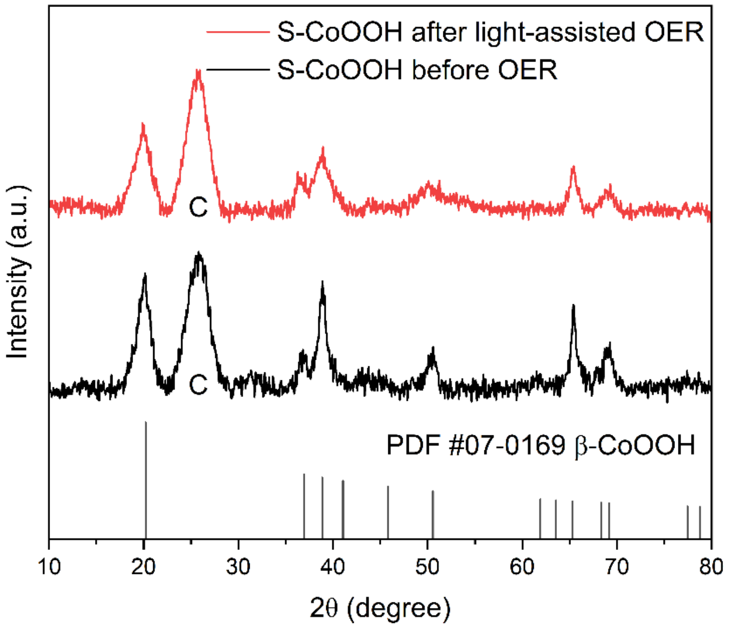

Supplementary Figure S19. XRD patterns of S-CoOOH based on carbon cloth before (bottom) and after (top) light-assisted OER test, with standard β-CoOOH listed (PDF #07-0169).

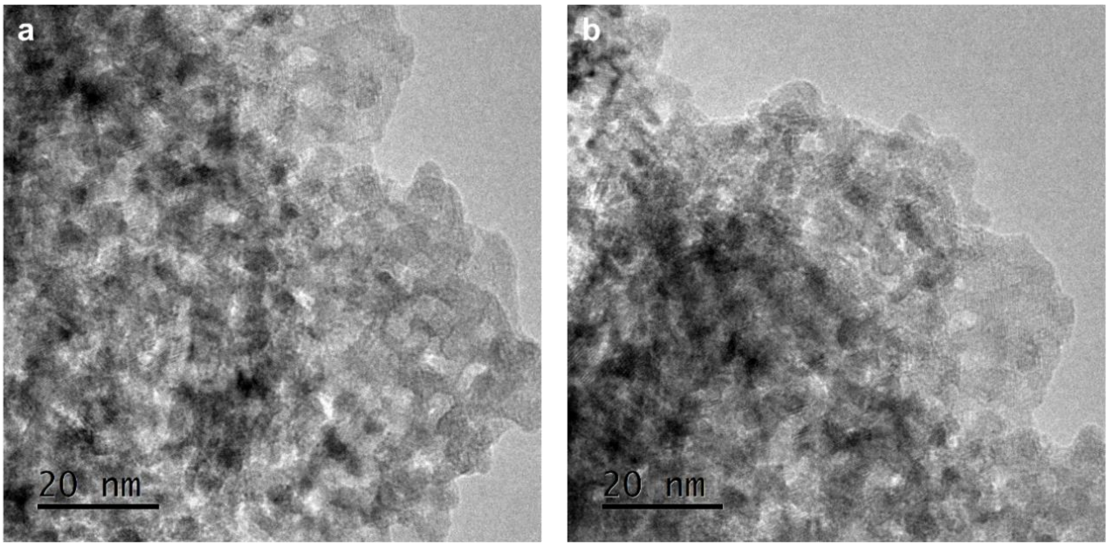

Supplementary Figure S20. The high-resolution transmission electron microscopy (HR-TEM) images of S-CoOOH before (**a**) and after (**b**) light-assisted OER test.

5. In response to the fourth question raised by the third reviewer, the authors repeatedly emphasize that CI-NEB and DOS analyses were performed based on first-principles calculations; however, all computations were conducted in vacuum, and the structural models employed are not fully consistent with those used in

previous studies. The justification that “the differences from earlier results are negligible” is therefore unconvincing. For the oxygen evolution reaction (OER) system, solvent polarization and interfacial electric fields play a crucial role in stabilizing intermediates and influencing the O-O coupling barrier. Calculations performed under vacuum conditions may overestimate the energy barriers or misrepresent the reaction mechanism. It is thus recommended that the authors at least include an implicit solvation model (e.g., VASPsol) or apply surface charge corrections to validate their results and improve the physical reliability and comparability of the conclusions.

Response: To address the reviewer’s concern that our calculation in vacuum might misrepresent the reaction mechanism, we have compared the energy differences along the entire OER pathway under dark and light conditions (Fig.R3-16). All calculated Gibbs free energy profiles were carried out using VASPsol model to explicitly include solvent effects. As discussed above in response to Question R3-3, these results indicate that the OER pathway under light exhibits a smaller theoretical overpotential (1.57 V) compared with that in dark (1.72 V). The rate-determining step shifts from *OOH formation in dark to proton transfer under light, in good agreement with the experimental results.

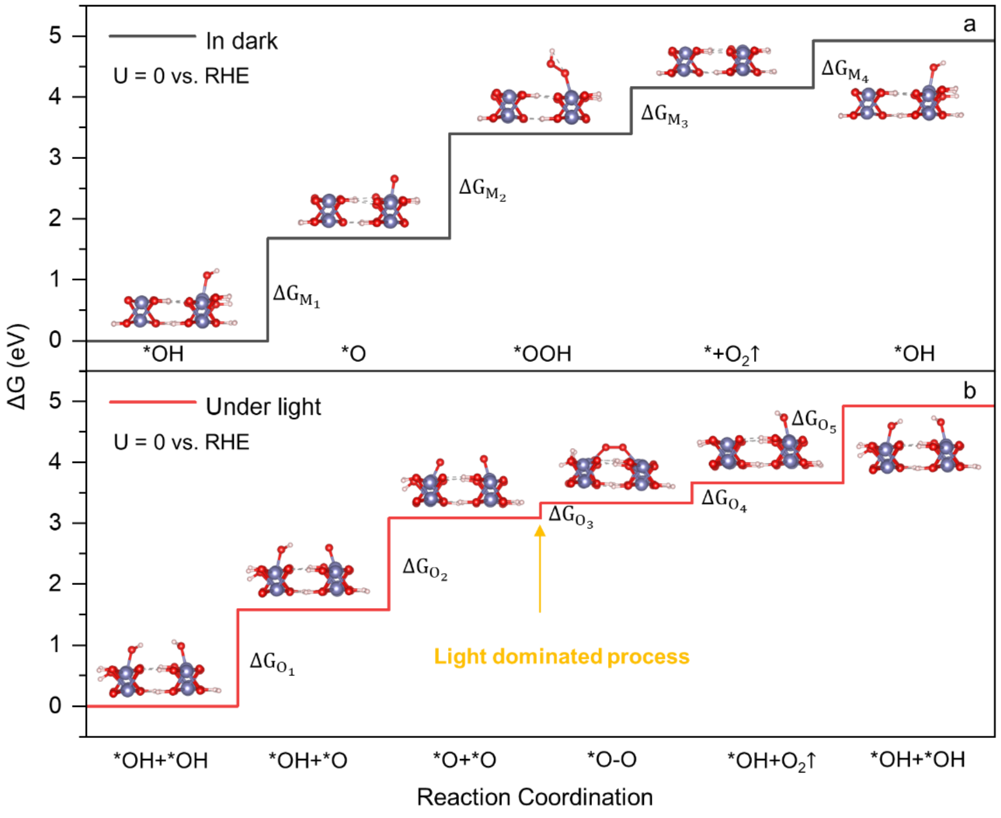

Fig.R3-16. Calculated reaction free energy diagrams of S-CoOOH in dark (a) and under light (b) on 0 V vs. RHE (where * refers to the metal sites). (Supplementary Fig.S37 in our revised Supplementary Information)

To address the comment, we have incorporated the proposed modifications into our manuscript (lines 531-534, page 21, highlighted in yellow): Thermodynamics calculations were performed using the optPBE-vdW functional with the van der Waals (vdW) correction. The solvent effect was considered using the implicit solvation model implemented in the VASP model, with the free energies referenced to U = 0 V vs. RHE.

Response to referees

We thank the three referees for taking the time to carefully review the manuscript and for giving many useful suggestions. The manuscript has been revised according to their comments and the changes have been highlighted in the revised version. We feel the quality of the paper has been improved greatly thanks to the input from the referees. Below is a point-by-point response.

Reviewer #1

Remarks to the Author: Most of problems have been addressed, but some points still need to be clarified.

1. Based on your responses, the PEIS for S-CoOOH display similar results in dark and under light irradiation. It is difficult to understand this point. The adsorbate configuration at catalyst surface should be changed by light-driven transformation, why the PEIS values are so such alike?

Response: The slight difference in PEIS values between dark and illuminated conditions is reasonable, since the effect of light on adsorption configurations involves **only a small fraction of the overall Co-O unit cells.** The detailed discussion is shown below:

a) **The effect of light on the adsorption configurations is limited to a specific step, rather than the whole OER pathway.** In our previous response to Q1-6, although light can influence adsorbate configurations, this effect is limited to O-O coupling step ($^{*}O+^{*}O \rightarrow ^{*}O\text{-}O$), rather than the entire OER process (Fig.R1-1). Therefore, we compared adsorption configurations only before and after this step (State 3 to State 4, in Fig.R1-2), rather than across the full OER pathway. Moreover, this light-induced O-O coupling does not involve interfacial electron transfer to the external circuit (Fig.R1-2), and thus has a minimal impact on the overall charge transfer resistance.

b) **Light only affects a small fraction of edge-confined high-spin Co^{3+} sites in S-CoOOH.** The light-induced transformation originates from coordinatively unsaturated Co atoms at the edge, which leads to a high-spin configuration and causes the splitting of Co 3*d* and 4*s* orbitals, thereby enabling light-triggered electron transfer. In our previous work, the proportion of high-spin Co^{3+} in S-CoOOH is small, only approximately 15 % based on M-T curves (Supplementary Fig.S19, *Nat. Commun.* 2024, 15, 1383).

c) **The light-induced electronic structural change in S-CoOOH is reversible,** as confirmed by nearly identical Co *K*-edge XAS spectra before and after OER (Fig.R1-3), indicating no structural reconstruction. As a result, the associated changes in adsorption configurations are also reversible and thereby induce minimal alteration of the catalyst-electrolyte interface.

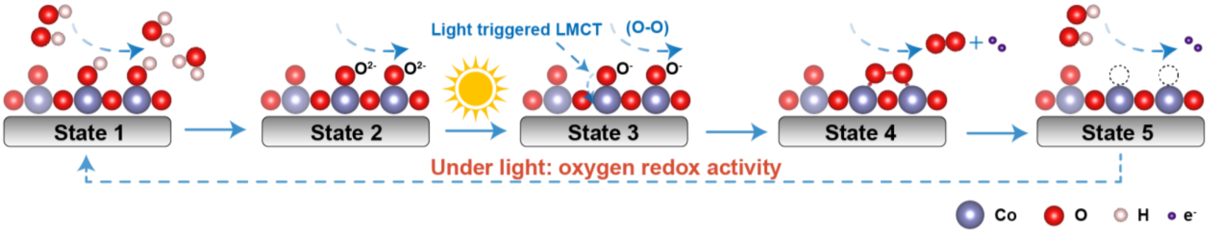

Fig.R1-1. Electron transfer pathway of S-CoOOH under light, with light-triggered electron transfer occurring during O-O coupling. (Fig.4d in our previous manuscript, Fig.4d in our revised manuscript)

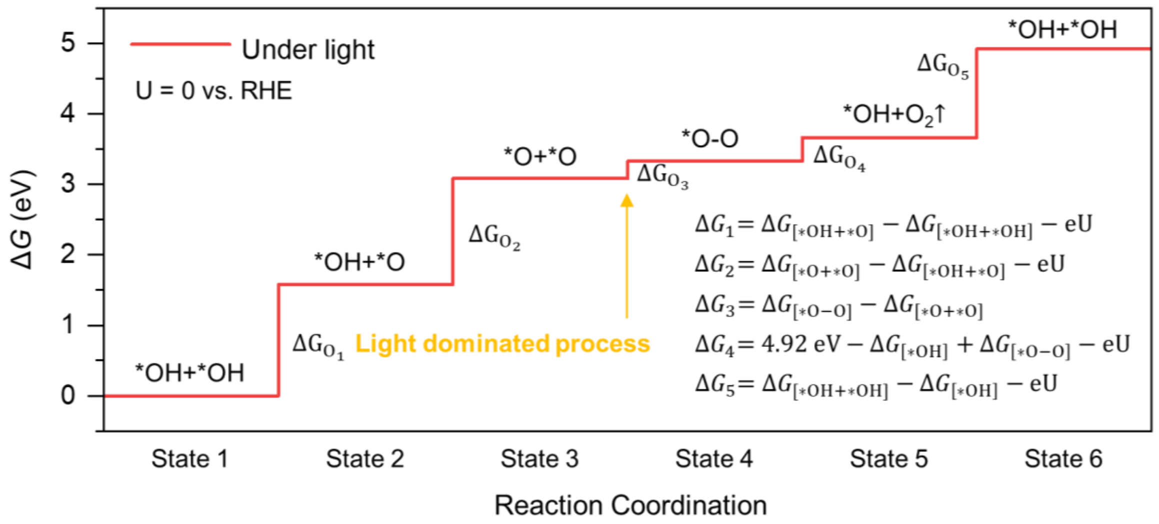

Fig.R1-2. Calculated reaction free energy diagram of S-CoOOH under light on U = 0 V vs. RHE, showing that light dominated process occurs at the O-O coupling step. (Supplementary Fig.S37b in our previous Supplementary Information, Supplementary Fig.S38a in our revised Supplementary Information)

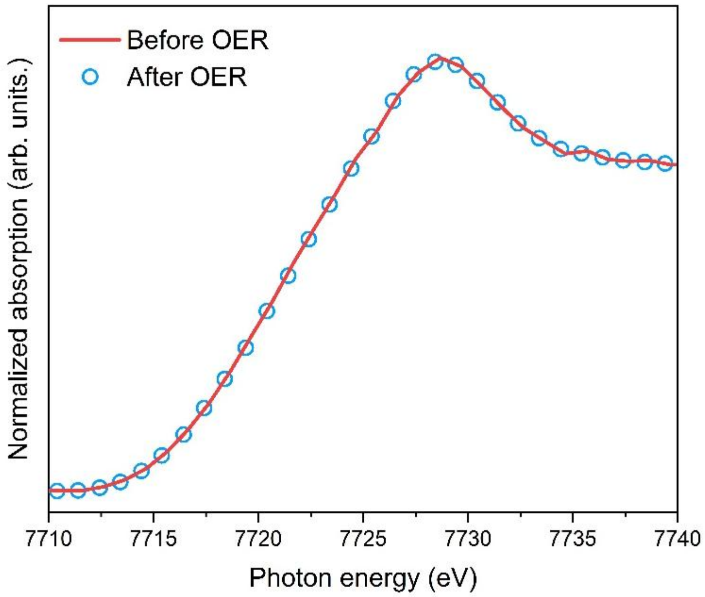

Fig.R1-3. Normalized Co *K*-edge XAS spectra of S-CoOOH before and after OER under light irradiation. (Supplementary Fig.S18 in our previous Supplementary Information, Supplementary Fig.S18 in our revised Supplementary Information)

To address the comment, we have incorporated the proposed modifications below Supplementary Figure S17: The slight difference in PEIS values between dark and illuminated conditions can be attributed to the fact that the effect of light on the adsorption configurations involves only a small fraction of the overall Co-O unit cells, due to the limited fraction of high-spin Co^{3+} species in S-CoOOH. Moreover, the effect of light on the adsorption configurations is limited to a specific step and is reversible, rather than affecting the whole OER process. Therefore, light induces minimal alteration of the overall catalyst-electrolyte interface, resulting in similar resistance values under dark and illuminated conditions.

2. In addition, how to implant the light irradiations into the industrial electrolyzer?

Response: Direct implementation of light irradiation in conventional industrial electrolyzers remains technically challenging, as typical electrolyzers employ opaque cover materials that prevent light from eventually reaching the catalyst (Fig.R1-4a). While these cover materials could in principle be replaced with glass or other transparent materials, light would still need to penetrate the current collector and catalyst layer, which are difficult to make transparent (Fig.R1-4b). Therefore, integrating light irradiation into conventional electrolyzer stacks is not straightforward.

However, an alternative strategy is to adopt a redox-decoupled water splitting device proposed by Prof. Wang Qing, which provides a possible route to introduce light irradiation into a specially designed industrial electrolyzer (*J. Am. Chem. Soc.* 2021, 143, 223-231; *Nature* 2022, 611, 702-708). This system enables the decoupling of HER and OER from the electrodes to spatially separated catalyst bed reactions via a pair of redox mediators (Fig.R1-5). Unlike conventional electrolyzer stacks, oxygen evolution in this device occurs in an independent oxygen evolution tank that can be constructed from transparent materials, enabling light irradiation to access the catalyst. In addition, the electrocatalyst powders can be directly dispersed in the electrolyte rather than coated on a substrate, which facilitates more effective utilization of light and allows photons to trigger oxygen redox activity. Therefore, our proposed light-triggered oxygen redox activity could potentially be implemented in this redox-decoupled water splitting technology. Motivated by this concept, in our future work, we plan to further explore the integration of our proposed light-triggered oxygen redox activity into such a redox-decoupled water splitting device.

[FIGURE REDACTED]

Fig.R1-4. Schematic diagram of a conventional electrolyzer stack. **a** Digital photograph of a conventional electrolyzer stack device (image retrieved from Fuel Cell Store). **b** Components of a conventional electrolyzer stack (ref: *ACS Sustainable Chem. Eng.* 2018, 6, 4829-4837).

[FIGURE REDACTED]

Fig.R1-5. Illustration of our proposed light-triggered oxygen redox activity in decoupled-redox water splitting devices. (ref: *J. Am. Chem. Soc.* 2021, 143, 223-231)

To address the comment, we have incorporated the proposed modifications into our manuscript (lines 378-381, page 17, highlighted in yellow): This work elucidates the role of light in enhancing the OER performance of metal oxyhydroxide-based alkaline catalysts, and such light-induced effect shows promising potential for extension to acidic catalysts that share a similar octahedral structure and to industrial electrolyzers.

3. In the DFT calculation, the contribution of pH values should be considered. The U values should be reconsidered to reevaluate their rate-limiting steps.

Response: In our previous calculations, the contribution of pH values had already been taken into account. The Gibbs free energies were calculated using the computational hydrogen electrode (CHE) model. In this work, all potentials were referenced to the reversible hydrogen electrode (RHE). Since the RHE potential intrinsically depends on pH, the pH effect is implicitly included in the potential term and is therefore not neglected in our calculations.

For completeness, if the free energy change (ΔG) is expressed on the standard hydrogen electrode (SHE) scale, the pH effect should be explicitly included through a pH correction term as ΔG_{pH}. In contrast, when

the potential is referenced to RHE, the pH dependence is implicitly included in the reference potential. The relationship between ΔG referenced to SHE and RHE can be written as:

$$\Delta G(U_{\mathrm{SHE}}, \mathrm{pH}) = \Delta E + \Delta ZPE - T\Delta S + \Delta G_{\mathrm{pH}}$$

$$\Delta G(U_{\mathrm{RHE}}) = \Delta E + \Delta ZPE - T\Delta S$$

$$\Delta G(U_{\mathrm{RHE}}) = \Delta G(U_{\mathrm{SHE}}) + \Delta G_{\mathrm{pH}} = \Delta G(U_{\mathrm{SHE}}) + \frac{k_B \mathrm{TIn10}}{\mathrm{e}}\mathrm{pH}$$

where ΔG is the Gibbs free energy change, ΔE is the electronic energy difference, ΔZPE is the zero-point energy difference, and ΔS is the entropy change. The pH correction term is given by $\Delta G_{\mathrm{pH}} = \frac{k_B \mathrm{TIn10}}{e}\mathrm{pH}$, where k_B is the Boltzmann constant, *e* is electron transferred, and T = 300 K.

We acknowledge that the treatment of the pH effect was not sufficiently clarified in our previous version, which may have led to misunderstanding. We have now revised the corresponding content in the manuscript and Supplementary Information to explicitly state the pH contribution as follows: The free energies were referenced to U = 0 V vs. RHE, where the pH effect is inherently included in the reference potential.

Following the reviewer's valuable suggestion, we have further calculated the Gibbs free energy diagrams for OER reactions in dark and under light at two different biases, i.e., U = 0 V and 1.23 V vs. RHE (Figs.R1-6 and R1-7). By using the potential at 1.23 V vs. RHE, the overpotential of each step and the rate-limiting step can be more clearly shown. The step exhibiting the largest free energy change (ΔG) corresponds to the rate-limiting step. As shown in Fig.R1-6b and R1-7b at U = 1.23 V vs. RHE, the rate-limiting step shifts from *OOH formation in dark to proton transfer under light, consistent with our previous discussion.

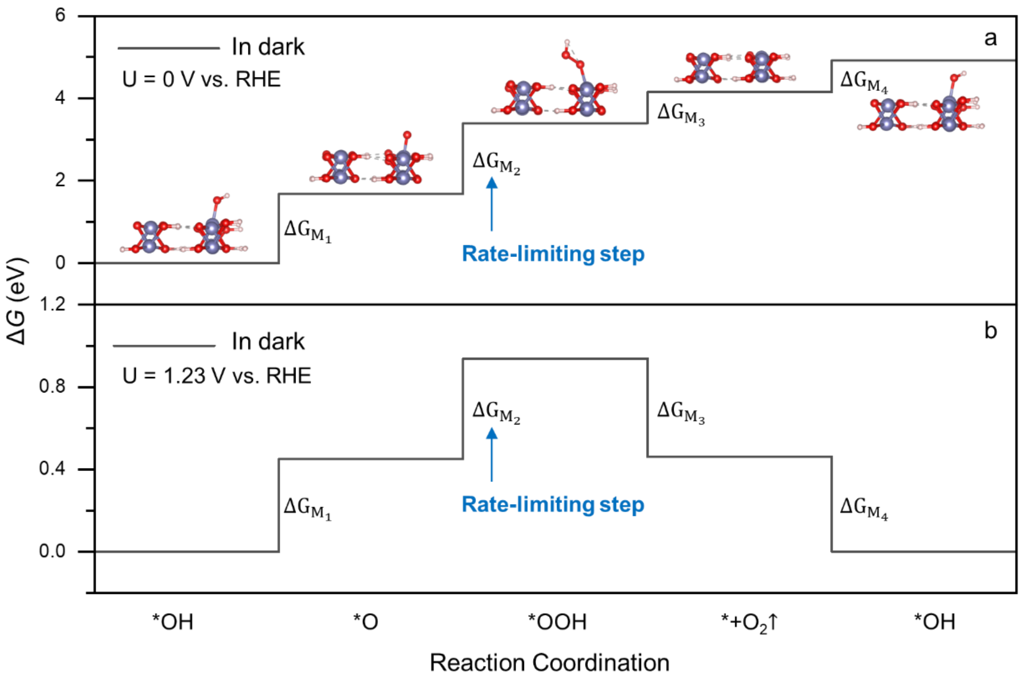

Fig.R1-6. Calculated reaction free energy diagrams of the OER pathway in dark (metal redox activity) on S-CoOOH at U = 0 V (**a**) and 1.23 V (**b**) vs. RHE. (Supplementary Fig.S37a in our previous Supplementary Information, Supplementary Fig.S37a,b in our revised Supplementary Information)

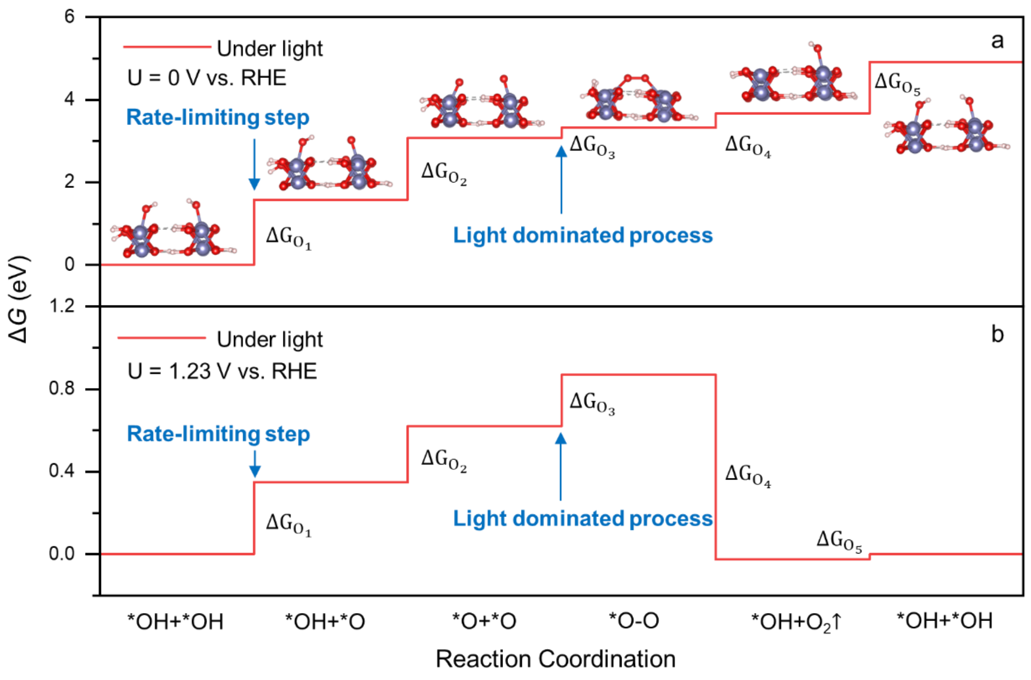

Fig.R1-7. Calculated reaction free energy diagrams of the OER pathway under light (oxygen redox activity) on S-CoOOH at U = 0 V (**a**) and 1.23 V (**b**) vs. RHE. (Supplementary Fig.S37b in our previous Supplementary Information, Supplementary Fig.S38a,b in our revised Supplementary Information)

To address the comment, we have incorporated the proposed modifications into our manuscript (lines 531-535, page 22, highlighted in yellow): The thermodynamic calculations were performed using the optPBE-vdW functional with the van der Waals (vdW) correction. The solvent effect was considered using the implicit solvation model implemented in the VASP model. The free energies were referenced to U = 0 V and 1.23 V vs. RHE, where the pH effect is inherently included in the reference potential. More detailed computational parameters are provided in Supplementary Information.

Moreover, we have revised the corresponding description of the thermodynamic calculations below Supplementary Figure S37: The thermodynamic calculations were performed using the optPBE-vdW functional with the van der Waals (vdW) correction. The Gibbs free energy change (ΔG) for each elemental step in OER can be obtained by the following formula:

$$\Delta G(U_{RHE}) = \Delta E + \Delta ZPE - T\Delta S$$

where ΔE is the electronic energy difference, ΔZPE is the zero-point energy difference, and ΔS is the entropy change. The relationship between ΔG referenced to standard hydrogen electrode (SHE) and reversible hydrogen electrode (RHE) can be described by a pH correction term: $\Delta G(U_{\mathrm{RHE}}) = \Delta G(U_{\mathrm{SHE}}) + \Delta G_{\mathrm{pH}}$, where $\Delta G_{\mathrm{pH}} = \frac{k_B \mathrm{TIn10}}{e}\mathrm{pH}$, with k_B is the Boltzmann constant and T = 300 K.

The associated electron transfer reactions in dark and under light are composed of the following elementary steps, respectively:

In dark (metal redox activity):

$\Delta G_{\mathrm{M}_1} = \Delta G_{[*\mathrm{O}]} - \Delta G_{[*\mathrm{OH}]} - \mathrm{eU}$

$\Delta G_{\mathrm{M}_2} = \Delta G_{[*\mathrm{OOH}]} - \Delta G_{[*\mathrm{O}]} - \mathrm{eU}$

$\Delta G_{\mathrm{M}_3} = 4.92\ \mathrm{eV} - \Delta G_{[*]} + \Delta G_{[*\mathrm{OOH}]} - \mathrm{eU}$

$\Delta G_{\mathrm{M}_4} = \Delta G_{[*\mathrm{OH}]} - \Delta G_{[*]} - \mathrm{eU}$

Under light (oxygen redox activity):

$\Delta G_{\mathrm{O}_1} = \Delta G_{[*\mathrm{OH}+*\mathrm{O}]} - \Delta G_{[*\mathrm{OH}+*\mathrm{OH}]} - \mathrm{eU}$

$\Delta G_{\mathrm{O}_2} = \Delta G_{[*\mathrm{O}+*\mathrm{O}]} - \Delta G_{[*\mathrm{OH}+*\mathrm{O}]} - \mathrm{eU}$

$\Delta G_{\mathrm{O}_3} = \Delta G_{[*\mathrm{O}-\mathrm{O}]} - \Delta G_{[*\mathrm{O}+*\mathrm{O}]}$ (Light dominated process)

$\Delta G_{O_4} = 4.92\ \mathrm{eV} - \Delta G_{[*OH]} + \Delta G_{[*O-O]} - \mathrm{eU}$

$\Delta G_{O_5} = \Delta G_{[*OH+*OH]} - \Delta G_{[*OH]} - \mathrm{eU}$

In addition, we have incorporated the proposed modifications into our manuscript (lines 262-264, page 12, highlighted in yellow): In addition to the kinetic processes discussed above, the thermodynamic processes under dark and light conditions are compared at U = 0 V and 1.23 V vs. RHE (Supplementary Figs.S37, S38).

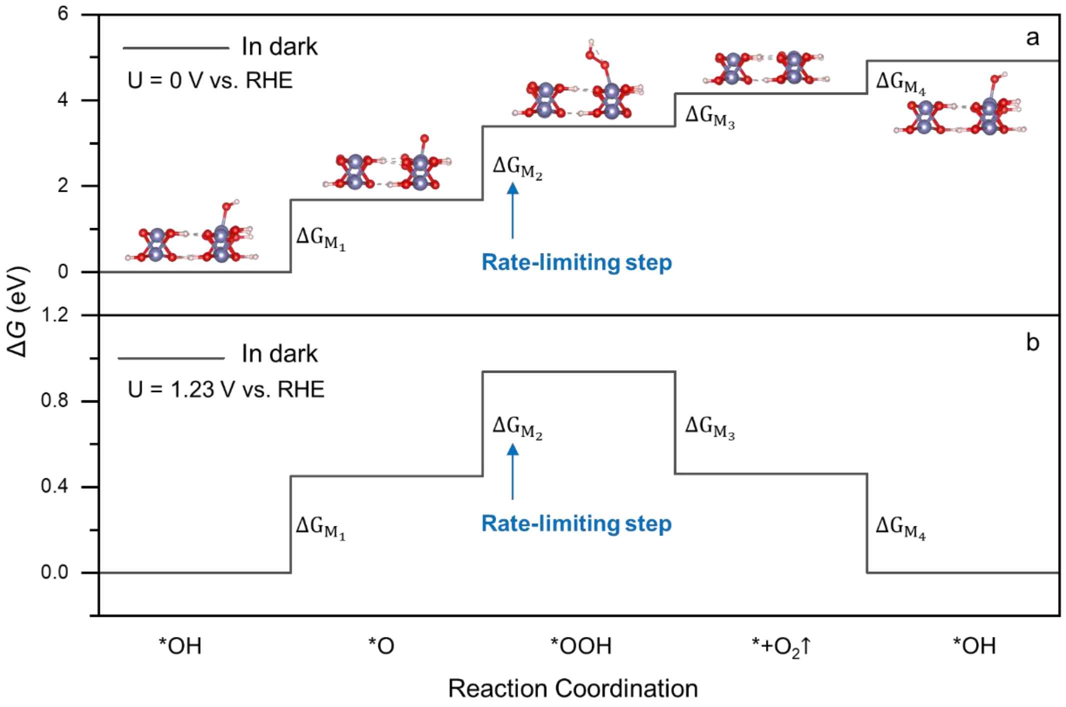

Supplementary Figure S37. Calculated reaction free energy diagrams of the OER pathway in dark (metal redox activity) on S-CoOOH at U = 0 V (**a**) and 1.23 V (**b**) vs. RHE.

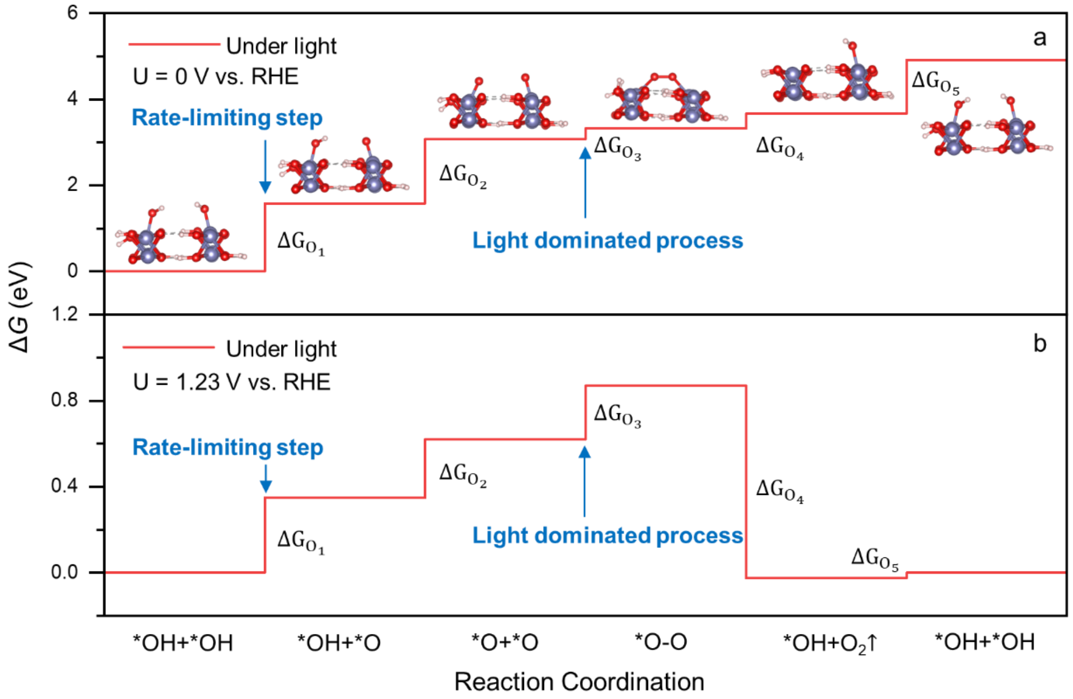

Supplementary Figure S38. Calculated reaction free energy diagrams of the OER pathway under light (oxygen redox activity) on S-CoOOH at U = 0 V (**a**) and 1.23 V (**b**) vs. RHE.

Reviewer #2

Remarks to the Author: publish as is

Response: We are sincerely grateful to the reviewer for the time and effort in assessing our manuscript. The reviewer's positive remarks and endorsement for publication are greatly appreciated.

Reviewer #3

Remarks to the Author: Authors have well readdressed the comments raised by the reviewers, now this manuscript can be accepted.

Response: We sincerely thank the reviewer for the thorough review and valuable feedback on our manuscript. We greatly appreciate the reviewer's positive evaluation of our revised manuscript. The reviwer's support and endorsement of our work are deeply appreciated.

The authors report a light-triggered LMCT from M–O to Co 3d orbitals at the edge sites of S-CoOOH. This photoinduced LMCT is proposed to enhance OER activity by facilitating the participation of O_{NB} sites in the formation of O–O intermediates during oxygen redox, effectively bypassing the rate-determining *OOH step in the conventional AEM. To elucidate the LMCT mechanism, the authors employed operando XAS and RIXS to probe the electronic structure at the edge of S-CoOOH. In addition, the OER mechanism involving O–O intermediate formation was investigated using ^{18}O labeling combined with operando DEMS. Overall, this study presents interesting mechanism and provides comprehensive characterization. However, publication can be considered after the following revisions are addressed.

1. The authors argue that light-triggered LMCT (from M–O to O) enhances OER activity, with no evidence that electrons from the M–O bonds are photo-excited.

2. S-CoOOH was prepared by oxidizing cobalt sulfides, but cobalt sulfides are photoelectrode materials. Have authors considered the remaining cobalt sulfides?

3. The LMCT has been triggered in S-CoOOH. What is photophysical property of the S-CoOOH to support the authors' explanation.

4. What potential was applied during the operando XAS? More experimental details needed.

5. Authors mentioned changes in the valence states of Co sites during the OER process. Structural reconstruction commonly happens in oxyhydroxides during OER, causing alterations of crystal structure. The bulk structure should be analyzed before and after the OER.

6. In Figure 2d, changes in the absorption edge are not properly presented. need to show clearly this region and the shifts.

7. TMA^+ was used as the O–O intermediate probe and intended to interact with O–O intermediates. TMA^+ likely alters the valence state of Co. Have direct reactivities of TMA^+ with surficial Co ions been considered?

8. In Figure 3d, the light was turned on after 10 minutes and $^{32}O_2$ increased after 20 minutes. Clarify discrepancy from the ^{18}O experiment.

9. In Figure 4, the color choice for atoms is confusing.

10. The proposed reaction mechanism suggests that hydroxyl plays a crucial role. Is this role specific to alkaline conditions? A comparative study with acidic conditions could enrich the paper further.
